# Tying up lots of loose ends: dealing with unpublished crystal structures

**DOI:** 10.1107/S2053229626006285

**Published:** 2026-06-25

**Authors:** William Clegg

**Affiliations:** a Chemistry, School of Natural and Environmental Sciences, Newcastle University, Newcastle upon Tyne, NE1 7RU, United Kingdom; University of Strathclyde, United Kingdom

**Keywords:** CSD Communication, Cambridge Structural Database, CSD, publication, crystal structure

## Abstract

Direct deposition of crystal structures into the Cambridge Structural Database as *CSD Communications* is a simple and effective means of bringing otherwise unpublished structural results into the public domain. This article of personal reflection describes the motivation and process of an extensive programme to achieve this aim for the unpublished results of a lifetime crystallographic career, with a summary of progress so far, illustrated by particular examples. It may serve as an encouragement and an illustrative model for others to do the same.

## Abstract 2

Over 2000 previously unpublished crystal structures have been deposited in the Cambridge Structural Database as *CSD Communications* in a project to make the results of public funding available to the scientific community.

## Introduction

Soon after taking formal retirement, I was invited to give a lecture at the British Crystallographic Association (BCA) Spring Meeting in 2010, held at Warwick University, in a parallel session devoted to the publication of crystallographic re­search. The title of the lecture was ‘*Reasons good and bad for not publishing crystal structures*’. It formed the basis of an article entitled ‘*Why do so many crystal structures remain unpublished?*’ in Issue Number 2 of the 2020 IUCr Newsletter (Clegg, 2020 ▸). As it attracted considerable attention, I offered the same topic as a European Crystallographic Association (ECA) virtual lunchtime seminar in January 2024, using the same title, and it prompted much discussion. Arising from this, the editor of *Crystallography Reviews*, who had been in the online audience, asked if I could contribute an article on the subject for publication there. With appropriate permission from the IUCr for copyright purposes, a revised and updated version became a review with the title ‘*Unpublished crystal structures: the hidden bulk of the iceberg?*’ (Clegg, 2024 ▸).

Among the reasons given in those presentations why successfully solved and refined crystal structures of publishable quality remain unpublished, a few are particularly relevant to the current article:

– the sheer number of results generated by contemporary structural re­search using powerful modern hardware and software overwhelms the manuscript production capability of most re­search groups and a selection, at best, of those available make their way into journal articles and other established forms of publication;

– key re­searchers involved in and responsible for the synthetic work leading to structural results develop new inter­ests and leave older ones behind, or they change employment, retire or die;

– re­search funding ends before publication can be achieved;

– the structural results are unexpected, not what was hoped for, or of insufficient novelty and inter­est;

– the structures raise questions, prompting further re­search effort to give a more complete overall result for publication;

– publication is attempted but is unsuccessful for reasons not directly related to the quality of the crystal structure result.

Substantial collections of crystal structures may also remain unpublished when the crystallographer responsible for the work retires or leaves re­search work for some other reason.

Publication of satisfactorily determined and refined crystal structures is to be encouraged for a variety of reasons outlined previously (Clegg, 2020 ▸; Clegg, 2024 ▸). These include the reputation of re­searchers and institutions, contribution to scientific knowledge and the avoidance of unnecessary duplicate work. Where the re­search has been supported by public funding, publication of results may also be considered a moral imperative, and it is increasingly a policy requirement of funding bodies in recent years with particular emphasis on Open Access publication. By ‘publication’ here, I mean any effective means of placing the results into the public domain, *i.e.* making it accessible to others outside the circle of those who have carried out the re­search; this may include not only traditional journal publication (whether in print, purely electronic or hybrid, and with or without peer review), but also conference presentations and abstracts, doctoral and other theses usually lodged in an institutional library, electronic document repositories, websites and accessible databases.

With a growing awareness of the likely massive and expanding horde of unpublished crystal structures as a result of major advances in crystallographic hardware and software, various attempts have been made to encourage and simplify the transfer of some of these into the public domain. These attempts include the introduction of new journals specifically for this purpose: the *New Crystal Structures* section of *Zeit­schrift für Kristallographie* in 1997 (*Zeitschrift für Kristallographie*, 1997 ▸) and *Acta Crystallographica Section E* in 2001 (Clegg & Watson, 2001 ▸). The impact has been modest at best, as significant time and effort are still needed to generate submissions in the prescribed format and satisfying editorial requirements.

Direct personal deposition of structures into databases is much simpler; the process has been streamlined in recent years and is actively encouraged by database managers. It is the primary mechanism for inclusion of structural results in the Crystallography Open Database (COD; Gražulis *et al.*, 2009 ▸). It has been available as an option for the Cambridge Structural Database (CSD; Groom *et al.*, 2016 ▸), for many years, originally using the designation *Private Communication*; more recently the term *CSD Communication* has been adopted. A common online inter­face has been introduced for depositions to both the CSD and the Inorganic Crystal Structures Database (ICSD; Zagorac *et al.*, 2019 ▸), and a Digital Object Identifier (DOI) is assigned for each deposited structure. Although a DOI is not a formal article reference recognized and abstracted by indexing services such as *Web of Science* and *Google Scholar*, it does provide a citable hyperlink reference as a form of publication.

When I formally retired in 2009, mainly giving up undergraduate teaching and administrative responsibilities while continuing with some re­search activities on a largely unpaid basis, I had around 2100 entries in the CSD, almost all of them corresponding to journal publications. I was convinced that this represented significantly fewer than the number of structural results of publishable quality remaining unpublished in filing cabinets and computer archives. Although some have subsequently been published in journal articles, the ma­jor­i­ty belonged in the various categories outlined above of structures unlikely to be included in future journal publications. I determined to work systematically through these, subject each to a further round of refinement using current software (some results were already many years old), making improvements where possible (such as treating previously unrecognized disorder, twinning or other problems), and deposit as many as possible as *CSD Communications*. This article describes the process, the progress to date and some specific examples of collections of related structures. It is intended as an encouragement and an illustrative model for others to do the same.

## The process

With a large field of candidate structures for deposition and the prospect of considerable time required for the exercise, some preliminary planning and organization was needed, together with the establishment of an efficient standard procedure to be followed as far as possible. These were identified as the steps involved.

– Set aside results that are part of ongoing re­search projects or for which there is a reasonable expectation of inclusion in a journal publication.

– Assign structures to sets of related results; in most cases, this means specific areas of chemical inter­est, mainly on the basis of individual collaborating re­search groups.

– Prioritize these sets to be tackled in turn; high on the list come the oldest structures and those resulting from collaborations with re­search group leaders who are no longer active for any reason, so publication is very unlikely.

– Within each set, generate a simple list or spreadsheet of candidate structures and check the availability of all required information, which usually means the refined structure already obtained, the processed diffraction data used in the refinement, possibly the original raw measured data if reprocessing is required, and relevant chemical, spectroscopic and other information. At this stage, a small proportion of structures had to be eliminated because of problems such as unreadable archive media or missing information. Check for duplication, both within the set of structures (this does happen occasionally) and with existing entries in the CSD. When an as-yet unpublished structure matches one already in the CSD, deposition is recommended if this represents a result of higher precision and/or an improved inter­pretation, for example, in cases of structural disorder or non-trivial assignment of H atoms.

– If at all possible, carry out a new structure refinement using current software. This is particularly important if the previous refinement did not generate a CIF satisfying present standards and matching the expectations of the CSD *Deposit Structures* inter­face. I have found that, particularly in the case of older structures, a new refinement can sometimes uncover previously unnoticed disorder or twinning effects, especially with appropriate use of restraints unavailable in earlier software, and it is possible to obtain a significantly improved structural model. For my own work, this has usually meant upgrading from older versions of *SHELXL*, *e.g. SHELXL97*, to the more recent *SHELXL2014* or *SHELXL2025* versions (Sheldrick, 2008 ▸; Sheldrick, 2015 ▸) with their far richer palette of restraints, treatment of twinning and other tools. Make use of the various _special_details fields in the CIF to provide information on non-routine aspects of the refinement. There may be cases where such an updated refinement is difficult, but deposition of older refined structures without corresponding diffraction data is much less satisfactory and will generate alerts that require response and justification. Comments are also recommended in the case of non-centrosymmetric structures for which Friedel pairs have been merged in the absence of significant resonant (also inappropriately called anomalous) scattering effects and the original raw data are no longer available for reprocessing; these may well generate alerts, including for a relatively poor data/parameter ratio.

– Deposit structures through the CSD website, preferably in batches for convenience and efficiency, and maintain a record of depositions, including refcodes and DOIs; this is conveniently done as a comprehensive spreadsheet. As part of the deposition, respond to the most serious *checkCIF* alerts (all level A and some level B as considered appropriate) (Spek, 2020 ▸) and complete as many additional data fields as possible (*e.g.* crystallization method and solvents, crystal colour and habit).

Prior to retirement in 2009, a small number of structures (around 40) had already been deposited in this way. The process began more seriously in 2014 after dealing with other concerns of higher priority and accelerated in the next few years. The period of imposed Covid lockdowns in the UK was particularly productive in this respect, as many other activities were prohibited and I was fortunate to have access to all relevant data and suitable computing facilities at home, having foreseen this eventuality. By the end of 2020, my number of *CSD Communications* exceeded 1000. It reached 2000 before the end of 2025.

## Progress and achievements

At the time of writing, the number of *CSD Communications* is 2065. This is believed to represent a large ma­jor­i­ty of refined structures of publishable quality in my personal collection that have not been published in journal articles and are unlikely to be so. They are structures determined in a wide range of col­lab­o­ra­tive re­search projects before about 2012. The ma­jor­i­ty of these are based on diffraction data collected in the X-ray crystallography laboratory at Newcastle University under my direction, with a smaller number using synchrotron data measured at the Daresbury Laboratory Synchrotron Radiation Source up to 2008 and Diamond Light Source between 2008 and 2012.

Collaborations have been with around 25 chemistry re­search groups at Newcastle University, around 15 groups elsewhere in the UK and half a dozen in other countries over a period of around 30 years. Apart from two particular cases considered in detail below, the number of structures associated with each re­search group varies from a handful to almost 200.

Most of the structures now remaining to be processed were determined between 2010 and 2012. These were not treated at the same time as others in their respective re­search group sets as there was still some prospect that they might be published relatively soon after they were obtained, but after the lapse of several more years this is now regarded as unlikely.

**Figure 1:**
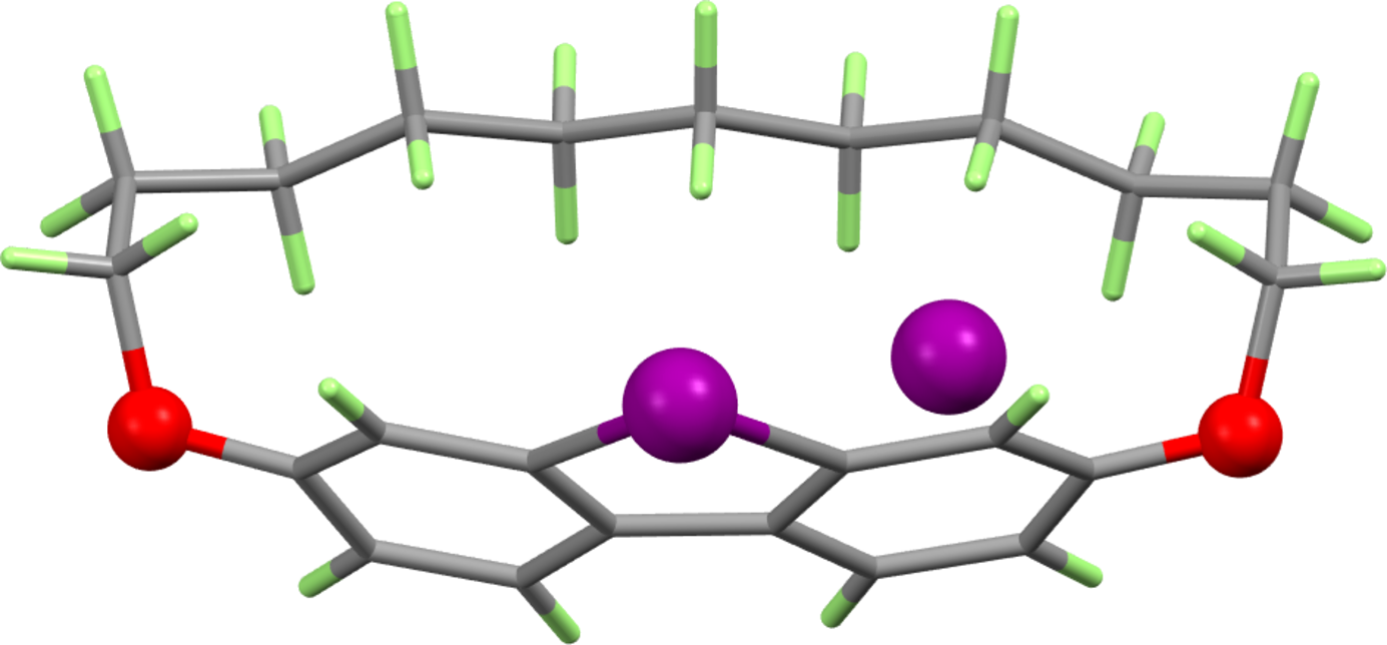
Refcode RUQCIS (doi:10.5517/ccdc.csd.cc259scn, CCDC 2005401), an iodo­lium iodide organic salt, C_23_H_28_IO_2_^+^·I^−^. In all figures, most atoms are shown in wireframe mode with grey C, pale green H, red O, blue N, pink B and darker green Cl. Colour code for atoms shown as spheres: red O and purple I.

Some representative examples of deposited structures are shown in Figs. 1 ▸–5 ▸ ▸ ▸ ▸ to illustrate the variety of chemistry and of the complexity of the structures.

Two particularly large groups of structures have received special attention as major col­lab­o­ra­tive exercises and will now be described.

## The structures of boron compounds and related materials from Leeds University

Collaboration with the re­search group of Professor John D. Kennedy at Leeds University began around the time when I led the major project to design, construct and commission a synchrotron single-crystal diffraction facility dedicated to chemistry and materials science, designated Station 9.8, at the Synchrotron Radiation Source (SRS) at Daresbury Laboratory. This project, funded by what was then the Science and Engineering Research Council (SERC) with significant financial, personnel and other resources support from the Council for the Central Laboratories of the Research Councils (CCLRC), began in 1994. Greatly assisted by my three-year Joint Appointment as a 50% seconded member of Daresbury Laboratory staff (con­tin­ued at a lower level in subsequent years up to 2008), the commissioning was completed on schedule in 1997 with a formal handover of the diffraction station from Newcastle University (as principal grant holder) to Daresbury Laboratory (Cernik *et al.*, 1997 ▸). Commissioning involved mainly the investigation of suitable samples, not amenable to standard in-house laboratory X-ray study, provided by a wide range of crystallographers and chemists in the UK and abroad. This included cluster metallaborane samples provided by the Kennedy re­search group, most of which were obtained as low-yield products consisting of very small air-sensitive crystals. One of these was among the first results from Station 9.8 to be published (Bould *et al.*, 1997 ▸). Several other similar samples were subsequently investigated at the SRS through Research Council-funded grants to Leeds and Newcastle and others were suitable for study in Newcastle; col­lab­o­ra­tive re­search of these groups over the last 30 years has led to 28 publications.

**Figure 6:**
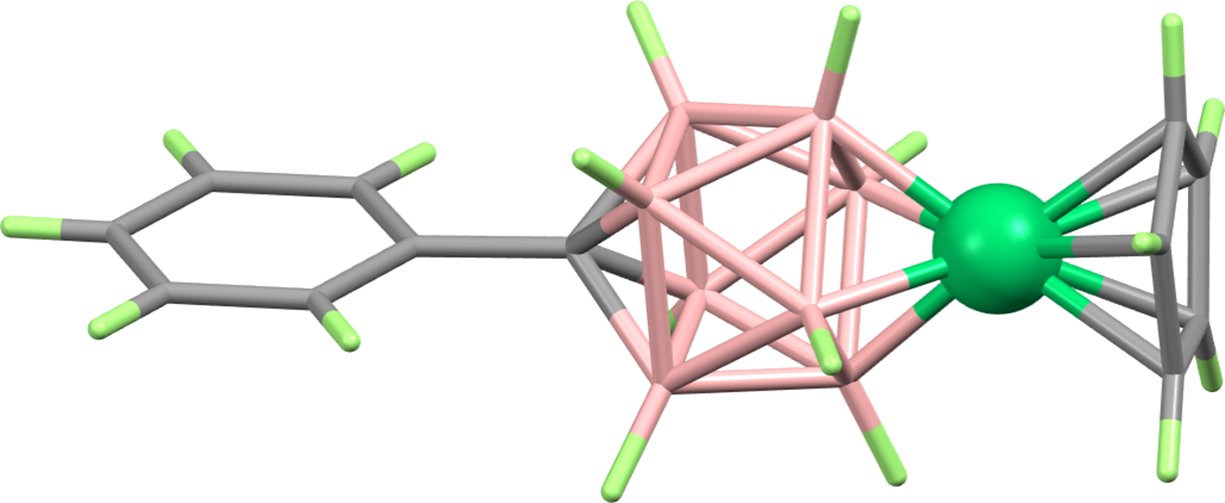
Refcode MARDUI (doi:10.5517/ccdc.csd.cc29xvrv, CCDC 2142898), a nickel carbaborane cluster, C_12_H_18_B_8_Ni. Colour code: green Ni.

At the retirement of both re­search group leaders, many other structures remained unpublished and we agreed to initiate a programme to convert as many as possible to *CSD Communications*, incorporating additional information, such as an indication of synthesis reactants and conditions, for the benefit of other re­searchers inter­ested in this chemical field. Other crystal structures of products of the Kennedy group, determined locally in Leeds, were also to be included, as the crystallographers involved were no longer available to carry out a parallel exercise. This began in 2021 and by the end of 2024 only a few structures remained, these presenting particular problems that have not yet been resolved. The number of deposited structures is currently 242. Some of these are shown in Figs. 6 ▸–8 ▸ ▸.

## The structures of main-group compounds from Strathclyde University

My long-term and very productive collaboration with inorganic chemists at Strathclyde University began while I was still working in the group of Professor George Sheldrick at the University of Göttingen in Germany in 1983. Professor Ken Wade at Durham University introduced me to one of his former PhD students, Dr Ron Snaith, who had set up a new re­search group at Strathclyde and was producing some very inter­esting organolithium compounds that had potentially intriguing structures, as evidenced by their spectroscopic and other properties. At the time, we had particularly good crystallographic facilities in Göttingen with capacity to take on a significant new project. A year later I moved to Newcastle and established my own re­search group with a new SERC-funded diffractometer and was able to continue the collaboration. That was a particularly productive year for publications from this work, seven appearing in *J. Chem. Soc. Chem. Commun.* during 1984. There were over 30 more, in a range of journals, over the next 20 years.

**Figure 9:**
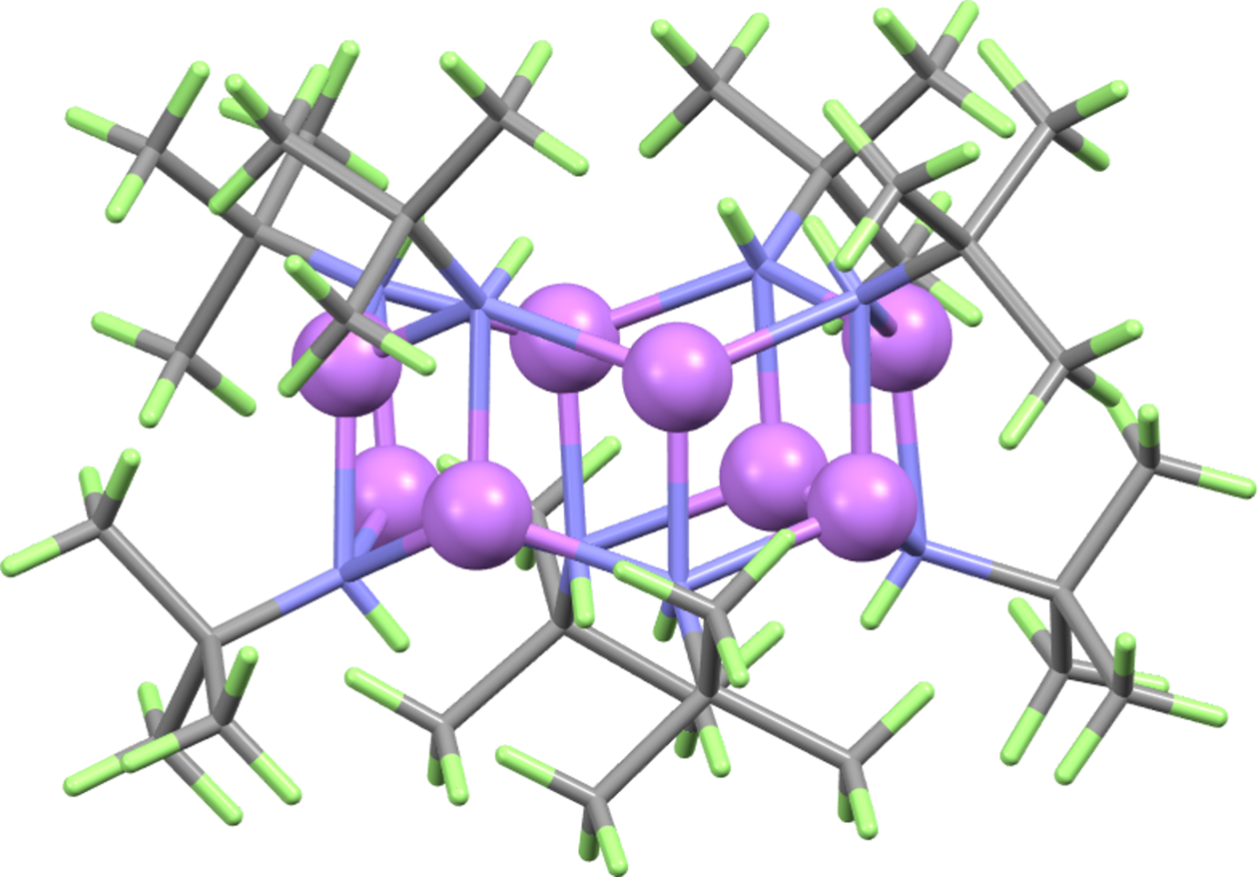
Refcode LUQCEJ (doi:10.5517/ccdc.csd.cc2n3g08, CCDC 2446179), a drum-shaped lithium amide octa­mer, C_32_H_80_Li_8_N_8_. Colour code: pink Li.

One of Ron Snaith’s PhD students at the beginning of our collaboration was Robert (Rab) E. Mulvey, who con­tin­ued with main-group chemistry re­search at Strathclyde when Snaith moved to Cambridge in 1986. He expanded the scope of this re­search to heavier alkali metals and then to other main-group metals. Many Mulvey samples came from Strathclyde to Newcastle and the re­search collaboration con­tin­ued to be highly productive, with almost 100 joint publications not including those with Ron Snaith. Even so, this represents probably under half the number of successfully determined and refined structures (a full catalogue has not yet been generated, as this particular exercise is currently underway). In 2025 we agreed to begin a programme similar to the one with John Kennedy in Leeds, which by then was almost complete. A total of 70 structures were deposited during 2025 and the project continues. Examples of *CSD Communications* from this work are shown in Figs. 9 ▸ and 10 ▸. These structures, many of them unusual and fascinating, would probably never make their way into the public domain without this post-retirement deposition exercise.

## Some practical considerations

The question may be asked, why deposit these unpublished structures in the CSD, which requires a licence for full access to its contents, rather than the COD, which provides fully open access to all? There are several reasons.

Essentially all published organic and metal–organic structures are captured in the CSD, whereas only some journals feed results into the COD, which relies otherwise on voluntary deposition by re­searchers; the CSD is thus much more com­pre­hen­sive. The inter­face for depositing structures in the CSD (serving also the ICSD for appropriate structures, few of which have been encountered in this work) provides a more extensive and detailed validation of the results, including manual curation, and it also assigns a DOI to each submitted CIF, making the deposited structure easily and uniquely citable. It generates a ‘2D view’, a conventional chemical structure diagram that is not provided by structures in the COD and is of particular inter­est to chemists browsing the database contents, especially when making use of substructure matching approaches; this diagram is automatically created during deposition and is checked and potentially modified as part of the manual curation (Bruno *et al.*, 2011 ▸; Holgate, 2019 ▸). The complete suite of CSD software includes other useful components for validation and statistical analysis to which *CSD Communications* contribute. The managers of the CSD (the Cambridge Crystallographic Data Centre, CCDC, a not-for-profit organization registered as a UK charity) aim to maximize access by providing not only individual institutional licences (with different prices for educational and commercial users), but also licences on a national level by negotiation depending on economic circumstances. It should also be noted that the CSD *Access Structures* inter­face (https://www.ccdc.cam.ac.uk/structures/) provides licence-free viewing and downloading of individual entries, which may be found using limited searching options, as well as directly through the refcode, CCDC deposition number or DOI.

A further question relates to authorship, which is linked to the ownership of intellectual property rights. This can be a thorny issue and overlaps with other factors such as employment contracts, accountability and funding. Some of my own re­search work has been carried out on a commercial basis, particularly in collaboration with pharmaceutical companies, usually under terms of non-disclosure agreements, and here clearly the intellectual property belongs to the commercial customer. I take the same approach to work that has had direct costs fully covered by a chemist supplying samples, but there are few examples of this in my own experience.

In other cases, the principle to be applied is the ethical standard on authorship asserted and applied by most pub­lish­ers of scientific journals: authors should be those who have made *significant direct* contribution to the work being presented. As a *CSD Communication* reports a crystal structure with, in most cases, virtually no information on chemical synthesis, properties or reactions, the appropriate authors are those, whether recognized crystallographers or chemists with supervision or training, who have contributed to the specifically crystallographic rather than synthetic aspects. This is the approach taken here.

Finally, it should be reiterated that the primary objective of this major exercise is to bring the results of re­search supported by public funding into the public domain where they can be seen and used by others with general benefit rather than being lost for ever as a tragic waste of resources and effort. It is heartening to observe that a similar approach has been adopted by other crystallographers across the world, with a steadily increasing number and proportion of *CSD Communications* year on year. The CCDC issues annual statistical summaries of the CSD contents (CCDC, 2026 ▸), and those for 1 January 2026 show just over 72500 *CSD Communications*, exceeding the contributions from all but two individual scientific journals and more than 5% of the entire database (5.07% in 2026, 4.75% in 2025, 4.37% in 2024, 4.09% in 2023, *etc*.). 58 persons are authors of over 200 *CSD Communications*, 20 with over 500 and 9 with over 1000.

## Supplementary Material

Table of CSD depositions. DOI: 10.1107/S2053229626006285/ky3234sup1.pdf

## Figures and Tables

**Figure 2:**
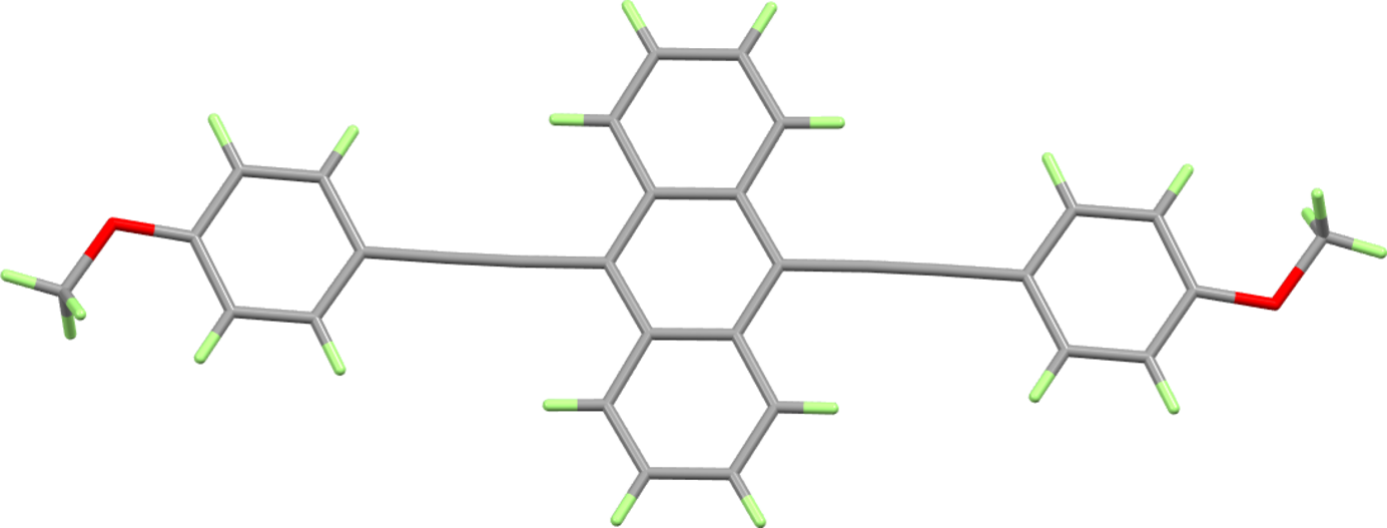
Refcode YEKVUI (doi:10.5517/ccdc.csd.cc7vs83, CCDC 234275), a con­jug­ated diyne rigid rod mol­ecule, C_32_H_22_O_2_.

**Figure 3:**
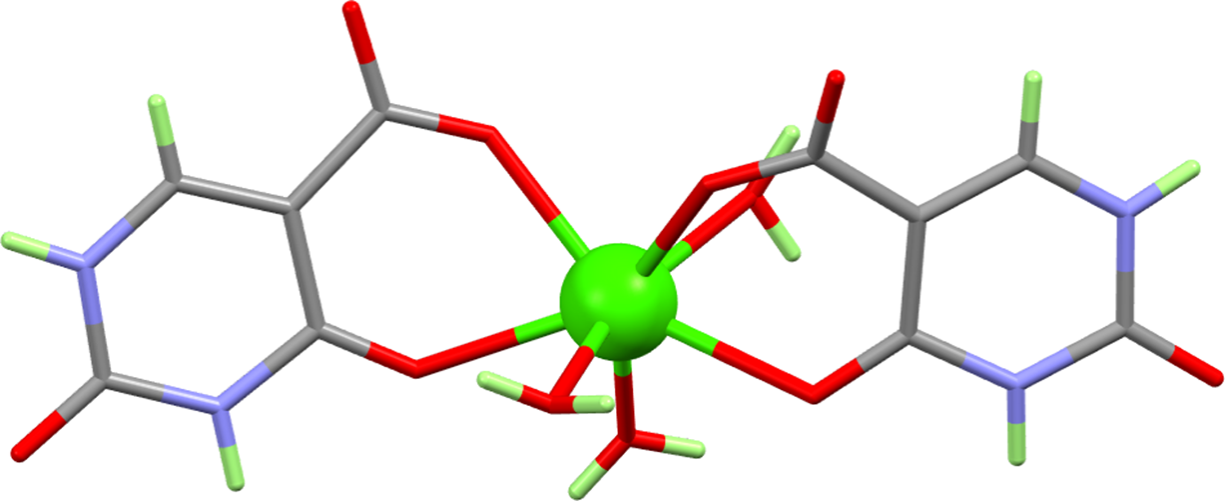
Refcode AROZOZ (doi:10.5517/ccdc.csd.cc27scn2, CCDC 2079004), a calcium complex, C_10_H_12_CaN_4_O_11_. Colour code: green Ca.

**Figure 4:**
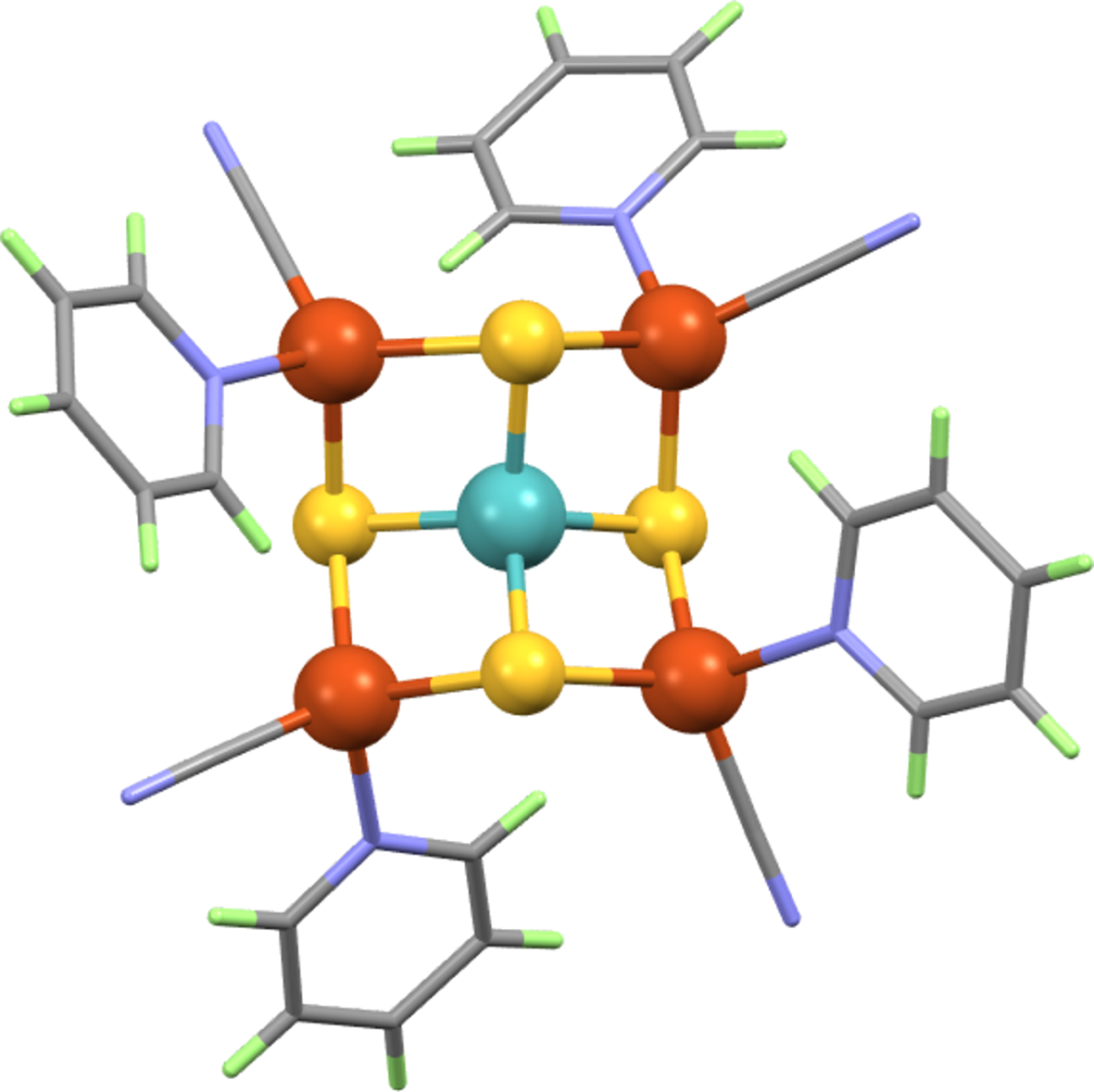
Refcode ZAKPIN (doi:10.5517/ccdc.csd.cc1nfph8, CCDC 1532500), the anion of a copper–molybdenum sulfur cluster complex, (NH_4_^+^)_2_[C_24_H_20_Cu_4_MoN_8_S_4_]^2−^. Colour code: yellow S, turquoise Mo and red–brown Cu.

**Figure 5:**
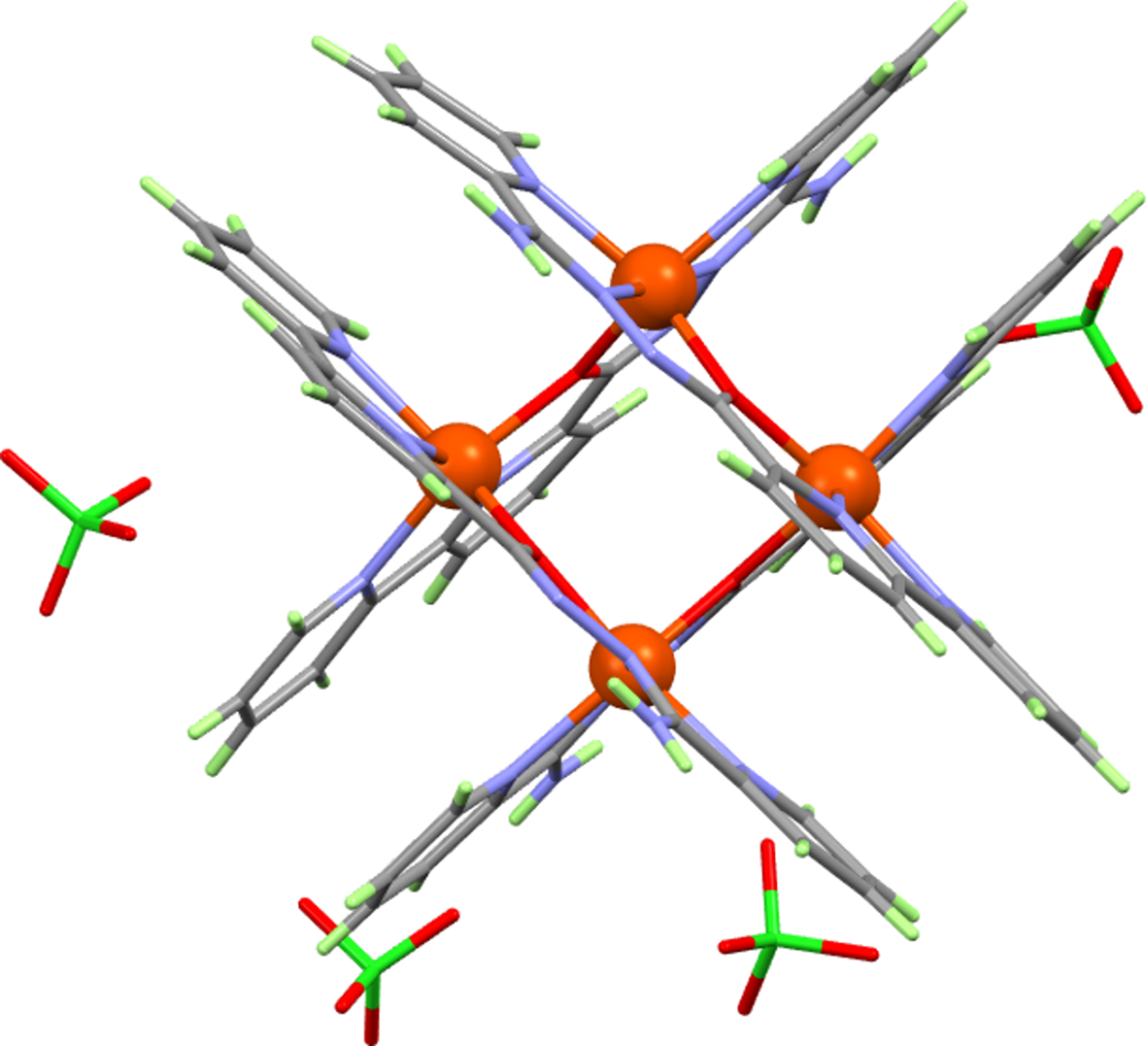
Refcode LOZCAH (doi:10.5517/ccdc.csd.cc23xj0l, CCDC 1963819), the simplest of a series of ligand-bridged metal grids, [C_68_H_52_Fe_4_N_24_O_4_]^6+^(ClO_4_^−^)_6_ (plus unidentified disordered solvent). Colour code: orange–red Fe.

**Figure 7:**
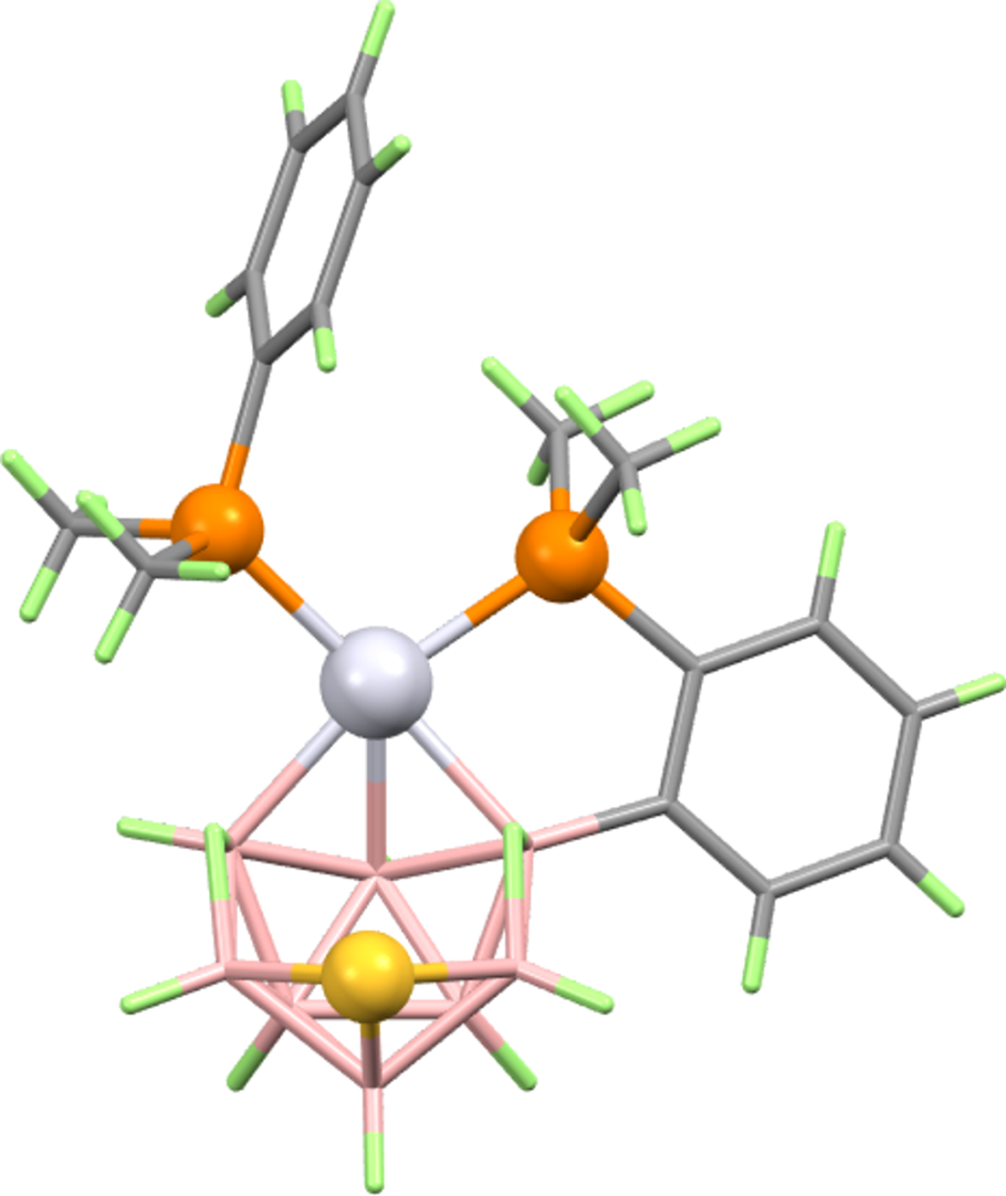
Refcode TISZUU (doi:10.5517/ccdc.csd.cc2h67lk, CCDC 2299908), a platinum thia­borane cluster, C_16_H_30_B_8_P_2_PtS. Colour code: yellow S, orange P and silver Pt.

**Figure 8:**
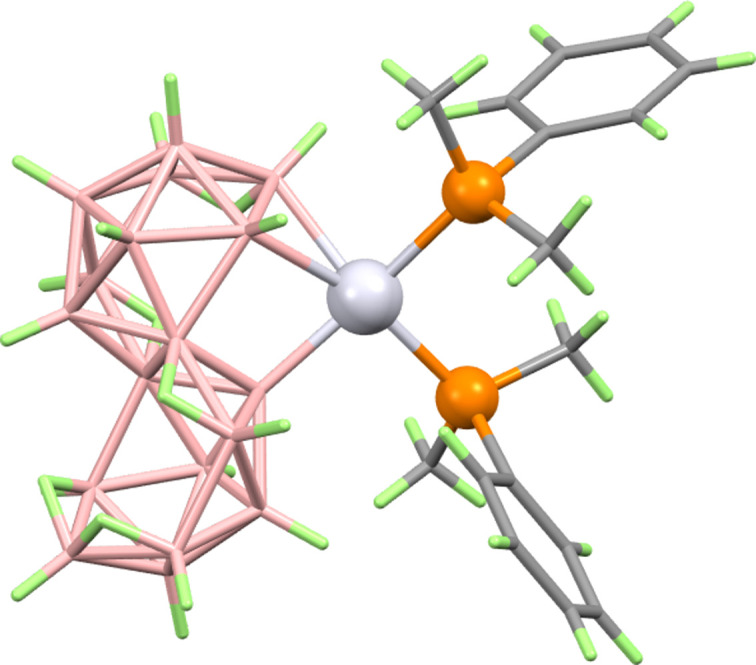
Refcode DAWFIU (doi:10.5517/ccdc.csd.cc2b8hbg, CCDC 2153115), a platinum-containing fused borane double cluster, C_16_H_42_B_18_P_2_Pt. Colour code: orange P and silver Pt.

**Figure 10:**
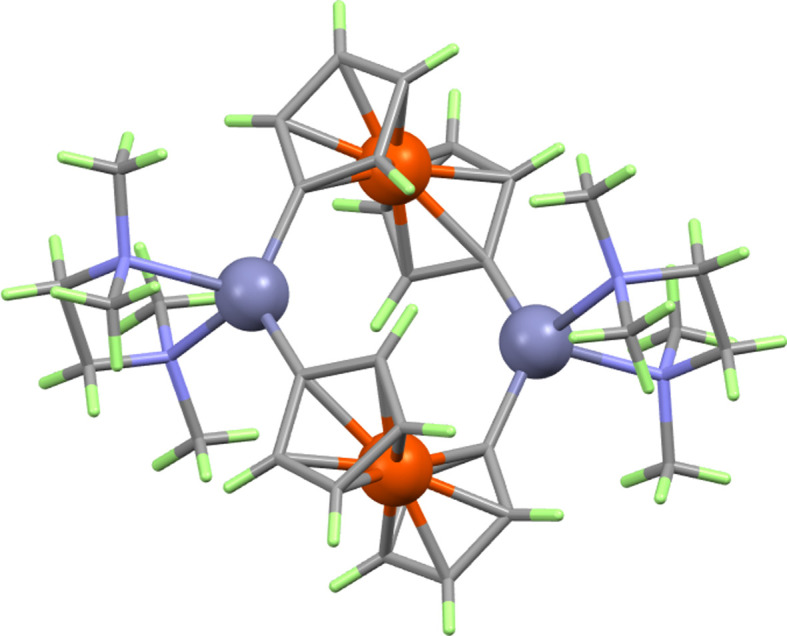
Refcode OQIDIF (doi:10.5517/ccdc.csd.cc2pzqfv, CCDC 2502178), a zinc-bridged diferrocene complex, C_32_H_48_Fe_2_N_4_Zn_2_. Colour code: orange Fe and blue-grey Zn.

## Data Availability

A complete tabulated list of all *CSD Communications* with W. Clegg as author is provided as supporting information for this article. The information includes, for each structure, the CCDC deposition number, Refcode, DOI, Authors, Source of material and Chemical formula, enabling all structures to be accessed in the CSD for viewing and downloading.

## References

[bb1] Bould, J., Clegg, W., Kennedy, J. D., Teat, S. J. & Thornton-Pett, M. (1997). *J. Chem. Soc. Dalton Trans.* pp. 2005–2008.

[bb2] Bruno, I. J., Shields, G. P. & Taylor, R. (2011). *Acta Cryst.* B**67**, 333–349. 10.1107/S0108768111024608 PMC3143025 21775812

[bb3] CCDC (2026). https://www.ccdc.cam.ac.uk/support-and-resources/documentation-and-resources (accessed on 1 June 2026).

[bb4] Cernik, R. J., Clegg, W., Catlow, C. R. A., Bushnell-Wye, G., Flaherty, J. V., Greaves, G. N., Burrows, I., Taylor, D. J., Teat, S. J. & Hamichi, M. (1997). *J. Synchrotron Rad.* **4**, 279–286. 10.1107/S090904959701008X 16699241

[bb5] Clegg, W. (2020). https://www.iucr.org/news/newsletter/volume-28/number-2/why-do-so-many-crystal-structures-remain-unpublished.

[bb6] Clegg, W. (2024). *Crystallogr. Rev.* **30**, 208–225.

[bb7] Clegg, W. & Watson, D. G. (2001). *Acta Cryst.* E**57**, e1–e2.

[bb8] Gražulis, S., Chateigner, D., Downs, R. T., Yokochi, A. F. T., Quirós, M., Lutterotti, L., Manakova, E., Butkus, J., Moeck, P. & Le Bail, A. (2009). *J. Appl. Cryst.* **42**, 726–729. 10.1107/S0021889809016690 PMC3253730 22477773

[bb9] Groom, C. R., Bruno, I. J., Lightfoot, M. P. & Ward, S. C. (2016). *Acta Cryst.* B**72**, 171–179. 10.1107/S2052520616003954 PMC4822653 27048719

[bb10] Holgate, S. (2019). https://www.ccdc.cam.ac.uk/discover/blog/csd-data-curation-the-human-touch/ (accessed on 1 June 2026).

[bb11] Sheldrick, G. M. (2008). *Acta Cryst.* A**64**, 112–122. 10.1107/S0108767307043930 18156677

[bb12] Sheldrick, G. M. (2015). *Acta Cryst.* C**71**, 3–8.

[bb13] Spek, A. L. (2020). *Acta Cryst.* E**76**, 1–11. 10.1107/S2056989019016244 PMC6944088 31921444

[bb14] Zagorac, D., Müller, H., Ruehl, S., Zagorac, J. & Rehme, S. (2019). *J. Appl. Cryst.* **52**, 918–925. 10.1107/S160057671900997X PMC6782081 31636516

[bb15] *Zeitschrift für Kristallographie* (1997). *Z. Kristallogr. New Cryst. Struct.* **212**, XVI–XVIII.

