# Supplementary material for: Tying up lots of loose ends: dealing with unpublished crystal structures
Source: Acta Crystallogr C Struct Chem. 2026 Jun 25;82(Pt 7):303–9. doi: 10.1107/S2053229626006285 (PMC13330907; doi:10.1107/S2053229626006285)
Supplement: Supplementary file 1 [file c-82-00303-sup1.pdf]

Table S1 CSD Communications of W. Clegg.

The entries in the table are sorted on CCDC deposition number (column 1).

CCDC	REFCODE	DOI	Authors	Source	Chemical formula
168723	GUMGON	10.5517/ccdc.csd.cc5nkp1	W.Clegg	Newcastle	C8 H21 N3 O2 2+,0.5(H2 O1),2(Cl1 1-)
199211	WUBDUV	10.5517/ccdc.csd.cc6p95b	W.Clegg	UK	C39 H45 Cl1 Co1 N3 1+,C24 H20 B1 1-,C1 H2 Cl2,H2 O1
214748	HEDXUM	10.5517/ccdc.csd.cc76gc7	W.Clegg	UK	C13 H12 F6 Si1
214749	HEFDEE	10.5517/ccdc.csd.cc76gd8	W.Clegg, L.Horsburgh	UK	C30 H30 B2 F12 O4
214750	HEFLIQ	10.5517/ccdc.csd.cc76gf9	W.Clegg, L.Horsburgh	UK	C30 H14 B2 F12 O4
214751	HEFLEM	10.5517/ccdc.csd.cc76ggb	W.Clegg, L.Horsburgh	UK	C30 H10 B1 F17 O2 Zn1
234275	YEKVUI	10.5517/ccdc.csd.cc1pw3th	W.Clegg, A.J.Scott	UK	C32 H22 O2
244781	WOXNAB	10.5517/ccdc.csd.cc86q5b	W.Clegg	UK	C50 H58 B2 Ge1
283104	IQAJOZ01	10.5517/cc9hldr	W.Clegg	International	C19 H15 1+,C30 B2 Cl1 F24 1-
283647	SAWSUF	10.5517/cc9j4xv	W.Clegg	Newcastle	C19 H19 Br1 O1
283648	XBPYRC02	10.5517/cc9j4yw	W.Clegg, M.R.J.Elsegood	Newcastle	C20 H20 Br6 Cu4 N4 O1
283649	SAWTAM	10.5517/cc9j4zx	W.Clegg, M.R.J.Elsegood	Newcastle	C4 H17 N4 O2 Re1 2+,2(Cl1 O4 1-)
283650	HODXOP	10.5517/cc9j50z	W.Clegg, M.R.J.Elsegood	Newcastle	C10 H16 N2 O2
283651	WERROA01	10.5517/cc9j510	W.Clegg, M.R.J.Elsegood	Newcastle	C19 H17 N1
283652	SAWTEQ	10.5517/cc9j521	W.Clegg, M.R.J.Elsegood	Newcastle	C12 H36 O6 S6 Zn1 2+,2(C1 F3 O3 S1 1-)
283653	SAWTIU	10.5517/cc9j532	W.Clegg, M.R.J.Elsegood	Newcastle	C16 H13 N3 O1 S1
283654	SAWTOA	10.5517/cc9j543	W.Clegg, M.R.J.Elsegood	Newcastle	C16 H13 N3 S2
283655	SAWTUG	10.5517/cc9j554	W.Clegg, A.J.Scott	Newcastle	C15 H10 Br1 N3
283656	SAWVAO	10.5517/cc9j565	W.Clegg	UK	C16 H13 N1 O4
283657	SAWVES	10.5517/cc9j576	W.Clegg	UK	C16 H8 N2 O4
283658	SAWVIW	10.5517/cc9j587	W.Clegg	UK	C15 H11 N1 O3
283659	SAWVOC	10.5517/cc9j598	W.Clegg	UK	C14 H8 F1 N1 O2
284020	SAWVUI	10.5517/cc9jjy8	W.Clegg, A.McCamley	International	C28 H48 Si4 Th1
284021	SAWWAP	10.5517/cc9jjz9	W.Clegg, A.McCamley	International	C17 H33 Si2 Ta1
284022	SAWWET	10.5517/cc9jk0c	W.Clegg, P.A.O'Neil	Newcastle	C29 H38 O5 S1 Si1
284023	SAWWIX	10.5517/cc9jk1d	W.Clegg	UK	C14 H20 Cl2 N4 S4
284024	SAWWOD	10.5517/cc9jk2f	W.Clegg, M.R.J.Elsegood	Newcastle	C5 H12 N1 O3 P1 S1,H2 O1
284025	SAWWUJ	10.5517/cc9jk3g	W.Clegg, M.R.J.Elsegood	Newcastle	Na1 1+,C5 H10 N1 O3 S2 1-,H2 O1
284026	SAWXAQ	10.5517/cc9jk4h	W.Clegg, P.A.O'Neil	UK	C20 H46 F6 Li2 N6 O6 S2
284708	SAWXEU	10.5517/cc9k847	W.Clegg	UK	C13 H8 N2 O2
284709	SAWXIY	10.5517/cc9k858	W.Clegg, C.Dalby	UK	C14 H8 Cl1 N1 O2
284710	VOLJOV01	10.5517/cc9k869	W.Clegg	UK	C16 H24 O4
284712	SAWXOE	10.5517/cc9k88c	W.Clegg, M.R.J.Elsegood	UK	C15 H11 N1 O3
284713	SAWXUK	10.5517/cc9k89d	W.Clegg, M.R.J.Elsegood	UK	C15 H11 N1 O2
284714	SAZQIU	10.5517/cc9k8bf	W.Clegg, M.R.J.Elsegood	UK	C14 H8 N2 O4
284715	SAWYAR	10.5517/cc9k8cg	W.Clegg	UK	C15 H8 N2 O2
286367	HAYQII01	10.5517/cc9lznh	W.Clegg, A.J.Scott	Newcastle	C24 H20 P1 1+,Br1 1-,C1 H2 Cl2
286368	REVKUZ	10.5517/cc9lzpj	W.Clegg, R.A.Coxall	International	C16 H20 N2 S2

286369	REVLAG	10.5517/cc9lzqk	W.Clegg, M.R.J.Elsegood, N.A.Hously	Newcastle	C8 H15 N1 O2
286370	REVLEK	10.5517/cc9lzrl	W.Clegg, M.R.J.Elsegood	Newcastle	C16 H36 N1 1+,C12 H10 O2 P1 1-
286371	REVLIO	10.5517/cc9lzsm	W.Clegg, D.C.R.Hockless, M.R.J.Elsegood	Newcastle	3(C16 H36 N1 1+),Mo12 O40 S1 3-,0.5(C2 H3 N1)
286372	REVLOU	10.5517/cc9lztn	W.Clegg, R.W.Harrington	Newcastle	4(C16 H36 N1 1+),O32 W10 4-,2(C2 H3 N1)
286373	REVLUA	10.5517/cc9lzvp	W.Clegg, R.W.Harrington	Newcastle	C21 H15 N3 O3,2(C1 H2 Cl2)
286374	REVMAH	10.5517/cc9lzwq	W.Clegg, A.J.Scott, M.R.J.Elsegood	Newcastle	C24 H27 O1 P1
288250	VECVAB	10.5517/cc9nyd8	W.Clegg	UK	C9 H20 N1 1+,B1 F4 1-
288251	VECVEF	10.5517/cc9nyf9	W.Clegg, P.A.O'Neil	Newcastle	C5 H8 O2
288252	VECVIJ	10.5517/cc9nygb	W.Clegg	UK	C14 H14 N1 1+,Cl3 Sn1 1-
288253	VECVOP	10.5517/cc9nyhc	W.Clegg, M.R.J.Elsegood	Newcastle	C6 H16 O2 P2,2(C5 H6 N1 1+),2(I1 1-)
288254	VECVUV	10.5517/cc9nyjd	W.Clegg, M.R.J.Elsegood, L.Horsburgh	Newcastle	C13 H14 P1 1+,B1 F4 1-
288255	VECWAC	10.5517/cc9nykf	W.Clegg	UK	C24 H20 P1 1+,C4 H10 Cl4 O2 W1 1-
288256	VECWEG	10.5517/cc9nylg	W.Clegg	UK	C16 H16 B1 N1
643418	LOZRUQ	10.5517/ccdc.csd.ccpljf6	S.Nlate, S.Shimada, L.Horsburgh, W.Clegg, P.S.Smith, A.S.Batsanov, J.A.K.Howard, S.Collins, W.E.Piers, T.B.Marder	UK	C24 H24 F12 Sn2
643419	LOZROK	10.5517/ccdc.csd.ccpljg7	S.Nlate, S.Shimada, L.Horsburgh, W.Clegg, P.S.Smith, A.S.Batsanov, J.A.K.Howard, S.Collins, W.E.Piers, T.B.Marder	UK	C22 H18 Cl2 F12 Sn2
686629	EYEDET	10.5517/ccdc.csd.cc1lcg1g	W.Clegg, R.W.Harrington	Newcastle	C16 H17 N1 O4
686630	YAFQII	10.5517/ccdc.csd.ccr1hcm	W.Clegg, R.W.Harrington	Newcastle	C13 H15 N1 O4
779309	TAZKUE	10.5517/ccdc.csd.ccv4y0x	W.Clegg, R.W.Harrington	Newcastle	C48 H48 N6,2(C1 H1 Cl3)
928043	YEKVIW	10.5517/ccdc.csd.cc104pwq	W.Clegg, A.J.Scott	UK	C16 H14 O2
969417	LOHVEK01	10.5517/cc11jrjv	W.Clegg, U.Baisch, M.R.Probert	Newcastle	C15 H12 S4
986550	GICXAV	10.5517/ccdc.csd.cc123l6z	W.Clegg, R.W.Harrington	Newcastle	C23 H30 N3 3+,3(Cl1 1-)
996574	HODXUV	10.5517/cc12g0k3	W.Clegg	UK	C13 H8 N2 O3
996575	HODYAC	10.5517/cc12g0l4	W.Clegg	UK	C14 H4 F5 N1 O2
996576	GOGBUB	10.5517/cc12g0m5	W.Clegg	UK	C13 H9 N2 O2 1+,Cl1 O4 1-
997234	HOGKOF	10.5517/cc12gpv2	W.Clegg, M.R.Probert, R.A.Henderson, A.Alwaaly	Newcastle	C68 H66 Ni1 O6 P6 2+,2(C24 H20 B1 1-)
997236	HOGKUL	10.5517/cc12gpx4	W.Clegg, M.R.Probert, R.A.Henderson, A.Alwaaly	Newcastle	C10 H16 N1 1+,0.75(C2 H3 N1),C24 H20 B1 1-
997242	ISUSIY01	10.5517/cc12gq3c	W.Clegg	International	C10 H18 N2 S2
997250	GEKVAT01	10.5517/cc12gqcm	W.Clegg	International	C14 H24 N2 S2
997251	GOGCEM	10.5517/cc12gqdn	W.Clegg	International	C10 H21 N2 S2 1+,C6 H2 N3 O7 1-
997460	GOGCIQ	10.5517/cc12gy4m	W.Clegg	International	C68 H98 Ag5 B2 F8 P4 Pt2 1+,B1 F4 1-,C3 H6 O1
997464	CIWKUQ	10.5517/cc12gy8r	W.Clegg	International	C68 H98 Ag5 B2 F8 P4 Pt2 1+,C6 H14,B1 F4 1-
997465	GOFQOJ	10.5517/cc12gy9s	W.Clegg	International	C80 H116 Ag6 P4 Pt2 2+,2(C3 H6 O1),2(B1 F4 1-)
998670	COBYEZ	10.5517/cc12j65z	W.Clegg	Newcastle	C10 H14 Br2 O2
998674	VAFSED	10.5517/cc12j693	W.Clegg	Newcastle	C21 H20 O5
998675	VAFSIH	10.5517/cc12j6b4	W.Clegg	Newcastle	C14 H28 O2 Si2

998676	COBYID	10.5517/cc12j6c5	W.Clegg	Newcastle	C12 H14 I2 O2
998677	TOHJIJ02	10.5517/cc12j6d6	W.Clegg	UK	C11 H11 N1 O3 S1
998678	COBYOJ	10.5517/cc12j6f7	W.Clegg	UK	C17 H12 N4 O1
998679	COBZUQ	10.5517/cc12j6g8	W.Clegg, A.J.Edwards	UK	C66 H88 Li4 N10,3(C7 H8)
998680	COBZAW	10.5517/cc12j6h9	W.Clegg, A.J.Edwards	UK	C36 H52 Br1 Li3 N6,0.5(C7 H8)
998681	COBYUP	10.5517/cc12j6jb	W.Clegg, A.J.Edwards	UK	C24 H42 I1 Li2 N5
998682	VAFSUT	10.5517/cc12j6kc	W.Clegg, A.J.Edwards	UK	C24 H42 Br1 Li2 N5
998683	VAFTAA	10.5517/cc12j6ld	W.Clegg, L.Horsburgh	UK	C24 H42 Cl1 Li2 N5
998684	KEKCOS01	10.5517/cc12j6mf	W.Clegg, K.A.Fraser	International	C62 H72 N2 P4 Pd2 S2 2+,2(C24 H20 B1 1-)
998685	COBZEA	10.5517/cc12j6ng	W.Clegg, K.A.Fraser	International	C72 H132 Cl2 Hg7 S12
998686	COBZIE	10.5517/cc12j6ph	W.Clegg, K.A.Fraser	International	C72 H132 Hg7 I2 S12
1000736	ZZZEQK01	10.5517/cc12lbtt	W.Clegg, A.J.Edwards	Newcastle	C14 H14 O4 S2
1000737	DOBKAI	10.5517/cc12lbvv	W.Clegg, L.Horsburgh	Newcastle	C15 H22 I1 N1 O2 S1
1000738	DOBKEM	10.5517/cc12lbvv	W.Clegg, L.Horsburgh	Newcastle	C17 H28 I1 N1 O3 Si1
1000739	DOBKIQ	10.5517/cc12lbxx	W.Clegg, L.Horsburgh	Newcastle	C12 H16 I1 N1 O3
1000740	DOBJUB	10.5517/cc12lbyy	W.Clegg	Newcastle	C15 H24 N2 O5
1000741	DOBKOW	10.5517/cc12lbzz	W.Clegg, M.R.J.Elsegood	Newcastle	C28 H44 O4 S2 Si2
1000742	DOBKUC	10.5517/cc12lc01	W.Clegg, M.R.J.Elsegood	Newcastle	C19 H27 N1 O6 S1
1000743	DOBLAJ	10.5517/cc12lc12	W.Clegg, M.R.J.Elsegood	Newcastle	C19 H25 Fe1 N1 O4
1000744	DOBLEN	10.5517/cc12lc23	W.Clegg, M.R.J.Elsegood	Newcastle	C8 H12 N2 O4
1000745	DOBLIR	10.5517/cc12lc34	W.Clegg, M.R.J.Elsegood	Newcastle	C30 H51 N1 O6,1.5(C1 H2 Cl2)
1000746	DOBLOX	10.5517/cc12lc45	W.Clegg, M.R.J.Elsegood	Newcastle	C11 H19 N1 O4
1000747	DOBLUD	10.5517/cc12lc56	W.Clegg, M.R.J.Elsegood	Newcastle	C23 H29 N1 O9
1000748	DOBJEL	10.5517/cc12lc67	W.Clegg, P.A.O'Neill	Newcastle	C29 H44 O4 S1 Si1
1000749	DOBJIP	10.5517/cc12lc78	W.Clegg, P.A.O'Neill	Newcastle	C27 H40 O4 S1 Si1
1308208	ZADVAB		A.J.Banister, N.Bricklebank, W.Clegg, M.R.J.Elsegood, C.I.Gregory, I.Lavender, J.M.Rawson, B.K.Tanner	UK	C6 F4 N3 S2
1410002	IVIWIV	10.5517/ccdc.csd.cc1jb6y0	W.Clegg, R.W.Harrington	Newcastle	C11 H13 N3 O2 S2
1411519	CUNHIE	10.5517/cc1jcswk	W.Clegg, D.R.Harbron	Newcastle	C40 H58 N4 Ni2 O9,0.5(C5 H5 N1)
1411537	GEDTAL01	10.5517/ccdc.csd.cc1n402w	W.Clegg, D.R.Harbron	Newcastle	(C12 H15 O6 Y1)n
1412957	CURVES	10.5517/cc1jf98j	W.Clegg	Newcastle	C16 H11 N1
1412968	DUFRAZ	10.5517/cc1jf9mw	W.Clegg, D.R.Harbron	Newcastle	C10 H18 Cd1 O6
1412969	DUFRED	10.5517/cc1jf9nx	W.Clegg, D.R.Harbron	Newcastle	C40 H44 Cd2 N4 O8
1412971	DUFRIH	10.5517/cc1jf9qz	W.Clegg, D.R.Harbron	Newcastle	C23 H27 N3 Ni1 O5
1412974	DUFRON	10.5517/cc1jf9t2	W.Clegg, D.R.Harbron	Newcastle	C18 H24 Co1 N2 O6
1413004	DUFSOO	10.5517/cc1jfbs2	W.Clegg, D.R.Harbron	Newcastle	C64 H60 N12 Na1 Ni4 O12 1+,C5 H5 N1,9(H2 O1),Cl1 1-
1413008	DUFSUU	10.5517/cc1jfbx6	W.Clegg, D.R.Harbron	Newcastle	C40 H50 N4 Ni2 O9
1415289	YAJGIA01	10.5517/cc1jhqh7	W.Clegg	Newcastle	(C40 H59 La3 O24)n
1417060	RUJJAJ	10.5517/cc1jkkm8	W.Clegg, A.McCamley	Newcastle	(C24 H38 O16 Y2)n,0.5(C4 H6 O2),H2 O1
1417061	RUJJEN	10.5517/cc1jkkn9	W.Clegg, A.McCamley	Newcastle	C12 H22 Cd1 N4 O4 S2
1417062	RUJJIR	10.5517/cc1jkkpb	W.Clegg, A.McCamley	Newcastle	C10 H18 N4 O4 Pb1 S2
1417063	RUJJOX	10.5517/cc1jkkqc	W.Clegg	Newcastle	C18 H18 N2 O4 Pb1,H2 O1

1417727	RUPSAY	10.5517/cc1jl84j	W.Clegg, M.R.J.Elsegood	Newcastle	(C35 H52 La2 O15)n,C5 H8 O2
1417729	RUQCIR	10.5517/cc1jl86l	W.Clegg, M.R.J.Elsegood, S.P.Thompson	Newcastle	C40 H36 Mn2 N4 O8,H2 O1
1417735	RUQCOX	10.5517/cc1jl8ds	W.Clegg, M.R.J.Elsegood	Newcastle	C16 H14 N2 O4 Pb1
1417736	ZAMJEC01	10.5517/cc1jl8ft	W.Clegg, M.R.J.Elsegood	Newcastle	(C12 H19 La1 O8)n
1417737	RUQDAK	10.5517/cc1jl8gv	W.Clegg, M.R.J.Elsegood	Newcastle	C18 H18 N2 O4 Zn1
1417837	RUQJUK	10.5517/cc1jlcp5	W.Clegg, S.P.Thompson	Newcastle	C8 H14 N4 O4 Pb1 S2
1422524	ZUTMOS	10.5517/cc1jr7wd	W.Clegg, P.A.O'Neil	Newcastle	(C12 H19 O8 Y1)n,C10 H8 N2
1422525	KIYSEQ01	10.5517/cc1jr7xf	W.Clegg, P.A.O'Neil	Newcastle	C18 H18 O6 Zn1
1422526	ZUTNAF	10.5517/cc1jr7yg	W.Clegg	Newcastle	C8 H14 Cd1 O6
1422527	ZUTNEJ	10.5517/cc1jr7zh	W.Clegg, M.R.J.Elsegood	Newcastle	(C12 H19 La1 O8)n,C10 H8 N2
1422528	ZUTNIN	10.5517/cc1jr80k	W.Clegg, M.R.J.Elsegood	Newcastle	C22 H24 Cd1 N2 O5,0.5(C10 H8 N2),H2 O1
1422529	ZZZNWQ02	10.5517/cc1jr81l	W.Clegg, M.R.J.Elsegood	Newcastle	C6 H8 O2
1422530	ZUTNUZ	10.5517/cc1jr82m	W.Clegg, M.R.J.Elsegood	Newcastle	C22 H24 N2 O5 Zn1
1422531	ZUTPAH	10.5517/cc1jr83n	W.Clegg	Newcastle	C48 H46 N4 O12 Y2
1422532	ZUTPEL	10.5517/cc1jr84p	W.Clegg, P.A.O'Neil	Newcastle	(C30 H36 N2 O8 Pb2)n,H2 O1
1422695	ZUTWIW	10.5517/cc1jrfd3	W.Clegg	Newcastle	(C240 H336 Cl1 O96 Pb24)n
1422764	ZUTWOC	10.5517/cc1jrhmd	W.Clegg, R.A.Coxall	Newcastle	(C240 H336 Br1 O96 Pb24)n
1443492	QILNAE	10.5517/ccdc.csd.cc1kg28c	W.Clegg, R.W.Harrington	Newcastle	C6 H15 F1 N1 O1 1+,C7 H7 O3 S1 1-
1443493	QILNEI	10.5517/ccdc.csd.cc1kg29d	W.Clegg, R.W.Harrington	Newcastle	C6 H15 F1 N1 O1 1+,Cl1 1-
1443730	HAWZEM01	10.5517/ccdc.csd.cc1kg9y8	W.Clegg, R.W.Harrington	Newcastle	(C12 H8 Cu1 N2 O4)n,C3 H7 N1 O1
1454007	BEQKUH	10.5517/ccdc.csd.cc1kt0gv	W.Clegg, R.W.Harrington	Newcastle	0.16(C38 H27 O1 P1),0.84(C38 H27 P1)
1458693	OJOMAD	10.5517/ccdc.csd.cc1kywm0	W.Clegg, R.W.Harrington	International	C16 H18 Mn1 N8 O4 S4,H2 O1
1458695	OJOMIL	10.5517/ccdc.csd.cc1kywp2	W.Clegg, R.W.Harrington	International	C20 H14 N4 O4
1458696	VUKPOI01	10.5517/ccdc.csd.cc1kywq3	W.Clegg, R.W.Harrington	International	C20 H18 N4 O4
1458697	HIQCIU01	10.5517/ccdc.csd.cc1kywr4	W.Clegg, R.W.Harrington	International	C38 H33 Cl1 Cu1 N4 O4 P1
1458702	OJOLEG	10.5517/ccdc.csd.cc1kywx9	W.Clegg, R.W.Harrington	International	(C26 H16 Cl2 Hg1 N4 O4 S2)n
1458707	OJOLIK	10.5517/ccdc.csd.cc1kyx2h	W.Clegg, R.W.Harrington	International	(C28 H22 Hg1 N4 O4 S2)n
1458708	SEHFUH01	10.5517/ccdc.csd.cc1kyx3j	W.Clegg, R.W.Harrington	International	C8 H32 Br2 N8 Ni2 2+,2(Br1 1-)
1458709	VAZHEK02	10.5517/ccdc.csd.cc1kyx4k	W.Clegg, R.W.Harrington	International	C20 H26 N4
1458710	IDOCEK01	10.5517/ccdc.csd.cc1kyx5l	W.Clegg, R.W.Harrington	International	C20 H18 Cl2 Co1 N4 O4
1458720	OJONEI	10.5517/ccdc.csd.cc1kyxhx	W.Clegg, R.W.Harrington	International	C31 H36 Co1 N4 O2 1+,Cl1 O4 1-
1458721	OJONOS	10.5517/ccdc.csd.cc1kyxjy	W.Clegg, R.W.Harrington	International	C40 H44 Co4 N16 O4
1458722	MIBFOT01	10.5517/ccdc.csd.cc1kyxkz	W.Clegg, R.W.Harrington	International	C18 H18 N4 O4
1458723	PAWHEC01	10.5517/ccdc.csd.cc1kyxl0	W.Clegg, R.W.Harrington	International	C18 H20 N2 O4
1458724	OJOPEK	10.5517/ccdc.csd.cc1kyxm1	W.Clegg, R.W.Harrington	International	C20 H24 N2 O4
1458926	SILJAZ01	10.5517/ccdc.csd.cc1kz44t	W.Clegg, R.W.Harrington	International	C38 H33 Br1 Cu1 N4 O4 P1
1458961	EQOBAN01	10.5517/ccdc.csd.cc1kz58z	W.Clegg, R.W.Harrington	International	C19 H22 N2 O4
1458967	OJONAE	10.5517/ccdc.csd.cc1kz5g5	W.Clegg, R.W.Harrington	International	C33 H34 Co1 N4 O2 1+,C2 H6 O1,Cl1 O4 1-
1458968	HINYUZ01	10.5517/ccdc.csd.cc1kz5h6	W.Clegg, R.W.Harrington	International	C32 H36 Co1 N4 O2 1+,C24 H20 B1 1-,C6 H7 N1
1458969	XIKZIB01	10.5517/ccdc.csd.cc1kz5j7	W.Clegg, R.W.Harrington	International	C38 H33 Cu1 I1 N4 O4 P1
1458970	TOHHED04	10.5517/ccdc.csd.cc1kz5k8	W.Clegg, R.W.Harrington	International	C14 H12 N2 O2
1458971	OJOQAH	10.5517/ccdc.csd.cc1kz5l9	W.Clegg, R.W.Harrington	International	(C48 H36 F6 Hg3 O12)n

1470745	OJEPIE	10.5517/ccdc.csd.cc1lcfds	W.Clegg	International	(C12 H22 Hg2 I2 S2)n
1470746	OJEPOK	10.5517/ccdc.csd.cc1lcfft	W.Clegg, K.A.Fraser	International	C28 H30 Cd2 I2 N4 O4 S2
1470747	OJEPUQ	10.5517/ccdc.csd.cc1lcfgv	W.Clegg, K.A.Fraser	International	C28 H30 Br2 Cd2 N4 O4 S2
1470749	OJEQAX	10.5517/ccdc.csd.cc1lcfjx	W.Clegg, K.A.Fraser	International	C12 H29 Br1 Hg1 N2 S2
1470750	OJEQEB	10.5517/ccdc.csd.cc1lcfky	W.Clegg, M.R.J.Elsegood	International	C26 H24 I2 P2 Pt1,C6 H6
1470751	IKUVUH	10.5517/ccdc.csd.cc1lcflz	W.Clegg	International	C24 H56 Br4 Hg4 N4 S4
1470752	IKUWAO	10.5517/ccdc.csd.cc1lcfm0	W.Clegg, L.Cucurull-Sanchez	International	C16 H22 N2 O6 Zn1 2+,2(N1 O3 1-)
1470753	ZNFORD09	10.5517/ccdc.csd.cc1lcfn1	W.Clegg, L.Cucurull-Sanchez	International	(C4 H12 O12 Zn2)n
1470754	IKUWIW	10.5517/ccdc.csd.cc1lcfp2	W.Clegg	International	C12 H28 N4 O1 S4 Zn1
1470755	IKUWOC	10.5517/ccdc.csd.cc1lcfq3	W.Clegg, R.A.Coxall	International	C78 H72 P6 Pt3 S2 2+,2(Cl1 O4 1-),5.5(C1 H1 Cl3)
1470756	ZNPICH07	10.5517/ccdc.csd.cc1lcfr4	W.Clegg, R.A.Coxall	International	C12 H12 N2 O6 Zn1,2(H2 O1)
1470758	IKUXAP	10.5517/ccdc.csd.cc1lcft6	W.Clegg, R.A.Coxall	International	C27 H33 N3 O6 Zn1 2+,2(F6 P1 1-)
1470759	KAKVIB01	10.5517/ccdc.csd.cc1lcfv7	W.Clegg, R.A.Coxall	International	C22 H20 N4 S2 Zn1
1470760	OJEQOL	10.5517/ccdc.csd.cc1lcfw8	W.Clegg, R.A.Coxall	International	C15 H30 N6 S4 Zn1
1470762	OJEQUR	10.5517/ccdc.csd.cc1lcfyb	W.Clegg, R.A.Coxall	International	(C12 H26 Cd1 N4 S4)n
1470763	OJERAY	10.5517/ccdc.csd.cc1lcfzc	W.Clegg, R.A.Coxall	International	C12 H28 N4 Ni1 S4 2+,2(Cl1 1-)
1470764	OJEREC	10.5517/ccdc.csd.cc1lcg0f	W.Clegg, R.A.Coxall	International	C24 H34 Cd1 N6 S4
1470765	EXONOU01	10.5517/ccdc.csd.cc1lcg1g	W.Clegg, R.A.Coxall	International	C18 H36 Ag2 N12 S6 2+,2(Cl1 O4 1-)
1470766	OJEROM	10.5517/ccdc.csd.cc1lcg2h	W.Clegg, R.A.Coxall	International	C36 H24 N6 Zn1 2+,C16 H20 N2 S2,2(Cl1 O4 1-),H2 O1
1470767	OJEVOQ	10.5517/ccdc.csd.cc1lcg3j	W.Clegg	International	C26 H24 As2 Cl2 Pt1,C1 H2 Cl2
1470768	OJEVUW	10.5517/ccdc.csd.cc1lcg4k	W.Clegg, N.R.Brooks	International	C57 H57 P4 Pt2 S2 1+,C1 H2 Cl2,Br1 1-
1474096	ABANEY01	10.5517/ccdc.csd.cc1lgxhg	W.Clegg, R.W.Harrington	International	C22 H15 N1 O3
1474097	ANEWEX	10.5517/ccdc.csd.cc1lgxjh	W.Clegg, R.W.Harrington	International	C20 H24 N2 O4
1474098	SILKOO01	10.5517/ccdc.csd.cc1lgxkj	W.Clegg, R.W.Harrington	International	C38 H33 Cu1 N7 O4 P1,C1 H1 Cl3
1474099	KIDBIJ01	10.5517/ccdc.csd.cc1lgxlk	W.Clegg, R.W.Harrington	International	C20 H24 N2 O4
1474102	TINREN03	10.5517/ccdc.csd.cc1lgxpn	W.Clegg, R.W.Harrington	International	C54 H45 Cl1 Cu1 P3,C2 H3 N1
1474103	TCTPAG02	10.5517/ccdc.csd.cc1lgxqp	W.Clegg, R.W.Harrington	International	C74 H60 Ag2 N2 P4 S2
1474104	CTPPCU05	10.5517/ccdc.csd.cc1lgxrq	W.Clegg, R.W.Harrington	International	C54 H45 Cl2 Cu2 P3
1474105	SIMLIK01	10.5517/ccdc.csd.cc1lgxsr	W.Clegg	International	C22 H30 N4
1474106	ANEXIC	10.5517/ccdc.csd.cc1lgxts	W.Clegg, R.W.Harrington	International	C18 H18 F2 N2
1474113	ANEXOI	10.5517/ccdc.csd.cc1lgy11	W.Clegg, R.W.Harrington	International	C19 H22 N2 O4
1474115	DAPCUN02	10.5517/ccdc.csd.cc1lgy33	W.Clegg, R.W.Harrington	International	C6 H20 Cu1 N6 O6
1474117	ANELIQ	10.5517/ccdc.csd.cc1lgy55	W.Clegg, R.W.Harrington	International	C19 H22 N2 O4
1474121	WICBIU03	10.5517/ccdc.csd.cc1lgy99	W.Clegg, R.W.Harrington	International	C18 H20 Cu1 N2 O5
1474123	QARNUT02	10.5517/ccdc.csd.cc1lgycc	W.Clegg, R.W.Harrington	International	C18 H20 Cu1 N2 O5
1474124	ANEMAJ	10.5517/ccdc.csd.cc1lgydd	W.Clegg, R.W.Harrington	International	C18 H18 Cu1 N2 O4,H2 O1
1474125	ANEMEN	10.5517/ccdc.csd.cc1lgyff	W.Clegg, R.W.Harrington	International	C17 H18 N2 O4
1474126	ANEMOX	10.5517/ccdc.csd.cc1lgygg	W.Clegg, R.W.Harrington	International	C19 H22 Cu1 N2 O5
1474128	ANEMIR	10.5517/ccdc.csd.cc1lgyjj	W.Clegg, R.W.Harrington	International	C19 H22 N2 O4
1485190	TECZIO	10.5517/ccdc.csd.cc1lvgc8	W.Clegg, R.W.Harrington	Newcastle	C30 H39 N15 Ni1 O9 S3 2+,2(N1 O3 1-),2(H2 O1)
1488882	UQUGAQ	10.5517/ccdc.csd.cc1lz9gb	W.Clegg, S.M.Hodgson	UK	C20 H32 Li1 N5
1488883	UQUGEU	10.5517/ccdc.csd.cc1lz9hc	W.Clegg, S.M.Hodgson	UK	C24 H56 Li2 N8 O2 P2 S2

1488884	UQUGIY	10.5517/ccdc.csd.cc1lz9jd	W.Clegg	UK	C19 H31 Li1 N4
1488885	UQUGOE	10.5517/ccdc.csd.cc1lz9kf	W.Clegg, S.M.Hodgson	UK	C22 H44 Li2 N8 O2 P2 S2
1488886	UQUGUK	10.5517/ccdc.csd.cc1lz9lg	W.Clegg	UK	C48 H54 Li2 N4 O4 Si2
1488889	UQUHAR	10.5517/ccdc.csd.cc1lz9pk	W.Clegg	UK	C16 H31 Li1 N4
1488893	UQUHEV	10.5517/ccdc.csd.cc1lz9tp	W.Clegg, S.T.Liddle	UK	C48 H92 Li4 N12 O12 P4
1488895	UQUHIZ	10.5517/ccdc.csd.cc1lz9wr	W.Clegg, S.T.Liddle	UK	C16 H36 Ba1 N10 O2 P2 S4 2-,2(C6 H18 N3 O1 P1),2(H4 N1 1+)
1488898	UQUHOF	10.5517/ccdc.csd.cc1lz9zv	W.Clegg, S.T.Liddle	UK	C6 H16 N1 1+,F6 P1 1-
1488899	UQUHUL	10.5517/ccdc.csd.cc1lzb0x	W.Clegg, S.T.Liddle	UK	C6 H18 N3 O1 P1,H4 N1 1+,C1 N1 S1 1-
1488900	UQUJAT	10.5517/ccdc.csd.cc1lzb1y	W.Clegg, S.T.Liddle	UK	C52 H68 Li6 N6 O10,2(C4 H8 O1)
1488901	UQUJEX	10.5517/ccdc.csd.cc1lzb2z	W.Clegg, M.R.J.Elsegood	UK	C32 H52 Ba1 N6 Si4
1488902	UQUJIB	10.5517/ccdc.csd.cc1lzb30	W.Clegg, S.T.Liddle	UK	(C7 H18 Li1 N4 O1 P1 S1)n
1488903	UQUJOH	10.5517/ccdc.csd.cc1lzb41	W.Clegg	UK	C60 H96 Li4 N4 O4
1500400	OTETOY	10.5517/ccdc.csd.cc1mc909	W.Clegg	UK	C8 H16 Fe2 O15,2(H2 O1)
1500402	OTETUE	10.5517/ccdc.csd.cc1mc92c	W.Clegg	UK	C16 H24 B2 Cu1 F8 N8
1500403	OTEVAM	10.5517/ccdc.csd.cc1mc93d	W.Clegg	UK	C16 H24 B2 Cu1 F8 N8
1500404	OTEVEQ	10.5517/ccdc.csd.cc1mc94f	W.Clegg	UK	C28 H34 Cu1 N8 O6 S2
1500405	RASSAH01	10.5517/ccdc.csd.cc1mc95g	W.Clegg	UK	C18 H24 Cu1 N10 S2
1500406	YEFBIV01	10.5517/ccdc.csd.cc1mc96h	W.Clegg	UK	C10 H12 Cu1 N6 S2
1500407	OTEVUG	10.5517/ccdc.csd.cc1mc97j	W.Clegg	UK	C16 H20 N2 O4 Pd1
1500408	OTEWAN	10.5517/ccdc.csd.cc1mc98k	W.Clegg	UK	Cl11 Cu10 Mo2 S8 5-,5(C8 H20 N1 1+),2(C3 H7 N1 O1)
1500409	DOMVEG02	10.5517/ccdc.csd.cc1mc99l	W.Clegg	UK	C24 H20 Fe2 S6 2-,2(C8 H20 N1 1+)
1500410	YIJVOD01	10.5517/ccdc.csd.cc1mc9bm	W.Clegg	UK	C60 H50 B1 P2 Rh1
1500411	OTEWOB	10.5517/ccdc.csd.cc1mc9cn	W.Clegg	UK	C24 H20 P1 1+,C18 H15 Pb1 S3 1-
1500412	OTEWUH	10.5517/ccdc.csd.cc1mc9dp	W.Clegg	UK	C24 H20 P1 1+,C18 H12 Mo1 S6 1-
1500414	OTEXAO	10.5517/ccdc.csd.cc1mc9gr	W.Clegg	UK	C84 H72 Cl1 N2 P4 Rh2 S2 2+,2(F6 P1 1-)
1500416	OTEXES	10.5517/ccdc.csd.cc1mc9jt	W.Clegg	UK	2(C24 H20 P1 1+),C16 H8 N4 O1 S4 W1 2-
1500418	OTEXIW	10.5517/ccdc.csd.cc1mc9lw	W.Clegg	UK	C16 H36 N1 1+,C16 H8 Mo1 N4 O1 S4 1-
1500527	ASOCIW	10.5517/ccdc.csd.cc1mcf3j	W.Clegg	UK	C7 H5 N2 S2 1+,F3 O2 S1 1-
1500528	ASOCOC	10.5517/ccdc.csd.cc1mcf4k	W.Clegg	UK	C10 H14 O4 Zn1
1500532	WIMNAH01	10.5517/ccdc.csd.cc1mcf8p	W.Clegg	UK	C7 H4 Cl1 N2 S2
1500533	LIXGAB02	10.5517/ccdc.csd.cc1mcf9q	W.Clegg, M.R.J.Elsegood	UK	C8 H4 F3 N2 S2
1500534	ASODET	10.5517/ccdc.csd.cc1mcfbr	W.Clegg, M.R.J.Elsegood	UK	C7 H4 Br1 N2 S2
1500535	ASODIX	10.5517/ccdc.csd.cc1mcfcs	W.Clegg, M.R.J.Elsegood	UK	C8 F4 N4 S4
1500536	ASODOD	10.5517/ccdc.csd.cc1mcfdt	W.Clegg	UK	C7 H4 Cl1 N2 S2 1+,F1 O3 S1 1-
1500537	ASODUJ	10.5517/ccdc.csd.cc1mcffv	W.Clegg, M.R.J.Elsegood	UK	C6 H4 N3 S2
1500538	ASOFAR	10.5517/ccdc.csd.cc1mcfgw	W.Clegg, M.R.J.Elsegood	UK	C7 H4 N3 O2 S2
1500540	ASOFEV	10.5517/ccdc.csd.cc1mcfjy	W.Clegg, M.R.J.Elsegood	UK	C8 F4 N3 S2
1501840	ETIKUP	10.5517/ccdc.csd.cc1mdsg8	W.Clegg	International	C39 H10 B2 F20 O2,1.5(C7 H8)
1501841	WISRUL02	10.5517/ccdc.csd.cc1mdsh9	W.Clegg	International	C24 B2 F18,2(C7 H8)
1501846	ETIMAX	10.5517/ccdc.csd.cc1mdsng	W.Clegg	International	C126 H72 B6 F75 K3 O18
1501854	ETIMIF	10.5517/ccdc.csd.cc1mdsxq	W.Clegg	International	C64 H8 B4 F48 O2 2-,2(C19 H15 1+),C1 H2 Cl2
1501855	ETIMOL	10.5517/ccdc.csd.cc1mdsyr	W.Clegg, R.W.Harrington	International	C23 H31 Al1 N2

1501861	ETIMUR	10.5517/ccdc.csd.cc1mdt4z	W.Clegg, R.W.Harrington	International	C35 H55 N2 1+,C1 F3 O3 S1 1-,2(C1 H2 Cl2)
1501876	ETINAY	10.5517/ccdc.csd.cc1mdtmg	W.Clegg, R.W.Harrington	International	C42 H60 Cl2 N2 O2 P1 Ru1 1+,C24 H20 B1 1-
1501881	ETINEC	10.5517/ccdc.csd.cc1mdtsm	W.Clegg, R.W.Harrington	International	C40 H2 B2 F24 O2,C1 H1 Cl3,0.5(H2 O1)
1505580	AVERAW	10.5517/ccdc.csd.cc1mjp3y	W.Clegg	UK	C8 H15 B10 F1
1505581	UHOMIO01	10.5517/ccdc.csd.cc1mjp4z	W.Clegg	UK	C14 H19 B10 F1
1505583	UHOMOU01	10.5517/ccdc.csd.cc1mjp61	W.Clegg	UK	C15 H22 B10 O1
1505585	DEQNIW01	10.5517/ccdc.csd.cc1mjp83	W.Clegg	UK	C9 H27 B1 Li1 N3
1505592	AVERUQ	10.5517/ccdc.csd.cc1mjphb	W.Clegg	UK	C14 H12 Br3 N1 Sn1
1505593	AVESAX	10.5517/ccdc.csd.cc1mjpjc	W.Clegg	UK	2(C14 H14 N1 1+),Br6 Sn1 2-
1505594	ICAGIC01	10.5517/ccdc.csd.cc1mjpkd	W.Clegg	UK	(C20 H24 Na2 O24 Zn1)n,2(H2 O1)
1505595	RUJKEN01	10.5517/ccdc.csd.cc1mjplf	W.Clegg	UK	C6 H16 N4 O4,4(H2 O1)
1505599	NADQAL03	10.5517/ccdc.csd.cc1mjpqk	W.Clegg	UK	C6 H12 O6
1505606	AVEWAB	10.5517/ccdc.csd.cc1mjpys	W.Clegg	UK	C24 H18 F4 N3 O2 P3
1505607	AVEWEF	10.5517/ccdc.csd.cc1mjpzt	W.Clegg	UK	C12 H8 F6 N3 P3
1505608	AVEWIJ	10.5517/ccdc.csd.cc1mjq0w	W.Clegg	UK	C18 H15 F3 N3 O1 P3
1505609	AVEWOP	10.5517/ccdc.csd.cc1mjq1x	W.Clegg	UK	C24 H20 F2 N3 P3
1505610	AVEWUV	10.5517/ccdc.csd.cc1mjq2y	W.Clegg	UK	C26 H28 B10 O2
1505612	KAHSEU01	10.5517/ccdc.csd.cc1mjq40	W.Clegg	UK	C12 H18 B10 N2
1505613	AVEXEG	10.5517/ccdc.csd.cc1mjq51	W.Clegg, M.R.J.Elsegood	UK	C18 H13 F5 N3 O1 P3
1505614	AVEXIK	10.5517/ccdc.csd.cc1mjq62	W.Clegg, M.R.J.Elsegood	UK	C40 H64 B20 Mo1 N2 O2
1505615	AVEXOQ	10.5517/ccdc.csd.cc1mjq73	W.Clegg, M.R.J.Elsegood	UK	C6 H26 B20 Cl4 Mo1 N2 2-,2(C6 H16 N1 1+)
1505616	AVEXUW	10.5517/ccdc.csd.cc1mjq84	W.Clegg, A.J.Edwards	UK	C36 H50 B20 O3 Zr2,2(C4 H8 O1)
1505617	AVEYAD	10.5517/ccdc.csd.cc1mjq95	W.Clegg	UK	C24 H16 Cl2 F4 N3 P3
1510672	CACKAT01	10.5517/ccdc.csd.cc1mpzcn	W.Clegg, E.Hevia, L.Davin	Strathclyde	C29 H42 N2
1510678	OXOMEV	10.5517/ccdc.csd.cc1mpzkv	W.Clegg, E.Hevia, L.Davin	Strathclyde	C66 H100 Mg2 N4 O4,C4 H8 O1
1519722	EZUZEG	10.5517/ccdc.csd.cc1n0d9c	W.Clegg, L.Horsburgh	Newcastle	C12 H15 N1 O2
1519723	EZUZIK	10.5517/ccdc.csd.cc1n0dbd	W.Clegg, L.Horsburgh	Newcastle	C19 H22 N2 O1
1519724	EZUZOQ	10.5517/ccdc.csd.cc1n0dcf	W.Clegg	Newcastle	C18 H13 N3 O6
1519725	EZUZUW	10.5517/ccdc.csd.cc1n0ddg	W.Clegg, R.W.Harrington	Newcastle	C18 H18 O6 S1
1519726	EBABAN	10.5517/ccdc.csd.cc1n0dfh	W.Clegg, R.W.Harrington	Newcastle	C14 H12 Br1 N3 O3
1519727	EBABER	10.5517/ccdc.csd.cc1n0dgj	W.Clegg, R.W.Harrington	Newcastle	C16 H10 F5 N3 O3
1520865	MIMKOJ01	10.5517/ccdc.csd.cc1n1l5g	W.Clegg, R.W.Harrington	Newcastle	C17 H15 N1 O3
1520866	LISZAQ01	10.5517/ccdc.csd.cc1n1l6h	W.Clegg, R.W.Harrington	Newcastle	C36 H54 N2 O2
1520869	MICHOW01	10.5517/ccdc.csd.cc1n1l9l	W.Clegg, R.W.Harrington	Newcastle	C64 H92 N4 O6 Ti2,2(C1 H2 Cl2)
1520870	YAGCOB	10.5517/ccdc.csd.cc1n1lbm	W.Clegg, R.W.Harrington	Newcastle	C14 H12 I1 N1 O1
1520871	YAGCUH	10.5517/ccdc.csd.cc1n1lcn	W.Clegg, R.W.Harrington	Newcastle	C20 H22 N2 O4
1523185	EVIHUM04	10.5517/ccdc.csd.cc1n400t	W.Clegg, R.W.Harrington	Newcastle	C15 H22 O2
1523186	UBOZIX	10.5517/ccdc.csd.cc1n401v	W.Clegg, R.W.Harrington	Newcastle	C10 H10 Cl1 N1 O1 S1
1523187	FEBZET01	10.5517/ccdc.csd.cc1n402w	W.Clegg, R.W.Harrington	Newcastle	C15 H13 N1 O2
1523188	UBOZUJ	10.5517/ccdc.csd.cc1n403x	W.Clegg, R.W.Harrington	Newcastle	C10 H3 F5 N2
1523189	UBUBAX	10.5517/ccdc.csd.cc1n404y	W.Clegg, R.W.Harrington	Newcastle	C25 H19 N3 O3
1523190	UBUBEB	10.5517/ccdc.csd.cc1n405z	W.Clegg, R.W.Harrington	Newcastle	C25 H19 Br1 N2 O1

1523191	UBUBIF	10.5517/ccdc.csd.cc1n4060	W.Clegg, R.W.Harrington	Newcastle	2(C12 H8 Ag1 N4 1+)n,F6 Si1 2-
1523192	UBUBOL	10.5517/ccdc.csd.cc1n4071	W.Clegg, R.W.Harrington	Newcastle	C26 H16 N10 Ni1 S2
1523193	UBUBUR	10.5517/ccdc.csd.cc1n4082	W.Clegg, R.W.Harrington	Newcastle	C28 H16 Cu2 N12 S4,0.5(C6 H4 N2)
1530209	JAMQUM	10.5517/ccdc.csd.cc1nc9lx	W.Clegg, R.W.Harrington	Newcastle	(C10 H10 K1 Mo12 N4 O40 P1 2-)n,n(C18 H17 Ag2 N7 2+),n(C2 H3 N1)
1530210	JAMRAT	10.5517/ccdc.csd.cc1nc9my	W.Clegg, R.W.Harrington	Newcastle	C7 H3 N3
1530211	JAMREX	10.5517/ccdc.csd.cc1nc9nz	W.Clegg, R.W.Harrington	Newcastle	C38 H32 N2 Ni1 O4 P2 S2,0.5(C2 H3 N1)
1530212	JAMRIB	10.5517/ccdc.csd.cc1nc9p0	W.Clegg, R.W.Harrington	Newcastle	C7 H3 N3
1530213	JAMROH	10.5517/ccdc.csd.cc1nc9q1	W.Clegg, R.W.Harrington	Newcastle	3(C44 H54 Ag2 N2 O12 2+),2(Mo12 O40 P1 3-)
1530218	CUPICH19	10.5517/ccdc.csd.cc1nc9w6	W.Clegg, R.W.Harrington	Newcastle	(C12 H8 Cu1 N2 O4)n,2(H2 O1)
1530219	JAMSAU	10.5517/ccdc.csd.cc1nc9x7	W.Clegg, R.W.Harrington	Newcastle	C24 H20 Cu4 Mo1 N8 S4 2-,4(C3 H7 N1 O1),2(H4 N1 1+)
1532500	ZAKPIN	10.5517/ccdc.csd.cc1nfph8	W.Clegg, R.W.Harrington	Newcastle	C24 H20 Cu4 Mo1 N8 S4 2-,2(H4 N1 1+)
1532501	CYPYCU12	10.5517/ccdc.csd.cc1nfpj9	W.Clegg, R.W.Harrington	Newcastle	(C7 H4 Cu1 N3)n
1532502	ZAKQAG	10.5517/ccdc.csd.cc1nfpkb	W.Clegg, R.W.Harrington	Newcastle	C12 H24 Ag6 Mo6 O6 S19 2-,1.75(C20 H24 O6),2(C2 H3 N1),2(Na1 1+)
1532503	ZAKQIO	10.5517/ccdc.csd.cc1nfplc	W.Clegg, R.W.Harrington	Newcastle	C6 H3 Cl1 N2
1532504	ZAKQOU	10.5517/ccdc.csd.cc1nfpmd	W.Clegg, R.W.Harrington	Newcastle	C4 H8 Mo2 O2 S6 2-,2(H4 N1 1+),2(H2 O1)
1532505	ZAKQUA	10.5517/ccdc.csd.cc1nfpnf	W.Clegg, R.W.Harrington	Newcastle	Mo12 O40 P1 3-,1.5(C10 H24 Mn1 O7 2+)
1532506	ZAKRAH	10.5517/ccdc.csd.cc1nfppg	W.Clegg, R.W.Harrington	Newcastle	C12 H24 Ag6 Mo6 O6 S19 2-,2(C8 H20 N1 1+),1.75(C3 H6 O1)
1570987	MAZQOW	10.5517/ccdc.csd.cc1pqr05	W.Clegg	Newcastle	C38 H41 P3
1571003	MAZREN	10.5517/ccdc.csd.cc1pqrjp	W.Clegg	Newcastle	C37 H46 P2
1571004	MAZRIR	10.5517/ccdc.csd.cc1pqrkq	W.Clegg, K.A.Fraser	Newcastle	C37 H46 Cl2 P2 Pt1 Se1,C6 D6
1571005	CEMSAP03	10.5517/ccdc.csd.cc1pqrlr	W.Clegg, K.A.Fraser	Newcastle	C29 H22 O4 P2 W1
1571006	MAZRUD	10.5517/ccdc.csd.cc1pqrms	W.Clegg, K.A.Fraser	Newcastle	C34 H37 Mo1 O6 P3
1571008	MAZSAK	10.5517/ccdc.csd.cc1pqrpv	W.Clegg	Newcastle	C37 H46 P2 S2,C6 H6
1571009	UYIPAV01	10.5517/ccdc.csd.cc1pqrqw	W.Clegg	Newcastle	C37 H41 N2 P2 Pd1 1+,F6 P1 1-
1571010	MAZSIS	10.5517/ccdc.csd.cc1pqrrx	W.Clegg	Newcastle	C47 H54 Cl2 N2 P2 Pd2,C1 H4 O1
1571011	MAZSOY	10.5517/ccdc.csd.cc1pqrsy	W.Clegg	Newcastle	C47 H60 N1 P2 Pd1 S1 1+,F6 P1 1-
1571012	MAZSUE	10.5517/ccdc.csd.cc1pqrtz	W.Clegg, M.R.J.Elsegood	Newcastle	C58 H51 Cl1 O2 P5 Pt1 1+,F6 P1 1-,4(C1 H4 O1)
1571013	MAZTAL	10.5517/ccdc.csd.cc1pqrv0	W.Clegg	Newcastle	C36 H38 N1 P2 Pd1 1+,F6 P1 1-
1571014	DOXFAX01	10.5517/ccdc.csd.cc1pqrw1	W.Clegg, L.Horsburgh	Newcastle	(C20 H26 Li1 O2 P1)n
1571015	MAZTIT	10.5517/ccdc.csd.cc1pqrx2	W.Clegg, A.J.Scott	Newcastle	C30 H42 Mo1 O4 P2
1571016	MAZTOZ	10.5517/ccdc.csd.cc1pqry3	W.Clegg, A.J.Scott	Newcastle	C47 H60 N1 P2 Pd1 1+,F6 P1 1-
1571017	MAZTUF	10.5517/ccdc.csd.cc1pqrz4	W.Clegg, A.J.Scott	Newcastle	C37 H40 N1 P2 Pd1 1+,F6 P1 1-,C1 H2 Cl2
1571018	MAZVAN	10.5517/ccdc.csd.cc1pqs06	W.Clegg, A.J.Scott	Newcastle	C22 H32 N1 O4 P1 W1
1571019	MAZVER	10.5517/ccdc.csd.cc1pqs17	W.Clegg, A.J.Scott	Newcastle	C30 H42 O4 P2 W1
1571020	MAZVIV	10.5517/ccdc.csd.cc1pqs28	W.Clegg, A.J.Scott	Newcastle	C21 H33 Cl1 P1 Rh1
1571021	ZUHKAO03	10.5517/ccdc.csd.cc1pqs39	W.Clegg, A.J.Scott	Newcastle	C25 H22 P2 S2
1571022	IXUCEK01	10.5517/ccdc.csd.cc1pqs4b	W.Clegg, A.J.Scott	Newcastle	C28 H26 Cl2 P2 Pd1
1571023	MAZWAO	10.5517/ccdc.csd.cc1pqs5c	W.Clegg, A.J.Edwards	Newcastle	C28 H44 P2
1571024	ZIVQOM01	10.5517/ccdc.csd.cc1pqs6d	W.Clegg, M.R.J.Elsegood	Newcastle	C24 H27 P1 Se1
1571025	MAZWIW	10.5517/ccdc.csd.cc1pqs7f	W.Clegg, M.R.J.Elsegood	Newcastle	C28 H43 Cl1 P2 Pt1,0.5(C1 H2 Cl2)
1571026	MAZWOC	10.5517/ccdc.csd.cc1pqs8g	W.Clegg, M.R.J.Elsegood	Newcastle	C26 H40 P2
1571078	MAZZAR	10.5517/ccdc.csd.cc1pqty5	W.Clegg, M.R.J.Elsegood	Newcastle	C27 H42 P2

1571079	MAZZEV	10.5517/ccdc.csd.cc1pqtz6	W.Clegg, L.Horsburgh	Newcastle	C26 H42 Cl2 P2 Pt1
1571080	MAZZIZ	10.5517/ccdc.csd.cc1pqv08	W.Clegg, L.Horsburgh	Newcastle	C75 H66 Ag3 Cl2 P6 1+,Cl1 1-
1571081	MAZZOF	10.5517/ccdc.csd.cc1pqv19	W.Clegg, M.R.J.Elsegood	Newcastle	C55 H58 Cl2 N2 P2 Pd2,C1 H4 O1
1571082	MAZZUL	10.5517/ccdc.csd.cc1pqv2b	W.Clegg, M.R.J.Elsegood	Newcastle	C46 H50 Cl2 N2 P2 Pd2
1571083	MEBBAZ	10.5517/ccdc.csd.cc1pqv3c	W.Clegg, M.R.J.Elsegood	Newcastle	C19 H24 O6 S2
1571084	MEBBED	10.5517/ccdc.csd.cc1pqv4d	W.Clegg, M.R.J.Elsegood	Newcastle	C40 H38 N1 P2 Pd1 1+,F6 P1 1-,C1 H4 O1
1571085	MEBBIH	10.5517/ccdc.csd.cc1pqv5f	W.Clegg, M.R.J.Elsegood	Newcastle	C46 H52 Cl2 N2 P2 Pd2
1571086	MEBBON	10.5517/ccdc.csd.cc1pqv6g	W.Clegg, M.R.J.Elsegood	Newcastle	C48 H54 Cl2 N2 P2 Pd2,C1 H2 Cl2,C1 H4 O1
1571087	IXUCEK02	10.5517/ccdc.csd.cc1pqv7h	W.Clegg, M.R.J.Elsegood	Newcastle	C28 H26 Cl2 P2 Pd1
1571088	MAZZUL01	10.5517/ccdc.csd.cc1pqv8j	W.Clegg, M.R.J.Elsegood	Newcastle	C46 H50 Cl2 N2 P2 Pd2
1571089	MEBCEE	10.5517/ccdc.csd.cc1pqv9k	W.Clegg, P.A.Champkin	Newcastle	C54 H56 Cl2 N2 P2 Pd2
1571090	MEBCII	10.5517/ccdc.csd.cc1pqvbl	W.Clegg, M.R.J.Elsegood	Newcastle	C38 H40 N1 P2 Pd1 1+,F6 P1 1-,C1 H4 O1
1571091	MEBCOO	10.5517/ccdc.csd.cc1pqvcm	W.Clegg	Newcastle	C18 H23 O6 P1
1571122	MEBHOT	10.5517/ccdc.csd.cc1pqwcn	W.Clegg, A.J.Edwards	Newcastle	C54 H42 P4 S4,C1 H2 Cl2
1575154	YEHZUJ	10.5517/ccdc.csd.cc1pw2f2	W.Clegg, A.J.Scott	UK	C24 H10 F8 O2
1575155	YEJBAT	10.5517/ccdc.csd.cc1pw2g3	W.Clegg, A.J.Scott	UK	C24 H6 F12 O2
1575159	YEJBEX	10.5517/ccdc.csd.cc1pw2l7	W.Clegg, A.J.Scott	UK	C24 H18 S2
1575160	YEJBIB	10.5517/ccdc.csd.cc1pw2m8	W.Clegg, A.J.Scott	UK	C24 H8 F4 N2
1575161	QESDOI02	10.5517/ccdc.csd.cc1pw2n9	W.Clegg, A.J.Scott	UK	C18 H14 O2
1575162	YEJBUN	10.5517/ccdc.csd.cc1pw2pb	W.Clegg, A.J.Scott	UK	C16 H8 F6
1575163	YEJCAU	10.5517/ccdc.csd.cc1pw2qc	W.Clegg, A.J.Scott	UK	C22 H10 F4
1575175	CATCOL17	10.5517/ccdc.csd.cc1pw33s	W.Clegg, A.J.Scott	UK	C6 H6 O2
1575176	RAWMIL01	10.5517/ccdc.csd.cc1pw34t	W.Clegg, A.J.Scott	UK	C42 H30 B4 O10
1575177	YEJCOI	10.5517/ccdc.csd.cc1pw35v	W.Clegg, A.J.Scott	UK	C24 H19 B2 N1 O6
1575178	YEJJAB	10.5517/ccdc.csd.cc1pw36w	W.Clegg, A.J.Scott	UK	C16 H11 N1 O1
1575179	YEJJEF	10.5517/ccdc.csd.cc1pw37x	W.Clegg, A.J.Scott	UK	C15 H11 N1 O3
1575180	YEJJIJ	10.5517/ccdc.csd.cc1pw38y	W.Clegg, A.J.Scott	UK	C24 H8 F10
1575181	VEXNER01	10.5517/ccdc.csd.cc1pw39z	W.Clegg, A.J.Scott	UK	C18 H12
1575182	YEJJUV	10.5517/ccdc.csd.cc1pw3b0	W.Clegg, A.J.Scott	UK	C16 H8 N2
1575183	YEJKAC	10.5517/ccdc.csd.cc1pw3c1	W.Clegg, A.J.Scott	UK	C24 H14 F4 S2
1575184	YEJKEG	10.5517/ccdc.csd.cc1pw3d2	W.Clegg, A.J.Scott	UK	C15 H11 N1 O2 S1
1575185	YEJKIK	10.5517/ccdc.csd.cc1pw3f3	W.Clegg, A.J.Scott	UK	C22 H12 N2 O4
1575186	YEJKOQ	10.5517/ccdc.csd.cc1pw3g4	W.Clegg, A.J.Scott	UK	C4 H14 B2 Cl4 N2
1575187	ZIFSOW01	10.5517/ccdc.csd.cc1pw3h5	W.Clegg, A.J.Scott	UK	C20 H16 O2
1575188	YEJTOZ	10.5517/ccdc.csd.cc1pw3j6	W.Clegg, A.J.Scott	UK	C15 H9 I1 O1
1575189	YEJTUF	10.5517/ccdc.csd.cc1pw3k7	W.Clegg, A.J.Scott	UK	C22 H4 F10
1575196	YEJVAN	10.5517/ccdc.csd.cc1pw3sg	W.Clegg, A.J.Scott	UK	C28 H36 B1 O4 Rh1
1575197	IBENAE02	10.5517/ccdc.csd.cc1pw3th	W.Clegg, A.J.Scott	UK	C11 H11 B1 O4
1575198	IBENAE01	10.5517/ccdc.csd.cc1pw3vj	W.Clegg, A.J.Scott	UK	C11 H11 B1 O4
1575199	YEJVOB	10.5517/ccdc.csd.cc1pw3wk	W.Clegg, A.J.Scott	UK	C40 H36 B1 O4 P2 Rh1
1575200	YEJVUH	10.5517/ccdc.csd.cc1pw3xl	W.Clegg, A.J.Scott	UK	C28 H40 B1 O4 1-,C12 H36 O2 P4 Rh1 1+
1575201	COSXEO01	10.5517/ccdc.csd.cc1pw3ym	W.Clegg, A.J.Scott	UK	C16 H14

1575202	CAPDIJ01	10.5517/ccdc.csd.cc1pw3zn	W.Clegg, A.J.Scott	UK	C18 H14
1575203	FEFPOW01	10.5517/ccdc.csd.cc1pw40q	W.Clegg, A.J.Scott	UK	C24 H18
1575204	YEJWOC	10.5517/ccdc.csd.cc1pw41r	W.Clegg, A.J.Scott	UK	C24 H12 N2
1575205	YEJWUI	10.5517/ccdc.csd.cc1pw42s	W.Clegg, A.J.Scott	UK	C30 H39 B1 O4 P1 Rh1,1.5(C7 H8)
1575206	VAQYUJ01	10.5517/ccdc.csd.cc1pw43t	W.Clegg, A.J.Scott	UK	C15 H4 F5 N1
1575275	QESNIM01	10.5517/ccdc.csd.cc1pw6b3	W.Clegg, A.J.Scott	UK	C16 H11 N1 S1
1575548	HEDWOF	10.5517/ccdc.csd.cc1pwh46	W.Clegg	UK	C20 H20 B1 O4 Rh1
1575560	HEDWUL	10.5517/ccdc.csd.cc1pwhjl	W.Clegg	UK	C31 H33 O2 P2 Rh1
1575561	HEDXAS	10.5517/ccdc.csd.cc1pwhkm	W.Clegg	UK	C46 H36 B1 Fe1 O4 P2 Rh1,1.5(C4 H8 O1)
1575562	HEDXEW	10.5517/ccdc.csd.cc1pwhln	W.Clegg, A.J.Scott	UK	C16 H10
1575563	HEDXIA	10.5517/ccdc.csd.cc1pwhmp	W.Clegg, A.J.Scott	UK	C38 H32 B1 O4 P2 Rh1,C11 H11 B1 O4
1575564	YEJJEF01	10.5517/ccdc.csd.cc1pwhnq	W.Clegg, A.J.Scott	UK	C15 H11 N1 O3
1575567	HEFDAA	10.5517/ccdc.csd.cc1pwhrt	W.Clegg	UK	C12 H11 N1 O1
1575568	HEFDII	10.5517/ccdc.csd.cc1pwhsv	W.Clegg, L.Horsburgh	UK	C10 H6 Cl1 N1 O2
1575570	HEFDOO	10.5517/ccdc.csd.cc1pwhvx	W.Clegg	UK	C20 H16 N2 O2
1575571	HEFDUU	10.5517/ccdc.csd.cc1pwhwy	W.Clegg	UK	C32 H16 N2
1575572	HEFFAC	10.5517/ccdc.csd.cc1pwhxz	W.Clegg	UK	C24 H12 F6
1575576	KUQSAS01	10.5517/ccdc.csd.cc1pwj14	W.Clegg, L.Horsburgh	UK	C14 H9 I1
1575577	HEFFIK	10.5517/ccdc.csd.cc1pwj25	W.Clegg	UK	C26 H18 F4 N2 O2
1575578	HEFFOQ	10.5517/ccdc.csd.cc1pwj36	W.Clegg	UK	C19 H13 N1 O3
1575579	GEQXUV01	10.5517/ccdc.csd.cc1pwj47	W.Clegg, L.Horsburgh	UK	C18 H15 B1 Br3 P1
1575580	NASKUN01	10.5517/ccdc.csd.cc1pwj58	W.Clegg, L.Horsburgh	UK	C12 H8 B2 O5
1575581	HEFLOW	10.5517/ccdc.csd.cc1pwj69	W.Clegg, L.Horsburgh	UK	C37 H30 B1 O4 P2 Rh1,C4 H8 O1
1575583	YEJVAN01	10.5517/ccdc.csd.cc1pwj8c	W.Clegg, L.Horsburgh	UK	C28 H36 B1 O4 Rh1
1575584	HEFMAJ	10.5517/ccdc.csd.cc1pwj9d	W.Clegg, L.Horsburgh	UK	C32 H33 O2 P2 Rh1
1833977	FEXLUS	10.5517/ccdc.csd.cc1zkdkh	W.Clegg, A.J.Scott	UK	C38 H44 P2 Rh1 1+,C4 H8 O1,B1 F4 1-
1836888	KEWSUD	10.5517/ccdc.csd.cc1znfgj	W.Clegg, R.W.Harrington	Newcastle	C18 H36 Mn1 O9 S12 W4 2-,2(C8 H20 N1 1+)
1836889	KEWTAK	10.5517/ccdc.csd.cc1znfhk	W.Clegg, R.W.Harrington	Newcastle	2(C12 H23 Cl1 Mn1 N1 O5 1+),Cl4 Fe4 S4 2-
1836895	KEWTEO	10.5517/ccdc.csd.cc1znfpr	W.Clegg, R.W.Harrington	Newcastle	C12 H24 Ag6 Mo6 O6 S19 2-,2(C9 H16 N1 1+),0.75(C3 H6 O1),H2 O1
1836897	KEWTIS	10.5517/ccdc.csd.cc1znfrt	W.Clegg, R.W.Harrington	Newcastle	C12 H24 Ag6 Mo6 O6 S19 2-,2(C8 H20 N1 1+),1.68(C2 H3 N1)
1836899	KEWTOY	10.5517/ccdc.csd.cc1znftw	W.Clegg, R.W.Harrington	Newcastle	C32 H32 Mn1 Mo2 N4 O2 S6,2(C1 H4 O1)
1836901	YEVHAI13	10.5517/ccdc.csd.cc1znfwy	W.Clegg, R.W.Harrington	Newcastle	(C10 H8 Mn1 N8)n
1836903	KEWVOA	10.5517/ccdc.csd.cc1znfy0	W.Clegg, R.W.Harrington	Newcastle	C8 H14 N2 2+,C4 H8 Mo2 O2 S6 2-,H2 O1
1836904	KEWVUG	10.5517/ccdc.csd.cc1znfz1	W.Clegg, R.W.Harrington	Newcastle	(C28 H28 Mn1 N6 S2)n,n(C1 H4 O1)
1836905	KEWWAN	10.5517/ccdc.csd.cc1zng03	W.Clegg, R.W.Harrington	Newcastle	2(C13 H15 N2 1+),C4 H8 Mo2 O2 S6 2-
1836908	KEWWER	10.5517/ccdc.csd.cc1zng36	W.Clegg, R.W.Harrington	Newcastle	Mo12 O40 P1 3-,C12 H8 Ag1 N4 1+,2(C6 H9 Ag1 N3 1+)
1836909	KEWWIV	10.5517/ccdc.csd.cc1zng47	W.Clegg, R.W.Harrington	Newcastle	C5 H10 N3 S1 1+,H2 O1,Cl1 1-
1836923	KEWWOB	10.5517/ccdc.csd.cc1znglp	W.Clegg, R.W.Harrington	Newcastle	C21 H22 N5 1+,N1 O3 1-
1836924	KEWWUH	10.5517/ccdc.csd.cc1zngmq	W.Clegg, R.W.Harrington	Newcastle	C4 H8 Mo2 O2 S6 2-,2(C10 H9 N2 1+),C10 H8 N2
1836926	KEWXAO	10.5517/ccdc.csd.cc1zngps	W.Clegg, R.W.Harrington	Newcastle	C14 H26 N6 Ni1 2+,0.5(C1 H4 O1),H2 O1,2(Cl1 1-)
1836927	HOHLAR01	10.5517/ccdc.csd.cc1zngqt	W.Clegg, R.W.Harrington	Newcastle	C10 H18 N2 O2
1836968	KEWXIW	10.5517/ccdc.csd.cc1znj16	W.Clegg, R.W.Harrington	Newcastle	C20 H18 Cu1 N4 O6,C1 H4 O1,H2 O1

1836969	KEWXOC	10.5517/ccdc.csd.cc1znj27	W.Clegg, R.W.Harrington	Newcastle	C14 H16 N2 S2
1836970	BUGNOG01	10.5517/ccdc.csd.cc1znj38	W.Clegg, R.W.Harrington	Newcastle	C34 H33 Br1 Ni1 P3 1+,C24 H20 B1 1-
1845393	ZAHJIB02	10.5517/ccdc.csd.cc1zy8t0	W.Clegg, G.S.Nichol	Newcastle	C3 H3 N3 O3,2(H2 O1)
1845394	AMYTAL12	10.5517/ccdc.csd.cc1zy8v1	W.Clegg, G.S.Nichol	Newcastle	C11 H18 N2 O3
1845395	CHPYRD01	10.5517/ccdc.csd.cc1zy8w2	W.Clegg, G.S.Nichol	Newcastle	C5 H4 Cl1 N1 O1
1845396	FAHDOI01	10.5517/ccdc.csd.cc1zy8x3	W.Clegg, G.S.Nichol	Newcastle	C5 H3 N2 O4 1-,K1 1+
1845397	DMANAP11	10.5517/ccdc.csd.cc1zy8y4	W.Clegg, G.S.Nichol	Newcastle	C14 H18 N2
1845403	OROTAC02	10.5517/ccdc.csd.cc1zy94c	W.Clegg, G.S.Nichol	Newcastle	C5 H4 N2 O4,H2 O1
1845404	SIMZOD02	10.5517/ccdc.csd.cc1zy95d	W.Clegg, G.S.Nichol	Newcastle	(C5 H5 Li1 N2 O5)n
1845408	LAVYOX02	10.5517/ccdc.csd.cc1zy99j	W.Clegg, G.S.Nichol	Newcastle	C8 H5 N4 O7 1-,H2 O1,Cs1 1+
1862204	PYIOBR04	10.5517/ccdc.csd.cc20hs3f	W.Clegg, M.R.J.Elsegood	Newcastle	C5 H5 Br1 I1 N1
1862217	SILSUE	10.5517/ccdc.csd.cc20hsjv	W.Clegg, A.J.Edwards	Newcastle	C8 H12 N1 1+,I1 1-
1862223	SILTAL	10.5517/ccdc.csd.cc20hsq1	W.Clegg	Newcastle	C6 H16 N1 1+,N1 P4 S9 1-
1862241	SILTEP	10.5517/ccdc.csd.cc20ht9n	W.Clegg, M.R.J.Elsegood	Newcastle	C21 H24 N3 P1
1862244	SILTIT	10.5517/ccdc.csd.cc20htdr	W.Clegg	Newcastle	C15 H26 N1 O8 P1 S1
1862251	SILTAL01	10.5517/ccdc.csd.cc20htmz	W.Clegg	Newcastle	C6 H16 N1 1+,N1 P4 S9 1-
1905417	NUWLID	10.5517/ccdc.csd.cc21yr2v	W.Clegg, R.W.Harrington	Newcastle	C17 H18 N3 1+,F6 P1 1-
1910620	ROGSUE	10.5517/ccdc.csd.cc2244x9	W.Clegg, R.W.Harrington, L.K.Leary	Newcastle	(C7 H8 Cl1 Cu1 N4 S1)n,2n(C3 H7 N1 O1)
1910621	ROGTAL	10.5517/ccdc.csd.cc2244yb	W.Clegg, R.W.Harrington, L.K.Leary	Newcastle	C13 H12 N6 S1,2(H2 O1)
1910622	ROGTEP	10.5517/ccdc.csd.cc2244zc	W.Clegg, R.W.Harrington, L.K.Leary	Newcastle	C13 H12 N6 S1,C3 H7 N1 O1
1910623	ROGTIT	10.5517/ccdc.csd.cc22450f	W.Clegg, R.W.Harrington, L.K.Leary	Newcastle	C13 H12 N6 S1,H2 O1
1910624	ROGTOZ	10.5517/ccdc.csd.cc22451g	W.Clegg, R.W.Harrington, L.K.Leary	Newcastle	C32 H33 N1 O3
1910626	ROGVAN	10.5517/ccdc.csd.cc22453j	W.Clegg, R.W.Harrington, L.K.Leary	Newcastle	C17 H14 N4 O4 S1,2.5(C3 H7 N1 O1)
1910628	ROGVER	10.5517/ccdc.csd.cc22455l	W.Clegg, R.W.Harrington, L.K.Leary	Newcastle	(C7 H7 Cl1 Cu1 N4 S1)n,C3 H7 N1 O1
1910629	ROGVIV	10.5517/ccdc.csd.cc22456m	W.Clegg, R.W.Harrington, L.K.Leary	Newcastle	C14 H16 N8 Ni1 S2 2+,C2 H6 O1,2(N1 O3 1-)
1910630	ROGVOB	10.5517/ccdc.csd.cc22457n	W.Clegg, R.W.Harrington, L.K.Leary	Newcastle	(C11 H12 N4 O4 S1 Zn1)n,H2 O1
1910631	ROGVUH	10.5517/ccdc.csd.cc22458p	W.Clegg, R.W.Harrington, L.K.Leary	Newcastle	C14 H16 N8 Ni1 S2 2+,C3 H7 N1 O1,O4 S1 2-
1910632	ROGWAO	10.5517/ccdc.csd.cc22459q	W.Clegg, R.W.Harrington, L.K.Leary	Newcastle	C14 H17 Cl1 Cu1 N8 S2 1+,Cl1 1-
1910876	ROJJOS	10.5517/ccdc.csd.cc224f5v	W.Clegg, R.W.Harrington	Newcastle	C7 H8 N4 S1,2(H2 O1)
1911011	REZMUF01	10.5517/ccdc.csd.cc224kjb	W.Clegg, R.W.Harrington	International	C19 H22 N2 O2
1911013	TCTPAG04	10.5517/ccdc.csd.cc224kld	W.Clegg, R.W.Harrington	International	C74 H60 Ag2 N2 P4 S2
1911014	POMQUE01	10.5517/ccdc.csd.cc224kmf	W.Clegg, R.W.Harrington	International	C21 H22 Br2 Co1 N2
1911015	XUBKOV01	10.5517/ccdc.csd.cc224kng	W.Clegg, R.W.Harrington	International	C21 H22 Cl2 Co1 N2
1911016	BIYLIH	10.5517/ccdc.csd.cc224kng	W.Clegg, R.W.Harrington	International	(C21 H22 Cu1 I1 N2)n
1911017	BIYLON	10.5517/ccdc.csd.cc224kqj	W.Clegg, R.W.Harrington	International	(C22 H22 Cu1 N3 S1)n
1911793	SOGKEE01	10.5517/ccdc.csd.cc225crd	W.Clegg, S.T.Liddle	Newcastle	(C8 H19 K1 O2)n
1911794	JIZWAT	10.5517/ccdc.csd.cc225csf	W.Clegg, S.T.Liddle	Newcastle	C16 H32 Li1 O8 1+,2(C11 H10 N2),I1 1-
1911795	JIZWEX	10.5517/ccdc.csd.cc225ctg	W.Clegg, S.T.Liddle	Newcastle	C20 H37 K1 N2 O6 Si1
1922463	WUKJER01	10.5517/ccdc.csd.cc22jgy1	W.Clegg, R.W.Harrington, A.S.Richardson	Newcastle	C7 H8 N4 S1
1922464	ROMMEM01	10.5517/ccdc.csd.cc22jgz2	W.Clegg, R.W.Harrington, A.S.Richardson	Newcastle	C7 H8 N4 S1
1922465	KOMVUG	10.5517/ccdc.csd.cc22jh04	W.Clegg, R.W.Harrington, A.S.Richardson	Newcastle	C7 H8 Cd1 Cl2 N4 S1,C3 H7 N1 O1

1922468	KOMXES	10.5517/ccdc.csd.cc22jh37	W.Clegg, R.W.Harrington, A.S.Richardson	Newcastle	C7 H12 Cd1 N5 O5 S1 1+,N1 O3 1-
1922471	KOMXIW	10.5517/ccdc.csd.cc22jh6b	W.Clegg, R.W.Harrington, A.S.Richardson	Newcastle	C18 H32 Co1 N8 O4 S2 2+,2(N1 O3 1-)
1922472	KOMXOC	10.5517/ccdc.csd.cc22jh7c	W.Clegg, R.W.Harrington, A.S.Richardson	Newcastle	(C11 H14 N4 O4 S1 Zn1)n,0.75(C3 H7 N1 O1)
1922485	KOMXUI	10.5517/ccdc.csd.cc22jhns	W.Clegg, R.W.Harrington, A.S.Richardson	Newcastle	C42 H42 Cu6 N24 S6,4(C2 H6 O1),3(H2 O1)
1922486	KOMYAP	10.5517/ccdc.csd.cc22jhpt	W.Clegg, R.W.Harrington, A.S.Richardson	Newcastle	C42 H42 Cu6 N24 S6,6(C3 H7 N1 O1),4(C2 H6 O1)
1922490	KONBEX	10.5517/ccdc.csd.cc22jhty	W.Clegg, R.W.Harrington, A.S.Richardson	Newcastle	C14 H24 Cu1 N8 O6 2+,H12 Cu1 O6 2+,2(O4 S1 2-)
1922491	KONBIB	10.5517/ccdc.csd.cc22jhvz	W.Clegg, R.W.Harrington, A.S.Richardson	Newcastle	C14 H16 N8 Ni1 O2 2+,2(I3 1-),0.5(H2 O1)
1922492	KONBOH	10.5517/ccdc.csd.cc22jhw0	W.Clegg, R.W.Harrington, A.S.Richardson	Newcastle	(C11 H14 N4 O4 Pb1 S1)n
1923051	GOGBUC	10.5517/ccdc.csd.cc22k2xn	W.Clegg, R.W.Harrington, H.A.Latchem	Newcastle	C7 H9 N4 S1 1+,C2 H2 N1 O3 1-
1923054	GOGCAJ	10.5517/ccdc.csd.cc22k30s	W.Clegg, R.W.Harrington, H.A.Latchem	Newcastle	C7 H9 N4 S1 1+,C7 H4 N1 O4 1-
1923055	GOGCEN	10.5517/ccdc.csd.cc22k31t	W.Clegg, R.W.Harrington, H.A.Latchem	Newcastle	C7 H9 N4 S1 1+,C6 H3 N2 O4 1-
1937637	TOQNAR	10.5517/ccdc.csd.cc2318fw	w.Clegg, R.W.Harrington, J.A.Knotts	Newcastle	C7 H9 N4 S1 1+,C8 H5 O4 1-,C3 H7 N1 O1,H2 O1
1937638	TOQNEV	10.5517/ccdc.csd.cc2318gx	W.Clegg, R.W.Harrington, J.A.Knotts	Newcastle	2(C7 H9 N4 S1 1+),C4 H2 O4 2-
1937644	TOQNIZ	10.5517/ccdc.csd.cc2318n3	W.Clegg, R.W.Harrington, J.A.Knotts	Newcastle	C7 H9 N4 S1 1+,C7 H4 N1 O4 1-,H2 O1
1937647	TOQNOF	10.5517/ccdc.csd.cc2318r6	W.Clegg, R.W.Harrington, J.A.Knotts	Newcastle	C7 H9 N4 S1 1+,C7 H8 N4 S1,C7 H4 N1 O4 1-,2(H2 O1)
1937648	TOQNUL	10.5517/ccdc.csd.cc2318s7	W.Clegg, R.W.Harrington, J.A.Knotts	Newcastle	C7 H8 N4 S1,C7 H5 N1 O4,C3 H7 N1 O1,H2 O1
1937650	TOQPAT	10.5517/ccdc.csd.cc2318v9	W.Clegg, R.W.Harrington, J.A.Knotts	Newcastle	2(C7 H9 N4 S1 1+),C8 H4 O4 2-
1937651	TOQPEX	10.5517/ccdc.csd.cc2318wb	W.Clegg, R.W.Harrington, J.A.Knotts	Newcastle	C7 H9 N4 S1 1+,C9 H5 O6 1-,C3 H7 N1 O1,H2 O1
1937652	TOQPIB	10.5517/ccdc.csd.cc2318xc	W.Clegg, R.W.Harrington, J.A.Knotts	Newcastle	0.75(C8 H14 O4),1.5(C7 H8 N4 S1),C3 H7 N1 O1,0.5(H2 O1)
1937653	TOQPOH	10.5517/ccdc.csd.cc2318yd	W.Clegg, R.W.Harrington, J.A.Knotts	Newcastle	C7 H9 N4 S1 1+,C5 H7 O4 1-
1937654	TOQPUN	10.5517/ccdc.csd.cc2318zf	W.Clegg, R.W.Harrington, J.A.Knotts	Newcastle	C7 H9 N4 S1 1+,C3 H3 O4 1-
1937655	TOQQAU	10.5517/ccdc.csd.cc23190h	W.Clegg, R.W.Harrington, J.A.Knotts	Newcastle	C7 H8 N4 S1,0.75(C4 H6 O4),0.5(H2 O1)
1937656	TOQQEY	10.5517/ccdc.csd.cc23191j	W.Clegg, R.W.Harrington, J.A.Knotts	Newcastle	C7 H9 N4 O1 1+,2(H2 O1),Cl1 1-
1937658	LOFQIJ	10.5517/ccdc.csd.cc23193l	W.Clegg, R.W.Harrington, J.A.Knotts	Newcastle	C7 H9 N4 O1 1+,C6 H3 N2 O4 1-
1941266	YOMQUP	10.5517/ccdc.csd.cc2351hv	W.Clegg, R.W.Harrington, S.E.Sanders	Newcastle	C9 H9 N3 O2 S1,H2 O1
1941267	YOMRAW	10.5517/ccdc.csd.cc2351jw	W.Clegg, R.W.Harrington, S.E.Sanders	Newcastle	C17 H14 N4 O5,H2 O1,x(C2 H6 O1)
1941268	HOTNIO01	10.5517/ccdc.csd.cc2351kx	W.Clegg, R.W.Harrington, S.E.Sanders	Newcastle	C7 H9 N4 S1 1+,N1 O3 1-,H2 O1
1941275	YOMTAY	10.5517/ccdc.csd.cc2351s4	W.Clegg, R.W.Harrington, S.E.Sanders	Newcastle	(C14 H14 N8 Ni1 S2)n
1941304	YOMTEC	10.5517/ccdc.csd.cc2352q3	W.Clegg, R.W.Harrington	Newcastle	(C10 H12 N4 O5 S1 Zn1)n,2(C3 H7 N1 O1)
1950281	JORCUR	10.5517/ccdc.csd.cc23gf9b	W.Clegg, A.J.Edwards	Newcastle	C17 H16 F2 N4 O5
1950282	JORDAY	10.5517/ccdc.csd.cc23gfbc	W.Clegg, R.A.Coxall	International	C15 H36 Ag1 N6 S3 1+,Cl1 O4 1-
1950283	KEKCUY01	10.5517/ccdc.csd.cc23gfcd	W.Clegg	International	C62 H72 N2 P4 Pt2 S2 2+,2(C24 H20 B1 1-)
1950284	JORDIG	10.5517/ccdc.csd.cc23gfdf	W.Clegg, A.J.Scott	International	C20 H16 B2 O4
1950363	JOSFUV	10.5517/ccdc.csd.cc23ghy1	W.Clegg, A.J.Edwards	International	C8 H20 Cu1 N5 O2 1+,Cl1 O4 1-,H2 O1

1951671	PORSAT	10.5517/ccdc.csd.cc23hw4n	W.Clegg	UK	C13 H12 F2 N6 O1
1951672	IVUQOF05	10.5517/ccdc.csd.cc23hw5p	W.Clegg	UK	C13 H12 F2 N6 O1
1951673	PORSOH	10.5517/ccdc.csd.cc23hw6q	W.Clegg	UK	2(C12 H30 Li1 O3 1+),C72 B6 Cl2 F60 Ti2 2-,2(C6 D6)
1951674	PORSUN	10.5517/ccdc.csd.cc23hw7r	W.Clegg, M.R.J.Elsegood	UK	C15 H29 N3 O5
1951675	PORTAU	10.5517/ccdc.csd.cc23hw8s	W.Clegg	UK	C60 H44 Eu1 O8 1-,C6 H16 N1 1+,0.81(C2 H6 O1)
1951676	FIDNIP03	10.5517/ccdc.csd.cc23hw9t	W.Clegg	UK	C60 H44 Eu1 O8 1-,C6 H16 N1 1+
1961783	TOWKOI	10.5517/ccdc.csd.cc23vdbq	W.Clegg, C.J.Matthews	UK	C54 H54 Mn4 N36 8+,8(Cl1 O4 1-)
1961785	TOWKUO	10.5517/ccdc.csd.cc23vdds	W.Clegg, C.J.Matthews	UK	C224 H160 Mn12 N96 O16 8+,8(C1 F3 O3 S1 1-)
1961786	TOWLAV	10.5517/ccdc.csd.cc23vdft	W.Clegg, C.J.Matthews	UK	C68 H52 Cr2 Cu2 N24 O4 6+,6(Cl1 O4 1-)
1961787	TOWLEZ	10.5517/ccdc.csd.cc23vdgv	W.Clegg, C.J.Matthews	UK	C68 H52 N24 Ni4 O4 4+,4(Cl1 O4 1-)
1961789	TOWLOJ	10.5517/ccdc.csd.cc23vdjx	W.Clegg, C.J.Matthews	UK	C68 H52 Fe4 N24 O4 6+,6(Cl1 O4 1-)
1961794	TOWLUP	10.5517/ccdc.csd.cc23vdp2	W.Clegg, C.J.Matthews	UK	C68 H52 Co4 N24 O4 4+,4(Cl1 O4 1-)
1961795	TOWMAW	10.5517/ccdc.csd.cc23vdq3	W.Clegg, C.J.Matthews	UK	C112 H84 Mn4 N48 O8 4+,4(Cl1 O4 1-)
1961796	TOWMEA	10.5517/ccdc.csd.cc23vdr4	W.Clegg, C.J.Matthews	UK	C56 H52 Mn4 N12 O12 2+,4(C2 H6 O1),2(Cl1 O4 1-)
1961797	TOWMIE	10.5517/ccdc.csd.cc23vds5	W.Clegg, C.J.Matthews	UK	C168 H136 Mn16 N96 O24 8+,8(C1 F3 O3 S1 1-)
1963804	LOZBIO	10.5517/ccdc.csd.cc23xhj2	W.Clegg, C.J.Matthews	UK	C66 H66 Cu7 F18 N24 O31 S6 4+,4(C1 F3 O3 S1 1-)
1963814	LOZBOU	10.5517/ccdc.csd.cc23xhvd	W.Clegg, C.J.Matthews	UK	C224 H160 Mn16 N96 O16 16+,16(C1 F3 O3 S1 1-)
1963815	LOZBUA	10.5517/ccdc.csd.cc23xhwf	W.Clegg, C.J.Matthews	UK	C144 H144 Cu20 N80 O32 24+,24(Cl1 O4 1-)
1963819	LOZCAH	10.5517/ccdc.csd.cc23xj0l	W.Clegg, C.J.Matthews	UK	C68 H52 Fe4 N24 O4 6+,6(Cl1 O4 1-)
1963820	LOZCEL	10.5517/ccdc.csd.cc23xj1m	W.Clegg, C.J.Matthews	UK	C48 H48 Fe4 N22 O8 6+,6(Cl1 O4 1-)
1964690	WOWBAO	10.5517/ccdc.csd.cc23yf3m	W.Clegg	UK	C12 H13 N1 O4
1964691	WOWBES	10.5517/ccdc.csd.cc23yf4n	W.Clegg	UK	C17 H41 N8 O1 P3,0.5(C4 H8 O1)
1964692	WOWBIW	10.5517/ccdc.csd.cc23yf5p	W.Clegg	UK	(C20 H16 Cu4 I4 N4 S4)n,0.67(C2 H3 N1)
1964693	WOWBOC	10.5517/ccdc.csd.cc23yf6q	W.Clegg	UK	C14 H10 Cl1 N3 O2
1964694	WOWBUI	10.5517/ccdc.csd.cc23yf7r	W.Clegg	UK	C20 H16 N2 O2 S1
1964695	WOWCAP	10.5517/ccdc.csd.cc23yf8s	W.Clegg	UK	C22 H34 O2 S1
1964696	WOWCET	10.5517/ccdc.csd.cc23yf9t	W.Clegg	UK	C9 H13 F1 N2 O3 Si1,C7 H8
1964697	WOWCIX	10.5517/ccdc.csd.cc23yfbv	W.Clegg	UK	C34 H42 Fe1 N10 2+,2(C8 N4 Ni1 S4 1-)
1964698	WOWCOD	10.5517/ccdc.csd.cc23yfcw	W.Clegg	UK	C14 H17 N2 O2 Si1 1+,0.5(H2 O1),Cl1 1-
1964699	WOWCUJ	10.5517/ccdc.csd.cc23yfdx	W.Clegg	UK	0.6(C20 H26 Cl1 N3 Rh1 1+),B1 F4 1-,0.4(C20 H26 Br1 N3 Rh1 1+)
1964700	WOWDAQ	10.5517/ccdc.csd.cc23yffy	W.Clegg	UK	(C20 H22 Mn1 O5 Si1)n
1964875	WOXHUP	10.5517/ccdc.csd.cc23ym2s	W.Clegg	UK	C6 H7 F1 N2
1964877	WOXJAX	10.5517/ccdc.csd.cc23ym4v	W.Clegg	UK	C14 H11 N3 O2
1964880	WOXJEB	10.5517/ccdc.csd.cc23ym7y	W.Clegg	UK	C40 H24 Cl2 N3 O4 P3
1964954	WOXRUZ	10.5517/ccdc.csd.cc23ypmd	W.Clegg	UK	C25 H23 Fe1 N3 O2
1964976	ILENAO01	10.5517/ccdc.csd.cc23yqb4	W.Clegg	UK	C6 H4 Br2 S3
1965602	MORYIE	10.5517/ccdc.csd.cc23zcj0	W.Clegg	UK	C26 H36 O4 Si2
1965610	MORYOK	10.5517/ccdc.csd.cc23zcs8	W.Clegg	UK	C34 H39 Br1 Fe1 N2 P1 Pd1 1+,C1 H1 Cl3,Br1 1-
1965611	MORYUQ	10.5517/ccdc.csd.cc23zct9	W.Clegg	UK	C60 H90 Fe1 P2 2+,2(C1 H3 O3 S1 1-),4(C1 H4 O1)
1965612	MORZAX	10.5517/ccdc.csd.cc23zcvb	W.Clegg	UK	C68 H89 Fe1 P2 1+,C1 H3 O3 S1 1-,3.5(C1 H4 O1)
1965629	MOSCEF	10.5517/ccdc.csd.cc23zddx	W.Clegg	Newcastle	C9 H8 N2 O2
1965630	MOSCIJ	10.5517/ccdc.csd.cc23zdfy	W.Clegg	Newcastle	C9 H8 N2 O2

1965631	MOSCOP	10.5517/ccdc.csd.cc23zdgz	W.Clegg	UK	C7 H5 N1 O4
1965845	MOTLOZ	10.5517/ccdc.csd.cc23zmc3	W.Clegg	UK	C58 H46 Fe1 I2 Ni1 O1 P4
1965846	MOTLUF	10.5517/ccdc.csd.cc23zmd4	W.Clegg	UK	C52 H52 Br2 Fe1 N2 Ni1 Si2,C1 H2 Cl2
1965847	MOTMEQ	10.5517/ccdc.csd.cc23zmf5	W.Clegg	UK	C106 H138 Ag4 N16 O12 P6,C106 H138 Ag4 N16 O12 P6
1965849	MOTMAM	10.5517/ccdc.csd.cc23zmh7	W.Clegg	UK	C110 H144 Ag4 N18 O12 P6,2(C1 H1 Cl3)
1966125	MUCROU	10.5517/ccdc.csd.cc23zxdf	W.Clegg	UK	C11 H17 I1 N1 1+,I1 1-
1966126	MUCRUA	10.5517/ccdc.csd.cc23zxfg	W.Clegg	UK	C16 H24 Br2 Fe1 N2 Ni1
1966127	MUCSAH	10.5517/ccdc.csd.cc23zxjk	W.Clegg	UK	C32 H12 B1 F24 1-,C12 H24 N1 1+
1966128	EGOLIX02	10.5517/ccdc.csd.cc23zxhj	W.Clegg	UK	C12 H8 Fe1 N2
1966129	MUCSOV	10.5517/ccdc.csd.cc23zxgh	W.Clegg	UK	C8 H1 I4 N1 O2,C2 H6 O1 S1
1966130	QIXVAU02	10.5517/ccdc.csd.cc23zxkl	W.Clegg	UK	C13 H18 B1 Fe1 N1 O2
1966131	MUCTAI	10.5517/ccdc.csd.cc23zxlm	W.Clegg	UK	C18 H12 Fe1 N2
1966152	QORPIZ	10.5517/ccdc.csd.cc23zy8b	W.Clegg	UK	C27 H18 B1 F2 N3 O6 S2,0.25(C4 H8 O2)
1966153	ACANIL07	10.5517/ccdc.csd.cc23zy9c	W.Clegg	UK	C8 H9 N1 O1
1967471	HOTNOW	10.5517/ccdc.csd.cc2419tb	W.Clegg	UK	C13 H27 Cl2 N6 O1 P3
1967612	USESUH01	10.5517/ccdc.csd.cc241gc1	W.Clegg	UK	C6 H14 Cl8 N8 P6
1967613	HOWJOV	10.5517/ccdc.csd.cc241gd2	W.Clegg	UK	C6 H12 Cl3 N4 O2 P3
1967827	HOYVID	10.5517/ccdc.csd.cc241p96	W.Clegg	UK	C32 H12 B1 F24 1-,C21 H32 N2 O1,C12 H24 N1 1+
1967831	HOYVOJ	10.5517/ccdc.csd.cc241pfb	W.Clegg	UK	C15 H16 Fe1 O3
1967834	OHIMEX01	10.5517/ccdc.csd.cc241pjf	W.Clegg	UK	C16 H28 Cl4 Pt2
1972785	RUCQUE	10.5517/ccdc.csd.cc246v7g	W.Clegg, D.C.R.Hockless	UK	C30 H45 Cl4 O4 Ta3,C7 H8
1972786	XBPYRC03	10.5517/ccdc.csd.cc246v8h	W.Clegg, M.R.J.Elsegood	UK	C20 H20 Br6 Cu4 N4 O1
1973413	EQEKAO01	10.5517/ccdc.csd.cc247hhd	W.Clegg, M.R.J.Elsegood	UK	C54 H78 Mo1 N2 O2
1973420	VEXDUA01	10.5517/ccdc.csd.cc247hqm	W.Clegg, M.R.J.Elsegood	UK	C38 H48 Cr1 N2
1973422	VOTGAM01	10.5517/ccdc.csd.cc247hsp	W.Clegg, M.R.J.Elsegood	UK	C18 H4 F18 Zn1
1973423	BOXYEV	10.5517/ccdc.csd.cc247htq	W.Clegg, M.R.J.Elsegood	UK	C74 H95 N1 O11 V3 1-,C4 H12 N1 1+,3(C2 H3 N1)
1973424	NACTEQ03	10.5517/ccdc.csd.cc247hvr	W.Clegg, M.R.J.Elsegood	UK	C25 H21 P1
1975926	YIXSIH01	10.5517/ccdc.csd.cc24b3k5	W.Clegg, G.S.Nichol	International	C30 H44 B2 N12 Zn1
1975930	JUDTEK	10.5517/ccdc.csd.cc24b3p9	W.Clegg, L.Russo	International	0.4(C16 H24 Cl2 Cu2 N8 S4),0.6(C16 H24 Br1 Cl1 Cu2 N8 S4)
1975931	NAMJIV01	10.5517/ccdc.csd.cc24b3qb	W.Clegg, R.W.Harrington	International	C8 H12 Cl2 Co1 N4 S2
1975932	JUDTOU	10.5517/ccdc.csd.cc24b3rc	W.Clegg, R.W.Harrington	International	C10 H20 Cl2 Co1 N4 S2
1975934	JUDTUA	10.5517/ccdc.csd.cc24b3tf	W.Clegg, L.Russo	International	C15 H22 B1 Cl3 Cu3 N6 S3 W1 1-,C8 H20 N1 1+
1979166	SUFHUZ	10.5517/ccdc.csd.cc24fh25	W.Clegg	UK	C42 H48 Br2 Ir1 N4,C7 H8
1979167	SUFJAH	10.5517/ccdc.csd.cc24fh36	W.Clegg	UK	C129 H102 Ag6 F12 N13 O12 P9 S4 2+,2(C1 F3 O3 S1 1-)
1980478	GEVBIU01	10.5517/ccdc.csd.cc24gvdw	W.Clegg, M.R.J.Elsegood	Newcastle	C21 H17 N1 O2
1980479	FULXOC	10.5517/ccdc.csd.cc24gvfx	W.Clegg, M.R.J.Elsegood	Newcastle	C25 H23 N1 O4
1980480	FULXUI	10.5517/ccdc.csd.cc24gvgy	W.Clegg, M.R.J.Elsegood	Newcastle	C29 H26 N2 O1
1980482	FULYAP	10.5517/ccdc.csd.cc24gvj0	W.Clegg, M.R.J.Elsegood	Newcastle	C24 H23 N1 O3
1980484	FULYIX	10.5517/ccdc.csd.cc24gvl2	W.Clegg, M.R.J.Elsegood	Newcastle	C11 H17 N5 O2,C1 H4 O1
1980485	FULYOD	10.5517/ccdc.csd.cc24gvm3	W.Clegg, R.W.Harrington	Newcastle	C17 H19 Cl1 N2 O2
1980486	FULYUJ	10.5517/ccdc.csd.cc24gvn4	W.Clegg, R.W.Harrington	Newcastle	C26 H24 N2 O3
1980487	FULZAQ	10.5517/ccdc.csd.cc24gvp5	W.Clegg, R.W.Harrington	Newcastle	C21 H17 N1 O3 S2

1980488	FULZEU	10.5517/ccdc.csd.cc24gvq6	W.Clegg, R.W.Harrington	Newcastle	C25 H23 N1 O3 S1
1980489	FULZIY	10.5517/ccdc.csd.cc24gvr7	W.Clegg, R.W.Harrington	Newcastle	C25 H19 N1 O3 S1
1980490	FULZOE	10.5517/ccdc.csd.cc24gvs8	W.Clegg, R.W.Harrington	Newcastle	C12 H7 N1 O3 S1
1980491	FULZUK	10.5517/ccdc.csd.cc24gvt9	W.Clegg, R.W.Harrington	Newcastle	C12 H7 N1 O4,C1 H4 O1
1980492	FUMBAT	10.5517/ccdc.csd.cc24gvvb	W.Clegg, R.W.Harrington	Newcastle	C25 H21 N1 O4,0.5(C1 H4 O1)
1980493	FUMBEX	10.5517/ccdc.csd.cc24gvwc	W.Clegg, R.W.Harrington	Newcastle	C7 H4 F3 N3 O4 S1
1980494	FUMBIB	10.5517/ccdc.csd.cc24gvxd	W.Clegg, R.W.Harrington	Newcastle	C13 H19 N5 O1
1980495	FUMBOH	10.5517/ccdc.csd.cc24gvyf	W.Clegg, R.W.Harrington	Newcastle	C10 H17 N3 O1 S1
1980496	FUMBUN	10.5517/ccdc.csd.cc24gvzg	W.Clegg, R.W.Harrington	Newcastle	C23 H19 N1 O3 S1
1980497	FUMCAU	10.5517/ccdc.csd.cc24gw0j	W.Clegg, R.W.Harrington	Newcastle	C18 H18 B1 N1 O5
1980498	FUMCEY	10.5517/ccdc.csd.cc24gw1k	W.Clegg, R.W.Harrington	Newcastle	C26 H15 N1 O2 S1
1980499	FUMCIC	10.5517/ccdc.csd.cc24gw2l	W.Clegg, R.W.Harrington	Newcastle	C14 H12 F3 N1 O6 S1
1980500	FUMCOI	10.5517/ccdc.csd.cc24gw3m	W.Clegg, R.W.Harrington	Newcastle	C14 H11 Br1 Cl2 O1 S1
1980501	FUMCUO	10.5517/ccdc.csd.cc24gw4n	W.Clegg, R.W.Harrington	Newcastle	C13 H19 N5 O1
1980502	FUMDAV	10.5517/ccdc.csd.cc24gw5p	W.Clegg, R.W.Harrington	Newcastle	C12 H7 Br1 O1 S1
1980503	FUMDEZ	10.5517/ccdc.csd.cc24gw6q	W.Clegg, R.W.Harrington	Newcastle	C12 H6 Cl1 N1 O2 S1
1980504	FUMDID	10.5517/ccdc.csd.cc24gw7r	W.Clegg, R.W.Harrington	Newcastle	C16 H14 Br1 N1 O2 S1
1980505	FUMDOJ	10.5517/ccdc.csd.cc24gw8s	W.Clegg, R.W.Harrington	Newcastle	C12 H11 Cl1 N4 O2
1980506	FUMDUP	10.5517/ccdc.csd.cc24gw9t	W.Clegg, R.W.Harrington	Newcastle	C11 H10 Cl1 N5 O3 S1,C1 H2 Cl2
1980507	FUMFAX	10.5517/ccdc.csd.cc24gwbv	W.Clegg, R.W.Harrington	Newcastle	C25 H26 N6 O4 S1,C1 H4 O1
1980508	FUMFEB	10.5517/ccdc.csd.cc24gwcw	W.Clegg, R.W.Harrington	Newcastle	C25 H23 Cl1 N2 O4
1980509	FUMFIF	10.5517/ccdc.csd.cc24gwdx	W.Clegg, R.W.Harrington	Newcastle	C21 H14 Cl2 N2 O4
1980510	FUMFOL	10.5517/ccdc.csd.cc24gwfy	W.Clegg, R.W.Harrington	Newcastle	C22 H17 Cl1 N2 O4
1980511	FUMFUR	10.5517/ccdc.csd.cc24gwgz	W.Clegg, R.W.Harrington	Newcastle	C13 H9 Br1 O2 S2
1980512	FUMGAY	10.5517/ccdc.csd.cc24gwh0	W.Clegg, R.W.Harrington	Newcastle	C19 H24 Cl1 N6 O2 S1 1+,C1 H1 O2 1-
1980513	FUMGEC	10.5517/ccdc.csd.cc24gwj1	W.Clegg, R.W.Harrington	Newcastle	C25 H20 N2 O3 S1
1980514	FUMGIG	10.5517/ccdc.csd.cc24gwk2	W.Clegg, R.W.Harrington	Newcastle	C28 H25 N1 O3 S1
1980515	JEFMAJ01	10.5517/ccdc.csd.cc24gwl3	W.Clegg, R.W.Harrington	Newcastle	C7 H9 N1 O4 S1
1980516	JEFMUD01	10.5517/ccdc.csd.cc24gwm4	W.Clegg, R.W.Harrington	Newcastle	C6 H6 N2 O5 S1
1980517	FUMHAZ	10.5517/ccdc.csd.cc24gwn5	W.Clegg, R.W.Harrington	Newcastle	C13 H11 Cl2 N3 O2
1980518	FUMHED	10.5517/ccdc.csd.cc24gwp6	W.Clegg, R.W.Harrington	Newcastle	C18 H19 N5 O3
1980519	FUMHIH	10.5517/ccdc.csd.cc24gwq7	W.Clegg, R.W.Harrington	Newcastle	C15 H12 N6 O1,C4 H8 O1
1980520	FUMHON	10.5517/ccdc.csd.cc24gwr8	W.Clegg, R.W.Harrington	Newcastle	C21 H27 Cl2 N3 Si1,C2 H3 N1
1980521	FUMHUT	10.5517/ccdc.csd.cc24gws9	W.Clegg, R.W.Harrington	Newcastle	C13 H10 N4 O1
1980522	FUMJAB	10.5517/ccdc.csd.cc24gwtb	W.Clegg, R.W.Harrington	Newcastle	C22 H16 Cl3 N1 O2,C1 H1 Cl3
1980523	FUMJEF	10.5517/ccdc.csd.cc24gwvc	W.Clegg, R.W.Harrington	Newcastle	C10 H11 N3 O1
1980525	FUMJOP	10.5517/ccdc.csd.cc24gwxf	W.Clegg, R.W.Harrington	Newcastle	C6 H5 Cl1 N4,C6 H5 Cl1 N4,H2 O1
1980526	FUMJUV	10.5517/ccdc.csd.cc24gwyg	W.Clegg, R.W.Harrington	Newcastle	C16 H14 F3 N1 O6 S1
1980527	FUMKAC	10.5517/ccdc.csd.cc24gwzh	W.Clegg	Newcastle	C27 H25 Cl2 N1 O3
1981224	XUDYUT	10.5517/ccdc.csd.cc24hmgr	W.Clegg, M.R.J.Elsegood	UK	C16 H21 Cl2 N1 Nb1 P1
1981225	XUDZAA	10.5517/ccdc.csd.cc24hmhs	W.Clegg, M.R.J.Elsegood	UK	C18 H32 Cl2 Mo1 N2 O2
1981226	XUDZEE	10.5517/ccdc.csd.cc24hmjt	W.Clegg, M.R.J.Elsegood	UK	C25 H30 Cl1 Mo1 N1

1981227	XUDZII	10.5517/ccdc.csd.cc24hmkv	W.Clegg, M.R.J.Elsegood, P.A.Porrelli	UK	C44 H60 Cr1 N2
1981228	XUDZOO	10.5517/ccdc.csd.cc24hmlw	W.Clegg, M.R.J.Elsegood	UK	C30 H35 F5 Mo1 N2
1981229	XUDZUU	10.5517/ccdc.csd.cc24hmmx	W.Clegg, M.R.J.Elsegood	UK	C14 H19 Cl2 F5 Mo1 N2 O2
1981230	XUFBAE	10.5517/ccdc.csd.cc24hmny	W.Clegg, M.R.J.Elsegood	UK	C42 H58 Mo1 N2
1981231	XUFBEI	10.5517/ccdc.csd.cc24hmpz	W.Clegg, M.R.J.Elsegood	UK	C31 H25 N5
1981546	XUGKOC	10.5517/ccdc.csd.cc24hyvg	W.Clegg, R.W.Harrington	Newcastle	C12 H14 Cl1 N1 O2
1981550	XUGKUI	10.5517/ccdc.csd.cc24hyzl	W.Clegg, A.J.Edwards	Newcastle	C19 H18 N4 O1
1982044	KUBXIR	10.5517/ccdc.csd.cc24jgx2	W.Clegg, C.Jamieson	Newcastle	C23 H21 N1 O2
1982052	KUBZIT	10.5517/ccdc.csd.cc24jh5c	W.Clegg, C.Jamieson	Newcastle	C20 H24 N2 O1
1982053	KUBZOZ	10.5517/ccdc.csd.cc24jh6d	W.Clegg, C.Jamieson	Newcastle	C29 H26 N2 O2
1982054	KUBZUF	10.5517/ccdc.csd.cc24jh7f	W.Clegg, C.Jamieson	Newcastle	C19 H21 N1 O2
1982055	KUCBAO	10.5517/ccdc.csd.cc24jh8g	W.Clegg, C.Jamieson	Newcastle	C35 H30 N2 O1
1982056	KUCBES	10.5517/ccdc.csd.cc24jh9h	W.Clegg, C.Jamieson	Newcastle	C18 H18 N2 O1
1982061	KUCBIW	10.5517/ccdc.csd.cc24jhgn	W.Clegg, C.Jamieson	Newcastle	C27 H28 N2 O2
1982062	KUCBOC	10.5517/ccdc.csd.cc24jhhp	W.Clegg	Newcastle	C17 H21 N5 O2,C1 H4 O1
1982063	KUCBUI	10.5517/ccdc.csd.cc24jhjq	W.Clegg, C.Jamieson	Newcastle	C20 H21 N1 O4
1982064	KUCCAP	10.5517/ccdc.csd.cc24jhkr	W.Clegg, R.W.Harrington	Newcastle	C13 H17 N1 O4 S1
1982065	KUCCET	10.5517/ccdc.csd.cc24jhls	W.Clegg, R.W.Harrington	Newcastle	C17 H15 N1 O4
1982068	KUCCIX	10.5517/ccdc.csd.cc24jhpw	W.Clegg, R.W.Harrington	Newcastle	C12 H7 N1 O4
1982069	KUCCOD	10.5517/ccdc.csd.cc24jhqx	W.Clegg, R.W.Harrington	Newcastle	C24 H20 Cl1 N1 O3,C2 H3 N1
1982071	KUCCUJ	10.5517/ccdc.csd.cc24jhsz	W.Clegg, R.W.Harrington	Newcastle	C23 H20 F1 N1 O4
1982072	KUCDAQ	10.5517/ccdc.csd.cc24jht0	W.Clegg, R.W.Harrington	Newcastle	C13 H6 F3 N1 O6 S1
1982073	KUCDEU	10.5517/ccdc.csd.cc24jhv1	W.Clegg, R.W.Harrington	Newcastle	C13 H6 Br1 Cl2 N1 S1
1982075	KUCDIY	10.5517/ccdc.csd.cc24jhx3	W.Clegg, R.W.Harrington	Newcastle	C12 H6 Br1 Cl1 S1
1982076	KUCDOE	10.5517/ccdc.csd.cc24jhy4	W.Clegg, R.W.Harrington	Newcastle	C10 H11 Cl1 N2 O3
1982077	KUCDUK	10.5517/ccdc.csd.cc24jhz5	W.Clegg, R.W.Harrington	Newcastle	C12 H6 Br1 Cl1 S1
1982079	KUCFAS	10.5517/ccdc.csd.cc24jj18	W.Clegg, R.W.Harrington	Newcastle	C11 H12 Cl1 N3 O6
1982081	KUCFEW	10.5517/ccdc.csd.cc24jj3b	W.Clegg, R.W.Harrington	Newcastle	C13 H8 F3 N1 O3
1982082	KUCFIA	10.5517/ccdc.csd.cc24jj4c	W.Clegg, R.W.Harrington	Newcastle	C12 H17 N1 O1
1982084	BUDYOP03	10.5517/ccdc.csd.cc24jj6f	W.Clegg, R.W.Harrington	Newcastle	C15 H11 F1 O1
1982085	KUCJOK	10.5517/ccdc.csd.cc24jj7g	W.Clegg, R.W.Harrington	Newcastle	C10 H7 F1 N2 O1
1982086	KUCJUQ	10.5517/ccdc.csd.cc24jj8h	W.Clegg, R.W.Harrington	Newcastle	C28 H33 N3 O6
1982087	KUCKAX	10.5517/ccdc.csd.cc24jj9j	W.Clegg, R.W.Harrington	Newcastle	C15 H16 N2 O3
1982089	KUCKEB	10.5517/ccdc.csd.cc24jjcl	W.Clegg, R.W.Harrington	Newcastle	C19 H28 Cl1 N5 O4
1982090	KUCKIF	10.5517/ccdc.csd.cc24jjdm	W.Clegg, R.W.Harrington	Newcastle	C14 H19 N1 O3
1983305	GUBXEJ	10.5517/ccdc.csd.cc24ksl3	W.Clegg, R.W.Harrington	Newcastle	C11 H13 F1 N4 O3
1984729	GUMSIT	10.5517/ccdc.csd.cc24m8jl	W.Clegg	Newcastle	C11 H12 Cl1 N1 O1
1984736	GUMSOZ	10.5517/ccdc.csd.cc24m8rt	W.Clegg	Newcastle	C14 H23 I1 O4
1984737	GUMKIL	10.5517/ccdc.csd.cc24m8sv	W.Clegg	Newcastle	C14 H26 N2 O5 Si1
1984739	GUMKOR	10.5517/ccdc.csd.cc24m8vx	W.Clegg	Newcastle	C7 H10 N2 O4
1984740	GUMKUX	10.5517/ccdc.csd.cc24m8wy	W.Clegg, M.R.J.Elsegood	Newcastle	C11 H11 N3 O2
1984741	GUMLAE	10.5517/ccdc.csd.cc24m8xz	W.Clegg, M.R.J.Elsegood	Newcastle	C15 H15 N5 O4,H2 O1

1984743	GUMLEI	10.5517/ccdc.csd.cc24m8z1	W.Clegg, M.R.J.Elsegood	Newcastle	C6 H7 Br1 O4
1984744	GUMLIM	10.5517/ccdc.csd.cc24m903	W.Clegg, L.Horsburgh	Newcastle	C15 H29 N1 O5
1984745	GUMLOS	10.5517/ccdc.csd.cc24m914	W.Clegg, S.L.Heath	Newcastle	C10 H19 N1 O3
1984746	GUMLUY	10.5517/ccdc.csd.cc24m925	W.Clegg, S.L.Heath	Newcastle	C15 H13 N3 O2
1984747	GUMMAF	10.5517/ccdc.csd.cc24m936	W.Clegg, M.R.J.Elsegood	Newcastle	C16 H25 Cl1 N2 O5
1984750	GUMMEJ	10.5517/ccdc.csd.cc24m969	W.Clegg, M.R.J.Elsegood	Newcastle	C16 H24 N2 O5
1984751	GUMMIN	10.5517/ccdc.csd.cc24m97b	W.Clegg, L.Horsburgh	Newcastle	C12 H13 N1 O3
1984752	GUMMOT	10.5517/ccdc.csd.cc24m98c	W.Clegg, L.Horsburgh	Newcastle	C13 H13 N1 O4
1984753	GUMMUZ	10.5517/ccdc.csd.cc24m99d	W.Clegg, M.R.J.Elsegood	Newcastle	C19 H13 O1 1+,B1 F4 1-
1984762	GUMFIG	10.5517/ccdc.csd.cc24m9lp	W.Clegg	Newcastle	C19 H24 N2 O4
1984763	GUMFOM	10.5517/ccdc.csd.cc24m9mq	W.Clegg, M.R.J.Elsegood	Newcastle	C20 H15 O2 1+,B1 F4 1-
1984764	GUMFUS	10.5517/ccdc.csd.cc24m9nr	W.Clegg	Newcastle	C5 H10 O2 S1
1984765	GUMGAZ	10.5517/ccdc.csd.cc24m9ps	W.Clegg, M.R.J.Elsegood	Newcastle	C11 H21 N1 O4
1984766	GUMGED	10.5517/ccdc.csd.cc24m9qt	W.Clegg	Newcastle	C15 H17 B1 F2 O2 S2
1984767	GUMGIH	10.5517/ccdc.csd.cc24m9rv	W.Clegg, N.C.Martin	Newcastle	C11 H26 Co1 N5 O2 2+,H2 O1,2(Br1 1-)
1984768	GUMGUT	10.5517/ccdc.csd.cc24m9sw	W.Clegg, R.W.Harrington	Newcastle	C23 H19 N1 O2
1984769	GUMHAA	10.5517/ccdc.csd.cc24m9tx	W.Clegg, R.W.Harrington	Newcastle	C7 H8 O6,C6 H6
1984771	GUMHEE	10.5517/ccdc.csd.cc24m9wz	W.Clegg, R.W.Harrington	Newcastle	C15 H15 Cl1 N2 O2
1984772	GUMHII	10.5517/ccdc.csd.cc24m9x0	W.Clegg, R.W.Harrington	Newcastle	C16 H19 N2 O3 1+,Cl1 1-
1984773	GUMHOO	10.5517/ccdc.csd.cc24m9y1	W.Clegg, R.W.Harrington	Newcastle	C14 H10 O1 S2
1984776	GUMHUU	10.5517/ccdc.csd.cc24mb15	W.Clegg	Newcastle	(C43 H60 K2 N12 O12 S2)n,C1 H4 O1
1984777	GUMNAG	10.5517/ccdc.csd.cc24mb26	W.Clegg, R.W.Harrington	Newcastle	C7 H8 F3 N1 O3
1984778	GUMNEK	10.5517/ccdc.csd.cc24mb37	W.Clegg, R.W.Harrington	Newcastle	C19 H23 N5 O3 S1
1984779	GUMNIO	10.5517/ccdc.csd.cc24mb48	W.Clegg, R.W.Harrington	Newcastle	C15 H15 Cl2 F3 N2 O5 S1
1984780	GUMNOU	10.5517/ccdc.csd.cc24mb59	W.Clegg, R.W.Harrington	Newcastle	C14 H9 Cl1 F1 N1 O3
1984781	GUMNUA	10.5517/ccdc.csd.cc24mb6b	W.Clegg, R.W.Harrington	Newcastle	C11 H6 F3 N1 O2
1984782	GUMPAI	10.5517/ccdc.csd.cc24mb7c	W.Clegg, R.W.Harrington	Newcastle	C12 H10 F3 N1 O4
1984783	GUMPEM	10.5517/ccdc.csd.cc24mb8d	W.Clegg	Newcastle	C12 H8 F3 N1 O3
1984784	GUMPIQ	10.5517/ccdc.csd.cc24mb9f	W.Clegg, R.W.Harrington	Newcastle	C11 H12 Cl2 N2 O3
1984785	GUMPOW	10.5517/ccdc.csd.cc24mbbg	W.Clegg, R.W.Harrington	Newcastle	C12 H19 N1 O7,C2 H1 F3 O2
1984787	GUMPUC	10.5517/ccdc.csd.cc24mbdj	W.Clegg, R.W.Harrington	Newcastle	C9 H24 Co1 N5 O3 2+,H2 O1,2(Cl1 1-)
1984790	GUMSUF	10.5517/ccdc.csd.cc24mbhm	W.Clegg, R.W.Harrington	Newcastle	C24 H20 O3
1984791	GUMTAM	10.5517/ccdc.csd.cc24mbjn	W.Clegg, R.W.Harrington	Newcastle	C20 H26 O6 S2
1984793	GUMTEQ	10.5517/ccdc.csd.cc24mblq	W.Clegg, R.W.Harrington	Newcastle	C20 H26 O6 S2
1984794	GUMTIU	10.5517/ccdc.csd.cc24mbmr	W.Clegg, R.W.Harrington	Newcastle	C10 H18 O4
1984796	GUMTOA	10.5517/ccdc.csd.cc24mbpt	W.Clegg, R.W.Harrington	Newcastle	C20 H26 O6 S2
1984800	GUMTUG	10.5517/ccdc.csd.cc24mbty	W.Clegg, R.W.Harrington	Newcastle	C14 H10 N4
1986391	NUFKUX	10.5517/ccdc.csd.cc24p040	W.Clegg	Newcastle	C24 H21 I1 N1 O4 1+,C2 F3 O2 1-
1986398	NUFLEI	10.5517/ccdc.csd.cc24p0c7	W.Clegg, R.W.Harrington	Newcastle	C16 H14 I1 N2 O3 S1 1+,C2 F3 O2 1-
1986399	NUFLIM	10.5517/ccdc.csd.cc24p0d8	W.Clegg, R.W.Harrington	Newcastle	C11 H12 I1 N2 O1 1+,C2 F3 O2 1-
1986401	NUFLOS	10.5517/ccdc.csd.cc24p0gb	W.Clegg, R.W.Harrington	Newcastle	C33 H29 N1 O6 Sn1
1986408	NUFLUY	10.5517/ccdc.csd.cc24p0pk	W.Clegg, R.W.Harrington	Newcastle	C13 H9 Br2 N1 S1

1986409	NUFMAF	10.5517/ccdc.csd.cc24p0ql	W.Clegg, R.W.Harrington	Newcastle	C14 H13 N1 O1 S1
1986410	NUFMEJ	10.5517/ccdc.csd.cc24p0rm	W.Clegg, R.W.Harrington	Newcastle	2(C37 H27 I1 N1 O7 1+),C8 H6 F9 O6 Sn1 1-,C2 F3 O2 1-
1986411	NUFMIN	10.5517/ccdc.csd.cc24p0sn	W.Clegg, R.W.Harrington	Newcastle	C24 H21 I1 N1 O4 1+,C2 F3 O2 1-
1986412	NUFMOT	10.5517/ccdc.csd.cc24p0tp	W.Clegg, R.W.Harrington	Newcastle	C12 H8 Cl2 O4 S2
1986413	NUFMUZ	10.5517/ccdc.csd.cc24p0vq	W.Clegg, R.W.Harrington	Newcastle	C24 H18 O6 S2
1986414	NUFNAG	10.5517/ccdc.csd.cc24p0wr	W.Clegg, R.W.Harrington	Newcastle	C14 H10 Br2 N2 O2 S1
1986415	NUFNEK	10.5517/ccdc.csd.cc24p0xs	W.Clegg, R.W.Harrington	Newcastle	C14 H12 I1 1+,C7 H7 O3 S1 1-
1986416	NUFNIO	10.5517/ccdc.csd.cc24p0yt	W.Clegg, R.W.Harrington	Newcastle	C12 H9 N1 O4 S2
1986417	NUFNOU	10.5517/ccdc.csd.cc24p0zv	W.Clegg, R.W.Harrington	Newcastle	C14 H12 I1 1+,Cl1 1-
1986418	NUFNUA	10.5517/ccdc.csd.cc24p10x	W.Clegg, R.W.Harrington	Newcastle	C17 H16 I1 N2 O1 1+,C2 F3 O2 1-
1986420	NUFPAI	10.5517/ccdc.csd.cc24p12z	W.Clegg, R.W.Harrington	Newcastle	C14 H10 I1 O1 1+,C7 H7 O3 S1 1-
1986421	NUFPEM	10.5517/ccdc.csd.cc24p130	W.Clegg, R.W.Harrington	Newcastle	C14 H12 I1 1+,B1 F4 1-
1986422	NUFPIQ	10.5517/ccdc.csd.cc24p141	W.Clegg, R.W.Harrington	Newcastle	C22 H18 S2 Si1
1986423	NINTIQ01	10.5517/ccdc.csd.cc24p152	W.Clegg, R.W.Harrington	Newcastle	C12 H10 I1 1+,C1 F3 O3 S1 1-
1986460	NUFSUF	10.5517/ccdc.csd.cc24p2c9	W.Clegg, R.W.Harrington	Newcastle	C16 H16 I1 O3 1+,C2 F3 O2 1-
1986461	NUFTAM	10.5517/ccdc.csd.cc24p2db	W.Clegg, R.W.Harrington	Newcastle	C16 H16 I1 O3 1+,C2 F3 O2 1-
1986476	NUFTEQ	10.5517/ccdc.csd.cc24p2wt	W.Clegg, R.W.Harrington	Newcastle	C18 H20 N2 O2 S2
1986477	NUFTIU	10.5517/ccdc.csd.cc24p2xv	W.Clegg, R.W.Harrington	Newcastle	C17 H12 I1 N1 O3
1986478	NUFTOA	10.5517/ccdc.csd.cc24p2yw	W.Clegg, R.W.Harrington	Newcastle	C5 H5 I1 N1 1+,C2 F3 O2 1-
1986479	NUGKUY	10.5517/ccdc.csd.cc24p2zx	W.Clegg, R.W.Harrington	Newcastle	C16 H16 I1 O3 1+,C2 F3 O2 1-
1986497	NUGLAF	10.5517/ccdc.csd.cc24p3kj	W.Clegg, R.W.Harrington	Newcastle	C16 H16 I1 O3 1+,C2 F3 O2 1-,0.5(C1 H2 Cl2)
1987521	NUMCOQ	10.5517/ccdc.csd.cc24q5ln	W.Clegg, R.W.Harrington	Newcastle	C16 H17 I1 N1 O2 1+,C2 F3 O2 1-
1987523	NUMCUW	10.5517/ccdc.csd.cc24q5nq	W.Clegg, R.W.Harrington	Newcastle	C12 H8 Br1 F2 N1
1987524	NUMDAD	10.5517/ccdc.csd.cc24q5pr	W.Clegg, R.W.Harrington	Newcastle	C13 H6 Br1 F2 N1 S1
1987525	NUMKIS	10.5517/ccdc.csd.cc24q5qs	W.Clegg, R.W.Harrington	Newcastle	C12 H8 Br1 F2 N1
1987526	NUMCUW01	10.5517/ccdc.csd.cc24q5rt	W.Clegg, R.W.Harrington	Newcastle	C12 H8 Br1 F2 N1
1987527	NUMKUE	10.5517/ccdc.csd.cc24q5sv	W.Clegg, R.W.Harrington	Newcastle	C12 H10 I1 1+,C7 H7 O3 S1 1-
1987528	NUMLAL	10.5517/ccdc.csd.cc24q5tw	W.Clegg, R.W.Harrington	Newcastle	C15 H14 I1 O2 1+,C2 F3 O2 1-
1987529	NUMNER	10.5517/ccdc.csd.cc24q5vx	W.Clegg, R.W.Harrington	Newcastle	C15 H11 F3 I1 O3 S1 1+,C1 F3 O3 S1 1-
1987530	NUMNIV	10.5517/ccdc.csd.cc24q5wy	W.Clegg, R.W.Harrington	Newcastle	C18 H15 I1 N1 O5 1+,C2 F3 O2 1-,C1 H2 Cl2
1987531	NUMNOB	10.5517/ccdc.csd.cc24q5xz	W.Clegg, R.W.Harrington	Newcastle	C11 H10 N2 O1
1989091	IHULIJ	10.5517/ccdc.csd.cc24rt7z	W.Clegg, R.W.Harrington	Newcastle	C15 H14 I1 O2 1+,C2 F3 O2 1-
1989093	IHULOP	10.5517/ccdc.csd.cc24rt91	W.Clegg, R.W.Harrington	Newcastle	C22 H16 N2 O8
1989094	IHULUV	10.5517/ccdc.csd.cc24rtb2	W.Clegg, R.W.Harrington	Newcastle	C14 H11 Cl1 I1 1+,B1 F4 1-,0.5(C1 H2 Cl2)
1989097	IHUMAC	10.5517/ccdc.csd.cc24rtf5	W.Clegg, R.W.Harrington	Newcastle	C24 H21 I1 N1 O1 1+,C2 F3 O2 1-
1989099	IHUMEG	10.5517/ccdc.csd.cc24rth7	W.Clegg, R.W.Harrington	Newcastle	C25 H23 I1 N1 O2 1+,C2 F3 O2 1-
1989100	IHUMIK	10.5517/ccdc.csd.cc24rtj8	W.Clegg, R.W.Harrington	Newcastle	C15 H11 I1 N1 O4 S1 1+,C2 F3 O2 1-
1989101	IHUMOQ	10.5517/ccdc.csd.cc24rtk9	W.Clegg, R.W.Harrington	Newcastle	C15 H11 N1 S1
1989102	YOSNEZ04	10.5517/ccdc.csd.cc24rtlb	W.Clegg, R.W.Harrington	Newcastle	C16 H10
1990239	TUKNIZ	10.5517/ccdc.csd.cc24t088	W.Clegg, R.W.Harrington	Newcastle	C17 H16 N2 O2,C1 H4 O1
1990240	TUKNOF	10.5517/ccdc.csd.cc24t099	W.Clegg, R.W.Harrington	Newcastle	C8 H6 F1 N1 O4
1990241	TUKNUL	10.5517/ccdc.csd.cc24t0bb	W.Clegg, R.W.Harrington	Newcastle	C17 H12 F3 I1 O2,0.5(C4 H10 O1)

1990245	TUKPAT	10.5517/ccdc.csd.cc24t0gg	W.Clegg, R.W.Harrington	Newcastle	C13 H15 I1 O6
1990246	TUKPEX	10.5517/ccdc.csd.cc24t0hh	W.Clegg, R.W.Harrington	Newcastle	C9 H8 F1 N1 O4
1990247	TUKPIB	10.5517/ccdc.csd.cc24t0jj	W.Clegg, R.W.Harrington	Newcastle	C12 H10 Br1 N1 O1
1990249	TUKPOH	10.5517/ccdc.csd.cc24t0ll	W.Clegg, R.W.Harrington	Newcastle	C12 H10 I1 1+,C2 F3 O2 1-
1990250	TUKPUN	10.5517/ccdc.csd.cc24t0mm	W.Clegg, R.W.Harrington	Newcastle	C25 H27 F1 N4 O2
1990254	QAKWEG01	10.5517/ccdc.csd.cc24t0rr	W.Clegg, R.W.Harrington	Newcastle	C18 H22 N4 O2
1990255	KABNUY01	10.5517/ccdc.csd.cc24t0ss	W.Clegg, R.W.Harrington	Newcastle	C25 H27 N5 O4
1990256	TUKQIC	10.5517/ccdc.csd.cc24t0tt	W.Clegg, R.W.Harrington	Newcastle	C14 H12 I1 O2 1+,C2 F3 O2 1-,C1 H2 Cl2
1990257	TUKQOI	10.5517/ccdc.csd.cc24t0vv	W.Clegg, R.W.Harrington	Newcastle	C8 H6 I2 O3 S2
1990258	TUKQUO	10.5517/ccdc.csd.cc24t0ww	W.Clegg, R.W.Harrington	Newcastle	C13 H10 I1 O1 1+,C2 F3 O2 1-
1990259	QUZQAE01	10.5517/ccdc.csd.cc24t0xx	W.Clegg, R.W.Harrington	Newcastle	C18 H24 N4 O1,H2 O1
1990260	TUKREZ	10.5517/ccdc.csd.cc24t0yy	W.Clegg, R.W.Harrington	Newcastle	C11 H8 I1 O1 S1 1+,C2 F3 O2 1-
1990261	TUKRID	10.5517/ccdc.csd.cc24t0zz	W.Clegg, R.W.Harrington	Newcastle	C23 H22 F1 N1 O4
1990262	TUKROJ	10.5517/ccdc.csd.cc24t101	W.Clegg, R.W.Harrington	Newcastle	C7 H9 N1 O2 S1
1990263	TUKRUP	10.5517/ccdc.csd.cc24t112	W.Clegg, R.W.Harrington	Newcastle	C13 H12 I1 O2 S1 1+,C2 F3 O2 1-
1990264	TUKSOK	10.5517/ccdc.csd.cc24t123	W.Clegg, R.W.Harrington	Newcastle	C14 H14 I1 O1 1+,C7 H7 O3 S1 1-,0.5(C4 H10 O1)
1990265	TUKSUQ	10.5517/ccdc.csd.cc24t134	W.Clegg, R.W.Harrington	Newcastle	C11 H8 I1 O1 S1 1+,C2 F3 O2 1-
1991310	LUHYIZ	10.5517/ccdc.csd.cc24v3ty	W.Clegg, R.W.Harrington	Newcastle	C15 H19 N1 O4 S1
1991311	VIZXOS02	10.5517/ccdc.csd.cc24v3vz	W.Clegg, R.W.Harrington	Newcastle	C22 H18 O2
1991312	LUHYUL	10.5517/ccdc.csd.cc24v3w0	W.Clegg, R.W.Harrington	Newcastle	C24 H20 O3
1991313	LUHZAS	10.5517/ccdc.csd.cc24v3x1	W.Clegg, R.W.Harrington	Newcastle	C21 H20 I2 N4 Pd1,C2 H6 O1 S1
1991314	LUHZEW	10.5517/ccdc.csd.cc24v3y2	W.Clegg, R.W.Harrington	Newcastle	C27 H23 N1 O3
1991323	LUJBIE	10.5517/ccdc.csd.cc24v47d	W.Clegg, R.W.Harrington	Newcastle	C72 H56 N2 P2 Pd1 2+,2(F6 Sb1 1-)
1991324	KEZJEF02	10.5517/ccdc.csd.cc24v48f	W.Clegg, R.W.Harrington	Newcastle	C10 H16 N4 2+,2(Br1 1-)
1991325	LUJBUQ	10.5517/ccdc.csd.cc24v49g	W.Clegg, R.W.Harrington	Newcastle	C25 H24 I2 N4 Pd1
1991327	LUJCAX	10.5517/ccdc.csd.cc24v4cj	W.Clegg, R.W.Harrington	Newcastle	C30 H32 N1 P2 Pd1 1+,F6 P1 1-
1991328	XACKER01	10.5517/ccdc.csd.cc24v4dk	W.Clegg, R.W.Harrington	Newcastle	C13 H18 N6 Pd1 2+,C2 H3 N1,2(B1 F4 1-)
1991329	LUJCIF	10.5517/ccdc.csd.cc24v4fl	W.Clegg, R.W.Harrington	Newcastle	C25 H26 N6 Pd1 2+,C2 H3 N1,2(B1 F4 1-)
1991330	LUJCOL	10.5517/ccdc.csd.cc24v4gm	W.Clegg, R.W.Harrington	Newcastle	C11 H12 Br1 N1 O3 S1
1991331	LUJCUR	10.5517/ccdc.csd.cc24v4hn	W.Clegg, R.W.Harrington	Newcastle	C26 H24 N2 O2
1991332	LUJDAY	10.5517/ccdc.csd.cc24v4jp	W.Clegg, R.W.Harrington	Newcastle	C26 H24 Cu1 F6 N4 O6 S2
1991333	TEPBUT07	10.5517/ccdc.csd.cc24v4kq	W.Clegg, R.W.Harrington	Newcastle	C28 H22
1991337	LUJDUS	10.5517/ccdc.csd.cc24v4pv	W.Clegg, R.W.Harrington	Newcastle	C32 H32 N2
1991338	LUJFAA	10.5517/ccdc.csd.cc24v4qw	W.Clegg, R.W.Harrington	Newcastle	C42 H34 N4 O2
1991339	LUJFEE	10.5517/ccdc.csd.cc24v4rx	W.Clegg, R.W.Harrington	Newcastle	C42 H46 Cl1 P2 Ru1 1+,F6 Sb1 1-,2.5(C1 H2 Cl2)
1991753	LUMDIJ	10.5517/ccdc.csd.cc24vl3q	W.Clegg, R.W.Harrington	Newcastle	C8 H13 N1 O1
1991754	LUMDOP	10.5517/ccdc.csd.cc24vl4r	W.Clegg, R.W.Harrington	Newcastle	C18 H17 N1 O3 S1
1991755	LUMDUV	10.5517/ccdc.csd.cc24vl5s	W.Clegg, R.W.Harrington	Newcastle	C17 H12 I1 N1 O3,C2 H6 O1
1991756	LUMTIZ	10.5517/ccdc.csd.cc24vl6t	W.Clegg, R.W.Harrington	Newcastle	C61 H60 N2 O2 P2 Pt1 2+,2.5(C1 H2 Cl2),0.5(H2 O1),2(Cl1 1-)
1991757	LUMTOF	10.5517/ccdc.csd.cc24vl7v	W.Clegg, R.W.Harrington	Newcastle	C42 H48 Cl2 O4 P2 Pt1,C1 H2 Cl2
1991758	LUMTUL	10.5517/ccdc.csd.cc24vl8w	W.Clegg, R.W.Harrington	Newcastle	C50 H48 Cl2 O4 P2 Pd1,3(C1 H2 Cl2)
1991759	LUMVAT	10.5517/ccdc.csd.cc24vl9x	W.Clegg, R.W.Harrington	Newcastle	C42 H48 Cl2 O4 P2 Pd1,4.5(C1 H2 Cl2)

1991760	LUMVEX	10.5517/ccdc.csd.cc24vlby	W.Clegg, R.W.Harrington	Newcastle	C40 H44 Cl2 P2 Pd1,2(C1 H2 Cl2)
1991761	LUMVIB	10.5517/ccdc.csd.cc24vlcz	W.Clegg, R.W.Harrington	Newcastle	C11 H16 N1 O3 P1
1991762	LUMVOH	10.5517/ccdc.csd.cc24vld0	W.Clegg, R.W.Harrington	Newcastle	C13 H18 N1 O4 P1,0.5(H2 O1)
1991763	LUMVUN	10.5517/ccdc.csd.cc24vlf1	W.Clegg, R.W.Harrington	Newcastle	C11 H16 N1 O4 P1
1991764	LUMWAU	10.5517/ccdc.csd.cc24vlg2	W.Clegg, R.W.Harrington	Newcastle	C48 H44 Cl2 Ni1 P2,C4 H10 O1
1991765	LUMWEY	10.5517/ccdc.csd.cc24vlh3	W.Clegg, R.W.Harrington	Newcastle	C40 H44 Cl2 Ni1 P2
1991766	LUMWIC	10.5517/ccdc.csd.cc24vlj4	W.Clegg, R.W.Harrington	Newcastle	C48 H44 Cl2 P2 Pt1,0.5(C6 H14),C1 H2 Cl2
1991767	BOKGIR04	10.5517/ccdc.csd.cc24vlk5	W.Clegg, R.W.Harrington	Newcastle	C8 H12 Cl2 Pt1
1991768	LUMWUO	10.5517/ccdc.csd.cc24vll6	W.Clegg, R.W.Harrington	Newcastle	C48 H56 Cl2 Ni1 P2,3(C1 H2 Cl2)
1991770	LUMXAV	10.5517/ccdc.csd.cc24vln8	W.Clegg, R.W.Harrington	Newcastle	C56 H56 P2 Rh1 1+,B1 F4 1-
1991771	LUMXEZ	10.5517/ccdc.csd.cc24vlp9	W.Clegg, R.W.Harrington	Newcastle	C48 H56 P2 Rh1 1+,B1 F4 1-
1993577	MUMGEJ	10.5517/ccdc.csd.cc24xgyh	W.Clegg, R.W.Harrington	Newcastle	C75 H54 Fe2 N18 4+,4(Cl1 O4 1-)
1993578	MUMGIN	10.5517/ccdc.csd.cc24xgzj	W.Clegg, R.W.Harrington	Newcastle	C8 H8 Br2 N2 O1
1993579	MUMGOT	10.5517/ccdc.csd.cc24xh0l	W.Clegg, R.W.Harrington	Newcastle	C13 H13 N1 O2
1993580	HOLBAM02	10.5517/ccdc.csd.cc24xh1m	W.Clegg, R.W.Harrington	Newcastle	C16 H13 N1 O1
1993581	ABUSIZ01	10.5517/ccdc.csd.cc24xh2n	W.Clegg, R.W.Harrington	Newcastle	C10 H9 N1 O1
1993582	MUMHEK	10.5517/ccdc.csd.cc24xh3p	W.Clegg, R.W.Harrington	Newcastle	C12 H13 N1 O1
1993583	MUMHIO	10.5517/ccdc.csd.cc24xh4q	W.Clegg, R.W.Harrington	Newcastle	C27 H22 N2 O2,0.25(C7 H8)
1993584	MUMHOU	10.5517/ccdc.csd.cc24xh5r	W.Clegg, R.W.Harrington	Newcastle	C13 H13 N3 O2 S1
1993587	MUMHUA	10.5517/ccdc.csd.cc24xh8v	W.Clegg, R.W.Harrington	Newcastle	C26 H24 N2 O2
1993588	MUMJAI	10.5517/ccdc.csd.cc24xh9w	W.Clegg, R.W.Harrington	Newcastle	C12 H12 N2 O2 S1
1993589	MUMJEM	10.5517/ccdc.csd.cc24xhbx	W.Clegg, R.W.Harrington	Newcastle	C27 H19 Cl1 N1 P1 S1
1994100	QUGYAV	10.5517/ccdc.csd.cc24y0ty	W.Clegg, M.R.J.Elsegood	Newcastle	C48 H63 Mo1 N1 O5
1994101	QUGYEZ	10.5517/ccdc.csd.cc24y0vz	W.Clegg, M.R.J.Elsegood	Newcastle	C28 H33 Fe2 N1 O9 P2
1994102	QUGYID	10.5517/ccdc.csd.cc24y0w0	W.Clegg, M.R.J.Elsegood	Newcastle	C27 H28 Fe2 N1 O6 P1
1994103	QUGYOJ	10.5517/ccdc.csd.cc24y0x1	W.Clegg, M.R.J.Elsegood	Newcastle	C31 H28 Fe2 N1 O6 P1
1994104	QUGYUP	10.5517/ccdc.csd.cc24y0y2	W.Clegg, M.R.J.Elsegood	Newcastle	C39 H36 Fe2 O5 P2 S1
1994105	QUGZAW	10.5517/ccdc.csd.cc24y0z3	W.Clegg, M.R.J.Elsegood	Newcastle	C19 H32 N1 P1 Si2
1994106	QUGZEA	10.5517/ccdc.csd.cc24y105	W.Clegg, M.R.J.Elsegood	Newcastle	C25 H26 Fe2 O9 P2,0.5(C1 H2 Cl2)
1994108	QUGZIE	10.5517/ccdc.csd.cc24y127	W.Clegg, M.R.J.Elsegood	Newcastle	C39 H48 Cl1 N3 O1 P2 Pd1 2+,2(Cl1 O4 1-),2(C1 H2 Cl2)
1994110	QUGZOK	10.5517/ccdc.csd.cc24y149	W.Clegg, M.R.J.Elsegood	Newcastle	C48 H58 Cl2 N4 P2 Pt1
1994111	QUGZUQ	10.5517/ccdc.csd.cc24y15b	W.Clegg, M.R.J.Elsegood	Newcastle	C25 H32 Cl1 N2 P1 Pd1
1994112	QUHBAZ	10.5517/ccdc.csd.cc24y16c	W.Clegg, M.R.J.Elsegood	Newcastle	C38 H26 Fe2 O6 P2
1994113	QUHBED	10.5517/ccdc.csd.cc24y17d	W.Clegg, M.R.J.Elsegood	Newcastle	C37 H26 Fe2 O5 P2
1994114	QUHBIH	10.5517/ccdc.csd.cc24y18f	W.Clegg, M.R.J.Elsegood	Newcastle	C24 H29 Cl2 N2 P1 Pd1,2(C1 H1 Cl3)
1994115	QUHBON	10.5517/ccdc.csd.cc24y19g	W.Clegg, A.J.Edwards	Newcastle	C20 H38 Mo1 N2 O2 Si1
1994121	QUHFUX	10.5517/ccdc.csd.cc24y1hn	W.Clegg, M.R.J.Elsegood	Newcastle	C36 H30 Fe2 O6 P2
1994122	QUHGAE	10.5517/ccdc.csd.cc24y1jp	W.Clegg, A.J.Edwards	Newcastle	C24 H40 N2 Si2
1994123	QUHGEI	10.5517/ccdc.csd.cc24y1kq	W.Clegg, A.J.Edwards	Newcastle	C27 H34 N2 P1 Pd1 1+,Cl1 O4 1-
1994128	QUHGIM	10.5517/ccdc.csd.cc24y1qw	W.Clegg, M.R.J.Elsegood	Newcastle	C18 H23 Fe2 O9 P1 S1
1994130	QUHGOS	10.5517/ccdc.csd.cc24y1sy	W.Clegg, A.J.Edwards	Newcastle	C24 H27 N1 P1 Pd1 1+,Cl1 O4 1-
1994790	QUNKUI	10.5517/ccdc.csd.cc24yr2y	W.Clegg, M.R.J.Elsegood	Newcastle	C23 H19 Fe2 O7 P1

1994791	QUNLAP	10.5517/ccdc.csd.cc24yr3z	W.Clegg, M.R.J.Elsegood	Newcastle	C18 H22 Fe2 O6 S2
1994792	QUNLET	10.5517/ccdc.csd.cc24yr40	W.Clegg, M.R.J.Elsegood	Newcastle	C55 H43 Fe2 O10 P3
1994793	QUNLIX	10.5517/ccdc.csd.cc24yr51	W.Clegg, M.R.J.Elsegood	Newcastle	C35 H30 Fe2 O5 P2
1994794	QUNLOD	10.5517/ccdc.csd.cc24yr62	W.Clegg, M.R.J.Elsegood	Newcastle	C26 H28 Fe2 O8 P2
1994795	QUHGAE01	10.5517/ccdc.csd.cc24yr73	W.Clegg, M.R.J.Elsegood	Newcastle	C24 H40 N2 Si2
1994796	QUNMAQ	10.5517/ccdc.csd.cc24yr84	W.Clegg, M.R.J.Elsegood	Newcastle	C27 H31 Fe2 N3 O6 P2
1994797	QUNMEU	10.5517/ccdc.csd.cc24yr95	W.Clegg, M.R.J.Elsegood	Newcastle	C41 H44 Fe2 O9 P4
1994798	QUNMIY	10.5517/ccdc.csd.cc24yrb6	W.Clegg, M.R.J.Elsegood	Newcastle	C42 H44 Fe2 O10 P4
1994799	QUNMOE	10.5517/ccdc.csd.cc24yrc7	W.Clegg, A.J.Edwards	Newcastle	C66 H54 N2 O4 Ti1,1.5(C4 H10 O1)
1994800	QUNMUK	10.5517/ccdc.csd.cc24yrd8	W.Clegg, A.J.Edwards	Newcastle	C26 H26 Fe2 N1 O5 P1
1994801	QUNNAR	10.5517/ccdc.csd.cc24yrf9	W.Clegg, A.J.Edwards	Newcastle	C25 H43 N2 P1 Si2
1994802	QUNNEV	10.5517/ccdc.csd.cc24yrgb	W.Clegg, A.J.Edwards	Newcastle	C61 H48 Fe2 O4 P4
1994803	CEHPIO02	10.5517/ccdc.csd.cc24yrhc	W.Clegg, M.R.J.Elsegood	Newcastle	C5 H5 Cl3 Ti1
1994804	JARNEW01	10.5517/ccdc.csd.cc24yrjd	W.Clegg, M.R.J.Elsegood	Newcastle	C16 H24 Cl2 O2 Ti1
1994805	QUNNUL	10.5517/ccdc.csd.cc24yrkf	W.Clegg, M.R.J.Elsegood	Newcastle	C32 H48 Cl4 Nb2 O5
1994806	QUNPAT	10.5517/ccdc.csd.cc24yrlg	W.Clegg, M.R.J.Elsegood	Newcastle	C42 H44 Cl4 N2 P2 Pd2
1994807	QUNPEX	10.5517/ccdc.csd.cc24yrmh	W.Clegg, M.R.J.Elsegood	Newcastle	C61 H48 Fe2 O4 P4,3(C1 H2 Cl2)
1994808	QUNPIB	10.5517/ccdc.csd.cc24yrnj	W.Clegg, M.R.J.Elsegood	Newcastle	C17 H23 Cl2 N1 O1 Zr1
1997434	YUMXAI	10.5517/ccdc.csd.cc251hc3	W.Clegg, M.R.J.Elsegood	Newcastle	C33 H24 Fe2 O7 P2
1997435	YUMXEM	10.5517/ccdc.csd.cc251hd4	W.Clegg, M.R.J.Elsegood	Newcastle	C42 H44 Cl2 N2 P2 Pd1
1997436	YUMXIQ	10.5517/ccdc.csd.cc251hf5	W.Clegg, M.R.J.Elsegood	Newcastle	C16 H24 N2 O4 Sn1
1997437	YUMXOW	10.5517/ccdc.csd.cc251hg6	W.Clegg, M.R.J.Elsegood	Newcastle	C21 H15 Fe2 O6 P1
1997438	GONPOO01	10.5517/ccdc.csd.cc251hh7	W.Clegg, M.R.J.Elsegood	Newcastle	C25 H19 Cl1 N1 Ni1 O1 P1
1997439	YUMYAJ	10.5517/ccdc.csd.cc251hj8	W.Clegg, M.R.J.Elsegood	Newcastle	C54 H80 Cl2 Li1 N2 O4 Y1,C4 H10 O1
1997440	YUMYEN	10.5517/ccdc.csd.cc251hk9	W.Clegg, M.R.J.Elsegood	Newcastle	C54 H76 Li2 N2 O4
1997441	YUMYIR	10.5517/ccdc.csd.cc251hlb	W.Clegg, M.R.J.Elsegood	Newcastle	C25 H19 N2 Ni1 O4 P1
1997442	YUMYOX	10.5517/ccdc.csd.cc251hmc	W.Clegg, M.R.J.Elsegood	Newcastle	C25 H21 N1 O1 P1 1+,Cl1 1-
1997443	YIYCUF01	10.5517/ccdc.csd.cc251hnd	W.Clegg, M.R.J.Elsegood	Newcastle	C23 H18 Cl1 N2 Ni1 P1
1997444	YUMZAK	10.5517/ccdc.csd.cc251hpf	W.Clegg, M.R.J.Elsegood	Newcastle	C28 H22 N3 Ni1 O1 P1
1997445	YUMZEO	10.5517/ccdc.csd.cc251hqg	W.Clegg, M.R.J.Elsegood	Newcastle	C38 H32 N1 P2 Pd1 1+,C3 H9 Cl2 Sn1 1-,1.5(C1 H2 Cl2)
1997446	YUMZIS	10.5517/ccdc.csd.cc251hrh	W.Clegg, M.R.J.Elsegood	Newcastle	C18 H28 P2
1997447	YUMZOY	10.5517/ccdc.csd.cc251hsj	W.Clegg, M.R.J.Elsegood	Newcastle	C92 H86 Cr2 N4 O6 P4,4(C1 H2 Cl2)
1997448	YUMZUE	10.5517/ccdc.csd.cc251htk	W.Clegg, M.R.J.Elsegood	Newcastle	C46 H42 Cl2 N2 Ni2 O4 P2,3(C1 H2 Cl2)
1997449	YUNBAN	10.5517/ccdc.csd.cc251hvl	W.Clegg, M.R.J.Elsegood	Newcastle	C18 H28 Cl2 P2 Pd1,2(C1 H2 Cl2)
1997450	YUNQIK	10.5517/ccdc.csd.cc251hwm	W.Clegg, M.R.J.Elsegood	Newcastle	C32 H39 N1 O1 Ti1
1997451	YUNBER	10.5517/ccdc.csd.cc251hxn	W.Clegg, M.R.J.Elsegood	Newcastle	C31 H37 N1 O1 Ti1
1997454	YUNBIV	10.5517/ccdc.csd.cc251j0s	W.Clegg, M.R.J.Elsegood	Newcastle	C16 H24 N2 O4 Ti1
1997856	HUFKOL	10.5517/ccdc.csd.cc251xz4	W.Clegg, M.R.J.Elsegood	Newcastle	C28 H28 P2 S2
1997857	HUFKUR	10.5517/ccdc.csd.cc251y06	W.Clegg, M.R.J.Elsegood	Newcastle	C18 H32 Al2 N2 O4
1997858	HUFMED	10.5517/ccdc.csd.cc251y17	W.Clegg, P.A.Champkin	Newcastle	C22 H24 P2
1997859	HUFMIH	10.5517/ccdc.csd.cc251y28	W.Clegg, P.A.Champkin	Newcastle	C32 H34 P2 S2
1997860	HUFMIH01	10.5517/ccdc.csd.cc251y39	W.Clegg, P.A.Champkin	Newcastle	C32 H34 P2 S2

1997861	HUFMUT	10.5517/ccdc.csd.cc251y4b	W.Clegg, M.R.J.Elsegood	Newcastle	C27 H25 Al1 N1 O1 P1
1997862	HUFNAA	10.5517/ccdc.csd.cc251y5c	W.Clegg, M.R.J.Elsegood	Newcastle	C16 H28 Al2 N2 O4
1997863	VIRTEY01	10.5517/ccdc.csd.cc251y6d	W.Clegg, S.T.Liddle	Newcastle	C11 H23 Al1 N2 O1
1997864	HUFNII	10.5517/ccdc.csd.cc251y7f	W.Clegg, M.R.J.Elsegood	Newcastle	C31 H33 Al1 N1 O1 P1,0.5(C7 H8)
1997865	HUFNOO	10.5517/ccdc.csd.cc251y8g	W.Clegg, M.R.J.Elsegood	Newcastle	C34 H34 Cl2 N4 P2 Pd1
1997866	HUFNUU	10.5517/ccdc.csd.cc251y9h	W.Clegg, M.R.J.Elsegood	Newcastle	C24 H20 Cl2 O1 P2 Pd1
1997867	WOXXEM01	10.5517/ccdc.csd.cc251ybj	W.Clegg, S.T.Liddle	Newcastle	C28 H30 Cl2 O2 P2 Pd1
1997868	HUFPEG	10.5517/ccdc.csd.cc251yck	W.Clegg, M.R.J.Elsegood	Newcastle	C40 H34 Cl2 N4 P2 Pd1,2(C1 H2 Cl2)
1997869	HUFPIK	10.5517/ccdc.csd.cc251ydl	W.Clegg, M.R.J.Elsegood	Newcastle	C40 H34 Cl2 N4 P2 Pt1,3(C1 H2 Cl2)
1997870	HUFPOQ	10.5517/ccdc.csd.cc251yfm	W.Clegg, S.T.Liddle	Newcastle	C32 H46 Cl2 P2 Pd1,C1 H2 Cl2
1997871	HUFPUW	10.5517/ccdc.csd.cc251ygn	W.Clegg, M.R.J.Elsegood	Newcastle	C44 H34 Cl2 P2 Pd1
1997872	HUFQAD	10.5517/ccdc.csd.cc251yhp	W.Clegg, P.A.Champkin	Newcastle	C32 H24 Cl2 P2 Pd1
2000024	AHALED	10.5517/ccdc.csd.cc2545xf	W.Clegg, R.W.Harrington	Newcastle	C42 H44 Cl2 N2 P2 Ru1
2000025	AHALIH	10.5517/ccdc.csd.cc2545yg	W.Clegg, R.W.Harrington	Newcastle	C46 H50 Cl2 N2 P2 Ru1
2000026	AHAMAA	10.5517/ccdc.csd.cc2545zh	W.Clegg, R.W.Harrington	Newcastle	C13 H17 N1 O2
2000028	AHAMII	10.5517/ccdc.csd.cc25461l	W.Clegg, R.W.Harrington	Newcastle	C38 H48 Cl2 N2 P2 Ru1
2000029	AHAMOO	10.5517/ccdc.csd.cc25462m	W.Clegg, R.W.Harrington	Newcastle	C32 H38 Al2 Cl1 N2 O5 1+,Al1 Cl4 1-,C1 H2 Cl2
2000030	AHAMUU	10.5517/ccdc.csd.cc25463n	W.Clegg, R.W.Harrington	Newcastle	C14 H17 N1 O2
2000031	AHANAB	10.5517/ccdc.csd.cc25464p	W.Clegg, R.W.Harrington	Newcastle	C52 H62 Al3 Cl1 N4 O8 2+,2(Al1 Cl4 1-),2(C1 H2 Cl2)
2000034	AHANEF	10.5517/ccdc.csd.cc25467s	W.Clegg, R.W.Harrington	Newcastle	C46 H48 Cl2 N2 P2 Ru1,4(C4 H8 O1)
2000035	AHANIJ	10.5517/ccdc.csd.cc25468t	W.Clegg, R.W.Harrington	Newcastle	C24 H26 N2 O1
2000036	AHANOP	10.5517/ccdc.csd.cc25469v	W.Clegg, R.W.Harrington	Newcastle	C21 H26 N2 O1
2000039	AHANUV	10.5517/ccdc.csd.cc2546dy	W.Clegg, R.W.Harrington	Newcastle	C47 H42 Cl2 O2 P2 Pt1,4(C1 H2 Cl2)
2000040	AHAPAD	10.5517/ccdc.csd.cc2546fz	W.Clegg, R.W.Harrington	Newcastle	C32 H36 Ir1 N2 O1 P1 1+,F6 Sb1 1-
2000041	AHAPEH	10.5517/ccdc.csd.cc2546g0	W.Clegg, R.W.Harrington	Newcastle	C31 H32 Cl2 Fe1 N1 P1 Ru1
2000042	AHAPIL	10.5517/ccdc.csd.cc2546h1	W.Clegg, R.W.Harrington	Newcastle	C46 H48 Cl1 N2 P2 Ru1 1+,F6 Sb1 1-,2.5(C1 H1 Cl3)
2000043	AHAPOR	10.5517/ccdc.csd.cc2546j2	W.Clegg, R.W.Harrington	Newcastle	C38 H46 Cl1 N2 P2 Ru1 1+,B1 F4 1-
2000050	AHEKUW	10.5517/ccdc.csd.cc2546r9	W.Clegg, R.W.Harrington	Newcastle	C50 H48 Cl2 O4 P2 Pt1
2000051	AHELAD	10.5517/ccdc.csd.cc2546sb	W.Clegg, R.W.Harrington	Newcastle	C44 H38 Cl2 P2 Pt1,3(C1 H2 Cl2)
2000053	AHELEH	10.5517/ccdc.csd.cc2546vd	W.Clegg, R.W.Harrington	Newcastle	C36 H38 Cl2 Ni1 P2
2000054	AHELIL	10.5517/ccdc.csd.cc2546wf	W.Clegg, R.W.Harrington	Newcastle	C46 H50 Cl2 N2 P2 Ru1,C1 H2 Cl2
2000055	AHELOR	10.5517/ccdc.csd.cc2546xg	W.Clegg, R.W.Harrington	Newcastle	C38 H48 Cl2 N2 P2 Ru1,C1 H2 Cl2
2000056	AHELUX	10.5517/ccdc.csd.cc2546yh	W.Clegg, R.W.Harrington	Newcastle	C50 H48 Cl2 Ni1 O4 P2
2000057	AHEMAE	10.5517/ccdc.csd.cc2546zj	W.Clegg, R.W.Harrington	Newcastle	C42 H48 Cl2 Ni1 O4 P2,C1 H1 Cl3
2002701	CUPNUZ	10.5517/ccdc.csd.cc256z8n	W.Clegg, R.W.Harrington	Newcastle	C12 H14 N1 1+,C9 H10 F3 O3 1-
2002702	CUPPAH	10.5517/ccdc.csd.cc256z9p	W.Clegg, R.W.Harrington	Newcastle	C42 H44 Cl2 N2 P2 Ru1
2002703	CUPPEL	10.5517/ccdc.csd.cc256zbq	W.Clegg, R.W.Harrington	Newcastle	C44 H50 P2 Rh1 1+,B1 F4 1-
2002704	CUPPIP	10.5517/ccdc.csd.cc256zcr	W.Clegg, R.W.Harrington	Newcastle	C76 H52 O2 P2 Pt1,2.5(C4 H8 O1)
2002705	CUPPOV	10.5517/ccdc.csd.cc256zds	W.Clegg, R.W.Harrington	Newcastle	C34 H25 P1
2002707	CUPPUB	10.5517/ccdc.csd.cc256zgv	W.Clegg, R.W.Harrington	Newcastle	C44 H40 Cl2 P2 Pt1,2(C1 H2 Cl2)
2002708	CUPQAI	10.5517/ccdc.csd.cc256zhw	W.Clegg, R.W.Harrington	Newcastle	C34 H37 P1,0.25(C1 H1 Cl3)
2002712	CUPQEM	10.5517/ccdc.csd.cc256zm0	W.Clegg, R.W.Harrington	Newcastle	C35 H39 O1 P1

2002713	CUPQIQ	10.5517/ccdc.csd.cc256zn1	W.Clegg, R.W.Harrington	Newcastle	C35 H27 O1 P1
2002714	CUPQOW	10.5517/ccdc.csd.cc256zp2	W.Clegg, R.W.Harrington	Newcastle	C13 H18 N1 O4 P1,0.5(C1 H2 Cl2)
2002715	CUPQUC	10.5517/ccdc.csd.cc256zq3	W.Clegg, R.W.Harrington	Newcastle	C36 H38 P2
2002716	CUPRAJ	10.5517/ccdc.csd.cc256zr4	W.Clegg, R.W.Harrington	Newcastle	C13 H18 N1 O4 P1,0.5(H2 O1)
2002717	CUPREN	10.5517/ccdc.csd.cc256zs5	W.Clegg, R.W.Harrington	Newcastle	C64 H52 P2 Rh1 1+,B1 F4 1-
2002725	CUPRIR	10.5517/ccdc.csd.cc2545xf	W.Clegg, R.W.Harrington	Newcastle	C58 H44 O4 P2,2(C1 H2 Cl2)
2002726	DBZACP13	10.5517/ccdc.csd.cc25701h	W.Clegg, R.W.Harrington	Newcastle	C51 H42 O3 Pd2,C1 H2 Cl2
2002727	CUPRUD	10.5517/ccdc.csd.cc25703k	W.Clegg, R.W.Harrington	Newcastle	C19 H26 N1 O1 P1
2002770	CUPZOF	10.5517/ccdc.csd.cc2571hz	W.Clegg, R.W.Harrington	Newcastle	C50 H44 O8 P2
2005398	RUQBUD	10.5517/ccdc.csd.cc259s8k	W.Clegg, R.W.Harrington	Newcastle	C26 H24 N2 O2
2005399	RUQCAK	10.5517/ccdc.csd.cc259s9l	W.Clegg, R.W.Harrington	Newcastle	C14 H16 N2 O2
2005400	RUQCEO	10.5517/ccdc.csd.cc259sbm	W.Clegg, R.W.Harrington	Newcastle	2(C24 H32 O8),C12 H14 N2 2+,2(F6 P1 1-)
2005401	RUQCIS	10.5517/ccdc.csd.cc259scn	W.Clegg, R.W.Harrington	Newcastle	C23 H28 I1 O2 1+,I1 1-
2005402	RUQCOY	10.5517/ccdc.csd.cc259sdp	W.Clegg, R.W.Harrington	Newcastle	C20 H22 I1 O5 1+,I1 1-
2005403	RAMPAX02	10.5517/ccdc.csd.cc259sfq	W.Clegg, R.W.Harrington	Newcastle	C12 H14 N2 2+,2(F6 P1 1-)
2005404	RUQDAL	10.5517/ccdc.csd.cc259sgr	W.Clegg, R.W.Harrington	Newcastle	C86 H58 N12 Ru2 2+,2(F6 P1 1-)
2005405	RUQDEP	10.5517/ccdc.csd.cc259shs	W.Clegg, R.W.Harrington	Newcastle	C33 H22 N1 1+,F6 P1 1-
2005406	ASOFOD05	10.5517/ccdc.csd.cc259sjt	W.Clegg, R.W.Harrington	Newcastle	C23 H22 N1 1+,Cl1 O4 1-
2005407	RUQDOZ	10.5517/ccdc.csd.cc259skv	W.Clegg, R.W.Harrington	Newcastle	C33 H18 N1 1+,F6 P1 1-
2005408	TPPOSS13	10.5517/ccdc.csd.cc259slw	W.Clegg, R.W.Harrington	Newcastle	C18 H15 P1 S1
2005409	RUQFAN	10.5517/ccdc.csd.cc259smx	W.Clegg, R.W.Harrington	Newcastle	C25 H18 O2
2005410	RUQFER	10.5517/ccdc.csd.cc259sny	W.Clegg, R.W.Harrington	Newcastle	C18 H18 I2 N2 O2 S2
2005411	RUQFIV	10.5517/ccdc.csd.cc259spz	W.Clegg, R.W.Harrington	Newcastle	C18 H18 I2 N2 O2 S2
2005412	RUQFOB	10.5517/ccdc.csd.cc259sq	W.Clegg, R.W.Harrington	Newcastle	C55 H34 N2 O3,C4 H8 O1
2005413	RUQFUH	10.5517/ccdc.csd.cc259sr1	W.Clegg, R.W.Harrington	Newcastle	C33 H21 Br1 N1 1+,C2 H3 N1,F6 P1 1-
2005414	RUQGAO	10.5517/ccdc.csd.cc259ss2	W.Clegg, R.W.Harrington	Newcastle	C26 H20 O2
2005415	RUQGES	10.5517/ccdc.csd.cc259st3	W.Clegg, R.W.Harrington	Newcastle	C41 H39 B1 F2 N2 O2
2005416	RUQGIW	10.5517/ccdc.csd.cc259sv4	W.Clegg, R.W.Harrington	Newcastle	C21 H21 B1 F2 N2 O2
2005417	RUQGOC	10.5517/ccdc.csd.cc259sw5	W.Clegg, R.W.Harrington	Newcastle	C35 H35 B1 F2 N2 O2
2005418	RUQGUI	10.5517/ccdc.csd.cc259sx6	W.Clegg, R.W.Harrington	Newcastle	C23 H27 B1 F2 N2 O2
2007766	JUKZAT	10.5517/ccdc.csd.cc25d7nh	W.Clegg, R.W.Harrington	Newcastle	C27 H25 B1 F2 N2 O2
2007767	TERYUN01	10.5517/ccdc.csd.cc25d7pj	W.Clegg, R.W.Harrington	Newcastle	C18 H13 B1 F2 N2 O1
2007768	JUKZIB	10.5517/ccdc.csd.cc25d7qk	W.Clegg, R.W.Harrington	Newcastle	C21 H20 B1 F2 N3
2007769	JUKZOH	10.5517/ccdc.csd.cc25d7rl	W.Clegg, R.W.Harrington	Newcastle	C21 H22 B1 Br1 F2 N2 O1 S1
2007770	YESBII01	10.5517/ccdc.csd.cc25d7sm	W.Clegg, R.W.Harrington	Newcastle	C15 H10 B1 F2 N3 O2
2007771	JULBAW	10.5517/ccdc.csd.cc25d7tn	W.Clegg, R.W.Harrington	Newcastle	C29 H36 B1 F2 N3
2007777	WALNEE02	10.5517/ccdc.csd.cc25d80w	W.Clegg, R.W.Harrington	Newcastle	C15 H17 N1 O1
2007778	JULBIE	10.5517/ccdc.csd.cc25d81x	W.Clegg, R.W.Harrington	Newcastle	C27 H28 B1 Br1 F2 N2
2007779	JULBOK	10.5517/ccdc.csd.cc25d82y	W.Clegg, R.W.Harrington	Newcastle	C33 H33 B1 N2 Si2
2007780	COLXAE01	10.5517/ccdc.csd.cc25d83z	W.Clegg, R.W.Harrington	Newcastle	C16 H14 B2 F4 N2 O2
2007781	JULCAX	10.5517/ccdc.csd.cc25d840	W.Clegg, R.W.Harrington	Newcastle	C34 H34 B1 F8 N5 O7 S2 Zn1,C2 H3 N1
2007782	JULCEB	10.5517/ccdc.csd.cc25d851	W.Clegg, R.W.Harrington	Newcastle	C20 H16 B1 F2 N2 O2 1+,B1 F4 1-

2007783	JULCIF	10.5517/ccdc.csd.cc25d862	W.Clegg, R.W.Harrington	Newcastle	C34 H31 B1 F2 N2 O1 S4
2007784	JULCOL	10.5517/ccdc.csd.cc25d873	W.Clegg, R.W.Harrington	Newcastle	C24 H29 B1 F2 N2 O1
2007785	HUFBAL01	10.5517/ccdc.csd.cc25d884	W.Clegg, R.W.Harrington	International	C16 H24 B2 N8 S4 Zn1
2010215	ZULLOK	10.5517/ccdc.csd.cc25gsn3	W.Clegg, M.R.J.Elsegood	Newcastle	C10 H21 N7 4+,Cl4 Pd1 2-,1.25(H2 O1),2(Cl1 1-)
2010216	ZULLUQ	10.5517/ccdc.csd.cc25gsp4	W.Clegg, M.R.J.Elsegood	Newcastle	C9 H16 Cl2 N7 O1 Rh1,6(H2 O1)
2010219	ZULSUX	10.5517/ccdc.csd.cc25gss7	W.Clegg, M.R.J.Elsegood	Newcastle	C15 H19 Fe1 N1 O3
2010221	ZULTAE	10.5517/ccdc.csd.cc25gsv9	W.Clegg, M.R.J.Elsegood	Newcastle	C8 H9 N5 O1,3(H2 O1)
2010223	ZULTEI	10.5517/ccdc.csd.cc25gsxc	W.Clegg, M.R.J.Elsegood	Newcastle	C14 H17 Fe1 N1 O3
2010224	ZULTIM	10.5517/ccdc.csd.cc25gsyd	W.Clegg, M.R.J.Elsegood	Newcastle	(C9 H17 Cl1 N5 O1 Zn1 1+)n,Cl1 O4 1-
2010225	ZULTOS	10.5517/ccdc.csd.cc25gszf	W.Clegg, M.R.J.Elsegood	Newcastle	(C36 H76 N8 O24 Y2 2+)n,8(H2 O1),2(Cl1 1-)
2010226	ZULTUY	10.5517/ccdc.csd.cc25gt0h	W.Clegg, M.R.J.Elsegood	Newcastle	C18 H38 Gd1 N8 O5 3+,C2 H6 O1,3(C1 F3 O3 S1 1-)
2010227	BULZUE01	10.5517/ccdc.csd.cc25gt1j	W.Clegg, M.R.J.Elsegood	Newcastle	(C10 H15 Cl1 Cu1 N5 S2)n
2010228	ZULVEK	10.5517/ccdc.csd.cc25gt2k	W.Clegg	Newcastle	C24 H46 Cl2 Cu2 N8 2+,2(C2 H6 O1),2(N1 O3 1-)
2010230	ZULVIO	10.5517/ccdc.csd.cc25gt4m	W.Clegg, P.A.Champkin	Newcastle	C18 H38 Eu1 N8 O5 3+,C2 H6 O1,3(C1 F3 O3 S1 1-)
2010233	ZULVOU	10.5517/ccdc.csd.cc25gt7q	W.Clegg, R.W.Harrington	Newcastle	C9 H15 Cl3 N7 Rh1,3(H2 O1)
2010234	ZULVUA	10.5517/ccdc.csd.cc25gt8r	W.Clegg	Newcastle	0.25(C3 H7 N1 O1),C1 F3 O3 S1 1-,0.25(H2 O1),K1 1+
2010235	ZULWAH	10.5517/ccdc.csd.cc25gt9s	W.Clegg	Newcastle	C16 H22 Cu1 N14 O6,2(C16 H24 Cu1 N13 O4 1+),2(H2 O1),2(Cl1 1-)
2010236	ZULWIP	10.5517/ccdc.csd.cc25gtbt	W.Clegg, R.W.Harrington	Newcastle	C9 H15 Cd1 Cl2 N7,H2 O1
2010238	ZULWOV	10.5517/ccdc.csd.cc25gtdw	W.Clegg, R.W.Harrington	Newcastle	C20 H42 Cu2 N18 O9 2+,5(N1 O3 1-),C20 H42 Cu2 N17 O6 3+
2012423	SUVGIC	10.5517/ccdc.csd.cc25k2wq	W.Clegg	Newcastle	C9 H17 N7 2+,F6 Si1 2-,3.5(H2 O1)
2012424	ZULVOU01	10.5517/ccdc.csd.cc25k2xr	W.Clegg, R.W.Harrington	Newcastle	C9 H15 Cl3 N7 Rh1,3(H2 O1)
2012425	SUVGUO	10.5517/ccdc.csd.cc25k2ys	W.Clegg, R.W.Harrington	Newcastle	C18 H25 N7 O2 S2
2012426	SUVHAV	10.5517/ccdc.csd.cc25k2zt	W.Clegg, R.W.Harrington	Newcastle	C17 H23 N7 O2 S2
2012427	SUVGUO01	10.5517/ccdc.csd.cc25k30w	W.Clegg	Newcastle	C18 H25 N7 O2 S2
2012428	SUVHID	10.5517/ccdc.csd.cc25k31x	W.Clegg, R.W.Harrington	Newcastle	C10 H15 N5 O1 S2
2012429	SUVHOJ	10.5517/ccdc.csd.cc25k32y	W.Clegg, R.W.Harrington	Newcastle	C21 H19 Cl2 Fe1 N3 Pd1
2012430	SUVHUP	10.5517/ccdc.csd.cc25k33z	W.Clegg, R.W.Harrington	Newcastle	C10 H16 N6 O2
2012431	SUVJAX	10.5517/ccdc.csd.cc25k340	W.Clegg, R.W.Harrington	Newcastle	C20 H30 Ag1 N10 S4 1+,C1 F3 O3 S1 1-
2012432	SUVJEB	10.5517/ccdc.csd.cc25k351	W.Clegg, R.W.Harrington	Newcastle	C18 H26 N4 O4 S2,2(H2 O1)
2014435	RAHGEQ	10.5517/ccdc.csd.cc25m5ss	W.Clegg, M.R.Probert, J.A.Gould, A.C.Benniston, I.Voda, C.Turta	Newcastle	(C23 H18 N4 Ni2 O7)n,H2 O1
2014436	RAHGIU	10.5517/ccdc.csd.cc25m5tt	W.Clegg, M.R.Probert, J.A.Gould, A.C.Benniston, I.Voda, C.Turta	Newcastle	(C38 H32 N12 Ni2 O8)n
2014437	UPUSUU04	10.5517/ccdc.csd.cc25m5vv	W.Clegg, M.R.Probert, J.A.Gould, A.C.Benniston, I.Voda, C.Turta	Newcastle	(C24 H18 N8 O8 Zn2)n
2014438	RAHGUG	10.5517/ccdc.csd.cc25m5ww	W.Clegg, M.R.Probert, J.A.Gould, A.C.Benniston, I.Voda, C.Turta	Newcastle	(C42 H34 N10 O8 Zn2)n,9.5(H2 O1)
2014439	DAVYIK01	10.5517/ccdc.csd.cc25m5xx	W.Clegg, M.R.Probert, J.A.Gould, A.C.Benniston, I.Voda, C.Turta	Newcastle	(C56 H48 Co2 N14 O8)n,8(H2 O1)
2016331	GUWNIY	10.5517/ccdc.csd.cc25p4yz	W.Clegg, R.W.Harrington	Newcastle	C10 H18 Cl2 N4 O2 Pd1,2(H2 O1)
2016332	GUWNOE	10.5517/ccdc.csd.cc25p4z0	W.Clegg, R.W.Harrington	Newcastle	C9 H18 Cu1 N8 O5 S1,2(H2 O1)
2016333	GUWNUK	10.5517/ccdc.csd.cc25p502	W.Clegg, R.W.Harrington	Newcastle	C18 H26 N4 O4 S2
2016334	GUWPAS	10.5517/ccdc.csd.cc25p513	W.Clegg, R.W.Harrington	Newcastle	C12 H15 N7,2(H2 O1)

2016335	GUWPEW	10.5517/ccdc.csd.cc25p524	W.Clegg, R.W.Harrington	Newcastle	(C9 H18 Cd1 Cl2 N8 O1)n,H2 O1
2016336	GUWPIA	10.5517/ccdc.csd.cc25p535	W.Clegg, R.W.Harrington	Newcastle	C21 H19 Cl2 Fe1 N3 Pt1
2016337	GUWPOG	10.5517/ccdc.csd.cc25p546	W.Clegg, R.W.Harrington	Newcastle	C10 H18 Cl2 N4 O2 Pt1,2(H2 O1)
2016338	GUWPUM	10.5517/ccdc.csd.cc25p557	W.Clegg, R.W.Harrington	Newcastle	C12 H15 N7
2016339	GUWQAT	10.5517/ccdc.csd.cc25p568	W.Clegg, R.W.Harrington	Newcastle	C10 H12 N2 O2
2016340	GUWQEX	10.5517/ccdc.csd.cc25p579	W.Clegg, R.W.Harrington	Newcastle	C12 H9 N1 S2
2016341	GUWQIB	10.5517/ccdc.csd.cc25p58b	W.Clegg, R.W.Harrington	Newcastle	C32 H24 N2 S4
2016342	GUWQOH	10.5517/ccdc.csd.cc25p59c	W.Clegg, R.W.Harrington	Newcastle	C6 H8 N2 O2
2016343	GUWQUN	10.5517/ccdc.csd.cc25p5bd	W.Clegg, R.W.Harrington	Newcastle	C8 H7 N1 S1
2016344	DIDPPD02	10.5517/ccdc.csd.cc25p5cf	W.Clegg	Newcastle	C36 H30 I2 P2 Pd1,2(C1 H2 Cl2)
2016345	GUWNIY01	10.5517/ccdc.csd.cc25p5dg	W.Clegg, R.W.Harrington	Newcastle	C10 H18 Cl2 N4 O2 Pd1,2(H2 O1)
2016346	GUWRIC	10.5517/ccdc.csd.cc25p5fh	W.Clegg, R.W.Harrington	Newcastle	C12 H15 N2 1+,F6 P1 1-
2017232	NUVQIH	10.5517/ccdc.csd.cc25q301	W.Clegg, R.W.Harrington	Newcastle	C20 H36 Ag2 N18 O6
2017233	NUVQON	10.5517/ccdc.csd.cc25q312	W.Clegg, R.W.Harrington	Newcastle	C12 H15 N3 O2
2017234	NUVQUT	10.5517/ccdc.csd.cc25q323	W.Clegg, R.W.Harrington	Newcastle	C16 H17 N7 S1,C3 H7 N1 O1
2017235	NUVRAA	10.5517/ccdc.csd.cc25q334	W.Clegg, R.W.Harrington	Newcastle	C20 H19 N7 S2
2017236	NUVREE	10.5517/ccdc.csd.cc25q345	W.Clegg, R.W.Harrington	Newcastle	C12 H15 N2 S1 1+,C12 H14 N2 S1,F6 P1 1-
2017237	NUVRII	10.5517/ccdc.csd.cc25q356	W.Clegg, R.W.Harrington	Newcastle	C18 H32 Br2 Cd1 N16,2(C9 H18 Cd1 Cl2 N8 O1)n,2(H2 O1)
2017239	GUWPUM01	10.5517/ccdc.csd.cc25q378	W.Clegg, R.W.Harrington	Newcastle	C12 H15 N7
2017240	NUVRUU	10.5517/ccdc.csd.cc25q389	W.Clegg, R.W.Harrington	Newcastle	C10 H18 Cl1 N8 Pt1 1+,H2 O1,Cl1 1-
2017241	NUVSAB	10.5517/ccdc.csd.cc25q39b	W.Clegg, R.W.Harrington	Newcastle	C12 H15 N7,C12 H15 N3 O2
2017242	NUVSEF	10.5517/ccdc.csd.cc25q3bc	W.Clegg, R.W.Harrington	Newcastle	C10 H19 N8 1+,Cl1 1-,C10 H18 N8
2018981	LUQQAS	10.5517/ccdc.csd.cc25rxf9	W.Clegg, D.C.R.Hockless	Newcastle	C1 H3 O15 W4 3-,3(C16 H36 N1 1+),H2 O1
2018983	LUQQEW	10.5517/ccdc.csd.cc25rxhc	W.Clegg	Newcastle	C2 H4 O2,Cl1 1-,C16 H36 N1 1+
2018986	SEFGIW01	10.5517/ccdc.csd.cc25rxlg	W.Clegg	Newcastle	C36 H30 N1 P2 1+,C1 H2 Cl2,H2 O1,Cl1 1-
2018987	LUQQOG	10.5517/ccdc.csd.cc25rxmh	W.Clegg, K.A.Fraser	Newcastle	2(C4 H12 N1 1+),O4 W1 2-,H2 O1
2018988	LUQQUM	10.5517/ccdc.csd.cc25rxnj	W.Clegg, K.A.Fraser	Newcastle	C4 H9 Mo6 N1 O18 2-,2(C16 H36 N1 1+),C2 H3 N1
2018995	LUQQOD01	10.5517/ccdc.csd.cc25rxwr	W.Clegg, K.A.Fraser	Newcastle	Mo8 O26 4-,4(C16 H36 N1 1+),4(C2 H3 N1)
2018996	BIBXAL07	10.5517/ccdc.csd.cc25rxxs	W.Clegg	Newcastle	Mo6 O19 2-,2(C16 H36 N1 1+)
2018997	LUQRIB	10.5517/ccdc.csd.cc25rxyt	W.Clegg, K.A.Fraser	Newcastle	C36 H30 N1 P2 1+,C5 H15 O6 W1 1-
2018998	LUQROH	10.5517/ccdc.csd.cc25rxzv	W.Clegg, K.A.Fraser	Newcastle	4(C16 H36 N1 1+),C2 H6 Mo8 O26 4-,2(C1 H4 O1),2(H2 O1)
2018999	LUQRUN	10.5517/ccdc.csd.cc25ry0x	W.Clegg, K.A.Fraser	Newcastle	2(C16 H36 N1 1+),2(C2 H4 Cl2),Cl4 O2 W1 2-
2019001	LUQSAU	10.5517/ccdc.csd.cc25ry2z	W.Clegg, K.A.Fraser	Newcastle	C6 H18 O16 W4 2-,2(C12 H28 N1 1+)
2019002	LUQSEY	10.5517/ccdc.csd.cc25ry30	W.Clegg, M.R.J.Elsegood	Newcastle	C20 H36 Mo2 O12 2-,2(C16 H36 N1 1+),2(C2 H3 N1)
2019003	LUQSIC	10.5517/ccdc.csd.cc25ry41	W.Clegg, M.R.J.Elsegood	Newcastle	2(C8 H20 N1 1+),C2 H3 N1,O4 W1 2-
2019004	XUBGUZ01	10.5517/ccdc.csd.cc25ry52	W.Clegg	Newcastle	C16 H36 N1 1+,0.5(C6 H6),Cl1 1-
2019005	LUQSUO	10.5517/ccdc.csd.cc25ry63	W.Clegg	Newcastle	C20 H28 O10 Ti2,2(C7 H8)
2019006	LUQTAV	10.5517/ccdc.csd.cc25ry74	W.Clegg, M.R.J.Elsegood	Newcastle	C26 H52 O14 Pb2,2(C1 H4 O1)
2021546	WUYGAB	10.5517/ccdc.csd.cc25vl5t	W.Clegg, M.R.J.Elsegood	Newcastle	C10 H36 Na2 O22 W4 2-,2(C10 H16 N1 1+),6(C1 H4 O1)
2021549	WUYGEF	10.5517/ccdc.csd.cc25vl8x	W.Clegg, K.A.Fraser	Newcastle	2(C16 H36 N1 1+),C10 H13 Mo6 N1 O18 2-
2021550	MEDXIC01	10.5517/ccdc.csd.cc25vl9y	W.Clegg, K.A.Fraser	Newcastle	C12 H17 Mo6 N1 O18 2-,2(C16 H36 N1 1+)
2021551	EFICUT03	10.5517/ccdc.csd.cc25vlbz	W.Clegg, K.A.Fraser	Newcastle	C10 H16 N1 1+,Cl1 1-

2021552	WUYSAN	10.5517/ccdc.csd.cc25vlc0	W.Clegg, K.A.Fraser	Newcastle	C6 H18 Mo4 O16 2-,2(C10 H16 N1 1+)
2021553	WUYSER	10.5517/ccdc.csd.cc25vld1	W.Clegg, M.R.J.Elsegood, L.Cucurull-Sanchez	Newcastle	C12 H34 N2 O12 W2
2021554	WUYSIV	10.5517/ccdc.csd.cc25vlf2	W.Clegg, L.Cucurull-Sanchez	Newcastle	(C4 H12 Na1 O5 V1)n
2021555	WUYSOB	10.5517/ccdc.csd.cc25vlg3	W.Clegg, M.R.J.Elsegood	Newcastle	2(C25 H22 P1 1+),Cl4 O1 V1 2-
2021557	WUYSUH	10.5517/ccdc.csd.cc25vlj5	W.Clegg, M.R.J.Elsegood	Newcastle	O34 V13 3-,3(C16 H36 N1 1+),0.5(C2 H3 N1)
2021558	TBUAWO06	10.5517/ccdc.csd.cc25vlk6	W.Clegg, M.R.J.Elsegood	Newcastle	2(C16 H36 N1 1+),O19 W6 2-
2021568	WUYTES	10.5517/ccdc.csd.cc25vlwj	W.Clegg, M.R.J.Elsegood	Newcastle	C12 H28 N1 1+,Cl1 1-
2021570	SEWBOM01	10.5517/ccdc.csd.cc25vlyl	W.Clegg, M.R.J.Elsegood	Newcastle	3(C16 H36 N1 1+),O19 W6 3-
2021573	BAMDMO01	10.5517/ccdc.csd.cc25vm1q	W.Clegg, R.L.Gill	Newcastle	(C6 H6 N2 O6 Zn1)n,n(H2 O1)
2021574	WUYTUI	10.5517/ccdc.csd.cc25vm2r	W.Clegg, M.R.J.Elsegood	Newcastle	C4 H10 Cl3 O3 V1
2021580	WUYVAQ	10.5517/ccdc.csd.cc25vm8y	W.Clegg, M.R.J.Elsegood	Newcastle	3(C16 H36 N1 1+),C2 H6 Nb3 O19 W3 3-,C2 H3 N1
2021582	WUYVEU	10.5517/ccdc.csd.cc25vmb0	W.Clegg, M.R.J.Elsegood	Newcastle	C26 H56 O12 Ti2
2021583	WUYVIY	10.5517/ccdc.csd.cc25vmc1	W.Clegg, M.R.J.Elsegood	Newcastle	(C20 H28 Cu1 N2 O8)n
2021592	WUYZUO	10.5517/ccdc.csd.cc25vmnb	W.Clegg, M.R.J.Elsegood	Newcastle	2(C16 H36 N1 1+),C10 H14 Mo6 N2 O18 2-,C4 H10 O1,C2 H3 N1
2026088	YABPAW	10.5517/ccdc.csd.cc2609p7	W.Clegg, D.C.R.Hockless	Newcastle	C23 H29 Cl3 N1 O1 P2 W1,0.5(C7 H8)
2026089	YABHUI	10.5517/ccdc.csd.cc2609q8	W.Clegg, D.C.R.Hockless	Newcastle	4(C16 H36 N1 1+),O32 W10 4-,4(C6 H6 O2)
2026090	YABJAQ	10.5517/ccdc.csd.cc2609r9	W.Clegg, D.C.R.Hockless	Newcastle	C4 H12 N1 1+,C16 H36 Cl5 Mo2 N4 1-,0.5(C7 H8)
2026091	YABBAI	10.5517/ccdc.csd.cc2609sb	W.Clegg, M.R.J.Elsegood	Newcastle	2(C36 H30 N1 P2 1+),C10 H15 Mo6 N1 O18 2-
2026092	YABBEM	10.5517/ccdc.csd.cc2609tc	W.Clegg, S.L.Heath	Newcastle	3(C16 H36 N1 1+),O14 V5 3-,C4 H10 O1,2(C2 H3 N1)
2026093	YUZTAR	10.5517/ccdc.csd.cc2609vd	W.Clegg, S.L.Heath	Newcastle	C16 H36 N1 1+,C6 H6 O3 P1 1-,C4 H8 O1
2026094	YUZTEV	10.5517/ccdc.csd.cc2609wf	W.Clegg, S.L.Heath	Newcastle	2(C16 H36 N1 1+),C12 H10 O10 P2 V2 2-
2026095	YUZTIZ	10.5517/ccdc.csd.cc2609xg	W.Clegg, S.L.Heath	Newcastle	4(Na1 1+),3(C4 H10 N1 S1 1+),H4 O40 W12 3-,H3 O40 W12 4-,9(C4 H9 N1 S1),4(H2 O1)
2026096	YUZTOF	10.5517/ccdc.csd.cc2609yh	W.Clegg, S.L.Heath	Newcastle	C10 H16 N1 1+,C2 H6 O4 V1 1-
2026097	BIBXAL08	10.5517/ccdc.csd.cc2609zj	W.Clegg, R.A.Coxall	Newcastle	2(C16 H36 N1 1+),Mo6 O19 2-
2026098	YABBOW	10.5517/ccdc.csd.cc260b0l	W.Clegg, S.L.Heath	Newcastle	C108 H118 B2 O20 Sr4,3(C4 H8 O1)
2026099	RERLOP01	10.5517/ccdc.csd.cc260b1m	W.Clegg, R.A.Coxall	Newcastle	C21 H15 O6 Sb1
2026100	NOCYOT01	10.5517/ccdc.csd.cc260b2n	W.Clegg, S.L.Heath	Newcastle	C40 H84 O16 Ti4
2026101	ARAPAK01	10.5517/ccdc.csd.cc260b3p	W.Clegg, R.A.Coxall	Newcastle	C18 H18 Mn1 O16,2(H2 O1)
2026102	YABPEA	10.5517/ccdc.csd.cc260b4q	W.Clegg, A.J.Edwards	Newcastle	2(C16 H36 N1 1+),C24 H20 O31 P4 V9 1-,Cl1 1-
2026103	YABPIE	10.5517/ccdc.csd.cc260b5r	W.Clegg, R.A.Coxall	Newcastle	C17 H27 N1 O7 V1
2026104	OXACTI02	10.5517/ccdc.csd.cc260b6s	W.Clegg, A.J.Edwards	Newcastle	C20 H28 O10 Ti2
2026105	HUQKEM	10.5517/ccdc.csd.cc260b7t	W.Clegg, A.J.Edwards	Newcastle	3(C16 H36 N1 1+),C30 H25 O26 P5 V6 3-,0.5(C2 H3 N1),0.5(C4 H10 O1)
2026106	HUQKIQ	10.5517/ccdc.csd.cc260b8v	W.Clegg, A.J.Edwards	Newcastle	2(C16 H36 N1 1+),C6 H6 Mo6 N2 O18 2-,0.33(C4 H10 O1)
2026107	HUQKOW	10.5517/ccdc.csd.cc260b9w	W.Clegg, R.A.Coxall	Newcastle	C34 H78 Li2 O12 Ti2
2030856	DUTGAD	10.5517/ccdc.csd.cc2658h5	W.Clegg, R.A.Coxall	Newcastle	C24 H48 O10 Ti2
2030858	DUTGEH	10.5517/ccdc.csd.cc2658k7	W.Clegg, A.J.Edwards	Newcastle	3(C16 H36 N1 1+),C6 H5 O11 P1 V3 3-,H2 O1
2030859	DUTGIL	10.5517/ccdc.csd.cc2658l8	W.Clegg, A.J.Edwards	Newcastle	(C9 H25 Na1 O10 V2)n
2030860	DUTGOR	10.5517/ccdc.csd.cc2658m9	W.Clegg, R.A.Coxall	Newcastle	C18 H28 O13 P2 Ta2 2-,2(C16 H36 N1 1+)
2030862	DUTGUX	10.5517/ccdc.csd.cc2658pc	W.Clegg, R.A.Coxall	Newcastle	C26 H44 O12 P2 Ti2 2-,2(C16 H36 N1 1+),C2 H3 N1
2030863	BAMVAN01	10.5517/ccdc.csd.cc2658qd	W.Clegg, R.W.Harrington	Newcastle	2(C16 H36 N1 1+),Mo2 O7 2-
2030864	DUTHEI	10.5517/ccdc.csd.cc2658rf	W.Clegg, R.A.Coxall	Newcastle	C18 H44 Cl4 O6 Zr2
2030865	DUTHIM	10.5517/ccdc.csd.cc2658sg	W.Clegg, R.A.Coxall	Newcastle	C16 H28 O6 Ti1

2030867	ACAMBR03	10.5517/ccdc.csd.cc2658vj	W.Clegg, R.A.Coxall	Newcastle	C2 H6 N1 O1 1+,Br1 1-,C2 H5 N1 O1
2030868	XONDIO01	10.5517/ccdc.csd.cc2658wk	W.Clegg, R.A.Coxall	Newcastle	C12 H28 Cl4 O4 Ti2
2030869	NAYDAS01	10.5517/ccdc.csd.cc2658xl	W.Clegg, R.A.Coxall	Newcastle	C30 H72 Hf2 O10
2030870	DUTJEK	10.5517/ccdc.csd.cc2658ym	W.Clegg, R.A.Coxall	Newcastle	2(C16 H36 N1 1+),C8 H24 O12 P2 Ti2 2-
2030872	DUTJIO	10.5517/ccdc.csd.cc26590q	W.Clegg, R.A.Coxall	Newcastle	C16 H40 O12 P2 Ti2 2-,2(C16 H36 N1 1+)
2030873	DUTJOU	10.5517/ccdc.csd.cc26591r	W.Clegg, R.A.Coxall	Newcastle	C24 H21 N3 O3,2(C2 H3 N1)
2030874	TAQCAR01	10.5517/ccdc.csd.cc26592s	W.Clegg, R.A.Coxall	Newcastle	Mo12 O42 P1 V2 3-,C2 H3 N1,3(C16 H36 N1 1+)
2030876	REVLUA01	10.5517/ccdc.csd.cc26594v	W.Clegg, R.A.Coxall	Newcastle	C21 H15 N3 O3,2(C1 H2 Cl2)
2030877	DUTKEL	10.5517/ccdc.csd.cc26595w	W.Clegg, R.A.Coxall	Newcastle	C6 H5 Hf1 O19 W5 3-,3(C16 H36 N1 1+)
2030879	DUTKIP	10.5517/ccdc.csd.cc26597y	W.Clegg, R.A.Coxall	Newcastle	C54 H124 O30 P4 Ti6
2030880	GATQOI01	10.5517/ccdc.csd.cc26598z	W.Clegg, R.A.Coxall	Newcastle	C38 H66 N2 O10 Ti2
2030881	DUTKUB	10.5517/ccdc.csd.cc265990	W.Clegg, R.A.Coxall	Newcastle	C9 H18 N1 O1 1+,0.25(H2 O1),Cl1 1-
2030883	DUTLEM	10.5517/ccdc.csd.cc2659c2	W.Clegg, R.A.Coxall	Newcastle	C16 H36 N1 1+,C2 H3 Cl3 N1 Pt1 1-
2030884	TOVYUY01	10.5517/ccdc.csd.cc2659d3	W.Clegg, R.A.Coxall	Newcastle	C18 H44 Cl4 O6 Ti2
2030885	DUTLOW	10.5517/ccdc.csd.cc2659f4	W.Clegg, R.A.Coxall	Newcastle	C66 H54 Cl2 N2 O5 Ti2
2030887	DUTLUC	10.5517/ccdc.csd.cc2659h6	W.Clegg, R.A.Coxall	Newcastle	C40 H48 N4 O8 Ti1
2031460	QICHOZ01	10.5517/ccdc.csd.cc265wz8	W.Clegg, R.A.Coxall, M.A.Echevarria	Newcastle	C40 H90 Bi2 O8
2031462	DUZZIK	10.5517/ccdc.csd.cc265x1c	W.Clegg, M.R.J.Elsegood, M.A.Echevarria	Newcastle	(C12 H26 O4 Pb1)n
2031463	DUZZOQ	10.5517/ccdc.csd.cc265x2d	W.Clegg, R.A.Coxall	Newcastle	C30 H38 O10 P2 Ti2
2031473	DUZZUW	10.5517/ccdc.csd.cc265xdq	W.Clegg, M.R.J.Elsegood, M.A.Echevarria	Newcastle	C24 H20 B1 O4 1-,4(C2 H3 N1),H4 N1 1+
2031474	DABBAN	10.5517/ccdc.csd.cc265xfr	W.Clegg, R.A.Coxall	Newcastle	C30 H36 O10 Ti1
2031478	DABMUS	10.5517/ccdc.csd.cc265xkw	W.Clegg, R.A.Coxall	Newcastle	C36 H56 Nb4 O24 P4
2031480	DABNAZ	10.5517/ccdc.csd.cc265xmy	W.Clegg, M.R.J.Elsegood, M.A.Echevarria	Newcastle	C24 H34 Ba1 O8,2(C6 H6 O1),2(C4 H8 O1)
2031481	DABNED	10.5517/ccdc.csd.cc265xnz	W.Clegg, M.R.J.Elsegood, M.A.Echevarria	Newcastle	C56 H56 B2 Ca1 O10
2031483	DABNIH	10.5517/ccdc.csd.cc265xq1	W.Clegg, R.A.Coxall	Newcastle	C30 H38 Nb2 O11 P2
2031484	DABNON	10.5517/ccdc.csd.cc265xr2	W.Clegg, M.A.Echevarria	Newcastle	C32 H56 O16 Pb4
2031485	DABNUT	10.5517/ccdc.csd.cc265xs3	W.Clegg, M.R.J.Elsegood	Newcastle	C38 H54 O10 P2 Ti2
2031486	DABPAB	10.5517/ccdc.csd.cc265xt4	W.Clegg, M.R.J.Elsegood	Newcastle	C56 H60 B2 O12 Sr1
2031487	DABPEF	10.5517/ccdc.csd.cc265xv5	W.Clegg, M.R.J.Elsegood	Newcastle	C20 H44 Ba1 O9 2+,2(C24 H20 B1 O4 1-)
2031488	FISJOI01	10.5517/ccdc.csd.cc265xw6	W.Clegg, M.R.J.Elsegood	Newcastle	C48 H96 O28 Ti6
2031490	DABPOP	10.5517/ccdc.csd.cc265xy8	W.Clegg, M.A.Echevarria	Newcastle	C16 H36 N1 1+,C12 H10 O2 P1 1-,2(C1 H4 O1)
2031491	DABPUV	10.5517/ccdc.csd.cc265xz9	W.Clegg, M.R.J.Elsegood, M.A.Echevarria	Newcastle	C60 H68 O16 P4 Ti4,C4 H8 O1
2031492	DABQAC	10.5517/ccdc.csd.cc265y0c	W.Clegg, M.R.J.Elsegood	Newcastle	C20 H44 O10 Sr1 2+,2(C24 H20 B1 O4 1-)
2031493	DABQEG	10.5517/ccdc.csd.cc265y1d	W.Clegg, M.R.J.Elsegood	Newcastle	C68 H70 B2 Mg2 O12,2(C4 H10 O1)
2034638	JUXQOL	10.5517/ccdc.csd.cc2696h7	W.Clegg, M.R.J.Elsegood, M.A.Echevarria	Newcastle	C20 H44 Ge1 O4
2034639	JUXQUR	10.5517/ccdc.csd.cc2696j8	W.Clegg, M.R.J.Elsegood, M.A.Echevarria	Newcastle	C24 H40 O12 Pb4,2(C7 H8)
2034641	XANFOH01	10.5517/ccdc.csd.cc2696lb	W.Clegg, M.R.J.Elsegood, M.A.Echevarria	Newcastle	C46 H79 O19 P3 Ti4,0.5(C4 H8 O1)
2034642	JUXREC	10.5517/ccdc.csd.cc2696mc	W.Clegg, M.R.J.Elsegood,	Newcastle	C48 H76 O13 P2 Ti3

			M.A.Echevarria		
2034643	JUXROM	10.5517/ccdc.csd.cc2696nd	W.Clegg, M.R.J.Elsegood	Newcastle	C16 H36 O8 Sr1 2+,2(C24 H20 B1 O4 1-)
2034644	JUXRUS	10.5517/ccdc.csd.cc2696pf	W.Clegg, M.R.J.Elsegood	Newcastle	C54 H54 B2 Mg1 O11,C7 H8
2034645	DUTGUX01	10.5517/ccdc.csd.cc2696qg	W.Clegg, M.R.J.Elsegood, M.A.Echevarria	Newcastle	C26 H44 O12 P2 Ti2 2-,2(C16 H36 N1 1+),C2 H3 N1
2034650	BIBDEV01	10.5517/ccdc.csd.cc2696wm	W.Clegg, M.R.J.Elsegood, M.A.Echevarria	Newcastle	C12 H28 O8 Pb6
2034651	JUXVUW	10.5517/ccdc.csd.cc2696xn	W.Clegg, M.R.J.Elsegood, M.A.Echevarria	Newcastle	C30 H38 Nb2 O11 P2
2034652	JUXWAD	10.5517/ccdc.csd.cc2696yp	W.Clegg, M.R.J.Elsegood, M.A.Echevarria	Newcastle	C30 H38 O11 P2 Ta2
2034653	JUXWEH	10.5517/ccdc.csd.cc2696zq	W.Clegg, M.R.J.Elsegood	Newcastle	C20 H24 Al2 N2 O4
2034654	JUXWIL	10.5517/ccdc.csd.cc26970s	W.Clegg, M.R.J.Elsegood	Newcastle	C44 H100 O24 P4 Ti4
2034655	JUXWOR	10.5517/ccdc.csd.cc26971t	W.Clegg, M.R.J.Elsegood	Newcastle	C20 H24 Al1 N2 O4 1+,2(C4 H8 O1),Cl1 1-
2034656	JUXWUX	10.5517/ccdc.csd.cc26972v	W.Clegg, M.R.J.Elsegood, N.Housley	Newcastle	C18 H23 Cl2 N1 O3 Ti1
2034657	FOJKEW01	10.5517/ccdc.csd.cc26973w	W.Clegg, M.R.J.Elsegood, N.Housley	Newcastle	C30 H36 Al2 N2 O4
2034658	JUXXOS	10.5517/ccdc.csd.cc26974x	W.Clegg, N.Housley	Newcastle	C34 H42 O10 P2 Ti2
2034659	JUXXUY	10.5517/ccdc.csd.cc26975y	W.Clegg, N.Housley	Newcastle	C28 H30 N2 O4 Ti1,0.5(C4 H8 O1)
2034660	JUXYAF	10.5517/ccdc.csd.cc26976z	W.Clegg, P.A.Champkin	Newcastle	C44 H64 O8 Ti2
2034661	JUXYEJ	10.5517/ccdc.csd.cc269770	W.Clegg, N.Housley	Newcastle	C20 H36 Al2 N2 O6
2034663	JUXYIN	10.5517/ccdc.csd.cc269792	W.Clegg	Newcastle	C42 H42 O10 P2 Ti2
2034664	JUXYOT	10.5517/ccdc.csd.cc2697b3	W.Clegg, R.W.Harrington	Newcastle	C24 H45 Cl2 O3 P2 Ru1,C1 H2 Cl2
2034665	JUXYUZ	10.5517/ccdc.csd.cc2697c4	W.Clegg, R.W.Harrington	Newcastle	H2 Mo12 O48 Ti4 6-,6(C16 H36 N1 1+),2(C5 H5 N1 O1),2(C2 H3 N1)
2039431	ZADPON	10.5517/ccdc.csd.cc26g630	W.Clegg, R.W.Harrington	Newcastle	C16 H36 N1 1+,H3 O4 P1,H2 O4 P1 1-
2039433	GATQOI02	10.5517/ccdc.csd.cc26g652	W.Clegg, R.W.Harrington	Newcastle	C38 H66 N2 O10 Ti2
2039434	ZADPUT	10.5517/ccdc.csd.cc26g663	W.Clegg, R.W.Harrington	Newcastle	C20 H44 Cl2 O4 Sb2
2039436	ZADQAA	10.5517/ccdc.csd.cc26g685	W.Clegg, R.W.Harrington	Newcastle	C38 H82 Li2 O12 Ti2
2039438	ZADQEE	10.5517/ccdc.csd.cc26g6b7	W.Clegg, R.W.Harrington	Newcastle	Mo12 O40 P1 3-,3(C16 H36 N1 1+)
2039439	ZADQII	10.5517/ccdc.csd.cc26g6c8	W.Clegg, R.W.Harrington	Newcastle	C18 H42 Cl2 Li2 O12 Sb2
2039440	ZADQOO	10.5517/ccdc.csd.cc26g6d9	W.Clegg, R.W.Harrington	Newcastle	C24 H44 Cl3 Mo1 O3 P2,2(C2 H3 N1)
2039441	ZADQUU	10.5517/ccdc.csd.cc26g6fb	W.Clegg, R.W.Harrington	Newcastle	C48 H88 Cl6 O5 P4 Ti2,C1 H2 Cl2
2039442	ZADRAB	10.5517/ccdc.csd.cc26g6gc	W.Clegg, R.W.Harrington	Newcastle	C24 H44 Al1 Cl2 O2 P2 1+,2(C1 H2 Cl2),Cl1 1-
2039443	ZADREF	10.5517/ccdc.csd.cc26g6hd	W.Clegg, R.W.Harrington	Newcastle	C24 H44 Cl3 Mo1 O3 P2,1.5(C1 H2 Cl2)
2039444	ZADRIJ	10.5517/ccdc.csd.cc26g6jf	W.Clegg, R.W.Harrington	Newcastle	C26 H62 Cl2 O10 Ti2 Zn2
2039445	ZADROP	10.5517/ccdc.csd.cc26g6kg	W.Clegg, R.W.Harrington	Newcastle	C24 H44 Cl2 O2 P2 Zn1
2040422	SAFVUU	10.5517/ccdc.csd.cc26h721	W.Clegg, R.W.Harrington	Newcastle	C32 H76 Cl2 Cu2 Li2 O14 Ti2
2040423	SAFWAB	10.5517/ccdc.csd.cc26h732	W.Clegg, R.W.Harrington	Newcastle	C34 H82 Mg1 O14 Ti3
2040424	IPRXAL15	10.5517/ccdc.csd.cc26h743	W.Clegg, R.W.Harrington	Newcastle	C36 H84 Al4 O12
2040425	SAFWIJ	10.5517/ccdc.csd.cc26h754	W.Clegg, R.W.Harrington	Newcastle	C15 H10 Mo5 N3 O19 Ti1 3-,3(C16 H36 N1 1+)
2040426	TBUAWO07	10.5517/ccdc.csd.cc26h765	W.Clegg, R.W.Harrington	Newcastle	2(C16 H36 N1 1+),O19 W6 2-
2040427	SAFWUV	10.5517/ccdc.csd.cc26h776	W.Clegg, R.W.Harrington	Newcastle	C15 H10 N3 O19 W5 Zr1 3-,3(C16 H36 N1 1+)
2040433	SAFXAC	10.5517/ccdc.csd.cc26h7fd	W.Clegg, R.W.Harrington	Newcastle	C15 H10 N3 O19 Ti1 W5 3-,3(C16 H36 N1 1+)
2040434	SAFXEG	10.5517/ccdc.csd.cc26h7gf	W.Clegg, R.W.Harrington	Newcastle	C15 H33 O3 Sb1
2040481	SAGFUF	10.5517/ccdc.csd.cc26h8zz	W.Clegg, R.W.Harrington	Newcastle	C26 H62 Cl2 Co2 O10 Ti2

2042265	BAMDMO01	10.5517/ccdc.csd.cc26k4jg	W.Clegg, M.R.J.Elsegood	Newcastle	2(C16 H36 N1 1+),Mo2 O7 2-
2042266	TBUAWO08	10.5517/ccdc.csd.cc26k4kh	W.Clegg, R.W.Harrington	Newcastle	2(C16 H36 N1 1+),O19 W6 2-
2042267	NAYZAO01	10.5517/ccdc.csd.cc26k4lj	W.Clegg, R.W.Harrington	Newcastle	C40 H88 O8 Ti2
2042268	TAGCIR	10.5517/ccdc.csd.cc26k4mk	W.Clegg, R.W.Harrington	Newcastle	C46 H102 O11 Ti3,C7 H8
2042274	TAGCOX	10.5517/ccdc.csd.cc26k4tr	W.Clegg, R.W.Harrington	Newcastle	C46 H102 O11 Ti3
2042278	TAGCUD	10.5517/ccdc.csd.cc26k4yw	W.Clegg, R.W.Harrington	Newcastle	C27 H64 Al2 Cl4 O10 Ti2
2042279	TAGCUD01	10.5517/ccdc.csd.cc26k4zx	W.Clegg, R.W.Harrington	Newcastle	C27 H64 Al2 Cl4 O10 Ti2
2042280	TAGDEO	10.5517/ccdc.csd.cc26k50z	W.Clegg, R.W.Harrington	Newcastle	C34 H78 Li2 O12 Zr2
2042281	TAGDIS	10.5517/ccdc.csd.cc26k510	W.Clegg, R.W.Harrington	Newcastle	C26 H62 Cl2 O10 Zn2 Zr2
2042282	BIBXAL09	10.5517/ccdc.csd.cc26k521	W.Clegg, R.W.Harrington	Newcastle	2(C16 H36 N1 1+),Mo6 O19 2-
2042283	TAGDUE	10.5517/ccdc.csd.cc26k532	W.Clegg, R.W.Harrington	Newcastle	(C27 H65 Cl4 Co3 Li1 O9 Zr1)n
2042285	TAGFAM	10.5517/ccdc.csd.cc26k554	W.Clegg, R.W.Harrington	Newcastle	Mo6 O19 3-,3(C10 H10 Fe1 1+)
2042286	TAGFEQ	10.5517/ccdc.csd.cc26k565	W.Clegg, R.W.Harrington	Newcastle	C34 H78 Hf2 Li2 O12
2046405	WAGYUC	10.5517/ccdc.csd.cc26pg2g	W.Clegg, R.W.Harrington	Newcastle	C18 H42 Cl6 Co3 O6 Zr1 2-,2(C16 H32 Li1 O4 1+)
2046406	WAGZAJ	10.5517/ccdc.csd.cc26pg3h	W.Clegg, R.W.Harrington	Newcastle	C36 H88 O16 Ti4
2046412	GEMCOS01	10.5517/ccdc.csd.cc26pg9p	W.Clegg, R.W.Harrington	Newcastle	C8 H16 Co1 N6,C1 H4 O1
2046414	WAHDIW	10.5517/ccdc.csd.cc26pgcr	W.Clegg, R.W.Harrington	Newcastle	C24 H20 O40 P2 W10 Zr2 6-,6(C16 H36 N1 1+),4(C2 H3 N1)
2046418	WAHXAI	10.5517/ccdc.csd.cc26pghw	W.Clegg, R.W.Harrington	Newcastle	C12 H24 Co1 N9
2046419	WAHXEM	10.5517/ccdc.csd.cc26pgjx	W.Clegg, R.W.Harrington	Newcastle	C16 H36 N1 1+,C8 H16 Co1 N6,Cl1 1-
2046420	WAHXIQ	10.5517/ccdc.csd.cc26pgky	W.Clegg, R.W.Harrington	Newcastle	C8 H16 Co1 N4 O2 2+,2(B1 F4 1-)
2046434	LUQQAS01	10.5517/ccdc.csd.cc26ph0f	W.Clegg, R.W.Harrington	Newcastle	3(C16 H36 N1 1+),C1 H3 O15 W4 3-,H2 O1
2046449	WAHXUC	10.5517/ccdc.csd.cc26phhx	W.Clegg, R.W.Harrington	Newcastle	C2 H7 Mo12 N1 Na2 O44 P1 V2 4-,4(C16 H36 N1 1+)
2046451	WAHYAJ	10.5517/ccdc.csd.cc26phkz	W.Clegg, R.W.Harrington	Newcastle	Cl2 Fe2 Mo12 O40 P1 4-,4(C16 H36 N1 1+)
2046456	LUQQOD02	10.5517/ccdc.csd.cc26phq4	W.Clegg, R.W.Harrington	Newcastle	4(C16 H36 N1 1+),Mo8 O26 4-,4(C2 H3 N1)
2046457	WAHYIR	10.5517/ccdc.csd.cc26phr5	W.Clegg, R.W.Harrington	Newcastle	7.5(C16 H36 N1 1+),O37 Ti2 W10 6-,0.5(H1 O19 Ti1 W5 3-),C4 H8 O1
2051665	EJIRUN	10.5517/ccdc.csd.cc26vxrr	W.Clegg, R.W.Harrington	Newcastle	C4 H6 Mo12 N2 O40 P1 Zn1 3-,3(C16 H36 N1 1+),C2 H3 N1
2051666	EJITID	10.5517/ccdc.csd.cc26vxss	W.Clegg, R.W.Harrington	Newcastle	6(C16 H36 N1 1+),H2 Co2 O36 W10 6-
2051667	EJITOJ	10.5517/ccdc.csd.cc26vxtt	W.Clegg, R.W.Harrington	Newcastle	12(C16 H36 N1 1+),O20 Ti1 W5 3-,3(H1 O19 Ti1 W5 3-)
2051668	LUQQOD03	10.5517/ccdc.csd.cc26vxvv	W.Clegg, R.W.Harrington	Newcastle	4(C16 H36 N1 1+),Mo8 O26 4-,4(C2 H3 N1)
2051671	EJIVIF	10.5517/ccdc.csd.cc26vxyy	W.Clegg, R.W.Harrington	Newcastle	C9 H27 O9 Sn2 1-,C10 H16 N1 1+
2051675	NAQMUN02	10.5517/ccdc.csd.cc26vy23	W.Clegg, R.W.Harrington	Newcastle	C8 H12 Cu1 N4 1+,B1 F4 1-
2051677	EJOCOY	10.5517/ccdc.csd.cc26vy45	W.Clegg, R.W.Harrington	Newcastle	C14 H34 Cl8 O8 P2 Ti2
2051687	JUXYAF01	10.5517/ccdc.csd.cc26vygh	W.Clegg, R.W.Harrington	Newcastle	C44 H64 O8 Ti2
2051691	FENVUQ04	10.5517/ccdc.csd.cc26vylm	W.Clegg, R.W.Harrington	Newcastle	C16 H36 N1 1+,Cl4 Fe1 1-
2051695	EJODEP	10.5517/ccdc.csd.cc26vyqr	W.Clegg, R.W.Harrington	Newcastle	C48 H60 O10 Ti2
2051717	EJODIT	10.5517/ccdc.csd.cc26vzfh	W.Clegg, R.W.Harrington	Newcastle	C16 H36 N1 1+,Cl1 1-,0.5(C2 H3 N1)
2051787	EJUTUB	10.5517/ccdc.csd.cc26w1pv	W.Clegg, R.W.Harrington	Newcastle	C16 H36 N1 1+,Cl1 1-,C2 H5 N1 O1
2051788	EJUVAJ	10.5517/ccdc.csd.cc26w1qw	W.Clegg, R.W.Harrington	Newcastle	C27 H64 O10 Sb2
2051789	EJUVEN	10.5517/ccdc.csd.cc26w1rx	W.Clegg, R.W.Harrington	Newcastle	C15 H38 N1 O5 Sb1
2054117	PEBTIB01	10.5517/ccdc.csd.cc26ygvh	W.Clegg, P.N.O'Shaughnessy	Newcastle	C19 H38 Li1 N3 Si1
2054118	IKEPIA	10.5517/ccdc.csd.cc26ygwj	W.Clegg, P.N.O'Shaughnessy	Newcastle	C28 H56 O7 Sm1 2+,2(C24 H20 B1 1-),C4 H8 O1
2054121	IKEPOG	10.5517/ccdc.csd.cc26ygzm	W.Clegg, P.N.O'Shaughnessy	Newcastle	C13 H33 O1 Si3 1-,K1 1+

2054125	IKEPUM	10.5517/ccdc.csd.cc26yh3s	W.Clegg, P.N.O'Shaughnessy	Newcastle	C25 H47 N2 P1 Si4
2054127	IKEQAT	10.5517/ccdc.csd.cc26yh5v	W.Clegg, P.N.O'Shaughnessy	Newcastle	C28 H52 Al1 N2 P1 Si2
2054131	IKEQEX	10.5517/ccdc.csd.cc26yh9z	W.Clegg, P.N.O'Shaughnessy	Newcastle	C64 H76 Li2 N4 P4 S4,3.5(C7 H8)
2054137	IKEQIB	10.5517/ccdc.csd.cc26yhh5	W.Clegg, P.N.O'Shaughnessy	Newcastle	C41 H71 N3 P2 Si4 Yb1,C4 H8 O1
2054138	IKEQOH	10.5517/ccdc.csd.cc26yhj6	W.Clegg, P.N.O'Shaughnessy	Newcastle	C33 H42 Cl2 Ni1 P2 Si2,C4 H10 O1
2054139	IKEQUN	10.5517/ccdc.csd.cc26yhk7	W.Clegg, S.T.Liddle	Newcastle	C38 H74 N2 O2 P2 Si4 Yb1
2054140	IKERAU	10.5517/ccdc.csd.cc26yhl8	W.Clegg	Newcastle	C21 H33 O2 P1 Si2
2054308	IKOQUX	10.5517/ccdc.csd.cc26yp0w	W.Clegg	Newcastle	C52 H46 P4
2054309	IKORAE	10.5517/ccdc.csd.cc26yp1x	W.Clegg, R.W.Harrington	Newcastle	C52 H46 P4 S4,2(C1 H1 Cl3)
2054310	IKOREI	10.5517/ccdc.csd.cc26yp2y	W.Clegg, S.T.Liddle	Newcastle	C27 H35 N2 P1
2054311	IKORIM	10.5517/ccdc.csd.cc26yp3z	W.Clegg, R.W.Harrington	Newcastle	C18 H48 I4 O2 Si6 Tm2 2-,2(C16 H32 Li1 O4 1+)
2054663	OJIMUS	10.5517/ccdc.csd.cc26z1gq	W.Clegg, R.W.Harrington	Newcastle	C52 H84 N4 Na2 P4 S4
2054678	CESJUF02	10.5517/ccdc.csd.cc26z1y6	W.Clegg, R.W.Harrington	Newcastle	C8 H20 Br1 Li1 O4
2054728	OJISOS	10.5517/ccdc.csd.cc26z3kw	W.Clegg	Newcastle	0.625(C23 H31 Cl1 P2),0.375(C23 H31 Br1 P2)
2054729	HUXPOF01	10.5517/ccdc.csd.cc26z3lx	W.Clegg	Newcastle	C35 H55 Li1 O3 P2
2054783	OJITUZ	10.5517/ccdc.csd.cc26z5bq	W.Clegg, S.T.Liddle	Newcastle	C80 H172 Li8 N4 Si12
2054784	OJIVAH	10.5517/ccdc.csd.cc26z5cr	W.Clegg, R.W.Harrington	Newcastle	C36 H90 Nd2 O6 Si6
2055793	OLAFEP	10.5517/ccdc.csd.cc2706xd	W.Clegg, R.W.Harrington	Newcastle	C23 H56 B2 F3 I1 P2 Si2 Sn1
2055795	OLAFIT	10.5517/ccdc.csd.cc2706zg	W.Clegg, R.W.Harrington	Newcastle	C44 H68 Li2 N4 P2 S2
2055796	OLAFOZ	10.5517/ccdc.csd.cc27070j	W.Clegg, R.W.Harrington	Newcastle	C48 H70 N2 P2
2055797	OLAFUF	10.5517/ccdc.csd.cc27071k	W.Clegg, R.W.Harrington	Newcastle	C25 H43 N2 Sb1 Si2
2055808	OLAGAM	10.5517/ccdc.csd.cc2707dx	W.Clegg, R.W.Harrington	Newcastle	3(C17 H31 As1 O1 Si2 2-),C4 H9 O1 1-,C4 H8 O1,5(K1 1+),2(Li1 1+)
2056192	AKAQEL	10.5517/ccdc.csd.cc270msp	W.Clegg, R.W.Harrington	Newcastle	C46 H78 B2 Li2 O4 P2 Si4,2(C7 H14)
2056193	AKAQIP	10.5517/ccdc.csd.cc270mtq	W.Clegg, R.W.Harrington	Newcastle	C28 H72 B4 P4
2056196	AKAQOV	10.5517/ccdc.csd.cc270mxt	W.Clegg, R.W.Harrington	Newcastle	C50 H84 Li4 N2 O4 P2 Si6
2056197	WAVPUF02	10.5517/ccdc.csd.cc270myv	W.Clegg, R.W.Harrington	Newcastle	C9 H16 B1 N1
2056198	WAVPUF01	10.5517/ccdc.csd.cc270mzw	W.Clegg, R.W.Harrington	Newcastle	C9 H16 B1 N1
2056580	AKOSIF	10.5517/ccdc.csd.cc27119n	W.Clegg, R.W.Harrington	Newcastle	C36 H50 Si4
2056581	AKOSOL	10.5517/ccdc.csd.cc2711bp	W.Clegg, R.W.Harrington	Newcastle	C38 H82 I4 O15 Y4
2056582	AKOSUR	10.5517/ccdc.csd.cc2711cq	W.Clegg, R.W.Harrington	Newcastle	C14 H42 B2 P2 Si2
2056583	TUMPOG02	10.5517/ccdc.csd.cc2711dr	W.Clegg, R.W.Harrington	Newcastle	C18 H42 Li1 O3 Si3
2056584	AKOTEC	10.5517/ccdc.csd.cc2711fs	W.Clegg, R.W.Harrington	Newcastle	C30 H58 N2 P2 Si4
2057291	ALETUJ	10.5517/ccdc.csd.cc271s7b	W.Clegg, R.W.Harrington	Newcastle	(C12 H38 B2 Li2 P2 Si2)n
2057292	ALEVAR	10.5517/ccdc.csd.cc271s8c	W.Clegg, R.W.Harrington	Newcastle	C19 H20 B1 P1
2057293	ALEVEV	10.5517/ccdc.csd.cc271s9d	W.Clegg, R.W.Harrington	Newcastle	C12 H32 Li1 N4 1+,C4 H12 B1 1-
2057294	ALEVOF	10.5517/ccdc.csd.cc271sbf	W.Clegg, R.W.Harrington	Newcastle	C51 H65 B2 Li1 O3 P2
2058663	ELOFAP	10.5517/ccdc.csd.cc2736h2	W.Clegg, R.W.Harrington	Newcastle	(C24 H56 B3 Li2 N1 O2 P1 Si2)n
2058668	ELOFIX	10.5517/ccdc.csd.cc2736n7	W.Clegg, R.W.Harrington	Newcastle	C6 H20 B1 P1 Si1
2058669	ELOFOD	10.5517/ccdc.csd.cc2736p8	W.Clegg, R.W.Harrington	Newcastle	C56 H110 N4 P4 Si8 Sn4
2058670	ELOFUJ	10.5517/ccdc.csd.cc2736q9	W.Clegg, R.W.Harrington	Newcastle	C18 H52 B2 P2 Si4
2058671	ELOGAQ	10.5517/ccdc.csd.cc2736rb	W.Clegg, R.W.Harrington	Newcastle	C38 H62 N2 P2 Si4
2058672	ELOGEU	10.5517/ccdc.csd.cc2736sc	W.Clegg, R.W.Harrington	Newcastle	C34 H66 Li1 O3 P1 Si2

2058674	ELOGIY	10.5517/ccdc.csd.cc2736vf	W.Clegg, R.W.Harrington	Newcastle	C34 H66 Ge1 N2 P2 Si4
2059713	UMANOO	10.5517/ccdc.csd.cc2749qf	W.Clegg, R.W.Harrington	Newcastle	C10 H10 Pb1
2059722	UMANUU	10.5517/ccdc.csd.cc2749nc	W.Clegg, R.W.Harrington	Newcastle	C70 H120 Li3 P4 Si4 1-,C12 H32 Li1 N4 1+,2(C7 H8)
2059723	QUGZAW01	10.5517/ccdc.csd.cc2749pd	W.Clegg, R.W.Harrington	Newcastle	C19 H32 N1 P1 Si2
2059724	UMANOO01	10.5517/ccdc.csd.cc2749qf	W.Clegg, R.W.Harrington	Newcastle	C10 H10 Pb1
2059725	LEGFIM03	10.5517/ccdc.csd.cc2749rg	W.Clegg, R.W.Harrington	Newcastle	C6 H22 B2 N2
2059727	UMAPOQ	10.5517/ccdc.csd.cc2749tj	W.Clegg, R.W.Harrington	Newcastle	C30 H76 B2 Li2 O3 P2 Si4,C30 H76 B2 Li2 O3 P2 Si4
2059728	UMAPUW	10.5517/ccdc.csd.cc2749vk	W.Clegg, R.W.Harrington	Newcastle	C35 H60 Br2 Ge2 P2 Si2
2060498	ILAQOE	10.5517/ccdc.csd.cc2753p7	W.Clegg, R.W.Harrington	Newcastle	C18 H36 Cl1 Ge1 N1 P1 Si2 1+,Al1 Cl4 1-
2060499	ILAQUK	10.5517/ccdc.csd.cc2753q8	W.Clegg, R.W.Harrington	Newcastle	C16 H32 Cl1 Ge1 N1 P1 Si2 1+,Al1 Cl4 1-
2060506	WEXLIU03	10.5517/ccdc.csd.cc2753yh	W.Clegg, R.W.Harrington	Newcastle	C16 H32 Ca1 I2 O4
2060592	ILATEX	10.5517/ccdc.csd.cc2756qc	W.Clegg, R.W.Harrington	Newcastle	(C15 H29 K1 N1 P1 Si2)n
2060859	ILEWII	10.5517/ccdc.csd.cc275hb8	W.Clegg, R.W.Harrington	Newcastle	C15 H29 Cl1 Ge1 N1 P1 Si2
2060860	ILEWOO	10.5517/ccdc.csd.cc275hc9	W.Clegg, R.W.Harrington	Newcastle	C11 H22 B1 P1 Si1
2060861	ILEWUU	10.5517/ccdc.csd.cc275hdb	W.Clegg, R.W.Harrington	Newcastle	C32 H54 Li2 O2
2060862	ILEXAB	10.5517/ccdc.csd.cc275hfc	W.Clegg, R.W.Harrington	Newcastle	C18 H66 B6 P6 Sn2
2060863	ILEXEF	10.5517/ccdc.csd.cc275hgd	W.Clegg, R.W.Harrington	Newcastle	C78 H90 B6 P6 Sn2,4(C1 H2 Cl2)
2061991	IMIPIG	10.5517/ccdc.csd.cc276nvz	W.Clegg, R.W.Harrington	Newcastle	0.6(C15 H44 B1 P1 Si4),0.4(C14 H41 B1 Cl1 P1 Si4)
2061992	LILQAY02	10.5517/ccdc.csd.cc276nw0	W.Clegg, R.W.Harrington	Newcastle	C16 H32 Li1 O4 1+,C24 H20 B1 1-
2062000	IMIPUS	10.5517/ccdc.csd.cc276p49	W.Clegg, R.W.Harrington	Newcastle	C36 H64 Cl4 Li4 N8
2062001	ILAQUK01	10.5517/ccdc.csd.cc276p5b	W.Clegg, R.W.Harrington	Newcastle	C16 H32 Cl1 Ge1 N1 P1 Si2 1+,Al1 Cl4 1-
2062003	LUFZIV01	10.5517/ccdc.csd.cc276p7d	W.Clegg, R.W.Harrington	Newcastle	C32 H62 N2 P2 Si4
2062004	IMIQIH	10.5517/ccdc.csd.cc276p8f	W.Clegg, R.W.Harrington	Newcastle	C15 H29 Cl1 N1 P1 Si2 Sn1
2062005	IMIQON	10.5517/ccdc.csd.cc276p9g	W.Clegg, R.W.Harrington	Newcastle	C21 H40 Li1 O4 P1 S1 Si2
2062509	OLODUR	10.5517/ccdc.csd.cc2776k8	W.Clegg, R.W.Harrington	Newcastle	C41 H80 Br1 Li3 N4 P2 Si2
2062510	OLOFAZ	10.5517/ccdc.csd.cc2776l9	W.Clegg, R.W.Harrington	Newcastle	C19 H46 Si3
2062511	HAWKEY02	10.5517/ccdc.csd.cc2776mb	W.Clegg, R.W.Harrington	Newcastle	C78 H144 Li6 O6 P6 Si12,0.87(C7 H8)
2062512	OLOFIH	10.5517/ccdc.csd.cc2776nc	W.Clegg, R.W.Harrington	Newcastle	C18 H36 Cl1 Ge1 N1 P1 Si2 1+,Cl3 Ge1 1-
2062513	OLOFON	10.5517/ccdc.csd.cc2776pd	W.Clegg, R.W.Harrington	Newcastle	C25 H45 N2 P1 Si2 2+,2(Cl3 Ge1 1-)
2062514	OLOFUT	10.5517/ccdc.csd.cc2776qf	W.Clegg, R.W.Harrington	Newcastle	C21 H36 B1 O1 P1 Si2
2062515	OLOGAA	10.5517/ccdc.csd.cc2776rg	W.Clegg, R.W.Harrington	Newcastle	C16 H24 B1 P1 Si1
2062830	OMEDAO	10.5517/ccdc.csd.cc277jxy	W.Clegg, R.W.Harrington	Newcastle	C20 H54 B2 P2 Si2 Sn1
2062831	OMEDES	10.5517/ccdc.csd.cc277jyz	W.Clegg, R.W.Harrington	Newcastle	C20 H42 Li1 N2 O1 P1 Si2
2062832	OMEDIW	10.5517/ccdc.csd.cc277jz0	W.Clegg, R.W.Harrington	Newcastle	C11 H21 N2 1+,Cl1 1-
2062833	OMEDOC	10.5517/ccdc.csd.cc277k02	W.Clegg, R.W.Harrington	Newcastle	C21 H36 B1 O2 P1 Si2
2062834	OMEDUI	10.5517/ccdc.csd.cc277k13	W.Clegg, R.W.Harrington	Newcastle	C31 H51 B2 Cl1 O1 P3 Rh1 Si2 Sn1
2062899	HBUPPT02	10.5517/ccdc.csd.cc277m48	W.Clegg, R.W.Harrington	Newcastle	C24 H56 P2 Pt1
2062900	NEZCOL01	10.5517/ccdc.csd.cc277m59	W.Clegg, R.W.Harrington	Newcastle	C18 H26 Ca1 O2
2062901	OMIFUO	10.5517/ccdc.csd.cc277m6b	W.Clegg, R.W.Harrington	Newcastle	C29 H51 B1 Li1 O3 P1 Si2
2064052	AMOQEB	10.5517/ccdc.csd.cc278tbp	W.Clegg, R.W.Harrington	Newcastle	C18 H18 B1 P1
2064053	AMOQIF	10.5517/ccdc.csd.cc278tcq	W.Clegg, R.W.Harrington	Newcastle	C60 H92 P4 Sn4
2064054	IMIJIY01	10.5517/ccdc.csd.cc278tdr	W.Clegg, R.W.Harrington	Newcastle	C14 H30 B1 P1 Si2

2064055	AMOQUR	10.5517/ccdc.csd.cc278tfs	W.Clegg, R.W.Harrington	Newcastle	C50 H90 B2 Na2 O4 P2 Si4,C4 H10 O1
2064056	AMORAY	10.5517/ccdc.csd.cc278tgt	W.Clegg, R.W.Harrington	Newcastle	C28 H52 Cl2 O2 P2 Si4 Sn2
2064057	AMOREC	10.5517/ccdc.csd.cc278thv	W.Clegg, R.W.Harrington	Newcastle	C50 H90 B2 K2 O4 P2 Si4
2064058	AMORIG	10.5517/ccdc.csd.cc278tjw	W.Clegg, R.W.Harrington	Newcastle	C15 H25 P1 S2 Si2
2064059	AMOROM	10.5517/ccdc.csd.cc278tkx	W.Clegg, R.W.Harrington	Newcastle	C15 H28 B1 P1 S2 Si2
2064060	AMORUS	10.5517/ccdc.csd.cc278tly	W.Clegg, R.W.Harrington	Newcastle	C16 H32 Na1 O8 1+,C11 H21 B1 O1 P1 Si1 1-
2065416	EMUJUU	10.5517/ccdc.csd.cc27b7b5	W.Clegg, R.W.Harrington	Newcastle	C48 H42 Cl2 N2 P2 Pd2
2065417	DPPETH03	10.5517/ccdc.csd.cc27b7c6	W.Clegg, R.W.Harrington	Newcastle	C26 H24 P2
2065418	TPPOSS14	10.5517/ccdc.csd.cc27b7d7	W.Clegg, R.W.Harrington	Newcastle	C18 H15 P1 S1
2065419	EMUKIJ	10.5517/ccdc.csd.cc27b7f8	W.Clegg, R.W.Harrington	Newcastle	C19 H14 N1 O1 1+,Br1 1-,0.5(C1 H1 Cl3),0.5(H2 O1)
2065420	TPEPHO18	10.5517/ccdc.csd.cc27b7g9	W.Clegg, R.W.Harrington	Newcastle	C18 H15 O1 P1
2065421	EMUKUV	10.5517/ccdc.csd.cc27b7hb	W.Clegg, R.W.Harrington	Newcastle	3(C21 H16 O4 P1 1-),3(C6 H16 N1 1+),C21 H17 O4 P1
2065422	EMULAC	10.5517/ccdc.csd.cc27b7jc	W.Clegg, R.W.Harrington	Newcastle	C14 H18 B1 O5 P1
2065423	PUYQOP01	10.5517/ccdc.csd.cc27b7kd	W.Clegg, R.W.Harrington	Newcastle	C8 H5 F6 N1 O4 S2
2065424	EMULIK	10.5517/ccdc.csd.cc27b7lf	W.Clegg, R.W.Harrington	Newcastle	C14 H16 Br1 O3 P1
2069675	INEYOS	10.5517/ccdc.csd.cc27gnq3	W.Clegg, R.W.Harrington	Newcastle	C84 H60 O4 P4,C1 H1 Cl3
2069703	INEZAF	10.5517/ccdc.csd.cc27gpm1	W.Clegg, R.W.Harrington	Newcastle	C14 H22 N1 O4 P1,H2 O1
2069713	INEZEJ	10.5517/ccdc.csd.cc27gpyc	W.Clegg, R.W.Harrington	Newcastle	C9 H9 N1 O3 P1 1+,C9 H8 N1 O3 P1,Br1 1-,H2 O1
2069714	INEZIN	10.5517/ccdc.csd.cc27gpzd	W.Clegg, R.W.Harrington	Newcastle	C27 H27 N3 O1 P1 1+,F6 P1 1-
2069720	TPEPHO19	10.5517/ccdc.csd.cc27gq5m	W.Clegg, R.W.Harrington	Newcastle	C18 H15 O1 P1
2069721	INEZUZ	10.5517/ccdc.csd.cc27gq6n	W.Clegg, R.W.Harrington	Newcastle	C10 H6 F3 N1 O3 S1
2069743	INEYUY	10.5517/ccdc.csd.cc27gqxc	W.Clegg, R.W.Harrington	Newcastle	C6 H12 N3 P1
2069753	INEBEP	10.5517/ccdc.csd.cc27gr7q	W.Clegg, R.W.Harrington	Newcastle	C20 H20 Cl2 N4 Ni1,0.43(H2 O1)
2073384	IQUWOJ	10.5517/ccdc.csd.cc27ljcr	W.Clegg, R.W.Harrington	Newcastle	C16 H19 N1 O5 P1 1+,Cl1 1-
2073385	IQUWUP	10.5517/ccdc.csd.cc27ljds	W.Clegg, R.W.Harrington	Newcastle	C35 H36 B1 Br1 N2
2073392	ZZZFNQ02	10.5517/ccdc.csd.cc27ljm0	W.Clegg, R.W.Harrington	Newcastle	C10 H10 Br2 Co1 N2
2073394	ANTCEN24	10.5517/ccdc.csd.cc27ljp2	W.Clegg, R.W.Harrington	Newcastle	C14 H10
2076625	XIBPII02	10.5517/ccdc.csd.cc27pwxr	W.Clegg, G.S.Nichol	Newcastle	(C3 H4 N3 Na1 O4)n
2076626	BAMVAN01	10.5517/ccdc.csd.cc27pwys	W.Clegg, A.J.Edwards	Newcastle	3(C16 H36 N1 1+),H1 O12 V4 3-
2076627	DODTEY01	10.5517/ccdc.csd.cc27pwzt	W.Clegg, G.S.Nichol	Newcastle	C3 H2 N3 O3 1-,0.75(H2 O1),0.25(Na1 1+),0.75(Cs1 1+)
2076628	IQUKOX	10.5517/ccdc.csd.cc27px0w	W.Clegg, G.S.Nichol	Newcastle	(C18 H25 Cs2 K3 N18 O24)n
2076629	IQUKUD	10.5517/ccdc.csd.cc27px1x	W.Clegg, G.S.Nichol	Newcastle	C3 H2 N3 O3 1-,H2 O1,0.21(Rb1 1+),0.79(K1 1+)
2076630	IQULAK	10.5517/ccdc.csd.cc27px2y	W.Clegg, G.S.Nichol	Newcastle	10(C3 H2 N3 O3 1-),9(H2 O1),6(Rb1 1+),3(Na1 1+),H3 O1 1+
2078998	ERELUL	10.5517/ccdc.csd.cc27scgw	W.Clegg, G.S.Nichol	Newcastle	(C16 H44 Ca4 N8 O28 4+)n,4(C4 H3 N2 O3 1-)
2078999	RAYJOT01	10.5517/ccdc.csd.cc27schx	W.Clegg, G.S.Nichol	Newcastle	(C8 H10 Ba1 N4 O8)n
2079000	AROYUE01	10.5517/ccdc.csd.cc27scjy	W.Clegg, K.Guille	Newcastle	C14 H19 N2 1+,2(H2 O1),I1 1-
2079002	AROZEP	10.5517/ccdc.csd.cc27scl0	W.Clegg, G.S.Nichol	Newcastle	C14 H19 N2 1+,C4 H2 N3 O4 1-,H2 O1
2079003	FAHDUO02	10.5517/ccdc.csd.cc27scm1	W.Clegg, G.S.Nichol	Newcastle	(C5 H3 N2 O4 Rb1)n
2079004	AROZOZ	10.5517/ccdc.csd.cc27scn2	W.Clegg, G.S.Nichol	Newcastle	C10 H12 Ca1 N4 O11
2079005	AROZUF	10.5517/ccdc.csd.cc27scp3	W.Clegg, G.S.Nichol	Newcastle	C5 H10 Mg1 N2 O8,3(H2 O1)
2080574	ESICIV	10.5517/ccdc.csd.cc27v09f	W.Clegg, G.S.Nichol	Newcastle	(C5 H3 Cs1 N2 O4)n
2080575	ESICOB	10.5517/ccdc.csd.cc27v0bg	W.Clegg, G.S.Nichol	Newcastle	2(C14 H19 N2 1+),C8 H5 N4 O7 1-,C4 H3 N2 O3 1-,4(C1 H4 O1)

2080576	ESICUH	10.5517/ccdc.csd.cc27v0ch	W.Clegg, G.S.Nichol	Newcastle	(C5 H5 Cs1 N2 O5)n
2080577	ESIDAO	10.5517/ccdc.csd.cc27v0dj	W.Clegg, G.S.Nichol	Newcastle	(C8 H10 Cs1 Li1 N4 O8)n
2080578	ESIDES	10.5517/ccdc.csd.cc27v0fk	W.Clegg, G.S.Nichol	Newcastle	H12 Mg1 O6 2+,2(C4 H3 N2 O3 1-),3(H2 O1)
2080579	ESIDIW	10.5517/ccdc.csd.cc27v0gl	W.Clegg, G.S.Nichol	Newcastle	C14 H19 N2 1+,C5 H3 N2 O4 1-,2(H2 O1)
2080580	SOTJOA01	10.5517/ccdc.csd.cc27v0hm	W.Clegg, G.S.Nichol	Newcastle	(C5 H8 Ca1 N2 O7)n,H2 O1
2081563	GUYRUN04	10.5517/ccdc.csd.cc27w16d	W.Clegg, K.Guille	Newcastle	C8 H12 Cu1 N4 O9
2081564	RAGLAO01	10.5517/ccdc.csd.cc27w17f	W.Clegg, K.Guille	Newcastle	(C8 H10 Mn1 N4 O8)n
2081565	DEFWUJ01	10.5517/ccdc.csd.cc27w18g	W.Clegg, K.Guille	Newcastle	C8 H14 Co1 N4 O10
2081568	SEDDOX01	10.5517/ccdc.csd.cc27w1ck	W.Clegg, K.Guille	Newcastle	(C4 H7 Li1 N2 O5)n
2081572	UTIWOM	10.5517/ccdc.csd.cc27w1hp	W.Clegg, K.Guille	Newcastle	(C4 H4 N3 Na1 O5)n
2081573	UTIWUS	10.5517/ccdc.csd.cc27w1jq	W.Clegg, K.Guille	Newcastle	C8 H12 Ca1 N6 O12,2(H2 O1)
2081574	UTIXAZ	10.5517/ccdc.csd.cc27w1kr	W.Clegg, K.Guille	Newcastle	(C8 H14 K2 N6 O16 S1)n
2082528	ITABIR	10.5517/ccdc.csd.cc27x1bk	W.Clegg, K.Guille	Newcastle	(C8 H4 N5 Na1 O6)n,H2 O1
2082529	ITABOX	10.5517/ccdc.csd.cc27x1cl	W.Clegg, K.Guille	Newcastle	C8 H6 N4 O6,H2 O1
2082530	ITABUD	10.5517/ccdc.csd.cc27x1dm	W.Clegg, K.Guille	Newcastle	C8 H6 N4 O6
2082531	ITACAK	10.5517/ccdc.csd.cc27x1fn	W.Clegg, K.Guille	Newcastle	C8 H7 N5 O6,3(H2 O1)
2082532	ITACEO	10.5517/ccdc.csd.cc27x1gp	W.Clegg, K.Guille	Newcastle	C8 H6 N4 O6,2(C4 H3 N3 O4),2(H2 O1)
2082533	ITACIS	10.5517/ccdc.csd.cc27x1hq	W.Clegg, K.Guille	Newcastle	C8 H5 N4 O7 1-,H4 N1 1+,H2 O1
2082534	ITACOY	10.5517/ccdc.csd.cc27x1jr	W.Clegg, K.Guille	Newcastle	(C8 H9 Li1 N4 O8)n,3(H2 O1)
2082535	ITACUE	10.5517/ccdc.csd.cc27x1ks	W.Clegg, K.Guille	Newcastle	(C7 H11 K1 N2 O3)n
2082536	ITADAL	10.5517/ccdc.csd.cc27x1lt	W.Clegg, K.Guille	Newcastle	(C6 H9 K1 N2 O3)n
2083362	OSOLIU	10.5517/ccdc.csd.cc27xx7b	W.Clegg, K.Guille	Newcastle	(C8 H7 Cs1 N4 O8)n
2083363	OSOLOA	10.5517/ccdc.csd.cc27xx8c	W.Clegg, K.Guille	Newcastle	(C16 H14 Cs2 N10 O16)n
2083364	OSOLUG	10.5517/ccdc.csd.cc27xx9d	W.Clegg, K.Guille	Newcastle	C16 H18 Ca1 N8 O16,H2 O1
2083365	OSONAO	10.5517/ccdc.csd.cc27xxbf	W.Clegg, K.Guille	Newcastle	C8 H15 Ca1 N4 O12 1+,C8 H4 N4 O6 2-,H4 N1 1+,2(H2 O1)
2083366	OSONES	10.5517/ccdc.csd.cc27xxcg	W.Clegg, K.Guille	Newcastle	(C12 H14 K2 N6 O13)n
2083367	OSONIW	10.5517/ccdc.csd.cc27xxdh	W.Clegg, K.Guille	Newcastle	(C16 H15 N10 Na3 O17)n,2(H2 O1)
2083369	OSONOC	10.5517/ccdc.csd.cc27xxgk	W.Clegg, K.Guille	Newcastle	(C16 H34 N10 Na4 O26)n
2085843	VIOLME09	10.5517/ccdc.csd.cc280h82	W.Clegg, K.Guille	Newcastle	C4 H3 N3 O4,H2 O1
2085844	EVELAV	10.5517/ccdc.csd.cc280h93	W.Clegg, K.Guille	Newcastle	C14 H19 N2 1+,C4 H3 N2 O3 1-
2085845	EVELEZ	10.5517/ccdc.csd.cc280hb4	W.Clegg, K.Guille	Newcastle	(C8 H5 N4 O7 Rb1)n,H2 O1
2085846	EVELID	10.5517/ccdc.csd.cc280hc5	W.Clegg, K.Guille	Newcastle	C32 H25 Li3 N16 O28
2085849	EVELOJ	10.5517/ccdc.csd.cc280hg8	W.Clegg, K.Guille	Newcastle	(C16 H18 N8 Na2 O17)n,4(H2 O1)
2086606	UVIZUX	10.5517/ccdc.csd.cc2818wh	W.Clegg	UK	C41 H58 O1 P2 Pd1
2086607	UVIZUX01	10.5517/ccdc.csd.cc2818xj	W.Clegg	UK	C41 H58 O1 P2 Pd1
2086608	UVOBEP	10.5517/ccdc.csd.cc2818yk	W.Clegg	UK	C34 H28 I2 N2 P2 Pd1
2086609	UVOBIT	10.5517/ccdc.csd.cc2818zl	W.Clegg	UK	C24 H48 O2 P2 Pd1 2+,2(B1 F4 1-)
2086610	UVOBOZ	10.5517/ccdc.csd.cc28190n	W.Clegg	UK	0.33(C51 H42 O3 Pd2),0.67(C51 H42 O3 Pd1)
2086611	LAJKEO01	10.5517/ccdc.csd.cc28191p	W.Clegg	UK	C24 H44 Cl2 P2 Pd1
2086612	UVOCAM	10.5517/ccdc.csd.cc28192q	W.Clegg, M.R.J.Elsegood	UK	C17 H15 O1 1+,B1 F4 1-
2086712	UVOTEH	10.5517/ccdc.csd.cc281d91	W.Clegg, M.R.J.Elsegood	UK	C37 H50 O1 P2 Pd1
2086713	UVOTIL	10.5517/ccdc.csd.cc281db2	W.Clegg, M.R.J.Elsegood	UK	C24 H44 Cl2 P2 Pd1,C1 H2 Cl2

2086714	UVOTOR	10.5517/ccdc.csd.cc281dc3	W.Clegg, M.R.J.Elsegood	UK	C51 H42 O3 Pd2,C7 H8
2086715	UVOTUX	10.5517/ccdc.csd.cc281dd4	W.Clegg, M.R.J.Elsegood	UK	C26 H48 O2 P2
2086716	IGIZUT01	10.5517/ccdc.csd.cc281df5	W.Clegg, M.R.J.Elsegood, S.L.Heath	UK	C24 H44 O2 P2 Pd1
2087032	UVUSAI	10.5517/ccdc.csd.cc281qmp	W.Clegg, S.L.Heath	UK	C22 H42 O6 P2 Pd1 S2,2(C4 H8 O1)
2087033	UVUSEM	10.5517/ccdc.csd.cc281qnq	W.Clegg, M.R.J.Elsegood	UK	C49 H66 O1 P2 Pd1,2.5(C7 H8)
2087034	UVUSIQ	10.5517/ccdc.csd.cc281qpr	W.Clegg	UK	C24 H48 O2 P2 Pd1 2+,2(C7 H7 O3 S1 1-)
2087780	ITUSIC	10.5517/ccdc.csd.cc282hrm	W.Clegg	UK	C50 H90 Br2 O2 P4 Pd2,2(C4 H8 O1)
2087781	ITUSOI	10.5517/ccdc.csd.cc282hsn	W.Clegg	UK	C44 H40 O1 P2 Pd1
2087782	ITUSUO	10.5517/ccdc.csd.cc282htp	W.Clegg, M.R.J.Elsegood, A.J.Edwards	UK	C53 H44 O1 P2 Pd1,C4 H8 O1
2087783	ITUTAV	10.5517/ccdc.csd.cc282hvq	W.Clegg, M.R.J.Elsegood, A.J.Edwards	UK	C24 H44 O1 P2
2087784	EXIRIM16	10.5517/ccdc.csd.cc282hwr	W.Clegg, A.J.Edwards	UK	C17 H14 O1
2087785	ITUTID	10.5517/ccdc.csd.cc282hxs	W.Clegg, A.J.Edwards	UK	C24 H43 N1 O2 P2
2087786	ITUTOJ	10.5517/ccdc.csd.cc282hyt	W.Clegg, A.J.Edwards	UK	C17 H15 O1 1+,C1 H4 O3 S1,C1 H3 O3 S1 1-
2088692	IWAYUD	10.5517/ccdc.csd.cc283g51	W.Clegg, A.J.Edwards	UK	C39 H54 O3 P2 Pd1
2088693	IWAZAK	10.5517/ccdc.csd.cc283g62	W.Clegg, A.J.Edwards	UK	C28 H52 O2 P2 Pd1
2088694	UQEWIZ01	10.5517/ccdc.csd.cc283g73	W.Clegg, M.R.J.Elsegood, L.Horsburgh	UK	C36 H28 P2
2088695	IWAZOY	10.5517/ccdc.csd.cc283g84	W.Clegg, L.Horsburgh	UK	C41 H58 O1 P2 Pt1
2088697	IWAZUE	10.5517/ccdc.csd.cc283gb6	W.Clegg, L.Horsburgh	UK	C28 H46 O2 P2 Pd1
2088699	IWEBAQ	10.5517/ccdc.csd.cc283gd8	W.Clegg, M.R.J.Elsegood	UK	C28 H46 O2 P2 Pd1,C4 H8 O1
2088704	IWEBEU	10.5517/ccdc.csd.cc283gkf	W.Clegg, M.R.J.Elsegood, L.Horsburgh	UK	C53 H42 O2 P2 Pd1,1.5(C4 H8 O1)
2090261	AVUBEB	10.5517/ccdc.csd.cc2852sb	W.Clegg, L.Horsburgh	UK	C19 H42 Cl2 P2 Pd1,0.5(C7 H8)
2090262	AVUBIF	10.5517/ccdc.csd.cc2852tc	W.Clegg, L.Horsburgh	UK	C26 H48 O2 P2 Pd1
2090264	AVUBOL	10.5517/ccdc.csd.cc2852wf	W.Clegg, L.Horsburgh	UK	C53 H42 O1 P2 Pd1
2090266	AVUBUR	10.5517/ccdc.csd.cc2852yh	W.Clegg, L.Horsburgh	UK	C32 H56 O2 P2 Pd1 2+,2(C1 H3 O3 S1 1-)
2090269	AVUCAY	10.5517/ccdc.csd.cc28531m	W.Clegg, M.R.J.Elsegood	UK	C25 H46 O2 P2 Pd1
2090271	AVUCEC	10.5517/ccdc.csd.cc28533p	W.Clegg, L.Horsburgh	UK	C38 H41 O4 P2 Pd1 S1 1+,C7 H7 O3 S1 1-
2090891	AWOGEB	10.5517/ccdc.csd.cc285r3b	W.Clegg, L.Horsburgh	UK	C42 H32 O3 P2 Pd1,C1 H2 Cl2
2090892	AWOGIF	10.5517/ccdc.csd.cc285r4c	W.Clegg, M.R.J.Elsegood, L.Horsburgh	UK	C45 H36 O3 P2 Pd1,C1 H2 Cl2
2090912	AWOGOL	10.5517/ccdc.csd.cc285rs0	W.Clegg, P.A.Champkin	UK	C26 H47 Cu1 O2 P2 1+,C2 H3 O2 1-
2090915	AWOGUR	10.5517/ccdc.csd.cc285rw3	W.Clegg, P.A.Champkin	UK	C24 H44 Br1 P2 Pd1,0.25(C7 H8)
2092195	EWEYEN	10.5517/ccdc.csd.cc28735t	W.Clegg, P.A.Champkin	UK	C25 H46 Cl2 P2 Pd1
2092198	EWEYIR	10.5517/ccdc.csd.cc28738x	W.Clegg, P.A.Champkin	UK	(C4 H6 Cl2 N2 Ni1)n
2092199	EWEYOX	10.5517/ccdc.csd.cc28739y	W.Clegg, P.A.Champkin	UK	C24 H44 Cl3 Ni1 P2
2092200	LAJKAK01	10.5517/ccdc.csd.cc2873bz	W.Clegg, P.A.Champkin	UK	C24 H44 P2
2092201	OWEFUS01	10.5517/ccdc.csd.cc2873c0	W.Clegg, P.A.Champkin	UK	C25 H44 F3 O3 P2 Pd1 S1 1+,C1 F3 O3 S1 1-
2092202	EWEZEO	10.5517/ccdc.csd.cc2873d1	W.Clegg, P.A.Champkin	UK	C24 H48 O2 P2 Pd1 2+,2(C1 F3 O3 S1 1-)
2092203	EWEZIS	10.5517/ccdc.csd.cc2873f2	W.Clegg, P.A.Champkin	UK	C24 H44 Br3 Ni1 P2
2092204	EWEZOY	10.5517/ccdc.csd.cc2873g3	W.Clegg, P.A.Champkin	UK	C27 H26 P2,C1 H2 Cl2
2092205	LAJKEO02	10.5517/ccdc.csd.cc2873h4	W.Clegg, P.A.Champkin	UK	C24 H44 Cl2 P2 Pd1

2092206	EWIBAQ	10.5517/ccdc.csd.cc2873j5	W.Clegg, P.A.Champkin	UK	C26 H46 O2 P2 Pd1,C2 H4 O2
2092209	EWIBEU	10.5517/ccdc.csd.cc2873m8	W.Clegg, P.A.Champkin	UK	C49 H42 O1 P2 Pd1
2095705	OYANUA	10.5517/ccdc.csd.cc28brds	W.Clegg, K.Guille	Newcastle	C4 H3 N2 O3 1-,C3 H2 N3 O3 1-,3.5(H2 O1),2(Na1 1+)
2095707	ZAHJIB03	10.5517/ccdc.csd.cc28brgv	W.Clegg, K.Guille	Newcastle	C3 H3 N3 O3,2(H2 O1)
2095708	VUQTOT02	10.5517/ccdc.csd.cc28brhw	W.Clegg, K.Guille	Newcastle	(C6 H8 N6 O8 Sr1)n
2095709	OYAPOW	10.5517/ccdc.csd.cc28brjx	W.Clegg, K.Guille	Newcastle	C3 H3 N3 O3,C4 H3 N2 O3 1-,3(H2 O1),K1 1+
2095710	OYAPUC	10.5517/ccdc.csd.cc28brky	W.Clegg, K.Guille	Newcastle	C4 H3 N2 O3 1-,C3 H3 N3 O3,2(H2 O1),Cs1 1+
2095883	OYEJOU	10.5517/ccdc.csd.cc28by4q	W.Clegg, K.Guille	Newcastle	C4 H2 N3 O4 1-,C3 H3 N3 O3,2(H2 O1),Cs1 1+
2095884	OYEJUA	10.5517/ccdc.csd.cc28by5r	W.Clegg, K.Guille	Newcastle	(C12 H28 N6 O28 Sr4 2+)n,2(C3 H2 N3 O3 1-),2(H2 O1)
2097520	CYTOSM17	10.5517/ccdc.csd.cc28dmy8	W.Clegg, K.Guille	Newcastle	C4 H5 N3 O1,H2 O1
2097521	AZABAH	10.5517/ccdc.csd.cc28dmz9	W.Clegg, K.Guille	Newcastle	C3 H3 N2 O4 1-,Rb1 1+
2097522	AZABEL	10.5517/ccdc.csd.cc28dn0c	W.Clegg, K.Guille	Newcastle	(C7 H6 Ba1 N4 O10)n
2097523	ROMTET01	10.5517/ccdc.csd.cc28dn1d	W.Clegg, K.Guille	Newcastle	(C3 H5 K1 N2 O5)n
2098930	SRVIOL01	10.5517/ccdc.csd.cc28g3f9	W.Clegg, K.Guille	Newcastle	(C8 H12 N6 O12 Sr1)n
2098931	ITACIS01	10.5517/ccdc.csd.cc28g3gb	W.Clegg, K.Guille	Newcastle	C8 H5 N4 O7 1-,H4 N1 1+,H2 O1
2100386	THYMMH01	10.5517/ccdc.csd.cc28hmdt	W.Clegg, K.Guille	Newcastle	C5 H6 N2 O2,H2 O1
2100387	DMANAP12	10.5517/ccdc.csd.cc28hmfv	W.Clegg, K.Guille	Newcastle	C14 H18 N2
2100388	AROYUE	10.5517/ccdc.csd.cc28hmgw	W.Clegg, G.S.Nichol	Newcastle	C14 H19 N2 1+,2(H2 O1),I1 1-
2100389	UZUSEQ	10.5517/ccdc.csd.cc28hmhx	W.Clegg, K.Guille	Newcastle	(C8 H10 N4 Na2 O8)n
2100581	ITACAK01	10.5517/ccdc.csd.cc28htp9	W.Clegg, K.Guille	Newcastle	C8 H7 N5 O6,3(H2 O1)
2100582	UDEGOA01	10.5517/ccdc.csd.cc28htqb	W.Clegg, K.Guille	Newcastle	C14 H19 N2 1+,I1 1-
2100583	UBEJEU	10.5517/ccdc.csd.cc28htrc	W.Clegg, K.Guille	Newcastle	C14 H19 N2 1+,C1 H4 O1,I1 1-
2100584	ZIMTEW01	10.5517/ccdc.csd.cc28htsd	W.Clegg, K.Guille	Newcastle	(C8 H11 N4 Na1 O8)n
2100586	ITACIS02	10.5517/ccdc.csd.cc28htvg	W.Clegg, K.Guille	Newcastle	H4 N1 1+,C8 H5 N4 O7 1-,H2 O1
2111603	KVIOLD04	10.5517/ccdc.csd.cc28w97q	W.Clegg, K.Guille	Newcastle	(C4 H6 K1 N3 O6)n
2111605	ERELUL01	10.5517/ccdc.csd.cc28w99s	W.Clegg, K.Guille	Newcastle	(C16 H44 Ca4 N8 O28 4+)n,4(C4 H3 N2 O3 1-)
2111606	UCUSIY	10.5517/ccdc.csd.cc28w9bt	W.Clegg, K.Guille	Newcastle	2(C3 H3 N3 O3),4(C3 H2 N3 O3 1-),5(H2 O1),4(Cs1 1+)
2115233	OCOQOQ	10.5517/ccdc.csd.cc2902br	W.Clebb, K.Guille	Newcastle	1.75(Na1 1+),1.25(Cs1 1+),C8 H5 N4 O6 1-,2(C4 H2 N3 O4),2(H2 O1)
2115234	UTIXAZ01	10.5517/ccdc.csd.cc2902cs	W.Clegg, K.Guille	Newcastle	(C8 H14 K2 N6 O16 S1)n
2115235	KVIOLD05	10.5517/ccdc.csd.cc2902dt	W.Clegg, K.Guille	Newcastle	(C4 H6 K1 N3 O6)n
2120255	YUKBIP03	10.5517/ccdc.csd.cc2959b4	W.Clegg, Z.Yuan	Newcastle	(C3 H9 Al2 O9 P3)n
2120256	XUWVEQ01	10.5517/ccdc.csd.cc2959c5	W.Clegg, Z.Yuan	Newcastle	(C2 H8 F2 Ga2 O8 P2)n,H2 O1
2120257	IZENEH01	10.5517/ccdc.csd.cc2959d6	W.Clegg, Z.Yuan	Newcastle	(C2 H7 Al1 O7 P2)n
2120259	PANKOI	10.5517/ccdc.csd.cc2959g8	W.Clegg, Z.Yuan	Newcastle	(C2 H7 Al1 O12 P4 2-)n,2(Cs1 1+)
2121223	ZAMNIO	10.5517/ccdc.csd.cc2969kd	W.Clegg	Newcastle	C16 H24 Cl2 Fe1 N2 Ni1
2129186	TARLAD	10.5517/ccdc.csd.cc29glft	W.Clegg, J.D.Kennedy, M.G.S.Londesborough	Leeds	H40 B36 Pt1 2-,2(C14 H19 N2 1+)
2129187	TARLEH	10.5517/ccdc.csd.cc29glgv	W.Clegg, J.D.Kennedy, M.G.S.Londesborough	Leeds	H40 B36 Pt1 2-,2(C14 H19 N2 1+),2(C1 H2 Cl2)
2129195	TARLIL	10.5517/ccdc.csd.cc29glq3	W.Clegg, J.D.Kennedy, M.G.S.Londesborough	Leeds	0.86(C24 H49 B16 P3 Pd2),0.14(C24 H48 B16 Cl1 P3 Pd2)
2129196	TARLOR	10.5517/ccdc.csd.cc29glr4	W.Clegg, J.D.Kennedy, M.G.S.Londesborough	Leeds	C16 H40 B18 P2 Pd1
2129198	TARLUX	10.5517/ccdc.csd.cc29glt6	W.Clegg, J.D.Kennedy, C.O'Dowd	Leeds	C24 H34 B10 N2

2129201	TARMAE	10.5517/ccdc.csd.cc29glx9	W.Clegg, J.D.Kennedy, M.G.S.Londesborough	Leeds	C6 H37 B18 O6 P2 Pt1 1-,C14 H19 N2 1+,2(C6 H6)
2129204	TARMEI	10.5517/ccdc.csd.cc29gm0f	W.Clegg, J.D.Kennedy, R.D.Kennedy	Leeds	C10 H15 N2 1+,H4 B5 O10 1-,0.5(C4 H10 O1),1.5(C2 H3 N1)
2129205	TARMIM	10.5517/ccdc.csd.cc29gm1g	W.Clegg, J.D.Kennedy, M.G.S.Londesborough	Leeds	C26 H43 B18 P2 Pd1
2131104	BAYTUR01	10.5517/ccdc.csd.cc29jl9r	W.Clegg	Leeds	C70,C35 H30 O5
2132888	WATYUP	10.5517/ccdc.csd.cc29lfv6	W.Clegg, Z.Yuan	Newcastle	2(H4 N1 1+),n(C2 H6 Al1 F1 O6 P2 2-),H2 O1
2132889	WATZAW	10.5517/ccdc.csd.cc29lfw7	W.Clegg, Z.Yuan	Newcastle	(C1 H5 Al1 O7 P2)n
2132890	WATZEA	10.5517/ccdc.csd.cc29lfx8	W.Clegg, Z.Yuan	Newcastle	C6 H22 N4 4+,(C2 H7 Ga1 O12 P4 3-)n,(C2 H7 Ga1 O12 P4 1-)n
2132891	WATZIE	10.5517/ccdc.csd.cc29lfy9	W.Clegg, Z.Yuan	Newcastle	(C1 H3 Ga1 O7 P2 2-)n,2(H4 N1 1+)
2133089	WAWFIN	10.5517/ccdc.csd.cc29lnbx	W.Clegg, Z.Yuan	Newcastle	(C9 H18 Al4 O18 P6)n
2133090	WAWFOT	10.5517/ccdc.csd.cc29lncy	W.Clegg, Z.Yuan	Newcastle	(C2 H9 Ga1 O12 P4)n
2133091	WAWFUZ	10.5517/ccdc.csd.cc29lndz	W.Clegg, Z.Yuan	Newcastle	(C2 H9 Al1 O12 P4)n
2133092	WAWGAG	10.5517/ccdc.csd.cc29lnf0	W.Clegg, Z.Yuan	Newcastle	(C2 H10 Al2 O10 P2)n
2133237	WAXXAY	10.5517/ccdc.csd.cc29lt3v	W.Clegg, Z.Yuan	Newcastle	(C2 H7 Ga1 O12 P4 2-)n,2n(Cs1 1+)
2133238	IZENEH03	10.5517/ccdc.csd.cc29lt4w	W.Clegg, Z.Yuan	Newcastle	(C2 H7 Al1 O7 P2)n
2141350	WAWFIN01	10.5517/ccdc.csd.cc29w7t8	W.Clegg, Z.Yuan	Newcastle	(C36 H72 Al16 O72 P24)n
2141353	WAWFIN02	10.5517/ccdc.csd.cc29w7xc	W.Clegg, Z.Yuan	Newcastle	(C9 H18 Al4 O18 P6)n
2142878	MAQPUT	10.5517/ccdc.csd.cc29xv36	W.Clegg, J.D.Kennedy, U.Doerfler	Leeds	C15 H29 B8 N3
2142879	MARCIV	10.5517/ccdc.csd.cc29xv47	W.Clegg, J.D.Kennedy, S.D.Perera, M.J.Carr	Leeds	C20 H46 B16 Cl2 Ru2
2142881	MARCOB	10.5517/ccdc.csd.cc29xv69	W.Clegg, J.D.Kennedy, N.J.Bullen, A.Franken	Leeds	C14 H22 B9 O1 1-,C8 H20 N1 1+
2142882	MARCUH	10.5517/ccdc.csd.cc29xv7b	W.Clegg, J.D.Kennedy, S.D.Perera, M.J.Carr, A.Franken	Leeds	C17 H27 B8 Cl2 Ru1 1-,C14 H19 N2 1+
2142891	MARDES	10.5517/ccdc.csd.cc29xvjm	W.Clegg, J.D.Kennedy, M.J.Carr, S.D.Perera	Leeds	C5 H25 B18 Ni1 1-,C14 H19 N2 1+
2142894	MARDIW	10.5517/ccdc.csd.cc29xvmq	W.Clegg, J.D.Kennedy, M.J.Carr, M.G.S.Londesborough	Leeds	C16 H40 B18 P2 Pt1 S2
2142897	MARDOC	10.5517/ccdc.csd.cc29xvqt	W.Clegg, J.D.Kennedy, M.J.Carr, M.G.S.Londesborough	Leeds	C16 H37 B17 P2 Pt1 S2,C1 H2 Cl2
2142898	MARDUI	10.5517/ccdc.csd.cc29xvrv	W.Clegg, J.D.Kennedy, M.J.Carr	Leeds	C12 H18 B8 Ni1
2142900	MARFOE	10.5517/ccdc.csd.cc29xvtx	W.Clegg, J.D.Kennedy, M.J.Carr	Leeds	C20 H46 B17 Cl1 Ru2
2142902	MARGAR	10.5517/ccdc.csd.cc29xvwz	W.Clegg, J.D.Kennedy, R.D.Kennedy	Leeds	C20 H40 B10 N4
2144508	AWIPAX01	10.5517/ccdc.csd.cc29zjpj	W.Clegg, J.D.Kennedy, M.J.Carr, A.Franken	Leeds	C14 H22 B11 1-,C8 H20 N1 1+
2144509	QATRIQ	10.5517/ccdc.csd.cc29zjqk	W.Clegg, J.D.Kennedy, M.J.Carr	Leeds	C14 H19 N2 1+,C5 H16 B11 Ni1 1-
2144510	QATROW	10.5517/ccdc.csd.cc29zjrl	W.Clegg, J.D.Kennedy, M.J.Carr	Leeds	C5 H23 B18 Ni1 1-,C14 H19 N2 1+
2144511	QATRUC	10.5517/ccdc.csd.cc29zjsm	W.Clegg, J.D.Kennedy, M.J.Carr, M.G.S.Londesborough	Leeds	C16 H37 B17 P2 Pt1 S2
2145533	YAQDAZ	10.5517/ccdc.csd.cc2b0lrq	W.Clegg, J.D.Kennedy, M.G.S.Londesborough	Leeds	0.9(H32 B28 Pd1 2-),2(C14 H19 N2 1+),0.1(H31 B28 Cl1 Pd1 2-)
2145534	YAQDED	10.5517/ccdc.csd.cc2b0lsr	W.Clegg, J.D.Kennedy, M.G.S.Londesborough	Leeds	H40 B36 Pd1 2-,2(C14 H19 N2 1+)
2145536	YAQDIH	10.5517/ccdc.csd.cc2b0lvt	W.Clegg, J.D.Kennedy, M.G.S.Londesborough	Leeds	C16 H42 B18 P2 Pd1
2149418	YAQDED01	10.5517/ccdc.csd.cc2b4n27	W.Clegg, J.D.Kennedy,	Leeds	H40 B36 Pd1 2-,2(C14 H19 N2 1+)

			M.G.S.Londesborough		
2149419	YASLAJ	10.5517/ccdc.csd.cc2b4n38	W.Clegg, J.D.Kennedy, P.Dosanjh	Leeds	C16 H37 B15 P2 Pt1 S2
2152308	MPPZNB01	10.5517/ccdc.csd.cc2b7n9k	W.Clegg, J.D.Kennedy, J.A.McGinnety, M.Thornton-Pett	Leeds	H24 B20 Zn1 2-,2(C19 H18 P1 1+)
2152309	VAYXAY	10.5517/ccdc.csd.cc2b7nbl	W.Clegg, J.D.Kennedy, T.D.McGrath	Leeds	H20 B18 Br2
2152310	VAYXEC	10.5517/ccdc.csd.cc2b7ncm	W.Clegg, J.D.Kennedy, T.D.McGrath	Leeds	H20 B18 Br2,C7 H8
2152311	VAYXIG	10.5517/ccdc.csd.cc2b7ndn	W.Clegg, J.D.Kennedy, T.D.McGrath	Leeds	H38 Au1 B36 Cl1 2-,2(C14 H19 N2 1+)
2153110	DAWDOY	10.5517/ccdc.csd.cc2b8h59	W.Clegg, J.D.Kennedy, M.G.S.Londesborough	Leeds	C32 H60 B18 P4 Pd2
2153111	DAWDUE	10.5517/ccdc.csd.cc2b8h6b	W.Clegg, J.D.Kennedy, M.G.S.Londesborough	Leeds	C32 H61 B18 Cl1 P4 Pt2,4(C1 H1 Cl3)
2153113	DAWFAM	10.5517/ccdc.csd.cc2b8h8d	W.Clegg, J.D.Kennedy, M.G.S.Londesborough	Leeds	C16 H42 B18 P2 Pt1
2153114	DAWFEQ	10.5517/ccdc.csd.cc2b8h9f	W.Clegg, J.D.Kennedy, M.G.S.Londesborough	Leeds	C32 H59 B18 Cl1 P4 Pt2
2153115	DAWFIU	10.5517/ccdc.csd.cc2b8hbg	W.Clegg, J.D.Kennedy, M.G.S.Londesborough	Leeds	C16 H42 B18 P2 Pt1
2153371	MESBAN01	10.5517/ccdc.csd.cc2b8rlz	W.Clegg, Z.Yuan	Newcastle	(C6 H12 Ga4 O18 P6)n
2153372	PURBIP01	10.5517/ccdc.csd.cc2b8rm0	W.Clegg, Z.Yuan	Newcastle	C6 H16 O6 P2
2153373	UDIPOM02	10.5517/ccdc.csd.cc2b8rn1	W.Clegg, Z.Yuan	Newcastle	C4 H12 O6 P2
2153655	DAXVIL	10.5517/ccdc.csd.cc2b91rg	W.Clegg, Z.Yuan	Newcastle	C20 H20 Cl2 N4 Ru1,0.37(H2 O1)
2154991	RAYQIV	10.5517/ccdc.csd.cc2bbfvz	W.Clegg, Z.Yuan	Newcastle	(C10 H25 Al1 O12 P4)n
2154992	ULIKAD01	10.5517/ccdc.csd.cc2bbfw0	W.Clegg, Z.Yuan	Newcastle	2(C10 H23 O6 P2 1-),C10 H10 N2 2+
2154993	ULIKAD02	10.5517/ccdc.csd.cc2bbfx1	W.Clegg, Z.Yuan	Newcastle	2(C10 H23 O6 P2 1-),C10 H10 N2 2+
2154994	RAYRAO	10.5517/ccdc.csd.cc2bbfy2	W.Clegg, Z.Yuan	Newcastle	(C2 H6 Al3 F6 O6 P2)n,H2 O1
2155464	CASGEM	10.5517/ccdc.csd.cc2bby3r	W.Clegg, Z.Yuan	Newcastle	(C1 H5 F1 Ga1 O4 P1)n
2155474	PURCAI01	10.5517/ccdc.csd.cc2bbyf2	W.Clegg, Z.Yuan	Newcastle	C10 H24 O6 P2
2155475	RAPHUM01	10.5517/ccdc.csd.cc2bbyg3	W.Clegg, Z.Yuan	Newcastle	C5 H14 O6 P2
2156985	JAVGEW	10.5517/ccdc.csd.cc2bdj5g	W.Clegg, Z.Yuan, G.S.Nichol	Newcastle	(C4 H16 F4 Ga4 O16 P4)n,2(H2 O1)
2157192	JAWXOY	10.5517/ccdc.csd.cc2bdqvb	W.Clegg, Z.Yuan	Newcastle	(C10 H22 Ga6 O30 P10)n
2157652	JEBGIK	10.5517/ccdc.csd.cc2bf6pp	W.Clegg, J.D.Kennedy, J.Bould, P.J.Zheng	Leeds	C80 H85 B20 O1 P5 Pd4,C1 H1 Cl3
2157656	DAWFAM01	10.5517/ccdc.csd.cc2bf6tt	W.Clegg, J.D.Kennedy, M.G.S.Londesborough	Leeds	C16 H42 B18 P2 Pt1
2157658	JEBGUW	10.5517/ccdc.csd.cc2bf6ww	W.Clegg, J.D.Kennedy, M.G.S.Londesborough	Leeds	C6 H38 B18 O6 P2 Pt1,0.5(C6 H6)
2157659	JEBHAD	10.5517/ccdc.csd.cc2bf6xx	W.Clegg, J.D.Kennedy, M.G.S.Londesborough	Leeds	C16 H42 B18 P2 Pd1
2157660	JEBHEH	10.5517/ccdc.csd.cc2bf6yy	W.Clegg, J.D.Kennedy, M.G.S.Londesborough	Leeds	C6 H38 B18 O6 P2 Pt1
2157678	JEBWUM	10.5517/ccdc.csd.cc2bf7jk	W.Clegg, J.D.Kennedy, M.G.S.Londesborough	Leeds	C6 H39 B18 O6 P2 Pt1
2158512	BARBAC04	10.5517/ccdc.csd.cc2bg3fc	W.Clegg, G.S.Nichol	Leeds	C4 H4 N2 O3
2158516	BAWDUC	10.5517/ccdc.csd.cc2bg3kh	W.Clegg, J.D.Kennedy, M.J.Carr, M.G.S.Londesborough	Leeds	H18 B17 S2 1-,C14 H19 N2 1+
2161792	ZAZTED	10.5517/ccdc.csd.cc2bkj7p	W.Clegg, J.D.Kennedy, J.Bould	Leeds	H24 B20 Hg1 2-,2(C14 H19 N2 1+)
2161793	ZAZTIH	10.5517/ccdc.csd.cc2bkj8q	W.Clegg, J.D.Kennedy, J.Bould	Leeds	H40 B36 Hg1 2-,2(C14 H19 N2 1+),4(C1 H1 Cl3)

2161794	ZAZTON	10.5517/ccdc.csd.cc2bkj9r	W.Clegg, J.D.Kennedy, J.Bould	Leeds	H40 B36 Hg1 2-,2(C14 H19 N2 1+)
2161799	ZAZTUT	10.5517/ccdc.csd.cc2bkjgx	W.Clegg, J.D.Kennedy, J.Bould	Leeds	2(C14 H19 N2 1+),Cl4 Hg1 2-
2161800	ZAZVAB	10.5517/ccdc.csd.cc2bkjhy	W.Clegg, J.D.Kennedy, T.D.McGrath	Leeds	H40 B36 Hg1 2-,2(C14 H19 N2 1+)
2161822	ZEBKAW	10.5517/ccdc.csd.cc2bkk6p	W.Clegg, J.D.Kennedy, P.I.MacKinnon, M.Thornton-Pett	Leeds	C26 H55 B18 P2 Pt1 Rh1
2162998	SAXNIS	10.5517/ccdc.csd.cc2bls4w	W.Clegg, J.D.Kennedy, M.J.Carr, S.D.Perera	Leeds	C23 H39 B18 O1 P1 Ru1,C1 H1 Cl3
2163000	SAXNOY	10.5517/ccdc.csd.cc2bls6y	W.Clegg, J.D.Kennedy, S.L.Shea	Leeds	C28 H58 B18 P2 Pt1 Ru1,2(C1 H2 Cl2)
2163002	SAXNUE	10.5517/ccdc.csd.cc2bls80	W.Clegg, J.D.Kennedy, S.L.Shea	Leeds	C22 H48 B18 Ir1 Ru1,1.5(C1 H2 Cl2)
2163003	SAXPAM	10.5517/ccdc.csd.cc2bls91	W.Clegg, J.D.Kennedy, A.R.HamiltonMcleod	Leeds	C18 H33 B16 O1 P1 S2 1-
2164794	XECDUI	10.5517/ccdc.csd.cc2bnn2r	W.Clegg, J.D.Kennedy, M.J.Carr	Leeds	H21 B18 1-,C14 H19 N2 1+
2165633	KAYNAD	10.5517/ccdc.csd.cc2bpj4q	W.Clegg, J.D.Kennedy, A.Brownless, P.A.Cooke	Leeds	C16 H46 B20 P2 Pt1,0.5(C1 H2 Cl2)
2165634	KAYNEH	10.5517/ccdc.csd.cc2bpj5r	W.Clegg, J.D.Kennedy, J.Bould	Leeds	H20 B18 I1 1-,C14 H19 N2 1+
2165635	KAYNIL	10.5517/ccdc.csd.cc2bpj6s	W.Clegg, J.D.Kennedy, J.Bould	Leeds	C54 H63 B18 P3 Pd1,C1 H1 Cl3
2165636	KAYNOR	10.5517/ccdc.csd.cc2bpj7t	W.Clegg, J.D.Kennedy, R.D.Kennedy	Leeds	C5 H25 B9 N2
2165637	KAYNUX	10.5517/ccdc.csd.cc2bpj8v	W.Clegg, J.D.Kennedy, M.J.Carr, S.D.Perera	Leeds	C43 H46 B9 P2 Ru1
2165638	KAYPAF	10.5517/ccdc.csd.cc2bpj9w	W.Clegg, J.D.Kennedy, R.D.Kennedy	Leeds	C8 H20 N1 1+,H3 B1 O3,H2 O1,Cl1 1-
2165641	KAYPEJ	10.5517/ccdc.csd.cc2bpjdz	W.Clegg, J.D.Kennedy, M.G.S.Londesborough	Leeds	C6 H36 B12 O6 P2 Pd2
2165643	KAYPOT	10.5517/ccdc.csd.cc2bpjg1	W.Clegg, J.D.Kennedy, M.G.S.Londesborough	Leeds	C36 H54 B20 P2 Pd1,0.75(C1 H2 Cl2)
2165644	KAYPUZ	10.5517/ccdc.csd.cc2bpjh2	W.Clegg, J.D.Kennedy, T.Jelinek, P.A.Cooke	Leeds	C24 H20 P1 1+,H19 B18 I2 1-
2165645	KAYPUZ01	10.5517/ccdc.csd.cc2bpjj3	W.Clegg, J.D.Kennedy, T.Jelinek, P.A.Cooke	Leeds	C24 H20 P1 1+,H19 B18 I2 1-
2169299	DOXADM03	10.5517/ccdc.csd.cc2btbdx	W.Clegg, R.W.Harrington	Newcastle	C10 H13 N5 O3,H2 O1
2169300	DIDPPD03	10.5517/ccdc.csd.cc2btbfy	W.Clegg, R.W.Harrington	Newcastle	C36 H30 I2 P2 Pd1,2(C1 H2 Cl2)
2169301	NAACET07	10.5517/ccdc.csd.cc2btbgz	W.Clegg, R.W.Harrington	Newcastle	(C4 H18 Na2 O10)n
2169587	BONLEV03	10.5517/ccdc.csd.cc2btmph	W.Clegg, J.D.Kennedy, M.J.Carr, R.D.Kennedy	Leeds	C18 H18 B1 P1
2169588	TEGKOJ	10.5517/ccdc.csd.cc2btmqj	W.Clegg, J.D.Kennedy, M.J.Carr	Leeds	C27 H33 Mo1 O3 P3
2174366	WEKBAT	10.5517/ccdc.csd.cc2bzlvs	W.Clegg, R.L.Gill	Newcastle	(C10 H12 N4 Ni1 O8)n
2174368	WEKBEX	10.5517/ccdc.csd.cc2bzlxv	W.Clegg, R.L.Gill	Newcastle	(C14 H13 Cl1 Co1 N2 O5 S1)n,C2 H6 O1 S1
2174370	AMUCET01	10.5517/ccdc.csd.cc2bzlzx	W.Clegg, R.L.Gill	Newcastle	(C36 H26 Eu2 N6 O16)n
2174371	WEKBOH	10.5517/ccdc.csd.cc2bzm0z	W.Clegg, R.L.Gill	Newcastle	(C14 H16 Eu1 N1 O8 S1)n
2174372	WEKBUN	10.5517/ccdc.csd.cc2bzm10	W.Clegg, R.L.Gill	Newcastle	(C42 H36 Eu2 N8 O16)n,2(C3 H7 N1 O1)
2174373	WEKCAU	10.5517/ccdc.csd.cc2bzm21	W.Clegg, R.L.Gill	Newcastle	(C34 H24 N4 O10 S2 Zn2)n
2174375	WEKCEY	10.5517/ccdc.csd.cc2bzm43	W.Clegg, R.L.Gill	Newcastle	C14 H26 Co2 N6 O10 S2
2174383	WEKCIC	10.5517/ccdc.csd.cc2bzmdc	W.Clegg, J.D.Kennedy, M.J.Carr	Leeds	C20 H49 B17 Ir2
2174384	WEKCOI	10.5517/ccdc.csd.cc2bzmfd	W.Clegg, J.D.Kennedy, M.J.Carr, S.D.Perera	Leeds	C5 H25 B18 Ru1 1-,C14 H19 N2 1+
2174385	WEKCUO	10.5517/ccdc.csd.cc2bzmgf	W.Clegg, J.D.Kennedy, S.L.Shea	Leeds	C12 H38 B18 Ru1
2174389	WEKDAV	10.5517/ccdc.csd.cc2bzmlk	W.Clegg, J.D.Kennedy, S.L.Shea	Leeds	C6 H27 B17 Ru1

2174390	WEKDEZ	10.5517/ccdc.csd.cc2bzmml	W.Clegg, J.D.Kennedy, S.L.Shea	Leeds	C6 H26 B18 Ru1
2174415	WEKJOP	10.5517/ccdc.csd.cc2bznff	W.Clegg, J.D.Kennedy, S.L.Shea	Leeds	C18 H45 B18 P1 Rh1
2174420	WEKJUV	10.5517/ccdc.csd.cc2bznll	W.Clegg, J.D.Kennedy, M.J.Carr	Leeds	(C14 H22 Ag1 B11)n
2174470	AYOZET01	10.5517/ccdc.csd.cc2bzq68	W.Clegg, J.D.Kennedy, S.L.Shea	Leeds	C13 H43 B18 Ir1 N2
2174756	MAZRAK	10.5517/ccdc.csd.cc2c00fv	W.Clegg, R.L.Gill	Newcastle	(C14 H13 Cl1 Co1 N2 O5 S1)n
2174757	MAZREO	10.5517/ccdc.csd.cc2c00gw	W.Clegg, R.L.Gill	Newcastle	(C33 H39 Co2 N5 O13 S2)n
2174758	XEBDEO03	10.5517/ccdc.csd.cc2c00hx	W.Clegg, R.L.Gill	Newcastle	C14 H10 O4 S2,2(C3 H7 N1 O1)
2175463	MEJMOH	10.5517/ccdc.csd.cc2c0r7d	W.Clegg, R.L.Gill	Newcastle	(C11 H18 Eu1 N1 O12 S1)n,C3 H7 N1 O1,H2 O1
2175464	MEJMUN	10.5517/ccdc.csd.cc2c0r8f	W.Clegg, R.L.Gill	Newcastle	(C14 H8 Cu1 N2 O8)n,2(C3 H7 N1 O1)
2175465	MEJNAU	10.5517/ccdc.csd.cc2c0r9g	W.Clegg, R.L.Gill	Newcastle	(C68 H78 N10 O26 S4 Zn4)n
2175584	MEJRIG	10.5517/ccdc.csd.cc2c0w4f	W.Clegg, R.L.Gill	Newcastle	(C8 H13 Eu1 O12 S1)n,3(H2 O1)
2175586	MEJROM	10.5517/ccdc.csd.cc2c0w6h	W.Clegg, R.L.Gill	Newcastle	(C21 H14 N1 O14 Zn3 1-)n,H3 O1 1+
2175758	SUCROS55	10.5517/ccdc.csd.cc2c11r7	W.Clegg, R.L.Gill	Newcastle	C12 H22 O11
2176067	QAZDII	10.5517/ccdc.csd.cc2c1cqj	W.Clegg, R.L.Gill	Newcastle	C20 H22 Co1 N4 O10,2(C3 H7 N1 O1)
2176068	QAZDOO	10.5517/ccdc.csd.cc2c1crk	W.Clegg, R.L.Gill	Newcastle	(C36 H26 N6 O16 Tb2)n
2176070	QUCDIC02	10.5517/ccdc.csd.cc2c1ctm	W.Clegg, R.L.Gill	Newcastle	C12 H14 N2 Ni1 O8,2(H2 O1)
2176071	QAZFEG	10.5517/ccdc.csd.cc2c1cvn	W.Clegg, R.L.Gill	Newcastle	(C27 H9 O18 Zn3 3-)n,3n(C2 H8 N1 1+)
2176575	QEDTOM	10.5517/ccdc.csd.cc2c1x3g	W.Clegg, R.L.Gill	Newcastle	(C51 H74 N9 O31 S3 Tb3)n,C3 H7 N1 O1,3(H2 O1)
2176576	QEDTUS	10.5517/ccdc.csd.cc2c1x4h	W.Clegg, R.L.Gill	Newcastle	(C33 H39 N5 O13 S2 Zn2)n
2176578	AZNPYD01	10.5517/ccdc.csd.cc2c1x6k	W.Clegg, R.L.Gill	Newcastle	(C6 H6 N2 O6 Zn1)n,n(H2 O1)
2177551	YAZDEM	10.5517/ccdc.csd.cc2c2xlz	W.Clegg, R.L.Gill	Newcastle	(C11 H18 N1 O12 S1 Tb1)n,C3 H7 N1 O1,H2 O1
2177552	ACIBEU02	10.5517/ccdc.csd.cc2c2xm0	W.Clegg, R.L.Gill	Newcastle	(C21 H27 Co3 N3 O15)n
2180578	CEKNOZ	10.5517/ccdc.csd.cc2c627x	W.Clegg, R.L.Gill	Newcastle	(C21 H17 N1 O16 Zn3 2-)n,2(C4 H12 N1 1+)
2180579	OKOWOC02	10.5517/ccdc.csd.cc2c628y	W.Clegg, R.L.Gill	Newcastle	2(C6 H11 N2 1+),Br4 Cd1 2-
2180580	CEKPAN	10.5517/ccdc.csd.cc2c629z	W.Clegg, R.L.Gill	Newcastle	(C34 H52 N6 O22 S2 Tb2)n,C3 H7 N1 O1,2.5(H2 O1)
2180581	CEKPER	10.5517/ccdc.csd.cc2c62b0	W.Clegg, R.L.Gill	Newcastle	(C34 H24 N4 O10 S2 Zn2)n
2180801	ACIHUT	10.5517/ccdc.csd.cc2c69fb	W.Clegg, R.L.Gill	Newcastle	(C54 H79 Eu3 N10 O31 S3)n,C3 H7 N1 O1
2181725	ADUBOU	10.5517/ccdc.csd.cc2c7874	W.Clegg, P.A.Champkin	UK	C24 H46 P2 2+,2(C1 H3 O3 S1 1-)
2181726	ADUBUA	10.5517/ccdc.csd.cc2c7885	W.Clegg, P.A.Champkin	Newcastle	C27 H26 Cl2 P2 Pd1,C1 H2 Cl2
2181727	LAJKEO03	10.5517/ccdc.csd.cc2c7896	W.Clegg, P.A.Champkin	Newcastle	C24 H44 Cl2 P2 Pd1
2181728	EWIBAQ01	10.5517/ccdc.csd.cc2c78b7	W.Clegg, P.A.Champkin	Newcastle	C26 H46 O2 P2 Pd1,C2 H4 O2
2181730	EWIBEU01	10.5517/ccdc.csd.cc2c78d9	W.Clegg, P.A.Champkin	Newcastle	C49 H42 O1 P2 Pd1
2181731	HEZJUS01	10.5517/ccdc.csd.cc2c78fb	W.Clegg, P.A.Champkin	Newcastle	C32 H28 O2 P2
2181733	ADUPIC	10.5517/ccdc.csd.cc2c78hd	W.Clegg, P.A.Champkin	Newcastle	C32 H28 P2
2181891	DECWUH	10.5517/ccdc.csd.cc2c7fln	W.Clegg, R.W.Harrington	Newcastle	C7 H9 N4 O1 1+,I3 1-
2181892	DECXAO	10.5517/ccdc.csd.cc2c7fmp	W.Clegg, R.W.Harrington	Newcastle	C30 H51 Fe11 O42,2(C7 H8 N4 S1)
2181893	WUKJER02	10.5517/ccdc.csd.cc2c7fnq	W.Clegg, R.W.Harrington	Newcastle	C7 H8 N4 S1
2182343	CEKSEU	10.5517/ccdc.csd.cc2c7x5q	W.Clegg, R.W.Harrington	Newcastle	(C60 H48 N20 Ni4 O24)n
2182344	CEKSIY	10.5517/ccdc.csd.cc2c7x6r	W.Clegg, R.W.Harrington	Newcastle	(C33 H31 N5 O9 Zn2)n
2182345	CEKSOE	10.5517/ccdc.csd.cc2c7x7s	W.Clegg, R.W.Harrington	Newcastle	(C42 H34 Cu2 N4 O12)n
2182346	CEKSUK	10.5517/ccdc.csd.cc2c7x8t	W.Clegg, R.W.Harrington	Newcastle	(C62 H68 Co3 N6 O14)n
2182347	CEKTAR	10.5517/ccdc.csd.cc2c7x9v	W.Clegg, R.W.Harrington	Newcastle	C24 H28 Cu1 N6 O6

2182348	CEKTEV	10.5517/ccdc.csd.cc2c7xbw	W.Clegg, R.W.Harrington	Newcastle	(C6 H8 N2 Ni1 O7)n,H2 O1
2182350	CEKTIZ	10.5517/ccdc.csd.cc2c7xdy	W.Clegg, R.W.Harrington	Newcastle	(C6 H6 N2 O6 Zn1)n,2(H2 O1)
2182352	UGURET02	10.5517/ccdc.csd.cc2c7xg0	W.Clegg, R.W.Harrington	Newcastle	C10 H4 O8 2-,H12 O6 Zn1 2+
2182353	CEKTUL	10.5517/ccdc.csd.cc2c7xh1	W.Clegg, R.W.Harrington	Newcastle	(C8 H24 N4 O8 Zn2 4+)n,4(N1 O3 1-)
2182354	CEKVAT	10.5517/ccdc.csd.cc2c7xj2	W.Clegg, R.W.Harrington	Newcastle	C26 H32 Co1 N4 O4 2+,2(C12 H10 N2),2(N1 O3 1-)
2182597	LIXWAR03	10.5517/ccdc.csd.cc2c85c6	W.Clegg, R.W.Harrington	Newcastle	(C48 H46 Mn3 N4 O16)n
2182598	LEVNOQ02	10.5517/ccdc.csd.cc2c85d7	W.Clegg, R.W.Harrington	Newcastle	(C48 H46 Mg3 N4 O16)n
2182599	DAXNOG04	10.5517/ccdc.csd.cc2c85f8	W.Clegg, R.W.Harrington	Newcastle	(C22 H22 N2 O10 Zn2)n
2182600	TOKPAK04	10.5517/ccdc.csd.cc2c85g9	W.Clegg, R.W.Harrington	Newcastle	(C10 H12 Cl2 Cu1 N2 O10)n,C10 H8 N2
2182601	MIMRAB02	10.5517/ccdc.csd.cc2c85hb	W.Clegg, R.W.Harrington	Newcastle	(C12 H8 O5 Zn1)n
2182884	CELBUU	10.5517/ccdc.csd.cc2c8gmr	W.Clegg, R.W.Harrington	Newcastle	(C10 H12 N4 Ni1 O8)n
2182885	NOCLAT01	10.5517/ccdc.csd.cc2c8gns	W.Clegg, R.W.Harrington	Newcastle	(C15 H13 Ca1 N1 O5)n
2182886	SUJQUK01	10.5517/ccdc.csd.cc2c8gpt	W.Clegg, R.W.Harrington	Newcastle	(C20 H14 N2 O4 Zn1)n,C3 H7 N1 O1
2182888	EZULAO02	10.5517/ccdc.csd.cc2c8grw	W.Clegg, R.W.Harrington	Newcastle	(C30 H26 Cd2 N2 O10)n
2182889	XIHSEN02	10.5517/ccdc.csd.cc2c8gsx	W.Clegg, R.W.Harrington	Newcastle	(C48 H46 Co3 N4 O16)n
2182890	CEJKAH	10.5517/ccdc.csd.cc2c8gty	W.Clegg, R.W.Harrington	Newcastle	(C12 H12 Mg6 O24)n,2(C4 H8 O2)
2182892	YOWNAC01	10.5517/ccdc.csd.cc2c8gw0	W.Clegg, R.W.Harrington	Newcastle	(C52 H54 Mg3 N4 O16)n
2182893	CIFDUS01	10.5517/ccdc.csd.cc2c8gx1	W.Clegg, R.W.Harrington	Newcastle	(C52 H54 Mn3 N4 O16)n
2182894	AGORUK01	10.5517/ccdc.csd.cc2c8gy2	W.Clegg, R.W.Harrington	Newcastle	(C4 H6 Cu1 N2 O5 S1)n
2182895	MACUFR31	10.5517/ccdc.csd.cc2c8gz3	W.Clegg, R.W.Harrington	Newcastle	(C3 H3 Cu1 O6 1-)n,C2 H8 N1 1+
2182896	CEJLEM	10.5517/ccdc.csd.cc2c8h05	W.Clegg, R.W.Harrington	Newcastle	(C6 H10 O6 Zn1)n
2182900	DADTUY15	10.5517/ccdc.csd.cc2c8h49	W.Clegg, R.W.Harrington	Newcastle	(C3 H3 Co1 O6 1-)n,C2 H8 N1 1+
2183128	CEJPIU	10.5517/ccdc.csd.cc2c8qhw	W.Clegg	UK	C6 H18 N3 O1 P1,H4 N2 O2 S1,H2 O1
2183129	CEJPOA	10.5517/ccdc.csd.cc2c8qjx	W.Clegg, S.L.Heath	UK	C34 H32 N3 O6 Zn1 1+,C24 H20 B1 1-
2183130	DELKIS	10.5517/ccdc.csd.cc2c8qky	W.Clegg, S.L.Heath	UK	C34 H32 Co1 N3 O6 1+,C24 H20 B1 1-,C1 H2 Cl2
2183131	DELKOY	10.5517/ccdc.csd.cc2c8qlz	W.Clegg, S.L.Heath	UK	C22 H27 N4 O4 Zn1 1+,C24 H20 B1 1-,C1 H4 O1
2183183	DEKMOV01	10.5517/ccdc.csd.cc2c8s8q	W.Clegg, L.Russo	AB75	C9 H15 Cl1 Cu1 N3 S6
2183184	TTPCUC06	10.5517/ccdc.csd.cc2c8s9r	W.Clegg, L.Russo	AB78	C54 H45 Cl1 Cu1 P3
2183185	TBUAWO10	10.5517/ccdc.csd.cc2c8sbs	W.Clegg, L.Russo	AB80	2(C16 H36 N1 1+),O19 W6 2-
2183186	CARQOB01	10.5517/ccdc.csd.cc2c8sct	W.Clegg, L.Russo	AB87	6(C8 H20 N1 1+),Ag6 I11 5-,I1 1-
2183193	NOGPAB01	10.5517/ccdc.csd.cc2c8sl1	W.Clegg, L.Russo	AB90	C16 H24 Cl2 Cu2 N8 S4
2183194	MIWFOO02	10.5517/ccdc.csd.cc2c8sm2	W.Clegg, L.Russo	AB91	C3 H8 N6 2+,O4 S1 2-
2183339	SORLAM02	10.5517/ccdc.csd.cc2c8y9x	W.Clegg, L.Russo	AB84	2(C16 H36 N1 1+),Cl4 Cu4 Mo1 S4 2-
2183426	VUNVOQ01	10.5517/ccdc.csd.cc2c913v	W.Clegg, R.W.Harrington	Newcastle	C24 H19 Au1 Cl1 P1
2183429	CEJCIH	10.5517/ccdc.csd.cc2c916y	W.Clegg, R.W.Harrington	Newcastle	C45 H41 Cl2 O1 P1 Ru1,0.5(C1 H2 Cl2)
2183430	CEJCON	10.5517/ccdc.csd.cc2c917z	W.Clegg, R.W.Harrington	Newcastle	C58 H44 Au2 Cl2 P2,4(C1 H1 Cl3)
2183431	DEMGUB	10.5517/ccdc.csd.cc2c9180	W.Clegg, R.W.Harrington	Newcastle	C56 H40 Au2 Cl2 P2,2(C1 H2 Cl2)
2183432	DEMHAI	10.5517/ccdc.csd.cc2c9191	W.Clegg, R.W.Harrington	Newcastle	C58 H44 Au2 Cl2 O2 P2,2(C1 H2 Cl2)
2183434	DEMHEM	10.5517/ccdc.csd.cc2c91c3	W.Clegg, R.W.Harrington	Newcastle	C24 H24 Au1 Cl1 O9 P1 S3 3-,C4 H10 O1,3(C1 H4 O1),3(Na1 1+)
2183435	DEMHIQ	10.5517/ccdc.csd.cc2c91d4	W.Clegg, R.W.Harrington	Newcastle	C28 H35 Au1 Cl1 P1
2183436	DEMHOW	10.5517/ccdc.csd.cc2c91f5	W.Clegg, R.W.Harrington	Newcastle	C9 H7 N3 O5 S1
2183437	CEJCUT	10.5517/ccdc.csd.cc2c91g6	W.Clegg, R.W.Harrington	Newcastle	C58 H44 O2 P2

2183438	CEJDAA	10.5517/ccdc.csd.cc2c91h7	W.Clegg, R.W.Harrington	Newcastle	C19 H23 N2 1+,Br1 1-
2183439	RECMOF	10.5517/ccdc.csd.cc2c91j8	W.Clegg, R.W.Harrington	Newcastle	C35 H39 Au1 Cl1 P1
2183610	RECPEY	10.5517/ccdc.csd.cc2c971z	W.Clegg, R.W.Harrington	Newcastle	C16 H18 N4 O2 S2
2183611	RECPIC	10.5517/ccdc.csd.cc2c9720	W.Clegg, R.W.Harrington	Newcastle	C48 H37 N5
2183612	CEJDEE	10.5517/ccdc.csd.cc2c9731	W.Clegg, R.W.Harrington	Newcastle	C27 H23 B1 F2 N2
2183613	SIYWUV01	10.5517/ccdc.csd.cc2c9742	W.Clegg, R.W.Harrington	Newcastle	C17 H16 B1 F2 N3
2183614	CEJDOO	10.5517/ccdc.csd.cc2c9753	W.Clegg, R.W.Harrington	Newcastle	C24 H16 B1 F2 N5
2183615	CEJDUU	10.5517/ccdc.csd.cc2c9764	W.Clegg, R.W.Harrington	Newcastle	C29 H28 B1 F5 N5 O5 S1 Zn1 1+,C1 F3 O3 S1 1-,H2 O1
2183616	CEJFAC	10.5517/ccdc.csd.cc2c9775	W.Clegg, R.W.Harrington	Newcastle	C17 H10 O2
2183617	CEJFEG	10.5517/ccdc.csd.cc2c9786	W.Clegg, R.W.Harrington	Newcastle	C12 H9 Br1 O2
2183618	CEJFIK	10.5517/ccdc.csd.cc2c9797	W.Clegg, R.W.Harrington	Newcastle	C26 H21 Br1 O2
2183619	COCHEJ05	10.5517/ccdc.csd.cc2c97b8	W.Clegg, R.W.Harrington	Newcastle	C19 H19 B1 F2 N2
2183620	CEJFUW	10.5517/ccdc.csd.cc2c97c9	W.Clegg, R.W.Harrington	Newcastle	C14 H10 F5 N1 O2
2183621	CEJGAD	10.5517/ccdc.csd.cc2c97db	W.Clegg, R.W.Harrington	Newcastle	C17 H15 N1 O5
2184072	REFQEC	10.5517/ccdc.csd.cc2c9pyb	W.Clegg, R.W.Harrington	Newcastle	C33 H32 B1 F8 N5 O7 S2 Zn1
2184078	COMGOA01	10.5517/ccdc.csd.cc2c9q4k	W.Clegg, R.W.Harrington	Newcastle	C17 H15 N1 O2
2184079	ZZZAWP11	10.5517/ccdc.csd.cc2c9q5l	W.Clegg, R.W.Harrington	Newcastle	C16 H14 N2 O2
2184081	KUSPUK01	10.5517/ccdc.csd.cc2c9q7n	W.Clegg, R.W.Harrington	Newcastle	C19 H14 B1 F7 N2
2184082	REHTIL	10.5517/ccdc.csd.cc2c9q8p	W.Clegg, R.W.Harrington	Newcastle	C22 H24 B1 F2 N5 O1
2184084	REHTOR	10.5517/ccdc.csd.cc2c9qbr	W.Clegg	Newcastle	C24 H20 N2 S1 2+,2(F6 P1 1-)
2184085	REHTUX	10.5517/ccdc.csd.cc2c9qcs	W.Clegg, R.W.Harrington	Newcastle	C16 H10 Cl4 N2 O2
2184086	REHVAF	10.5517/ccdc.csd.cc2c9qdt	W.Clegg, R.W.Harrington	Newcastle	C27 H22 N2 O1
2184093	REGCIT	10.5517/ccdc.csd.cc2c9qm1	W.Clegg, R.W.Harrington	Newcastle	C20 H16 N2 O3
2184096	REGCOZ	10.5517/ccdc.csd.cc2c9qq4	W.Clegg, R.W.Harrington	Newcastle	C26 H20 N2 O2
2184525	CEJJEK	10.5517/ccdc.csd.cc2cb5kg	W.Clegg, R.W.Harrington	Newcastle	C35 H34 B1 F2 N3
2184526	CEJJIO	10.5517/ccdc.csd.cc2cb5lh	W.Clegg, R.W.Harrington	Newcastle	C14 H20 N6 O2,2(H2 O1)
2184527	CEJSAP	10.5517/ccdc.csd.cc2cb5mj	W.Clegg, R.W.Harrington	Newcastle	C28 H18 O3
2184528	CEJSET	10.5517/ccdc.csd.cc2cb5nk	W.Clegg, R.W.Harrington	Newcastle	C27 H19 B1 F2 N2
2184529	CEJSIX	10.5517/ccdc.csd.cc2cb5pl	W.Clegg, R.W.Harrington	Newcastle	C22 H24 N6 O2
2184530	TIGXEN01	10.5517/ccdc.csd.cc2cb5qm	W.Clegg, R.W.Harrington	Newcastle	C14 H5 Cl4 N1 O2
2184532	CEJSUJ	10.5517/ccdc.csd.cc2cb5s	W.Clegg, R.W.Harrington	Newcastle	C27 H29 B1 F2 N2
2184988	CEJYOJ	10.5517/ccdc.csd.cc2cbnhw	W.Clegg, R.W.Harrington	Newcastle	C20 H20 N6 O2,4(H2 O1)
2185030	CEJYUP	10.5517/ccdc.csd.cc2cbpv8	W.Clegg, R.W.Harrington	Newcastle	C40 H30 Mn3 N2 O20
2185046	CEJZAW	10.5517/ccdc.csd.cc2cbqct	W.Clegg, R.W.Harrington	Newcastle	C35 H27 N7 O5 Zn1,2(C5 H5 N1)
2185077	CEJZEA	10.5517/ccdc.csd.cc2cbrcv	W.Clegg, R.W.Harrington	Newcastle	C27 H17 Br1 O1
2189153	CEKBED	10.5517/ccdc.csd.cc2cgzvp	W.Clegg, R.W.Harrington	Newcastle	C27 H16 Br1 O1 1+,B1 F4 1-
2189155	CEKBIH	10.5517/ccdc.csd.cc2cgzxr	W.Clegg, R.W.Harrington	Newcastle	C18 H16 Br1 N1
2189156	CEKBON	10.5517/ccdc.csd.cc2cgzys	W.Clegg, R.W.Harrington	Newcastle	C27 H31 B1 F2 N2 O4
2189157	CEKBUT	10.5517/ccdc.csd.cc2cgzzt	W.Clegg, R.W.Harrington	Newcastle	C32 H23 O5 1+,B1 F4 1-
2189158	CEKCAA	10.5517/ccdc.csd.cc2ch00x	W.Clegg, R.W.Harrington	Newcastle	C12 H14 I1 N1 O3
2189163	CEKCEE	10.5517/ccdc.csd.cc2ch052	W.Clegg, R.W.Harrington	Newcastle	C27 H12 Br1 O1 1+,C2 H3 N1,B1 F4 1-
2189164	CEKCII	10.5517/ccdc.csd.cc2ch063	W.Clegg, R.W.Harrington	Newcastle	C36 H27 Br1 N1 O3 1+,F6 P1 1-

2189165	VAWDED01	10.5517/ccdc.csd.cc2ch074	W.Clegg, R.W.Harrington	Newcastle	C15 H11 B1 F2 N2
2189166	CEKCUU	10.5517/ccdc.csd.cc2ch085	W.Clegg, R.W.Harrington	Newcastle	C24 H20 Br2 O8
2189700	CEMDOR	10.5517/ccdc.csd.cc2chkhy	W.Clegg, R.W.Harrington	Newcastle	C20 H15 Cl1 N2 O2
2189701	CEMDUX	10.5517/ccdc.csd.cc2chkjz	W.Clegg, R.W.Harrington	Newcastle	C34 H42 O8
2189702	CEMFAF	10.5517/ccdc.csd.cc2chkk0	W.Clegg, R.W.Harrington	Newcastle	C30 H34 O8
2189703	CEMFEJ	10.5517/ccdc.csd.cc2chkl1	W.Clegg, R.W.Harrington	Newcastle	C34 H42 O8
2189704	CEMFIN	10.5517/ccdc.csd.cc2chkm2	W.Clegg, R.W.Harrington	Newcastle	C24 H20 I2 O8
2189707	CEMFOT	10.5517/ccdc.csd.cc2chkq5	W.Clegg, R.W.Harrington	Newcastle	C13 H12 N2 O3
2189708	RIWTOG01	10.5517/ccdc.csd.cc2chkr6	W.Clegg, R.W.Harrington	Newcastle	C7 H5 I1 O1
2189709	CEMGAG	10.5517/ccdc.csd.cc2chks7	W.Clegg, R.W.Harrington	Newcastle	C14 H14 Br1 N1 O1
2189710	LOBDAJ02	10.5517/ccdc.csd.cc2chkt8	W.Clegg, R.W.Harrington	Newcastle	C14 H20 O11,H2 O1
2189711	CEMGIO	10.5517/ccdc.csd.cc2chkv9	W.Clegg, R.W.Harrington	Newcastle	C16 H22 O12,H2 O1
2190587	YENRIV01	10.5517/ccdc.csd.cc2cjh3j	W.Clegg, R.W.Harrington	Newcastle	C19 H18 B1 F3 N2
2190588	EDATUC	10.5517/ccdc.csd.cc2cjh4k	W.Clegg, R.W.Harrington	Newcastle	C32 H38 O8
2190594	EDAVAK	10.5517/ccdc.csd.cc2cjhbr	W.Clegg, R.W.Harrington	Newcastle	C36 H23 Br1 N1 O3 1+,F6 P1 1-,H2 O1
2190595	EDAVEO	10.5517/ccdc.csd.cc2cjhcs	W.Clegg	Newcastle	C19 H12 B1 F7 I2 N2
2190596	UJOJEI01	10.5517/ccdc.csd.cc2cjhdt	W.Clegg, R.W.Harrington	Newcastle	(C16 H18 Cl16 Mn4 O22)n
2190597	EDAXAM	10.5517/ccdc.csd.cc2cjhfv	W.Clegg, R.W.Harrington	Newcastle	(C4 H6 Ca1 Cl4 O6)n,C9 H11 N1 O3
2191049	EDUQIH	10.5517/ccdc.csd.cc2cjz0x	W.Clegg, R.W.Harrington	Newcastle	(C42 H30 Co4 N8 O18)n
2191051	EDUSAB	10.5517/ccdc.csd.cc2cjz2z	W.Clegg, R.W.Harrington	Newcastle	C20 H15 Br1 N2 O2
2191052	EDUSEF	10.5517/ccdc.csd.cc2cjz30	W.Clegg, R.W.Harrington	Newcastle	C46 H36 Cl4 Mn6 N6 O20,C4 H10 O1,5(H2 O1)
2191053	EDUSIJ	10.5517/ccdc.csd.cc2cjz41	W.Clegg, R.W.Harrington	Newcastle	(C20 H21 Cl2 Mn2 N3 O6 1+)n,Cl1 O4 1-
2191054	EDUSOP	10.5517/ccdc.csd.cc2cjz52	W.Clegg, R.W.Harrington	Newcastle	C46 H42 Cl2 Mn6 N6 O22 6+,6(N1 O3 1-)
2191055	EDUSUV	10.5517/ccdc.csd.cc2cjz63	W.Clegg, R.W.Harrington	Newcastle	(C21 H19 Mn1 N5 O5)n
2191056	EDUTAC	10.5517/ccdc.csd.cc2cjz74	W.Clegg, R.W.Harrington	Newcastle	C22 H19 Cl2 Mn1 N4 O3 1+,Cl1 O4 1-
2194406	BEQCUZ	10.5517/ccdc.csd.cc2cng9t	W.Clegg, R.W.Harrington	Newcastle	C25 H31 N1 O6 S1
2194407	BEQDAG	10.5517/ccdc.csd.cc2cngbv	W.Clegg, R.W.Harrington	Newcastle	C31 H36 N2 O5 S1
2194408	BEQDEK	10.5517/ccdc.csd.cc2cngcw	W.Clegg, R.W.Harrington	Newcastle	C29 H34 N4 O5 S1
2194409	BEQDIO	10.5517/ccdc.csd.cc2cngdx	W.Clegg, R.W.Harrington	Newcastle	C29 H34 N4 O5 S1
2194410	BEQDOU	10.5517/ccdc.csd.cc2cngfy	W.Clegg, R.W.Harrington	Newcastle	C28 H23 Au1 Cl1 P1,2(C1 H1 Cl3)
2194411	BEQDUA	10.5517/ccdc.csd.cc2cnggz	W.Clegg, R.W.Harrington	Newcastle	C33 H24 N1 O1 P1
2194413	OLEKUN01	10.5517/ccdc.csd.cc2cngj1	W.Clegg, R.W.Harrington	Newcastle	C28 H21 Au1 Cl1 P1,C1 H1 Cl3
2194414	BEQFEM	10.5517/ccdc.csd.cc2cngk2	W.Clegg, R.W.Harrington	Newcastle	C62 H45 Cl1 N1 P1 Pd1
2194415	BEQFIQ	10.5517/ccdc.csd.cc2cngl3	W.Clegg, R.W.Harrington	Newcastle	C16 H11 B1 Br2 F2 N2
2194505	OYULIF02	10.5517/ccdc.csd.cc2cnkh3	W.Clegg, R.W.Harrington	Newcastle	C19 H17 B1 Br2 F2 N2
2204927	KEPDES	10.5517/ccdc.csd.cc2d0dpj	W.Clegg, R.W.Harrington	Newcastle	C15 H14 I1 O2 1+,C2 F3 O2 1-
2204928	KEPGUL	10.5517/ccdc.csd.cc2d0dqk	W.Clegg, R.W.Harrington	Newcastle	C15 H16 I1 O1 1+,C7 H7 O3 S1 1-
2204929	KEPHAS	10.5517/ccdc.csd.cc2d0drl	W.Clegg, R.W.Harrington	Newcastle	C13 H12 I1 O1 1+,C7 H7 O3 S1 1-
2204932	KEPHEW	10.5517/ccdc.csd.cc2d0dvp	W.Clegg, R.W.Harrington	Newcastle	C14 H14 I1 O1 1+,C7 H7 O3 S1 1-,H2 O1
2204933	KEPHIA	10.5517/ccdc.csd.cc2d0dwq	W.Clegg, R.W.Harrington	Newcastle	C13 H11 F1 O1
2204934	KEPHOG	10.5517/ccdc.csd.cc2d0dxr	W.Clegg, R.W.Harrington	Newcastle	C13 H11 I1 O1
2204938	KEPHUM	10.5517/ccdc.csd.cc2d0f1x	W.Clegg, R.W.Harrington	Newcastle	C16 H16 I1 O3 1+,C2 F3 O2 1-

2204939	KEPJAU	10.5517/ccdc.csd.cc2d0f2y	W.Clegg, R.W.Harrington	Newcastle	C11 H8 Br1 N1 O4
2204999	NEZVIZ01	10.5517/ccdc.csd.cc2d0h0y	W.Clegg, R.W.Harrington	Newcastle	C17 H12 F3 I1 O3
2206344	TUKRUP01	10.5517/ccdc.csd.cc2d1wdr	W.Clegg, R.W.Harrington	Newcastle	C13 H12 I1 O2 S1 1+,C2 F3 O2 1-
2206345	NUMLAL01	10.5517/ccdc.csd.cc2d1wfs	W.Clegg, R.W.Harrington	Newcastle	C15 H14 I1 O2 1+,C2 F3 O2 1-
2206346	GEQMUO	10.5517/ccdc.csd.cc2d1wgt	W.Clegg, R.W.Harrington	Newcastle	C17 H14 I1 O1 S1 1+,C2 F3 O2 1-
2206347	GEQNAV	10.5517/ccdc.csd.cc2d1whv	W.Clegg, R.W.Harrington	Newcastle	C20 H18 I1 O2 1+,C2 F3 O2 1-
2206348	GEQNEZ	10.5517/ccdc.csd.cc2d1wjw	W.Clegg	Newcastle	C11 H13 I1 O5,0.12(H2 O1)
2206350	GEQMUO01	10.5517/ccdc.csd.cc2d1wly	W.Clegg, R.W.Harrington	Newcastle	C17 H14 I1 O1 S1 1+,C2 F3 O2 1-
2217542	DEQFAK	10.5517/ccdc.csd.cc2dfjm0	W.Clegg, J.D.Kennedy, T.R.Spalding, Faridoon	Leeds	C23 H36 B10 Cu1 O1 P1 Se1
2217543	DEQFEO	10.5517/ccdc.csd.cc2dfjn1	W.Clegg, J.D.Kennedy, S.L.Shea	Leeds	C20 H47 B18 Cl1 Ir2
2217546	DEQFIS	10.5517/ccdc.csd.cc2dfjr4	W.Clegg, J.D.Kennedy, P.K.Dosangh, J.Bould, T.Jelinek, N.P.Rath	Leeds	C16 H38 B20 P2
2217547	DEQFOY	10.5517/ccdc.csd.cc2dfjs5	W.Clegg, J.D.Kennedy, M.J.Carr	Leeds	C7 H9 B9 I5 1-,Ag1 1+
2217548	DEQFUE	10.5517/ccdc.csd.cc2dfjt6	W.Clegg, J.D.Kennedy, M.J.Carr	Leeds	C9 H21 B10 Mo1 O2 1-,C14 H19 N2 1+
2217549	DEQGAL	10.5517/ccdc.csd.cc2dfjv7	W.Clegg, J.D.Kennedy, M.J.Carr	Leeds	(C1 H6 Ag1 B11 I6)n
2217552	DEQGEP	10.5517/ccdc.csd.cc2dfjyb	W.Clegg, J.D.Kennedy, J.Bould, C.A.Kilner, M.G.S.Londesborough, C.O'Dowd	Leeds	C9 H20 B10 Co2 O5 S2
2217554	DEQGIT	10.5517/ccdc.csd.cc2dfk0f	W.Clegg, J.D.Kennedy, J.Bould, U.Doerfler	Leeds	H12 B10 Cl2
2217585	MECDUV01	10.5517/ccdc.csd.cc2dfl0g	W.Clegg, J.D.Kennedy, M.J.Carr	Leeds	C28 H35 B10 N1 O1 P1 Rh1,0.5(C1 H2 Cl2)
2217586	MECDOP01	10.5517/ccdc.csd.cc2dfl1h	W.Clegg, J.D.Kennedy, M.J.Carr	Leeds	C25 H28 B9 P1
2222651	JEWHAY	10.5517/ccdc.csd.cc2dlvf9	W.Clegg, J.D.Kennedy, T.Jelinek	Leeds	C5 H33 B18 N3
2222652	JEWHEC	10.5517/ccdc.csd.cc2dlvgb	W.Clegg, J.D.Kennedy, T.Jelinek	Leeds	C5 H33 B18 N1
2222654	JEWHIG	10.5517/ccdc.csd.cc2dlvjd	W.Clegg, J.D.Kennedy, T.Jelinek	Leeds	C6 H34 B18 N2
2222655	JEWHOM	10.5517/ccdc.csd.cc2dlvkf	W.Clegg, J.D.Kennedy, T.Jelinek	Leeds	C8 H20 N1 1+,C1 H9 B9 Br1 1-
2222656	JEWJII	10.5517/ccdc.csd.cc2dlvlg	W.Clegg, J.D.Kennedy, T.Jelinek	Leeds	C8 H20 N1 1+,C1 H9 B9 Br1 1-
2222764	JEWTAK	10.5517/ccdc.csd.cc2dlz22	W.Clegg, J.D.Kennedy, T.Jelinek, J.Plesek	Leeds	C8 H23 B10 O2 1-,C4 H12 N1 1+
2228183	ZEWVUW	10.5517/ccdc.csd.cc2dslwp	W.Clegg, J.D.Kennedy, A.Franken, M.J.Carr	Leeds	C6 H15 B11 N3 O3 1-,C8 H20 N1 1+
2228209	ZEWWUX	10.5517/ccdc.csd.cc2dsmqk	W.Clegg, J.D.Kennedy, A.Franken, M.J.Carr	Leeds	C14 H17 B9 N3 O2 1-,C8 H20 N1 1+
2228229	ZEWXAE	10.5517/ccdc.csd.cc2dsnc7	W.Clegg, J.D.Kennedy, A.Franken, M.J.Carr	Leeds	C15 H17 B9 N1 O2 1-,C8 H20 N1 1+,C2 H3 N1
2228266	ZEWXEI	10.5517/ccdc.csd.cc2dspkg	W.Clegg, J.D.Kennedy, A.Franken, M.J.Carr, A.Yip	Leeds	C6 H16 B10 N3 O3 1-,C8 H20 N1 1+
2228327	ZEWXIM	10.5517/ccdc.csd.cc2dsrjh	W.Clegg, J.D.Kennedy, A.Franken, M.J.Carr, A.Yip	Leeds	C1 H14 B11 N1,C3 H6 O1
2230055	FETWIK01	10.5517/ccdc.csd.cc2dvk83	W.Clegg, J.D.Kennedy, A.Franken, M.J.Carr, A.Yip	Leeds	C4 H20 B11 N1
2230082	ZEWZIO	10.5517/ccdc.csd.cc2dvl40	W.Clegg, J.D.Kennedy, A.Franken, M.J.Carr, A.Yip	Leeds	C8 H18 B10 N2 O2
2230103	ZEWZOU	10.5517/ccdc.csd.cc2dvltp	W.Clegg, J.D.Kennedy, A.Franken, M.J.Carr, A.Yip	Leeds	C24 H20 P1 1+,C1 H13 B11 N1 1-
2232326	ZICXIW	10.5517/ccdc.csd.cc2dxxjs	W.Clegg, J.D.Kennedy, M.J.Carr,	Leeds	Cs1 1+,C7 H16 B11 1-

			A.Franken		
2232327	ZICXOC	10.5517/ccdc.csd.cc2dxxkt	W.Clegg, J.D.Kennedy, M.J.Carr, D.E.Bryant	Leeds	2(C9 H14 N1 1+),H12 B12 2-
2232328	ZICXUI	10.5517/ccdc.csd.cc2dxxlv	W.Clegg, J.D.Kennedy, M.J.Carr, D.E.Bryant	Leeds	C9 H14 N1 1+,I2,I3 1-
2232329	ZICYAP	10.5517/ccdc.csd.cc2dxxmw	W.Clegg, J.D.Kennedy, M.J.Carr	Leeds	Ag1 1+,C7 H14 B9,I1 1-
2234133	SIFGOH	10.5517/ccdc.csd.cc2dzst0	W.Clegg, R.W.Harrington	Newcastle	C19 H16 I1 O1 1+,C2 F3 O2 1-
2234135	SIFGUN	10.5517/ccdc.csd.cc2dzsw2	W.Clegg, R.W.Harrington	Newcastle	C8 H6 N2 O7,C4 H8 O2
2234136	SIFHAU	10.5517/ccdc.csd.cc2dzsx3	W.Clegg, R.W.Harrington	Newcastle	C8 H6 F1 N1 O4
2234137	SIFHEY	10.5517/ccdc.csd.cc2dzsy4	W.Clegg, R.W.Harrington	Newcastle	C13 H19 N1 O3
2234145	SIFHIC	10.5517/ccdc.csd.cc2dzt6f	W.Clegg, R.W.Harrington	Newcastle	C17 H14 I1 O1 S1 1+,C2 F3 O2 1-,C2 H3 N1
2234146	SIFHOI	10.5517/ccdc.csd.cc2dzt7g	W.Clegg, R.W.Harrington	Newcastle	C11 H13 I1 O5,0.5(C1 H2 Cl2)
2234147	IBZDAC14	10.5517/ccdc.csd.cc2dzt8h	W.Clegg, R.W.Harrington	Newcastle	C10 H11 I1 O4
2234148	IHUMOQ01	10.5517/ccdc.csd.cc2dzt9j	W.Clegg, R.W.Harrington	Newcastle	C15 H11 N1 S1
2234149	SIFJEA	10.5517/ccdc.csd.cc2dztbk	W.Clegg, R.W.Harrington	Newcastle	C16 H16 I1 O3 1+,C2 H4 O2,C2 F3 O2 1-
2234150	SIFPEG	10.5517/ccdc.csd.cc2dztcl	W.Clegg, R.W.Harrington	Newcastle	C15 H14 I1 O3 1+,C2 F3 O2 1-,C2 H3 N1
2234970	IHUMOQ02	10.5517/ccdc.csd.cc2f0nty	W.Clegg, R.W.Harrington	Newcastle	C15 H11 N1 S1
2234971	XIDKOO	10.5517/ccdc.csd.cc2f0nvz	W.Clegg, R.W.Harrington	Newcastle	C20 H18 I1 O1 1+,C2 F3 O2 1-
2234972	XIDKUU	10.5517/ccdc.csd.cc2f0nw0	W.Clegg, R.W.Harrington	Newcastle	C17 H14 F3 I1 O5
2234973	XIDLAB	10.5517/ccdc.csd.cc2f0nx1	W.Clegg, R.W.Harrington	Newcastle	C21 H16 O4
2234974	XIDLEF	10.5517/ccdc.csd.cc2f0ny2	W.Clegg, R.W.Harrington	Newcastle	C10 H6 I1 N1 O2
2234975	XIDLIJ	10.5517/ccdc.csd.cc2f0nz3	W.Clegg, R.W.Harrington	Newcastle	C13 H10 I1 O1 1+,C2 F3 O2 1-
2234976	XEBZIP01	10.5517/ccdc.csd.cc2f0p05	W.Clegg, R.W.Harrington	Newcastle	C11 H9 N1 O3
2234977	XIDLUV	10.5517/ccdc.csd.cc2f0p16	W.Clegg, R.W.Harrington	Newcastle	C17 H15 N1 O3
2237358	GICXEZ	10.5517/ccdc.csd.cc2f34vk	W.Clegg	Newcastle	C12 H9 Cl2 N3 O2,C2 H6 O1 S1
2237359	GICXID	10.5517/ccdc.csd.cc2f34wl	W.Clegg, R.W.Harrington	Newcastle	C11 H16 O2
2237360	GICXOJ	10.5517/ccdc.csd.cc2f34xm	W.Clegg, R.W.Harrington	Newcastle	C8 H12 O3 S1
2237373	GICXUP	10.5517/ccdc.csd.cc2f35b2	W.Clegg, R.W.Harrington	Newcastle	C27 H24 Cl3 N1 O3
2237375	GICYAW	10.5517/ccdc.csd.cc2f35d4	W.Clegg, R.W.Harrington	Newcastle	C11 H10 O4
2237376	GUMTIU01	10.5517/ccdc.csd.cc2f35f5	W.Clegg, R.W.Harrington	Newcastle	C10 H18 O4
2237377	GICYIE	10.5517/ccdc.csd.cc2f35g6	W.Clegg, R.W.Harrington	Newcastle	C8 H14 O4
2237378	GICYOK	10.5517/ccdc.csd.cc2f35h7	W.Clegg	Newcastle	C9 H16 O4
2237380	GICYUQ	10.5517/ccdc.csd.cc2f35k9	W.Clegg, R.W.Harrington	Newcastle	C27 H24 Cl3 N1 O4
2237381	GICZAX	10.5517/ccdc.csd.cc2f35lb	W.Clegg, R.W.Harrington	Newcastle	C27 H24 Cl3 N1 O4
2237382	GICZEB	10.5517/ccdc.csd.cc2f35mc	W.Clegg	Newcastle	C24 H34 O6 S2
2238355	NIDPAV	10.5517/ccdc.csd.cc2f460t	W.Clegg, J.D.Kennedy, M.J.Carr	Leeds	(C1 H7 Ag1 B11 Br5)n
2238407	NIDSAY	10.5517/ccdc.csd.cc2f47pj	W.Clegg, J.D.Kennedy, M.J.Carr	Leeds	(C2 H12 Ag2 B22 Br12)n
2238408	NIDSEC	10.5517/ccdc.csd.cc2f47qk	W.Clegg, J.D.Kennedy, M.J.Carr	Leeds	(C7 H14 Ag1 B9)n
2238409	NIDSIG	10.5517/ccdc.csd.cc2f47rl	W.Clegg, J.D.Kennedy, M.J.Carr	Leeds	(C7 H15 Ag1 B11 I1)n
2238410	NIDSOM	10.5517/ccdc.csd.cc2f47sm	W.Clegg, J.D.Kennedy, M.J.Carr	Leeds	(C14 H30 Ag2 B22 Br2)n
2239287	TEZHUF	10.5517/ccdc.csd.cc2f552w	W.Clegg, J.D.Kennedy, A.Franken, M.J.Carr	Leeds	C36 H30 N1 P2 1+,C3 H6 O1,H2 O1,Br1 1-
2239288	TURDAL01	10.5517/ccdc.csd.cc2f553x	W.Clegg, J.D.Kennedy, A.Franken, M.J.Carr	Leeds	C42 H42 I2 P2 Pd1

2239289	TEZJER	10.5517/ccdc.csd.cc2f554y	W.Clegg, J.D.Kennedy, A.Franken, M.J.Carr	Leeds	C36 H30 N1 P2 1+,C3 H6 O1,H2 O1,I1 1-
2240880	FIDPER	10.5517/ccdc.csd.cc2f6tgy	W.Clegg, J.D.Kennedy, A.Franken, N.J.Bullen	Leeds	C14 H22 B9 N1
2240883	FIDPAN	10.5517/ccdc.csd.cc2f6tk1	W.Clegg, J.D.Kennedy, A.Franken, N.J.Bullen	Leeds	C7 H14 B9 I1 N1 1-,C8 H20 N1 1+
2240889	FIDPIV	10.5517/ccdc.csd.cc2f6tr7	W.Clegg, J.D.Kennedy, A.Franken, N.J.Bullen	Leeds	C7 H15 B9 I1 1-,C8 H20 N1 1+
2240891	FIDPOB	10.5517/ccdc.csd.cc2f6tt9	W.Clegg, J.D.Kennedy, A.Franken, N.J.Bullen	Leeds	C8 H16 B9 I2 1-,C8 H20 N1 1+
2241379	NOLJEF01	10.5517/ccdc.csd.cc2f7bkl	W.Clegg, J.D.Kennedy, R.D.Kennedy, N.J.Bullen	Leeds	C8 H14 B1 N1
2241380	LEWWAP	10.5517/ccdc.csd.cc2f7blm	W.Clegg, J.D.Kennedy, R.D.Kennedy, N.J.Bullen	Leeds	C5 H19 B9 N2
2241381	LEWWET	10.5517/ccdc.csd.cc2f7bmn	W.Clegg, J.D.Kennedy, R.D.Kennedy, N.J.Bullen	Leeds	C5 H19 B9 N2
2241382	LEWWIX	10.5517/ccdc.csd.cc2f7bnp	W.Clegg, J.D.Kennedy, U.Doerfler, N.J.Bullen	Leeds	C3 H20 B9 Cl1 O1 S1
2241383	LEWWOD	10.5517/ccdc.csd.cc2f7bpq	W.Clegg, J.D.Kennedy, U.Doerfler, N.J.Bullen	Leeds	C2 H18 B9 Cl1 S1
2241384	LEWWUJ	10.5517/ccdc.csd.cc2f7bqr	W.Clegg, J.D.Kennedy, U.Doerfler, N.J.Bullen	Leeds	C3 H21 B9 O1 S1
2243689	WEZVAC	10.5517/ccdc.csd.cc2f9r2l	W.Clegg, J.D.Kennedy, A.Franken	Leeds	C9 H20 B11 1-,C8 H20 N1 1+
2243691	WEZVIK	10.5517/ccdc.csd.cc2f9r4n	W.Clegg, J.D.Kennedy, A.Franken	Leeds	C17 H22 B11 1-,C8 H20 N1 1+
2243692	WEZVOQ	10.5517/ccdc.csd.cc2f9r5p	W.Clegg, J.D.Kennedy, A.Franken	Leeds	C9 H20 B11 1-,C8 H20 N1 1+
2243693	WEZVUW	10.5517/ccdc.csd.cc2f9r6q	W.Clegg, J.D.Kennedy, A.Franken	Leeds	C7 H10 B11 Br6 1-,C8 H20 N1 1+
2243694	WEZWAD	10.5517/ccdc.csd.cc2f9r7r	W.Clegg, J.D.Kennedy, A.Franken	Leeds	0.896(C7 H13 B9 Br1 1-),C8 H20 N1 1+,0.104(C7 H12 B9 Br1 Cl1 1-)
2248974	YIGZAT	10.5517/ccdc.csd.cc2fh7kr	W.Clegg, J.D.Kennedy, R.D.Kennedy	Leeds	H26 B20 O1
2248975	YIGZEX	10.5517/ccdc.csd.cc2fh7ls	W.Clegg, J.D.Kennedy, R.D.Kennedy, A.Franken	Leeds	C9 H22 B10 N1 1-,C8 H20 N1 1+
2248976	YIGZIB	10.5517/ccdc.csd.cc2fh7mt	W.Clegg, J.D.Kennedy, A.Franken	Leeds	C7 H13 B9 Br1 1-,C8 H20 N1 1+
2248977	YIGZOH	10.5517/ccdc.csd.cc2fh7nv	W.Clegg, J.D.Kennedy, A.Franken	Leeds	C7 H13 B9 I1 1-,C8 H20 N1 1+
2248978	YIGZUN	10.5517/ccdc.csd.cc2fh7pw	W.Clegg, J.D.Kennedy, A.Franken	Leeds	C7 H13 B9 Cl1 1-,C8 H20 N1 1+
2249299	HEZQIQ	10.5517/ccdc.csd.cc2fhl1l	W.Clegg, J.D.Kennedy, N.J.Bullen	Leeds	C13 H20 B9 N1
2249300	HEZQOW	10.5517/ccdc.csd.cc2fhl2m	W.Clegg, J.D.Kennedy, N.J.Bullen	Leeds	C13 H24 B9 N1
2249301	HEZQUC	10.5517/ccdc.csd.cc2fhl3n	W.Clegg, J.D.Kennedy, N.J.Bullen	Leeds	C15 H24 B9 N1
2249302	HEZRAJ	10.5517/ccdc.csd.cc2fhl4p	W.Clegg, J.D.Kennedy, N.J.Bullen	Leeds	C12 H26 B10 N2,3(C6 H6)
2249303	HEZREN	10.5517/ccdc.csd.cc2fhl5q	W.Clegg, J.D.Kennedy, N.J.Bullen	Leeds	C16 H20 B9 N1,C1 H1 Cl3
2249304	HEZRIR	10.5517/ccdc.csd.cc2fhl6r	W.Clegg, J.D.Kennedy, N.J.Bullen	Leeds	C16 H24 B9 N1,0.5(C1 H1 Cl3)
2249305	HEZROX	10.5517/ccdc.csd.cc2fhl7s	W.Clegg, J.D.Kennedy, N.J.Bullen	Leeds	C14 H30 B10 N6,2(C3 H7 N1 O1)
2249306	HEZSAK	10.5517/ccdc.csd.cc2fhl8t	W.Clegg, J.D.Kennedy, R.D.Kennedy, N.J.Bullen	Leeds	C12 H16 B1 N1
2249309	NOLJEF02	10.5517/ccdc.csd.cc2fhlcx	W.Clegg, J.D.Kennedy, R.D.Kennedy, N.J.Bullen	Leeds	C8 H14 B1 N1
2249748	HIFFEL	10.5517/ccdc.csd.cc2fj1jk	W.Clegg, R.W.Harrington	Newcastle	C31 H24 Cl1 N1 Zr1,C31 H24 Br1 N1 Zr1
2249749	HIFFIP	10.5517/ccdc.csd.cc2fj1kl	W.Clegg, R.W.Harrington	Newcastle	C21 H16 N1 1+,C1 H1 Cl3,Cl1 1-
2249750	CNANTH03	10.5517/ccdc.csd.cc2fj1lm	W.Clegg, R.W.Harrington	Newcastle	C15 H9 N1

2249751	HIFFUB	10.5517/ccdc.csd.cc2fj1mn	W.Clegg, R.W.Harrington	Newcastle	C25 H22 N1 P1 S1
2249752	HIFGAI	10.5517/ccdc.csd.cc2fj1np	W.Clegg, R.W.Harrington	Newcastle	C26 H24 N1 P1 S1,C7 H8 O1
2249753	CPDCZR07	10.5517/ccdc.csd.cc2fj1pq	W.Clegg	Newcastle	C10 H10 Cl2 Zr1
2249754	HIFGIQ	10.5517/ccdc.csd.cc2fj1qr	W.Clegg, R.W.Harrington	Newcastle	C44 H56 N2 O8 P2 Zr2 4+,4(C1 H1 Cl3),4(Cl1 1-)
2249755	HIFGOW	10.5517/ccdc.csd.cc2fj1rs	W.Clegg	Newcastle	C14 H14
2249756	PAWVIU02	10.5517/ccdc.csd.cc2fj1st	W.Clegg, R.W.Harrington	Newcastle	C17 H15 N1 O2 S1
2249757	HIFHAJ	10.5517/ccdc.csd.cc2fj1tv	W.Clegg, R.W.Harrington	Newcastle	C15 H10 N2 O5 S1
2249758	MEDYOL02	10.5517/ccdc.csd.cc2fj1vw	W.Clegg	Newcastle	C27 H24 F3 N3 O5
2249759	HIFHIR	10.5517/ccdc.csd.cc2fj1wx	W.Clegg, R.W.Harrington	Newcastle	C34 H27 N3 O3
2249760	HIFHOX	10.5517/ccdc.csd.cc2fj1xy	W.Clegg, R.W.Harrington	Newcastle	C22 H19 N3 O4
2249761	HIFHUD	10.5517/ccdc.csd.cc2fj1yz	W.Clegg, R.W.Harrington	Newcastle	C22 H21 N3 O4 S1
2249762	UCEKET01	10.5517/ccdc.csd.cc2fj1z0	W.Clegg, R.W.Harrington	Newcastle	C7 H4 Br2 Cl3 N1 O1
2249763	BETPOH01	10.5517/ccdc.csd.cc2fj202	W.Clegg, R.W.Harrington	Newcastle	C6 H2 Br2 Cl3 N1 O1
2249764	WEYYUV02	10.5517/ccdc.csd.cc2fj213	W.Clegg, R.W.Harrington	Newcastle	C7 H6 Cl3 N1 O1
2249765	HIFJOZ	10.5517/ccdc.csd.cc2fj224	W.Clegg, R.W.Harrington	Newcastle	C6 H2 Cl5 N1 O1
2249892	FIDPUH	10.5517/ccdc.csd.cc2f6tvb	W.Clegg, J.D.Kennedy, A.Franken, N.J.Bullen	Leeds	C7 H13 B9 I1 1-,C8 H20 N1 1+
2256489	BIJREV	10.5517/ccdc.csd.cc2fr1z7	W.Clegg, J.D.Kennedy, N.J.Bullen	Leeds	C16 H26 B9 N1
2256490	BIJREV01	10.5517/ccdc.csd.cc2fr209	W.Clegg, J.D.Kennedy, N.J.Bullen	Leeds	C16 H26 B9 N1
2256497	BIJROF	10.5517/ccdc.csd.cc2fr27j	W.Clegg, J.D.Kennedy, N.J.Bullen	Leeds	C7 H15 B9 Cl1 1-,C8 H20 N1 1+
2256498	BIJRUL	10.5517/ccdc.csd.cc2fr28k	W.Clegg, J.D.Kennedy, N.J.Bullen	Leeds	C7 H14 B9 Cl2 1-,C8 H20 N1 1+
2256499	BIJSAS	10.5517/ccdc.csd.cc2fr29l	W.Clegg, J.D.Kennedy, N.J.Bullen	Leeds	0.842(C7 H14 B9 Cl2 1-),C8 H20 N1 1+,0.158(C7 H13 B9 Cl3 1-)
2256500	BIJSEW	10.5517/ccdc.csd.cc2fr2bm	W.Clegg, J.D.Kennedy, N.J.Bullen	Leeds	C17 H30 B9 N1,0.5(C1 H2 Cl2)
2257516	UDOYAR	10.5517/ccdc.csd.cc2fs43h	W.Clegg, J.D.Kennedy, U.Doerfler, P.A.Cooke	Leeds	C21 H35 B9 N1 Rh1,C1 H2 Cl2
2257517	UDOYEV	10.5517/ccdc.csd.cc2fs44j	W.Clegg, J.D.Kennedy, U.Doerfler, N.J.Bullen	Leeds	C4 H22 B10 Cl2 S2
2257518	UDOYIZ	10.5517/ccdc.csd.cc2fs45k	W.Clegg, J.D.Kennedy, U.Doerfler, N.J.Bullen	Leeds	H12 B10 Cl2
2257519	UDOYOF	10.5517/ccdc.csd.cc2fs46l	W.Clegg, J.D.Kennedy, U.Doerfler, N.J.Bullen	Leeds	C4 H22 B10 Cl2 S2
2257520	UDOYUL	10.5517/ccdc.csd.cc2fs47m	W.Clegg, J.D.Kennedy, U.Doerfler, N.J.Bullen	Leeds	C16 H31 B10 Cl3 P2
2257521	UDOZAS	10.5517/ccdc.csd.cc2fs48n	W.Clegg, J.D.Kennedy, U.Doerfler, N.J.Bullen	Leeds	C4 H23 B10 Cl1 S2
2258560	PILBUL	10.5517/ccdc.csd.cc2ft6s8	W.Clegg, J.D.Kennedy, N.Sriprang, P.K.Dosangh, S.J.Milne, P.A.Cooke	Leeds	C25 H36 O12 Ti2,3(C1 H1 Cl3)
2258562	PILCEW	10.5517/ccdc.csd.cc2ft6vb	W.Clegg, J.D.Kennedy, N.Sriprang, P.K.Dosangh, S.J.Milne, P.A.Cooke	Leeds	C13 H20 O6 Ti1
2261361	OFUCAX	10.5517/ccdc.csd.cc2fx44n	W.Clegg, J.D.Kennedy, U.Doerfler, N.P.Rath	Leeds	C22 H42 B7 N1 Rh2
2261466	OFUTES	10.5517/ccdc.csd.cc2fx7j4	W.Clegg, J.D.Kennedy, Y.-H.Kim, P.A.Cooke	Leeds	C27 H46 B9 Cl1 P2 Pt1 Ru1
2261467	OFUTIW	10.5517/ccdc.csd.cc2fx7k5	W.Clegg, J.D.Kennedy, Y.-H.Kim, P.A.Cooke	Leeds	C26 H45 B9 Cl1 P2 Pt1 Rh1
2261468	OFUTOC	10.5517/ccdc.csd.cc2fx7l6	W.Clegg, J.D.Kennedy, Y.-H.Kim, P.A.Cooke	Leeds	C20 H40 B8 Ru2

2261469	OFUTUI	10.5517/ccdc.csd.cc2fx7m7	W.Clegg, J.D.Kennedy, Y.-H.Kim, P.A.Cooke	Leeds	C46 H51 B9 P2 Ru1,C1 H2 Cl2
2262222	XIJLIP	10.5517/ccdc.csd.cc2fy0xb	W.Clegg, J.D.Kennedy, M.G.S.Londesborough, U.Doerfler, P.A.Cooke	Leeds	C13 H28 B8 N2
2262224	XIJLOV	10.5517/ccdc.csd.cc2fy0zd	W.Clegg, J.D.Kennedy, U.Doerfler, P.A.Cooke	Leeds	C16 H32 B8 N2
2262232	XIJLUB	10.5517/ccdc.csd.cc2fy17p	W.Clegg, J.D.Kennedy, U.Doerfler, P.A.Cooke	Leeds	C16 H32 B8 N1 P1
2262233	XIJMAI	10.5517/ccdc.csd.cc2fy18q	W.Clegg, J.D.Kennedy, U.Doerfler, P.A.Cooke	Leeds	C15 H30 B8 N1 P1
2262234	XIJMEM	10.5517/ccdc.csd.cc2fy19r	W.Clegg, J.D.Kennedy, U.Doerfler, P.A.Cooke	Leeds	C12 H24 B8 N2
2262298	XIJPUF	10.5517/ccdc.csd.cc2fy3cw	W.Clegg, J.D.Kennedy, U.Doerfler, P.A.Cooke	Leeds	C2 H20 B8 N2
2262663	XILLAJ	10.5517/ccdc.csd.cc2fyh41	W.Clegg, J.D.Kennedy, R.Macias, M.Thornton-Pett	Leeds	C39 H41 B8 O1 P2 Rh1
2262664	XILLEN	10.5517/ccdc.csd.cc2fyh52	W.Clegg, J.D.Kennedy, R.Macias, M.Thornton-Pett	Leeds	C25 H45 B9 P3 Rh1 S2
2262666	BELJEH01	10.5517/ccdc.csd.cc2fyh74	W.Clegg, J.D.Kennedy, R.Macias, M.Thornton-Pett	Leeds	C36 H44 B9 Ir1 P2
2263110	XIQLES	10.5517/ccdc.csd.cc2fyykx	W.Clegg, J.D.Kennedy, E.J.Ditzel	Leeds	C18 H38 B10 P1 Rh1
2263111	XIQLIW	10.5517/ccdc.csd.cc2fyyly	W.Clegg, J.D.Kennedy, E.J.Ditzel	Leeds	C20 H44 B9 N2 Rh1
2263113	XIQGIR	10.5517/ccdc.csd.cc2fyyn0	W.Clegg, J.D.Kennedy, E.J.Ditzel	Leeds	C16 H40 B9 N1 Ru1
2263114	XIQGOX	10.5517/ccdc.csd.cc2fyyp1	W.Clegg, J.D.Kennedy, E.J.Ditzel	Leeds	C22 H47 B9 N2 Ru1
2263684	KIJHUK	10.5517/ccdc.csd.cc2fzk22	W.Clegg, J.D.Kennedy, E.K.Newby, C.O'Dowd	Leeds	C22 H30 B10 N2
2263685	KIJJAS	10.5517/ccdc.csd.cc2fzk33	W.Clegg, J.D.Kennedy, E.K.Newby, C.O'Dowd	Leeds	C12 H20 B10 N4
2263687	KIJJOG	10.5517/ccdc.csd.cc2fzk55	W.Clegg, J.D.Kennedy, E.K.Newby, C.O'Dowd	Leeds	C13 H21 B9 N2
2263688	EXUSEY02	10.5517/ccdc.csd.cc2fzk66	W.Clegg, J.D.Kennedy, E.K.Newby, C.O'Dowd	Leeds	C10 H22 B10 N2
2263689	EXUSEY03	10.5517/ccdc.csd.cc2fzk77	W.Clegg, J.D.Kennedy, E.K.Newby, C.O'Dowd	Leeds	C10 H22 B10 N2
2265161	SIJZAQ	10.5517/ccdc.csd.cc2g12qb	W.Clegg, J.D.Kennedy, C.O'Dowd, L.M.Brown	Leeds	C11 H12 B1 N1
2265162	SIJZEU	10.5517/ccdc.csd.cc2g12rc	W.Clegg, J.D.Kennedy, C.O'Dowd, L.M.Brown	Leeds	C12 H14 B1 N1
2265164	SIJZIY	10.5517/ccdc.csd.cc2g12tf	W.Clegg, J.D.Kennedy, C.O'Dowd, L.M.Brown	Leeds	C6 H11 B1 N2
2265165	SIJZOE	10.5517/ccdc.csd.cc2g12vg	W.Clegg, J.D.Kennedy, C.O'Dowd, L.M.Brown	Leeds	C10 H14 B1 N4 1+,2(C5 H6 N2),Cl1 1-
2265168	SIKGUS	10.5517/ccdc.csd.cc2g12yk	W.Clegg, J.D.Kennedy, C.O'Dowd, L.M.Brown	Leeds	C5 H9 B1 N2
2265169	SIKHAZ	10.5517/ccdc.csd.cc2g12zl	W.Clegg, J.D.Kennedy, C.O'Dowd, L.M.Brown	Leeds	C6 H11 B1 N2
2271608	TISHOW	10.5517/ccdc.csd.cc2g7sp6	W.Clegg, J.D.Kennedy, R.S.Coldicott	Leeds	C39 H40 B9 Ir1 O3 P2,C1 H2 Cl2
2271610	TISHUC	10.5517/ccdc.csd.cc2g7sr8	W.Clegg, J.D.Kennedy, R.S.Coldicott	Leeds	C26 H49 B9 O2 P3 Re1
2281134	MIPLEG	10.5517/ccdc.csd.cc2gkpzq	W.Clegg, J.D.Kennedy, C.O'Dowd	Leeds	C14 H30 B10 N2
2281135	MIPLIK	10.5517/ccdc.csd.cc2gkq0s	W.Clegg, J.D.Kennedy, C.O'Dowd	Leeds	C18 H26 B10 N2

2281136	MIPLOQ	10.5517/ccdc.csd.cc2gkq1t	W.Clegg, J.D.Kennedy, C.O'Dowd	Leeds	C8 H20 B10 N4
2281137	MIPLUW	10.5517/ccdc.csd.cc2gkq2v	W.Clegg, J.D.Kennedy, C.O'Dowd	Leeds	C8 H20 B10 N4
2281138	MIPMAD	10.5517/ccdc.csd.cc2gkq3w	W.Clegg, J.D.Kennedy, C.O'Dowd	Leeds	C24 H34 B10 N2
2281139	MIPMEH	10.5517/ccdc.csd.cc2gkq4x	W.Clegg, J.D.Kennedy, C.O'Dowd	Leeds	C14 H26 B10 N2 O2
2281140	MIPNAE	10.5517/ccdc.csd.cc2gkq5y	W.Clegg, J.D.Kennedy, C.O'Dowd	Leeds	C5 H8 B1 N1
2281864	BMPPPT11	10.5517/ccdc.csd.cc2glgj2	W.Clegg, J.D.Kennedy, J.Bould	Leeds	C16 H40 B12 P2 Pt2
2281865	QIJZOC	10.5517/ccdc.csd.cc2glgk3	W.Clegg, J.D.Kennedy, J.Bould	Leeds	C12 H28 B18 N2 Pt1,0.5(C1 H2 Cl2)
2281866	QIJZUI	10.5517/ccdc.csd.cc2glgl4	W.Clegg, J.D.Kennedy, J.Bould, R.D.Kennedy	Leeds	C5 H15 B10 I1 O2
2281867	QIKBAR	10.5517/ccdc.csd.cc2glgm5	W.Clegg, J.D.Kennedy, J.Bould, R.D.Kennedy	Leeds	C8 H16 B10
2281868	UZUVUH01	10.5517/ccdc.csd.cc2glgn6	W.Clegg, J.D.Kennedy, J.Bould, C.M.Pask	Leeds	C32 H54 B10 P4 Pd2
2282841	QIQDON	10.5517/ccdc.csd.cc2gmh1n	W.Clegg, J.D.Kennedy, K.Nestor, M.Thornton-Pett, J.Holub	Leeds	C37 H40 B8 P2 Ru1 S1
2282843	QIQDUT	10.5517/ccdc.csd.cc2gmh3q	W.Clegg, J.D.Kennedy, K.Nestor, M.Thornton-Pett, J.Holub	Leeds	C13 H30 B9 Rh1 S1
2283083	QIRPEQ	10.5517/ccdc.csd.cc2gmqvp	W.Clegg, R.W.Harrington	Newcastle	C18 H14 Cl2 N2 O4
2283084	HUZBIN01	10.5517/ccdc.csd.cc2gmqwq	W.Clegg, R.W.Harrington	Newcastle	C7 H5 Cl3 N2 O3
2283085	QIRPOA	10.5517/ccdc.csd.cc2gmqxr	W.Clegg, R.W.Harrington	Newcastle	C16 H15 N1 O3 S1
2283086	QIRPUG	10.5517/ccdc.csd.cc2gmqys	W.Clegg, R.W.Harrington	Newcastle	C17 H16 Cl2 O3
2283087	QIRQAN	10.5517/ccdc.csd.cc2gmqzt	W.Clegg, R.W.Harrington	Newcastle	C15 H15 N1 O4
2283088	QIRQER	10.5517/ccdc.csd.cc2gmr0w	W.Clegg	Newcastle	C25 H24 Cl2 O5
2283089	QIRQIV	10.5517/ccdc.csd.cc2gmr1x	W.Clegg, R.W.Harrington	Newcastle	C16 H17 N1 O4
2283090	QIRQOB	10.5517/ccdc.csd.cc2gmr2y	W.Clegg, R.W.Harrington	Newcastle	C16 H13 N1 O3 S1
2283091	QIRQUH	10.5517/ccdc.csd.cc2gmr3z	W.Clegg, R.W.Harrington	Newcastle	C15 H14 N2 O2
2283092	CACMUP01	10.5517/ccdc.csd.cc2gmr40	W.Clegg, R.W.Harrington	Newcastle	C11 H9 N1 O3
2283093	QIRRES	10.5517/ccdc.csd.cc2gmr51	W.Clegg, R.W.Harrington	Newcastle	C20 H20 B1 Br1 F2 N2 O1
2283094	XOGDUT05	10.5517/ccdc.csd.cc2gmr62	W.Clegg, R.W.Harrington	Newcastle	C17 H23 B1 F2 N2
2285746	HIMSAB	10.5517/ccdc.csd.cc2gqhrg	W.Clegg, R.W.Harrington	Newcastle	C21 H22 B1 F2 N3 O2
2285748	HIMSEF	10.5517/ccdc.csd.cc2gqhtj	W.Clegg, R.W.Harrington	Newcastle	C22 H24 B1 Br1 F2 N2 O1
2285749	HIMSIJ	10.5517/ccdc.csd.cc2gqhvk	W.Clegg, R.W.Harrington	Newcastle	C12 H14 O3
2285750	HIMSOP	10.5517/ccdc.csd.cc2gqhwl	W.Clegg, R.W.Harrington	Newcastle	C21 H21 B1 Br1 F3 N2
2285753	HIMSUV	10.5517/ccdc.csd.cc2gqhzp	W.Clegg, R.W.Harrington	Newcastle	C13 H11 Br1 N2 O3
2285754	HIMTEG	10.5517/ccdc.csd.cc2gqj0r	W.Clegg, R.W.Harrington	Newcastle	C13 H11 Br1 F1 N1 O1
2285755	CEMFOT01	10.5517/ccdc.csd.cc2gqj1s	W.Clegg, R.W.Harrington	Newcastle	C13 H12 N2 O3
2285757	HIMNAW	10.5517/ccdc.csd.cc2gqj3v	W.Clegg, R.W.Harrington	Newcastle	C14 H8 Br2 O5
2285758	HIMNEA	10.5517/ccdc.csd.cc2gqj4w	W.Clegg, R.W.Harrington	Newcastle	C13 H12 F1 N1 O1
2285763	UHISOV01	10.5517/ccdc.csd.cc2gqj91	W.Clegg, R.W.Harrington	Newcastle	C23 H18 Cl2 N2 O7
2285765	HIMNUQ	10.5517/ccdc.csd.cc2gqjc3	W.Clegg, R.W.Harrington	Newcastle	C25 H27 B1 F2 N2 O3
2285767	HIMPAY	10.5517/ccdc.csd.cc2gqjf5	W.Clegg, R.W.Harrington	Newcastle	C22 H21 N1 O2
2285768	HIMPEC	10.5517/ccdc.csd.cc2gqjg6	W.Clegg, R.W.Harrington	Newcastle	C20 H20 N2 O5
2285770	HIMPIG	10.5517/ccdc.csd.cc2gqjj8	W.Clegg, R.W.Harrington	Newcastle	C27 H22 N2 O4 S1
2285772	HIMPOM	10.5517/ccdc.csd.cc2gqjlb	W.Clegg, R.W.Harrington	Newcastle	C13 H11 Cl2 N1 O2

2285776	HIMPUS	10.5517/ccdc.csd.cc2gqjqg	W.Clegg, R.W.Harrington	Newcastle	C19 H19 Cl2 N1 O4
2285777	HIMQAZ	10.5517/ccdc.csd.cc2gqjrh	W.Clegg, R.W.Harrington	Newcastle	C15 H17 N1 O2
2285778	HIMQED	10.5517/ccdc.csd.cc2gqjsj	W.Clegg, R.W.Harrington	Newcastle	C16 H19 N1 O2
2285779	HIMQIH	10.5517/ccdc.csd.cc2gqjtk	W.Clegg	Newcastle	C26 H28 B1 F3 N2 O2
2285780	HIMTOQ	10.5517/ccdc.csd.cc2gqjvl	W.Clegg, R.W.Harrington	Newcastle	C14 H14 Br1 N1 O2
2285781	HIMTUW	10.5517/ccdc.csd.cc2gqjwm	W.Clegg, R.W.Harrington	Newcastle	C14 H15 N1 O2
2285782	HIMVAE	10.5517/ccdc.csd.cc2gqjxn	W.Clegg, R.W.Harrington	Newcastle	C27 H31 B1 F2 N2 O3
2285783	HIMVEI	10.5517/ccdc.csd.cc2gqjyp	W.Clegg, R.W.Harrington	Newcastle	C22 H24 B1 F2 I1 N2 O1
2285784	HIMVIM	10.5517/ccdc.csd.cc2gqjzq	W.Clegg, R.W.Harrington	Newcastle	C20 H19 B1 F2 I2 N2 O1
2285785	HIMVOS	10.5517/ccdc.csd.cc2gqk0s	W.Clegg, R.W.Harrington	Newcastle	C29 H31 B1 F2 N2 O2
2285786	HIMVUY	10.5517/ccdc.csd.cc2gqk1t	W.Clegg, R.W.Harrington	Newcastle	C20 H21 B1 F2 N2 O1
2285787	HIMWAF	10.5517/ccdc.csd.cc2gqk2v	W.Clegg, R.W.Harrington	Newcastle	C20 H19 B1 Br2 F2 N2 O1
2285791	HIMWEJ	10.5517/ccdc.csd.cc2gqk6z	W.Clegg, R.W.Harrington	Newcastle	C33 H32 N2 O7 S1
2285792	HIMWIN	10.5517/ccdc.csd.cc2gqk70	W.Clegg, R.W.Harrington	Newcastle	C17 H15 N1 O1
2285793	HIMWOT	10.5517/ccdc.csd.cc2gqk81	W.Clegg, R.W.Harrington	Newcastle	C6 H4 Br2 O3
2285794	HIMWUZ	10.5517/ccdc.csd.cc2gqk92	W.Clegg, R.W.Harrington	Newcastle	C6 H6 Br4 O3
2285799	HIMXAG	10.5517/ccdc.csd.cc2gqkg7	W.Clegg, R.W.Harrington	Newcastle	C28 H31 N3 O7 S1
2285800	HIMXEK	10.5517/ccdc.csd.cc2gqkh8	W.Clegg, J.D.Kennedy, M.Thornton-Pett, J.Holub, K.Nestor	Leeds	C24 H44 B9 P3 Ru1 S1
2285804	HIMXIO	10.5517/ccdc.csd.cc2gqkmd	W.Clegg, J.D.Kennedy, M.Thornton-Pett, J.Holub, K.Nestor	Leeds	C39 H46 B10 Ir1 O2 P2 1-,C6 H16 N1 1+
2285805	HINCEQ	10.5517/ccdc.csd.cc2gqknf	W.Clegg, J.D.Kennedy, K.Nestor, J.Holub	Leeds	C11 H23 B8 Cl1 Ir1 1-,C14 H19 N2 1+
2286342	HIRGIC	10.5517/ccdc.csd.cc2gr3zb	W.Clegg, J.D.Kennedy, C.O'Dowd	Leeds	C10 H18 B10 Br4 N2
2286343	HIRGOI	10.5517/ccdc.csd.cc2gr40d	W.Clegg, J.D.Kennedy, C.O'Dowd	Leeds	C10 H20 B10 Br2 N2
2286344	HIRGUO	10.5517/ccdc.csd.cc2gr41f	W.Clegg, J.D.Kennedy, C.O'Dowd	Leeds	C7 H7 N1 O1
2286345	HIRHAV	10.5517/ccdc.csd.cc2gr42g	W.Clegg, J.D.Kennedy, C.O'Dowd, M.Thornton-Pett	Leeds	C6 H11 B1 N2
2286346	HIRHEZ	10.5517/ccdc.csd.cc2gr43h	W.Clegg, J.D.Kennedy, C.O'Dowd, M.Thornton-Pett	Leeds	C14 H30 B10 N2
2286347	HIRHID	10.5517/ccdc.csd.cc2gr44j	W.Clegg, J.D.Kennedy, C.O'Dowd, M.Thornton-Pett	Leeds	C18 H26 B10 N2,C3 H7 N1 O1
2286348	HIRHOJ	10.5517/ccdc.csd.cc2gr45k	W.Clegg, J.D.Kennedy, C.O'Dowd, M.G.S.Londesborough	Leeds	C11 H12 B1 N1
2290956	DINSOM	10.5517/ccdc.csd.cc2gwxt3	W.Clegg, J.D.Kennedy, J.Bould, C.A.Kilner	Leeds	C32 H66 B20 O1 P4 Pt2,C1 H2 Cl2
2290958	DINTAZ	10.5517/ccdc.csd.cc2gwxw5	W.Clegg, J.D.Kennedy, J.Bould, C.A.Kilner	Leeds	C16 H32 B10 Cl2 P2 Pt1,C1 H2 Cl2
2290959	DINTED	10.5517/ccdc.csd.cc2gwxx6	W.Clegg, J.D.Kennedy, J.Bould, C.A.Kilner	Leeds	C16 H32 B10 Cl2 P2 Pt1
2290960	DINTIH	10.5517/ccdc.csd.cc2gwxy7	W.Clegg, J.D.Kennedy, J.Bould, C.A.Kilner	Leeds	C6 H30 B10 P2 Pt1
2290961	DINTON	10.5517/ccdc.csd.cc2gwxz8	W.Clegg, J.D.Kennedy, J.Bould, C.A.Kilner	Leeds	C20 H31 B10 N2 P1 Pd1
2297679	GITXAM	10.5517/ccdc.csd.cc2h3xp7	W.Clegg, J.D.Kennedy, C.O'Dowd, C.M.Pask	Leeds	C18 H38 B10 N2

2297681	GITXEQ	10.5517/ccdc.csd.cc2h3xr9	W.Clegg, J.D.Kennedy, C.O'Dowd, C.M.Pask	Leeds	C8 H20 B10 N4
2297682	VIZBIQ01	10.5517/ccdc.csd.cc2h3xsb	W.Clegg, J.D.Kennedy, C.O'Dowd, C.M.Pask	Leeds	C12 H20 B10 N4,C1 H2 Cl2
2297683	GITXOA	10.5517/ccdc.csd.cc2h3xtc	W.Clegg, J.D.Kennedy, C.O'Dowd, C.M.Pask	Leeds	C16 H34 B10 N2
2299906	TISZII	10.5517/ccdc.csd.cc2h67jh	W.Clegg, J.D.Kennedy, M.Thornton-Pett, B.Stibr, J.H.Jones	Leeds	C36 H36 B8 P2 Pt1 S1,0.5(C1 H2 Cl2)
2299907	TISZOO	10.5517/ccdc.csd.cc2h67kj	W.Clegg, J.D.Kennedy, M.Thornton-Pett, B.Stibr, J.H.Jones	Leeds	C36 H39 B8 N1 P2 Pt1
2299908	TISZUU	10.5517/ccdc.csd.cc2h67lk	W.Clegg, J.D.Kennedy, M.Thornton-Pett, B.Stibr, J.H.Jones	Leeds	C16 H30 B8 P2 Pt1 S1
2299909	MOBLUL01	10.5517/ccdc.csd.cc2h67ml	W.Clegg, J.D.Kennedy, P.A.Cooke, Y.H.Kim	Leeds	C17 H34 B8 P2 Pt1
2301267	SIVQUN	10.5517/ccdc.csd.cc2h7nfv	W.Clegg, J.D.Kennedy, M.Thornton-Pett, B.Stibr, J.H.Jones	Leeds	C38 H40 B8 P2 Pt1
2301268	SIVRAU	10.5517/ccdc.csd.cc2h7ngw	W.Clegg, J.D.Kennedy, M.Thornton-Pett, B.Stibr, J.H.Jones	Leeds	C18 H31 B7 P2 Pt1
2301271	SIVREY	10.5517/ccdc.csd.cc2h7nkz	W.Clegg, J.D.Kennedy, M.Thornton-Pett, B.Stibr, J.H.Jones	Leeds	C38 H41 B9 P2 Pt1,0.5(C1 H2 Cl2)
2301273	SIVRIC	10.5517/ccdc.csd.cc2h7nm1	W.Clegg, J.D.Kennedy, J.Bould, C.M.Pask	Leeds	C6 H30 B10 P2 Pd1 S1
2302615	FIWREM	10.5517/ccdc.csd.cc2h91xs	W.Clegg, J.D.Kennedy, A.Franken, M.J.Carr	Leeds	C10 H14 B9 Cl2 N4 1-,C8 H20 N1 1+,C3 H6 O1
2302616	AWIPOL01	10.5517/ccdc.csd.cc2h91yt	W.Clegg, J.D.Kennedy, A.Franken	Leeds	C9 H21 B11 S1
2302617	FIWROW	10.5517/ccdc.csd.cc2h91zv	W.Clegg, J.D.Kennedy, A.Franken, M.J.Carr, N.J.Bullen	Leeds	C7 H12 B9 Br1 I1 1-,C8 H20 N1 1+
2302926	FIXYEU	10.5517/ccdc.csd.cc2h9cy4	W.Clegg, J.D.Kennedy, A.Franken	Leeds	C7 H11 B9 I3 1-,C8 H20 N1 1+
2303335	FOBSIC	10.5517/ccdc.csd.cc2h9t4s	W.Clegg, R.W.Harrington	Newcastle	C26 H25 N3 O5 S2
2303336	MSORHA01	10.5517/ccdc.csd.cc2h9t5t	W.Clegg, R.W.Harrington	Newcastle	C16 H32 O10 Rh2 S2
2303337	ILICEM01	10.5517/ccdc.csd.cc2h9t6v	W.Clegg, R.W.Harrington	Newcastle	(C8 H18 Br1 K1 O11 Rh2)n,H2 O1
2303338	FOBTAV	10.5517/ccdc.csd.cc2h9t7w	W.Clegg, R.W.Harrington	Newcastle	C12 H24 Cu1 N10 2+,O4 S1 2-,C1 H4 O1,4.5(H2 O1)
2303339	FOBTEZ	10.5517/ccdc.csd.cc2h9t8x	W.Clegg, R.W.Harrington	Newcastle	C20 H36 Cl4 N16 Pd3 2+,2(Cl1 1-)
2303342	FOBTID	10.5517/ccdc.csd.cc2h9tc0	W.Clegg, R.W.Harrington	Newcastle	3(C9 H12 N2 O9 P1 1-),3(C9 H11 N2 O9 P1 2-),9(Na1 1+),28(H2 O1)
2303343	THGUAN11	10.5517/ccdc.csd.cc2h9td1	W.Clegg, R.W.Harrington	Newcastle	C5 H5 N5 S1
2303344	NUVQIH01	10.5517/ccdc.csd.cc2h9tf2	W.Clegg, R.W.Harrington	Newcastle	C20 H36 Ag2 N18 O6
2303345	FOBVAX	10.5517/ccdc.csd.cc2h9tg3	W.Clegg, R.W.Harrington	Newcastle	C34 H28 N2 S4
2303346	FOBVAX01	10.5517/ccdc.csd.cc2h9th4	W.Clegg, R.W.Harrington	Newcastle	C34 H28 N2 S4
2303347	GUWPAS01	10.5517/ccdc.csd.cc2h9tj5	W.Clegg, R.W.Harrington	Newcastle	C12 H15 N7,2(H2 O1)
2303348	FOBYEE	10.5517/ccdc.csd.cc2h9tk6	W.Clegg, R.W.Harrington	Newcastle	C11 H18 Cl1 N6 Pd1 S2 1+,Cl1 1-
2303351	FOBYII	10.5517/ccdc.csd.cc2h9tn9	W.Clegg, R.W.Harrington	Newcastle	(C21 H25 Ag3 N6 O14)n,2(H2 O1)
2303473	THGUAN12	10.5517/ccdc.csd.cc2h9ylc	W.Clegg, R.W.Harrington	Newcastle	C5 H5 N5 S1
2304090	UJEYEO02	10.5517/ccdc.csd.cc2hblhy	W.Clegg, J.D.Kennedy, M.G.S.Londesborough, B.Stibr	Leeds	C7 H16 B9 Co1
2304092	LIYBUU	10.5517/ccdc.csd.cc2hblk0	W.Clegg, J.D.Kennedy, R.Macias	Leeds	C24 H30 N2 O2

2304093	LIYCAB	10.5517/ccdc.csd.cc2hbll1	W.Clegg, J.D.Kennedy, J.Bould, C.A.Kilner, C.M.Pask	Leeds	C32 H66 B20 O1 P4 Pt2,C1 H1 Cl3
2304203	LIZFIN	10.5517/ccdc.csd.cc2hbq4q	W.Clegg, J.D.Kennedy, B.Stibr, J.H.Jones, T.Jelinek	Leeds	C18 H26 B12 P1
2306310	TBUAWO11	10.5517/ccdc.csd.cc2hdx3y	W.Clegg, R.W.Harrington	Newcastle	2(C16 H36 N1 1+),O19 W6 2-
2306312	WODPAK	10.5517/ccdc.csd.cc2hdx50	W.Clegg, R.W.Harrington	Newcastle	3(C16 H36 N1 1+),H1 O19 Sn1 W5 3-
2306313	WODPEO	10.5517/ccdc.csd.cc2hdx61	W.Clegg, R.W.Harrington	Newcastle	3(C16 H36 N1 1+),H1 O19 Sn1 W5 3-,C2 H3 N1
2306315	WODPOY	10.5517/ccdc.csd.cc2hdx83	W.Clegg, R.W.Harrington	Newcastle	4(C16 H36 N1 1+),H2 O37 Ti2 W10 4-
2306322	TBUAWO12	10.5517/ccdc.csd.cc2hdxhb	W.Clegg, R.W.Harrington	Newcastle	2(C16 H36 N1 1+),O19 W6 2-
2326554	FUQFOM01	10.5517/ccdc.csd.cc2j2z4r	W.Clegg, J.D.Kennedy, R.Macias	RM82B	C11 H17 B10 N1 O2
2326555	IXOGUX01	10.5517/ccdc.csd.cc2j2z5s	W.Clegg, J.D.Kennedy, R.Macias	RM82C	C11 H21 B10 N1 O2
2330037	POMMAJ	10.5517/ccdc.csd.cc2j6lhv	W.Clegg, J.D.Kennedy, D.Cundy, R.D.Kennedy, N.J.Bullen	Leeds	C8 H14 B1 N1 O1
2330039	POMMEN	10.5517/ccdc.csd.cc2j6lkx	W.Clegg, J.D.Kennedy, D.Cundy, R.D.Kennedy, N.J.Bullen	Leeds	C4 H14 B1 N1
2404480	TOJYIA01	10.5517/ccdc.csd.cc2lq1w8	W.Clegg, J.D.Kennedy, A.Franken, P.A.Cooke	Leeds	C14 H19 N2 1+,C1 H13 B8 1-
2404482	SUGYUS	10.5517/ccdc.csd.cc2lq1yb	W.Clegg, J.D.Kennedy, M.Thornton-Pett, B.Stibr	Leeds	1.02(C2 H8 B8 Br2),0.98(C2 H9 B8 Br1)
2404839	SUJZAC	10.5517/ccdc.csd.cc2lqfg7	W.Clegg, J.D.Kennedy, M.Thornton-Pett, K.Nestor	Leeds	C20 H40 B9 Cl1 Ir1 Rh1
2425895	DURFUV	10.5517/ccdc.csd.cc2mfbp3	W.Clegg, R.W.Harrington	Newcastle	3(C16 H36 N1 1+),C3 H9 O19 Si1 Sn1 W5 3-,0.5(C2 H3 N1)
2425896	DURGAC	10.5517/ccdc.csd.cc2mfbq4	W.Clegg, R.W.Harrington	Newcastle	4(C16 H36 N1 1+),O36 Ti2 W10 4-,2(C2 H3 N1)
2425897	EJUTUB01	10.5517/ccdc.csd.cc2mfbr5	W.Clegg, R.W.Harrington	Newcastle	C16 H36 N1 1+,C2 H5 N1 O1,Cl1 1-
2425898	DURGIK	10.5517/ccdc.csd.cc2mfbs6	W.Clegg, R.W.Harrington	Newcastle	4(C16 H36 N1 1+),O37 Sn2 W10 4-,2(C6 H14 O1),2(C2 H3 N1)
2425900	DURGOQ	10.5517/ccdc.csd.cc2mfbv8	W.Clegg, R.W.Harrington	Newcastle	(H8 Cs2 Mo12 O44 P1 2-)n,2(C16 H36 N1 1+)
2425901	REVLOU01	10.5517/ccdc.csd.cc2mfbw9	W.Clegg, R.W.Harrington	Newcastle	4(C16 H36 N1 1+),O32 W10 4-,2(C2 H3 N1)
2425902	DURHAD	10.5517/ccdc.csd.cc2mfbxb	W.Clegg, R.W.Harrington	Newcastle	C16 H36 N1 1+,C15 H11 N3 O1,C15 H10 N3 O1 1-
2444195	CUGJAP02	10.5517/ccdc.csd.cc2n1d04	W.Clegg, L.Horsburgh, R.E.Mulvey, S.A.Couper	Strathclyde	C36 H58 Li2 O4
2444196	TUYMOT	10.5517/ccdc.csd.cc2n1d15	W.Clegg, L.Horsburgh, R.E.Mulvey, M.J.Ross	Strathclyde	C22 H50 Mg1 N6 O6 P2
2444197	TUYMUZ	10.5517/ccdc.csd.cc2n1d26	W.Clegg, L.Horsburgh, R.E.Mulvey, H.Nothe	Strathclyde	C44 H60 Li4 O8
2444198	TUYNAG	10.5517/ccdc.csd.cc2n1d37	W.Clegg, L.Horsburgh, R.E.Mulvey, F.M.Mackenzie	Strathclyde	C24 H44 Li2 N6
2444199	TUYNEK	10.5517/ccdc.csd.cc2n1d48	W.Clegg, L.Horsburgh, R.E.Mulvey, F.M.Mackenzie	Strathclyde	C36 H60 Li2 N2 O4
2444200	TUYNIO	10.5517/ccdc.csd.cc2n1d59	W.Clegg, L.Horsburgh, R.E.Mulvey, F.M.Mackenzie	Strathclyde	C32 H60 Li2 N6
2444385	REMHAS01	10.5517/ccdc.csd.cc2n1l4g	W.Clegg, L.Horsburgh, R.E.Mulvey, M.J.Ross	Strathclyde	C23 H29 N3
2446165	LUPZOP	10.5517/ccdc.csd.cc2n3fks	W.Clegg, L.Horsburgh, R.E.Mulvey, F.J.Craig	Strathclyde	C16 H32 Li1 N4 1+,I1 1-
2446169	LUPZOP01	10.5517/ccdc.csd.cc2n3fpx	W.Clegg, L.Horsburgh, R.E.Mulvey, F.J.Craig	Strathclyde	C16 H32 Li1 N4 1+,I1 1-
2446172	LUQBAE	10.5517/ccdc.csd.cc2n3fs0	W.Clegg, L.Horsburgh, R.E.Mulvey,	Strathclyde	C21 H27 I1 Li1 N3

			F.J.Craig		
2446173	LUQBEI	10.5517/ccdc.csd.cc2n3ft1	W.Clegg, L.Horsburgh, R.E.Mulvey, F.J.Craig	Strathclyde	C17 H34 Li1 N4 1+,I1 1-
2446174	LUQBIM	10.5517/ccdc.csd.cc2n3fv2	W.Clegg, L.Horsburgh, R.E.Mulvey, F.J.Craig	Strathclyde	C9 H25 Li1 N3 O1 1+,I1 1-
2446175	BOFVAT01	10.5517/ccdc.csd.cc2n3fw3	W.Clegg, L.Horsburgh, R.E.Mulvey, F.J.Craig	Strathclyde	C36 H48 Li4 N4
2446176	LUQBUY	10.5517/ccdc.csd.cc2n3fx4	W.Clegg, L.Horsburgh, R.E.Mulvey, F.J.Craig	Strathclyde	C17 H34 Li1 N4 O1 1+,I1 1-
2446177	LUQCAF	10.5517/ccdc.csd.cc2n3fy5	W.Clegg, L.Horsburgh, R.E.Mulvey, F.J.Craig	Strathclyde	C32 H44 Cl2 Li2 N4
2446179	LUQCEJ	10.5517/ccdc.csd.cc2n3g08	W.Clegg, L.Horsburgh, R.E.Mulvey, F.M.Mackenzie, D.M.Lindsay	Strathclyde	C32 H80 Li8 N8
2455116	FUSJUC	10.5517/ccdc.csd.cc2ndr94	W.Clegg, L.Horsburgh, R.E.Mulvey, R.Konradi	Strathclyde	C60 H114 K2 Li2 N12 Na2 O2
2455117	FUSKAJ	10.5517/ccdc.csd.cc2ndrb5	W.Clegg, L.Horsburgh, R.E.Mulvey, R.Garrioch	Strathclyde	C22 H40 Li2 N6
2455118	FUSKEN	10.5517/ccdc.csd.cc2ndrc6	W.Clegg, R.E.Mulvey, A.M.Drummond	Strathclyde	C30 H78 Li6 N12,C4 H8 O2
2455119	FUSKIR	10.5517/ccdc.csd.cc2ndrd7	W.Clegg, R.E.Mulvey, F.J.Craig	Strathclyde	C13 H34 Al1 K1 N2 O1
2455120	FUSKOX	10.5517/ccdc.csd.cc2ndrf8	W.Clegg, R.E.Mulvey, F.J.Craig	Strathclyde	C34 H88 Al4 K2 N6 O4
2455121	RAPQEE02	10.5517/ccdc.csd.cc2ndrg9	W.Clegg, P.N.O'Shaughnessy, R.E.Mulvey, F.J.Craig	Strathclyde	C20 H46 Al2 Li2 N2 O2
2455122	FUSLAK	10.5517/ccdc.csd.cc2ndrhb	W.Clegg, R.E.Mulvey, R.B.Rowlings	Strathclyde	C24 H72 Mg1 N12 O4 P4 2+,2(C4 H12 Al1 1-)
2455123	FUSLEO	10.5517/ccdc.csd.cc2ndrjc	W.Clegg, A.J.Edwards, R.E.Mulvey, F.J.Craig	Strathclyde	C12 H32 N4 Na1 1+,C4 H12 Al1 1-
2455124	FUSMUF01	10.5517/ccdc.csd.cc2ndrkd	W.Clegg, P.N.O'Shaughnessy, R.E.Mulvey, F.M.Mackenzie	Strathclyde	C26 H48 Li2 N6
2455125	FUSLOY	10.5517/ccdc.csd.cc2ndrlf	W.Clegg, P.N.O'Shaughnessy, R.E.Mulvey, F.J.Craig	Strathclyde	(C11 H20 Al1 Na1 O1)n
2455126	FUSLUE	10.5517/ccdc.csd.cc2ndrmg	W.Clegg, P.N.O'Shaughnessy, R.E.Mulvey, A.M.Drummond	Strathclyde	C40 H64 N10 Rb2
2455130	FUSMAL	10.5517/ccdc.csd.cc2ndrrl	W.Clegg, S.T.Liddle, R.E.Mulvey, A.M.Drummond	Strathclyde	(C63 H74 N8 Rb2)n
2455131	FUSMEP	10.5517/ccdc.csd.cc2ndrsm	W.Clegg, S.T.Liddle, R.E.Mulvey, A.M.Drummond	Strathclyde	(C32 H29 N4 Rb1)n
2455133	FUSMIT	10.5517/ccdc.csd.cc2ndrvp	W.Clegg, S.T.Liddle, R.E.Mulvey, A.Robertson	Strathclyde	C42 H72 Li1 N10 Na3
2455134	FUSMUF	10.5517/ccdc.csd.cc2ndrwq	W.Clegg, S.T.Liddle, R.E.Mulvey, A.Robertson	Strathclyde	C26 H48 Li2 N6
2455135	FUSNAM	10.5517/ccdc.csd.cc2ndrxr	W.Clegg, S.T.Liddle, R.E.Mulvey, R.B.Rowlings	Strathclyde	C42 H42 Mg1 N4 O2 S2,C7 H8
2455139	FUSNEQ	10.5517/ccdc.csd.cc2nds1x	W.Clegg, S.T.Liddle, R.E.Mulvey, R.B.Rowlings	Strathclyde	C50 H66 Mg1 N10 O2 P2,C7 H8
2479377	EJIMET	10.5517/ccdc.csd.cc2p6zxt	W.Clegg, S.T.Liddle, R.E.Mulvey, A.M.Drummond	Strathclyde	(C42 H98 Li8 N2 O10 Rb2)n,C6 H14
2479378	EJIMIX	10.5517/ccdc.csd.cc2p6zyv	W.Clegg, S.T.Liddle, R.E.Mulvey, A.Robertson	Strathclyde	C32 H60 N6 Na2
2479379	EJIMIX01	10.5517/ccdc.csd.cc2p6zzw	W.Clegg, S.T.Liddle, R.E.Mulvey,	Strathclyde	C32 H60 N6 Na2

			A.Robertson		
2479381	EJIMUJ	10.5517/ccdc.csd.cc2p7010	W.Clegg, S.T.Liddle, R.E.Mulvey, A.Robertson	Strathclyde	C32 H60 Li2 N6
2479382	EJINAQ	10.5517/ccdc.csd.cc2p7021	W.Clegg, S.T.Liddle, R.E.Mulvey, A.Robertson	Strathclyde	(C56 H88 Li4 N4 O8)n,2(C4 H8 O2)
2479383	EJINEU	10.5517/ccdc.csd.cc2p7032	W.Clegg, S.T.Liddle, R.E.Mulvey, A.Robertson	Strathclyde	C32 H62 N8 Na2
2479384	EJINIY	10.5517/ccdc.csd.cc2p7043	W.Clegg, S.T.Liddle, R.E.Mulvey, A.Robertson	Strathclyde	C40 H48 N6 Na2
2479385	EJINOE	10.5517/ccdc.csd.cc2p7054	W.Clegg, S.T.Liddle, R.E.Mulvey, A.Robertson	Strathclyde	C38 H74 N8 Na2
2479386	EJINUK	10.5517/ccdc.csd.cc2p7065	W.Clegg, S.T.Liddle, R.E.Mulvey, A.M.Drummond	Strathclyde	C42 H98 N2 Na7 O10 Rb3
2479388	EJIPAS	10.5517/ccdc.csd.cc2p7087	W.Clegg, S.T.Liddle, R.E.Mulvey, A.M.Drummond	Strathclyde	C36 H82 Li8 O10 Rb2,C7 H8
2479391	EJIPEW	10.5517/ccdc.csd.cc2p70cb	W.Clegg, S.T.Liddle, R.E.Mulvey, A.M.Drummond	Strathclyde	C36 H82 K4 Na6 O10,C7 H8
2479393	EJIPIA	10.5517/ccdc.csd.cc2p70fd	W.Clegg, S.T.Liddle, R.E.Mulvey, A.M.Drummond, P.Jetter	Strathclyde	C48 H108 Cl1 Li6 Na7 O12,C24 H54 Li6 O6
2479394	EJIPOG	10.5517/ccdc.csd.cc2p70gf	W.Clegg, S.T.Liddle, R.E.Mulvey, J.G.MacLellan	Strathclyde	C26 H48 N6 Na2 O2
2479395	JILCEP01	10.5517/ccdc.csd.cc2p70hg	W.Clegg, S.T.Liddle, R.E.Mulvey, A.M.Drummond, C.O'Hara	Strathclyde	C40 H88 K4 Li4 O8
2479396	EJIQAT	10.5517/ccdc.csd.cc2p70jh	W.Clegg, S.T.Liddle, R.E.Mulvey, J.G.MacLellan	Strathclyde	C5 H11 O1 1-,C4 H8 O1,2(K1 1+),C7 H7 1-
2479397	EJIQEX	10.5517/ccdc.csd.cc2p70kj	W.Clegg, S.T.Liddle, R.E.Mulvey, J.G.MacLellan	Strathclyde	C6 H16 N2,C9 H11 1-,Na1 1+
2486979	EKOJEX	10.5517/ccdc.csd.cc2pgx47	W.Clegg, D.M.Tooke, R.E.Mulvey, G.Forbes	Strathclyde	C21 H47 K1 N2 Si4 Zn1
2486980	EKOJIB	10.5517/ccdc.csd.cc2pgx58	W.Clegg, D.M.Tooke, R.E.Mulvey, R.B.Rowlings	Strathclyde	C45 H87 N7 O2 Si8 Sn2
2486981	EKODUH	10.5517/ccdc.csd.cc2pgx69	W.Clegg, D.M.Tooke, R.E.Mulvey, G.Forbes	Strathclyde	C18 H54 N3 Si6 Zn1 1-,C12 H32 Li1 N4 1+
2486982	EKOFAP	10.5517/ccdc.csd.cc2pgx7b	W.Clegg, D.M.Tooke, R.E.Mulvey, G.Forbes	Strathclyde	C16 H45 Li1 N2 Si4 Zn1
2486984	EKOFET	10.5517/ccdc.csd.cc2pgx9d	W.Clegg, S.H.Dale, R.E.Mulvey, G.Honeyman	Strathclyde	C21 H38 N2 O1 Zn1
2486985	CAXGIS01	10.5517/ccdc.csd.cc2pgxbf	W.Clegg, S.H.Dale, R.E.Mulvey, E.Hevia	Strathclyde	C26 H34 Fe2 N2 Zn1
2486988	EKOFOD	10.5517/ccdc.csd.cc2pgxfj	W.Clegg, S.H.Dale, R.E.Mulvey, E.Hevia	Strathclyde	C18 H54 N3 Si6 Zn1 1-,C18 H48 K1 N6 1+
2486989	EKOFUJ	10.5517/ccdc.csd.cc2pgxgk	W.Clegg, S.H.Dale, R.E.Mulvey, L.M.Hogg	Strathclyde	C27 H52 N3 Na1 O1 Zn1
2486990	EKIYOQ	10.5517/ccdc.csd.cc2pgxhl	W.Clegg, S.H.Dale, R.E.Mulvey, L.M.Hogg, G.Honeyman	Strathclyde	C25 H52 N1 Na1 O2 Zn1
2486991	EKIYUW	10.5517/ccdc.csd.cc2pgxjm	W.Clegg, S.H.Dale, R.E.Mulvey, G.E.Hevia	Strathclyde	C26 H50 N3 Na1 O1 Zn1
2486996	EKIZEH	10.5517/ccdc.csd.cc2pgxps	W.Clegg, S.H.Dale, R.E.Mulvey, G.Honeyman	Strathclyde	C30 H52 N3 Na1 O1 Zn1
2486999	EKIZIL	10.5517/ccdc.csd.cc2pgxsw	W.Clegg, S.H.Dale, R.E.Mulvey,	Strathclyde	C23 H52 N3 Na1 O1 Zn1

			E.Hevia		
2487000	EKIZOR	10.5517/ccdc.csd.cc2pgxtx	W.Clegg, G.S.Nichol, R.E.Mulvey, L.M.Hogg	Strathclyde	C33 H44 F9 N4 Na1 Zn1
2487050	EKIZAD	10.5517/ccdc.csd.cc2pgzfl	W.Clegg, S.H.Dale, R.E.Mulvey, L.M.Hogg	Strathclyde	C27 H52 N3 Na1 O1 Zn1
2502177	OQIDEB	10.5517/ccdc.csd.cc2pzqdt	W.Clegg, G.S.Nichol, R.E.Mulvey, L.M.Hogg	Strathclyde	C27 H52 N3 Na1 O1 Zn1
2502178	OQIDIF	10.5517/ccdc.csd.cc2pzqfv	W.Clegg, S.H.Dale, R.E.Mulvey, E.Hevia	Strathclyde	C32 H48 Fe2 N4 Zn2
2502179	OQIDOL	10.5517/ccdc.csd.cc2pzqgw	W.Clegg, S.H.Dale, R.E.Mulvey, L.M.Hogg	Strathclyde	C28 H55 N4 Na1 Zn1
2502180	OQIDUR	10.5517/ccdc.csd.cc2pzqhx	W.Clegg, S.H.Dale, R.E.Mulvey, L.M.Hogg	Strathclyde	C40 H64 N8 Na2 Zn1
2502181	OQIFAZ	10.5517/ccdc.csd.cc2pzqjy	W.Clegg, G.S.Nichol, R.E.Mulvey, L.M.Hogg	Strathclyde	C28 H54 N3 Na1 O1 Zn1
2502182	OQIFED	10.5517/ccdc.csd.cc2pzqkz	W.Clegg, R.W.Harrington, R.E.Mulvey, L.M.Hogg	Strathclyde	C24 H62 N4 Na2 O4 Zn2
2502185	OQIFIH	10.5517/ccdc.csd.cc2pzqn2	W.Clegg, R.W.Harrington, R.E.Mulvey, L.M.Hogg	Strathclyde	C54 H96 N4 Na2 Zn2,C10 H8
